# An ictogenic marker in the mesial temporal epilepsy and its temporal evolutionary features

**DOI:** 10.3389/fneur.2024.1510108

**Published:** 2025-01-10

**Authors:** Hongjuan Lu, Haoran Yang, Wei Zhang, Xingzhou Liu, Wei Sun

**Affiliations:** ^1^Department of Neurology, Xuanwu Hospital Capital Medical University, Beijing, China; ^2^Department of Neurology, Beijing Luhe Hospital, Capital Medical University, Beijing, China; ^3^Department of Neurology, Beijing Tsinghua Changgung Hospital, School of Clinical Medicine, Tsinghua University, Beijing, China; ^4^Epilepsy Center, Shanghai Deji Hospital, Qingdao University, Shanghai, China

**Keywords:** hypersynchronous pattern, meisal temporal seizure, ictogensis, dynamic evolution, preictal changes

## Abstract

**Objective:**

To observe and measure the morphological and temporal evolutionary features of the hypersynchronous (HYP) pattern in the mesial temporal seizure.

**Methods:**

The HYP patterns during preictal and interictal states of 16 mesial temporal epileptic patients were analyzed. The wave components of the HYP transients were firstly observed and measured. The dynamic deformations and parameter changes of the components were further analyzed along the preictal-ictal axis. The difference of emergence rate of HYP transients and typical interictal spike during interictal periods was also compared.

**Results:**

The HYP transients were invariably composed of slow-wave proper, sharp wave and post-slow component among studied patients for all the 93 seizures. During preictal epoch, all of the seizures incorporated present evolutionary manner of type 1 characterized by smooth modification of HYP transients in morphology, including gradual shortening of the inter-transient interval, increase of amplitude and time duration of slow-wave proper and sharp wave, amplitude decrease of the post-slow component, as well as amplitude increases of ripple and fast ripple, and 2/3 seizures showed some more sophisticated transitional manners (type 2) following type 1, including reduction in amplitude with decrease of inter-transient intervals, superimposed or followed by the emergent low amplitude rhythmic activities, or both of them. The HYP transients and typical interictal spikes were found to mutually “repelling” each other in interictal period.

**Conclusion:**

The HYP transients showed a combinational feature and temporal evolution manners during preictal state. The emergence of HYP transients in cluster reflects the transitional trend from interictal to ictal state.

**Significance:**

HYP should be viewed as an index of ictogenesis in the mesial temporal seizure.

## 1 Introduction

The epileptic brain is known to have an enduring predisposition to generate seizures. Thus, the epileptic brain exists in two states, interictal and ictal, respectively. The former is usually featured by the interictal discharges, which are commonly characterized by repeated, localized single spike, spike–wave, or polyspikes. In contrast, the latter is in general characterized by the emergence of full “ictal” electrical events, with gradual or abrupt temporal and spatial evolution (1). Despite this condition, from the viewpoint of clinical practice, it is somewhat arbitrary to define “a precise point of time” where a switch between interictal and ictal states (1, 2). From a dynamical perspective, there should be an unstable state (preictal state) separating interictal to ictal state and reflecting the seizure proneness of epileptic brain (2, 3). A deep understanding the transitional mechanisms will be helpful to predict and hamper disabling seizures.

Extensive clinical and experimental evidence have indicated two types of intracranial electroencephalography onset patterns in mesial temporal lobe epilepsy (MTLE) referred to as hypersynchronous (HYP) and low voltage fast activity (LVFA) respectively. HYP, characterized by low-frequency, high-amplitude broad spike having a frequency under 2 Hz and lasting more than 5 s, is associated with restricted distribution of the epileptogenic zone marked by hippocampal sclerosis prominently (4, 5). On the other hand, LVFA is featured by high-frequency, low-amplitude discharges over 10 Hz and spreading more widely over polar and lateral temporal lobe, insular and peri-sylvian, or orbitofrontal cortex (4, 5).

*In vitro* evidence suggest that various biological mechanisms contribute to the two distinct onset patterns. LVFA is probably mediated by a local increase in potassium concentration associated with synchronized interneural firing (6). HYP may reflect a complicated interplay of excitatory and inhibitory mechanisms (7, 8). In contrast to the universality of LVFA as onset pattern in limbic and neocortical epileptic seizure, HYP is highly correlated with mesial temporal lobe seizure and has been viewed as one type of preictal discharges preceding ictal-like events (8). There are much more difficulties on the mechanism study for HYP pattern under clinical condition.

Herein, we present the detailed observation about the morphological features and temporal evolutionary manner of the HYP pattern in MTLE patients. We hypothesized that (1) the HYP pattern recorded under clinical condition is homologous, at least in part, with the HYP patterns obtained from the brain slice in rat or human studies; (2) the HYP pattern reflects the ictogenic tend trigged by epileptogenic zone during both interictal and preictal state. These viewpoints provide a new perspective for clinical practice.

## 2 Materials and methods

### 2.1 Patient cohort

The 16 patients included in the present study were derived from a population of 148 consecutive patients with medically intractable seizures who underwent stereo-electroencephalography (SEEG) at the Epilepsy Center of Shanghai Deji Hospital from March 2017 to December 2019. This study was approved by the Ethics Committee of Shanghai Deji Hospital and the Ethics Committee of Xuanwu Hospital, Capital Medical University. The inclusion criteria were as follows: (i) SEEG confirmed the epileptogenicity of the mesial temporal structures, including hippocampus, amygdala, temporal polar and/or peri-hippocampal cortices (9); (ii) the patients achieved good outcome (Engel class I or II) from resectional surgery that targeted mesial temporal structures prominently, with a minimum of 3-year follow-up; (iii) HYP pattern was explored in mesial temporal structures from at least one habitual seizure during SEEG monitoring.

### 2.2 Intracerebral signal sampling and recording

In order to sample cortical signal and further to localize the epileptogenic zone, multiple contacts (Huake Hengsheng Medical Technology Co., Ltd. (China) or Alcis (France); length: 2 mm, diameter: 0.8 mm, 1.5 mm apart) intracerebral electrodes were implanted over temporal and related cortices according to Talairach’s stereotactic method with assistance of the ROSA robot (Medtech, Montpellier, France). A post-operative computerized tomography (CT) scan without contrast was then performed to verify the absence of bleeding and location of the electrodes. Finally, a co-registration of preoperative MRI and postoperative CT images was used to locate each contact along the electrode trajectory. In all the 16 patients, 206 multiple-contact electrodes were implanted, among which 1835 contacts located in the grey matter (the mesial temporal structures: 353; the temporal neocortex: 775; the extratemporal regions: 707). Among the 353 contacts implanted in the mesial temporal structures, 116 contacts located in the amygdala (ipsilateral 82, contralateral 34), 77 in the anterior hippocampi (ipsilateral 45, contralateral 32), 18 located in the posterior hippocampi (ipsilateral 18, contralateral 0); 102 in the parahippocampal areas (ipsilateral 97, contralateral 5); 40 in the mesial part of the temporal polar (ipsilateral 40, contralateral 0).

The SEEG signal was high-pass filtered at 0.16 Hz, low-pass filtered at 600 Hz, sampled at 2000 Hz and recorded via a 256-channel Nihon Kohden EEG monitoring system (Shanghai Kohden Medical Instrument Co. Ltd). A referential montage (the reference being a contact located in white matter on the same electrode with the recording contacts) was used during signal acquisition.

### 2.3 SEEG data selection and signal analysis

#### 2.3.1 Ictal, preictal, and interictal epochs selection

**Figure 1:**
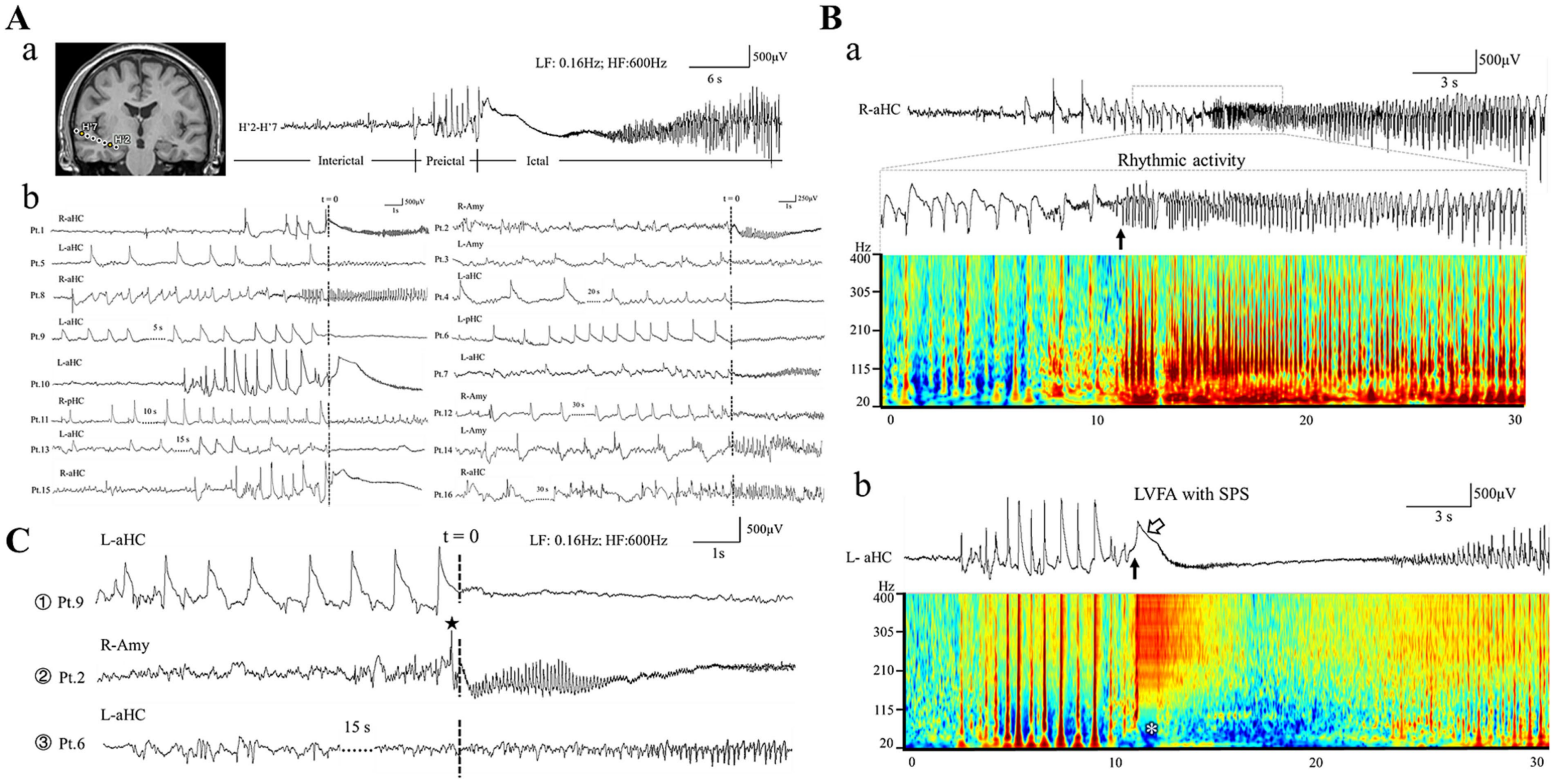
Morphology of ictal and preictal patterns. **(Aa)** Shows an example of interictal-preictal-ictal continuity recorded from human hippocampus. The exact locations of each contact are shown in left insert (MRI slice). **(Ab)** Presents typical HYP patterns from the 16 patients. Note that “t = 0” present the electrical onset of the ictal tonic discharges. The ictal pattern of rhythmic spikes followed by HYP is shown in panel **(Ba)**. The electrical onset is showed by the black arrow (the inset below). Note all the spikes span wide frequency bands in the time-frequency plot. **(Bb)** Indicates the ictal pattern of low voltage fast activity (LVFA, the black arrow) with a slow polarizing shift (SPS, marked by the hollow arrow). Note the slow polarizing shift corresponds the low frequency suppression labeled by the white asterisk in the time-frequency plot. **(C)** Shows the three types of preictal pattern: ① HYP pattern; ② sentinel spike; ③ non-specific pattern. Note that “*t* = 0” present the electrical onset of the ictal tonic discharges. Amy, amygdala; aHC, anterior hippocampus; pHC, posterior hippocampus; L, left; R, right.

Firstly, all the 132 seizures (3 to 22 per patient) of the 16 patients recorded from SEEG monitoring were selected for overall characteristics observation. Secondly, in order to reduce sampling bias, 3–5 seizures of each patient were selected for detailed analysis of HYP morphological features, and a total of 60 seizures were included. Each preictal epoch was limited within the time window spanning from seconds to tens of seconds, defined by the morphological modulation before an electrical onset (see below). Typical HYP patterns and the consequent tonic discharges of the 16 patients are shown in Figure 1A. In order to uncover the temporal incompatibility between the HYP transients and typical interictal spikes (IIS), we sampled multiple interictal epochs and observed the emergence rate of the two distinct discharges. For each individual, we randomly selected 20 non-consecutive interictal epochs lasting for 1 min and incorporated them into further processing.

#### 2.3.2 Identification of ictal and preictal patterns

Based on previous studies, we identified two distinct tonic discharge patterns as the electrical onset among all the included seizures (4, 10). The first one was low-voltage fast activity (LVFA) superimposing on an initial slow polarizing shift (SPS) (Figure 1B); whereas the second was characterized by the progressive build-up of a low- to medium-voltage sharply contoured rhythmic activity (RA) without association with an initial SPS (Figure 1B) (10, 11). Subsequently, we categorized the preictal discharges occurring seconds to tens of seconds before the tonic patterns into 2 types. Type 1, known as hypersynchronous activity (HYP), was defined as a low-frequency, high-amplitude rhythmic transients with frequency below 2 Hz, lasting more than 5 s (Figure 1C). Type 2 was characterized by a “sentinel” spike preceding the initiation of tonic discharges (Figure 1C) (4, 5). In the present study, we focus on the morphological features, wave components of HYP transient, and its temporal evolutionary manner.

#### 2.3.3 Observing and measuring the wave components of HYP pattern

For each individual, only the mesial structure manifesting the most prominent HYP transients during the preictal stages was selected for analysis. We carefully observed wave components of each transient during each preictal epoch and measured the amplitude and duration of each component as well as the duration of inter-transient intervals.

We also investigated the high frequency oscillations (HFOs) during each preictal epoch. HFOs were visually inspected based on the widely accepted definition and methods (12). In brief, we displayed finite impulse response (FIR) filters to eliminate ringing at the time resolution of approximately 1.0 s per screen. Ripples (80–250 Hz) and fast ripples (250–600 Hz) were marked separately over their respective screen. An event was only regarded as an HFO if it consisted of at least four oscillatory cycles above baseline activity. The amplitude of HFOs was defined as the voltage difference between the highest peak and lowest trough in a series of oscillations.

#### 2.3.4 Temporal evolution of HYP pattern in preictal periods

To elucidate the temporal transition dynamics of mesial temporal seizures, we carefully measured the parameter changes of each HYP transient along the preictal-ictal axis, including the amplitude, duration of each wave component and width of inter-transient intervals. The serial number of each transient was decided on their proximity to the onset of tonic ictal discharges. The nearest one preceding the onset was labeled as T (−1), the nearest one preceding T (−1) was labeled as T (−2); and the nearest one preceding T (−2) was labeled as T (−3) … By analogy, the inter-transient time intervals were named as Ti (−1), Ti (−2), Ti (−3), … Each incorporated seizure was analyzed independently, and finally the overall summary would be made.

#### 2.3.5 Comparison of the emergence between HYP transients and IISs during interictal epochs

An interesting thing is that the “rudimentary HYP pattern” (not evolved into a true seizure), which morphologically resembled the true HYP occurring during preictal epoch, emerged frequently during interictal periods in all 16 patients. We hypothesized that the interictal “HYP transient” was fundamentally different and even to certain degree incompatible with typical IIS during interictal periods (13). Hence, we visually inspected typical IIS and interictal “HYP transient” in interictal epochs. Twenty 1-min interictal epochs (10 in sleep and 10 in wakefulness) with at least five epileptic discharges were randomly incorporated. We excluded the time epochs within 6 h before and after seizure to avoid the interference on the emergence of interictal epileptic discharges by seizures. The number of interictal “HYP transients” and IISs per minute was counted separately.

### 2.4 Statistical analysis

Continuous variables were summarized using the median and range. Categorical variables were summarized using counts and percentages. When analyzing the evolutionary trend of HYP in the preictal period toward tonic discharges, we conducted linear analysis on the data of each seizure and determined it according to the r value. When *r* > 0, it was considered as increasing, and when *r* < 0, it was considered as decreasing. Spearman correlation analysis was used to explore the link between two sets of data. The level of significance was set at *p* < 0.05.

## 3 Results

The clinical features of the 16 patients (*n* = 8, 50% males; median age: 28 years, range 13–47 years) with drug-resistant temporal lobe epilepsy of varied etiologies are summarized in Table 1.

**Table 1:** Clinical characteristics of the included patients.

Patients	Sex/age (years)	Chronological semiology	Scaple EEG discharge location	MRI abnormalities	FDG-PET	No. of electrodes (in brackets) and implantation site	EZ and Surgery	Engel (f/u months)
1	f/47	Aura (palpitation) → OAAs, eyes blinking → vocalization → tachycardia → L dystonia	R T and F	R T pole and T med T2H	*R Amy, T med, Ins and basal ganglia	(14) Amy, T pole, Ins, ACC, OFC, T lat, and F pole, L and R; Hc, paraHc, RSC, MCC, and LPFC, R	R T ant-med, Ins and OFC	Ia (44)
2	f/19	Aura (palpitation) → nausea, vomiting → OAAs → L facial TCS	R hemisphere	R T lat-med and B Ins T2H	R T and Ins	(11) Hc, T pole, and T lat, L and R; Amy, paraHc, Ins, SMG, and rolandic region, R	R T ant-med	Ia (60)
3	f/44	OAAs, B HA → dystonia (B hands, face) → L deviation	B T	L T pole and T med T2H	B T and Ins	(14) Amy, Hc, Ins, OFC, T lat, LPFC, and rolandic region, L and R; T pole, and paraHc, L	L T ant-med	Ia (47)
4	f/29	Aura (deja vu) → OAAs, B HA → L deviation → tachycardia	L T	L T pole, T med and FuG T2H	L T pole, T med, and FuG	(9) Amy, Hc, paraHc, T pole, Ins, OFC, RSC, T lat, F pole and LPFC, L	L T ant-med	Ia (49)
5	m/21	Aura (fear and palpitation) → verbalization → OAAs, B HA → R hand dystonia	L T	L T pole and T med T2H	L T pole, T med, and Ins	(13) Amy, Ins, T lat, and LPFC, L and R; Hc, paraHc, T pole, OFC，ACC, RSC, and SMG, L	L T ant-med	Ia (43)
6	m/28	Aura (jamais vu) → OAAs，B HA	L T	L T pole and T med T2H	L T pole, T med, and Ins	(11) Hc, T pole, Ins, and T lat, L and R; Amy, paraHc, and OFC, L	L T ant-med and Ins	Ia (66)
7	m/33	Aura (epigastric → B deafness) → tachycardia → L gazing with nystagmus →R hand dystonia	L T	L Hc atrophy; L F pole encephalomalacia	L T and Ins operculum	(14) Hc, Ins, and T lat, L and R; Amy, paraHc, OFC, LPFC, and rolandic region, L	L T ant-med and Ins	Ia (52)
8	f/28	OAAs → R deviation →L hand dystonia →L facial TCS → tachycardia	R hemisphere	R T med T2H; R hemispheric diffuse atrophy	R hemispheric diffuse lesions	(15) Amy, Hc, paraHc, T pole, Ins, OFC, T lat, LPFC, SPL, and O, R	R T ant-med	Ia (49)
9	m/18	Aura (fear, palpitation) → motionless →L HA	L T	L T med and Ins T2H	L T pole, T med and Ins	(12) Hc, T pole, Ins, and T lat, L and R; Amy, paraHc, OFC, and LPFC, L	L T ant-med	Ia (31)
10	m/31	Aura (blank in the mind) → eyes blinking → OAAs → coughing	L T	L T pole loss of grey-white demarcating; L Amy swelling	L T pole, T med and perisylvian areas	(13) Amy, Hc, paraHc, and T lat, L and R; T pole, Ins, OFC, LPFC, and rolandic region, L	L T ant-med and Ins	Ia (38)
11	m/34	Aura (deja vu) → OAAs, B HA → flushing, tachycardia → verbalization	R T	R T and Ins T2H	R T, Ins, and F	(15) Amy, Hc, T pole, Ins, and T lat, L and R; paraHc, OFC, ACC, PCC, and LPFC, R	R T ant-med	Ia (56)
12	m/23	Aura (nausea, epigastric) → tachycardia, hypersalivation, apnea →OAAs	R T	R T pole and T med T2H	R T pole and T med, B Ins and OFR	(14) Hc, paraHc, T pole, T lat, SMG, and rolandic region, L and R; Amy, T pole, Ins, and PCC, R	R T ant-med	IIb (51)
13	f/29	Aura (epigastric) → OAAs, B HA → R hand dystonia → hypersalivation	Diffused	L Hc and B Ins T2H	L T and Ins	(10) Amy, Hc, paraHc, Ins, OFC, RSC, T lat, LPFC, rolandic region and SMG, L	L T ant-med	Ib (63)
14	m/13	Aura (nausea) → OAAs, B HA, eyes blinking → B facial dystonia → hypersalivation, tachycardia	L T	L T pole and T med T2H	B T and Ins	(12) Amy, Hc, and T lat, L and R; paraHc, T pole, Ins, OFC, RSC, T pole, LPFC and rolandic region, L	L. T. ant-med and Ins	Ia (47)
15	f/26	Aura (palpitation) → tachycardia → OAAs	R T and F	R T ant and Ins T2H	R T ant and Ins	(13) Amy, Hc, paraHc, T pole, Ins, OFC, T lat, F pole, LPFC and rolandic region, R	R T ant-med and Ins	Ia (57)
16	m/29	Aura (piloerection, epigastric) → tachycardia → OAAs → L deviation → B hands dystonia	R T	B T med and R Ins T2H; B F encephalomalacia	R T pole, T med, and Ins;	(16) Amy, Hc, paraHc, Ins, and T lat, L and R; OFC, ACC, RSC and LPFC, R	R T ant-med	Ia (62)

The lesions in PDG-PET for all patients were hypometabolic, except for patient 1 with hypermetabolic, which may be related to the ictal period.

ACC, anterior cingulate cortex; Amy, amygdala; ant, anterior; B, Bilateral; F, frontal; f, female; FuG, fusiform gyrus; f/u, follow-up; HA, hand automatism; Hc, hippocampus; Ins, Insula; lat, lateral; L, left; med., medial; m, male; MCC, middle cingulate cortex; T, temporal; T2H, High signal intensity on T_2_ -weighted MRI; O, occipital; OAA, oroalimentary automatism; OFC, orbitofrontal cortex; PCC, posterior cingulate cortex; R, right; RSC, Retrosplenial cortex; SMG, supramarginal gyrus.

### 3.1 Preictal-ictal patterns of mesial temporal epileptic seizures

**Figure 2:**
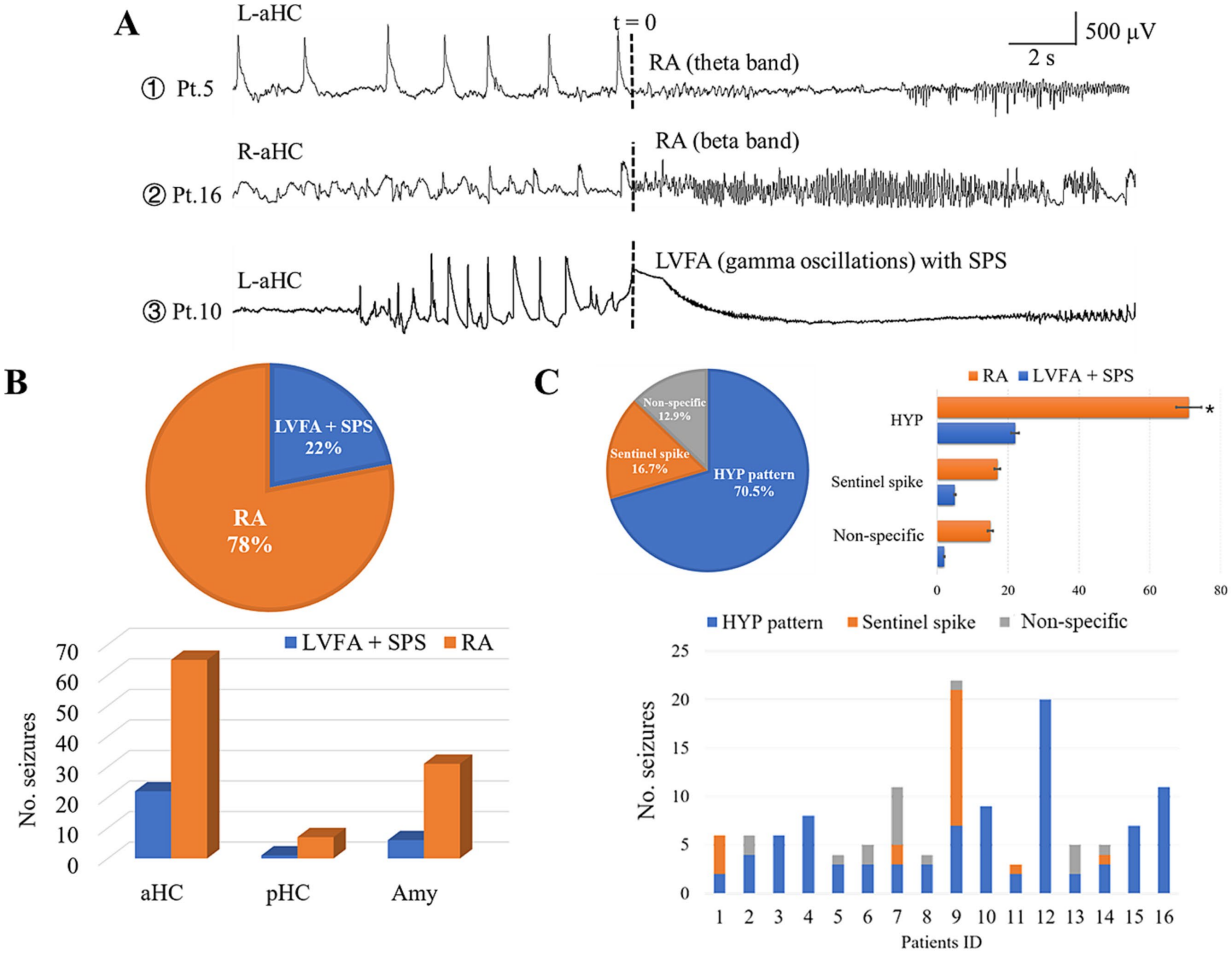
Ictal and preictal pattern correlations. **(A)** Shows the examples of the distinct ictal patterns obtained from patient 5, 10, and 16, respectively. **(B)** reveals the proportional dominance of RA pattern (78%, the upper pie chart) and hippocampus implication (the lower bar graph). **(C)** Shows the occurrence of preictal patterns. The upper pie chart indicates that the HYP pattern was the most prominent one out of the three preictal patterns. The upper bar graph shows that each preictal pattern could evolved into both RA and LVFA pattern (HYP: 71 into RA, 22 into LVFA; sentinel spike: 17 into RA, 5 into LVFA; non-specific: 15 into RA, 2 into LVFA). The lower bar graph shows the preictal pattern in each patient. Note that 10 out of the 16 patients showed more than one type of preictal patterns. LVFA, low voltage fast activity; SPS, slow polarizing shift; RA, rhythmic activity; Amy, amygdala; aHC, anterior hippocampus; pHC, posterior hippocampus; L, left; R, right.

The seizure-onset zones of all the 132 seizures incorporated were restricted within the mesial temporal structures: the anterior hippocampi (87, 65.9%), the amygdala (37, 28.0%), and posterior hippocampi (8, 6.1%). According to our definition of the onset pattern, 103 seizures (78%) presented the pattern of RA, and the remaining 29 seizures (22%) manifested the pattern of LVFA with SPS (Figure 2B). The frequency range of the former commonly spanned from theta to beta band (10 ~ 30 Hz), rarely in gamma band (30 ~ 80 Hz) (Figure 2A). The anterior hippocampus was the most active area among the mesial temporal regions, being implicated in 65 RA seizures and 22 LVFA + SPS seizures (Figure 2B).

HYP was the overwhelming preictal pattern (93 out of 132 seizures, 70.5%), following which 71 (76.3%) evolved into RA seizure while the remaining (22 seizures, 23.7%) developed into the LVFA pattern (Figure 2C). In contrast, only 22 seizures (16.7%) out of all 132 seizures presented a sentinel spike just before its tonic onset pattern, in which the RA pattern and LVFA pattern were 17 and 5, respectively, (Figure 2C).

The minority of seizures (17 seizures, 12.9% in all) displayed the third preictal pattern not specifically defined by us (non-specific pattern, Figure 1C). It was featured by the manifestation that the epileptic seizure activity gradually and smoothly built up with the disappearance of background activity and interictal discharges. The majority (15 out of 17 seizures) of this preictal pattern evolved into RA pattern (Figure 2C).

Ten out of the 16 individuals exhibited two or three types of preictal patterns (Figure 2C). It was noticeable that the preictal pattern of both HYP and “sentinel” spikes trended to develop into the RA, instead of LVFA, pattern.

### 3.2 Component and morphological analysis of HYP transients

Sixty out of 93 HYP seizures were selected for component and morphological analysis of HYP transients, with no more than five seizures incorporated for each of the 16 individuals. For each preictal epoch, the initial three transients were chosen for observation and measurement, avoiding interference to morphological analysis caused by their deformation along the time axis during each preictal epoch. Thus, a total of 176 HYP transients from the selected 60 seizures were incorporated.

**Figure 3:**
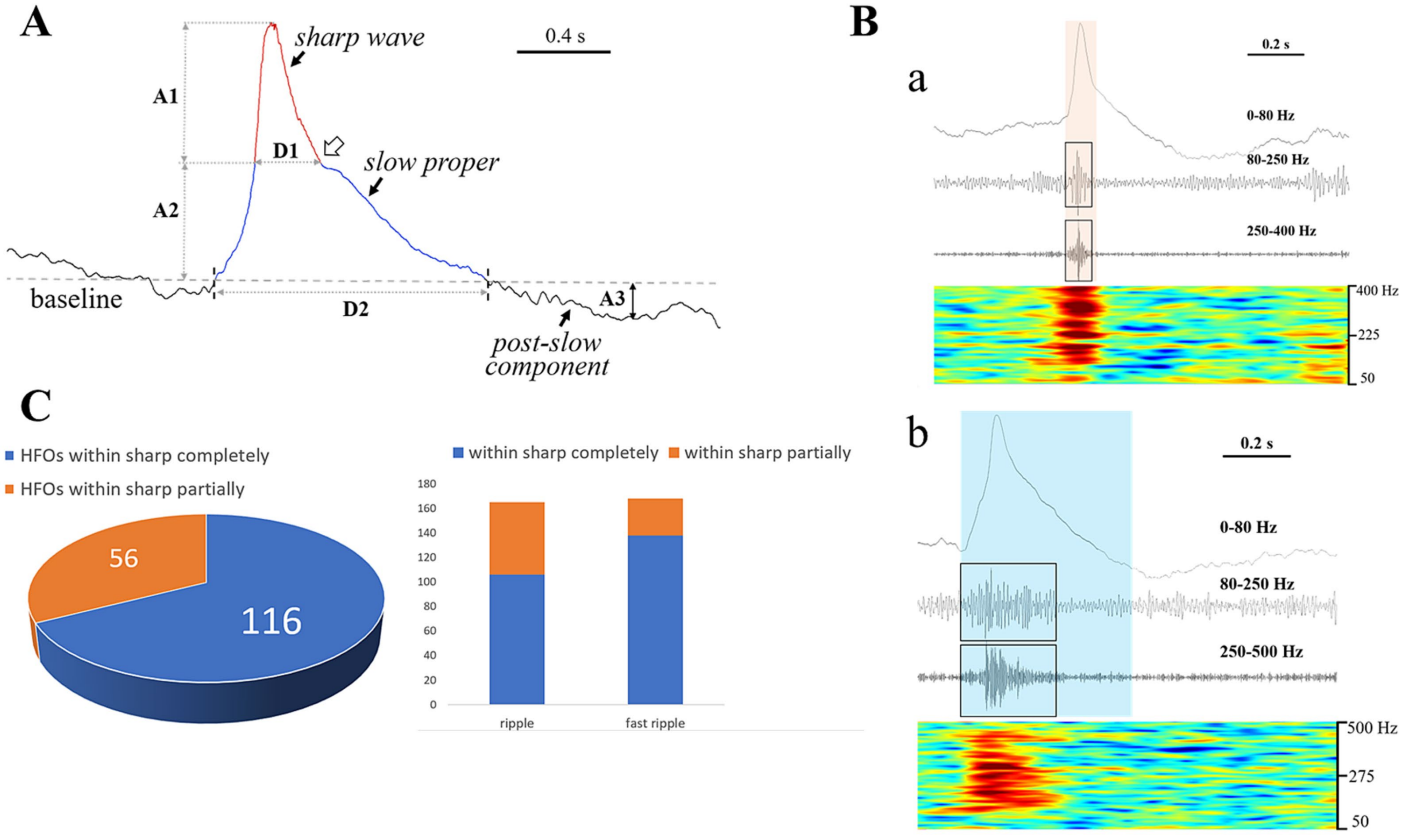
Component analysis of HYP pattern and associated HFOs. **(A)** Shows the morphological feature of a typical HYP transient. A sharp wave (rea) rode on a broad plateau termed “slow proper” (blue). The joint between the descending branch of the sharp wave and the slow proper presents a downward arciform or a notched appearance (the hollow arrow). A post-slow component with opposite polarity followed the slow-sharp complex (SSC). The amplitude and duration are represented by A and D (1, sharp wave; 2, slow proper; 3, post-slow component). **(B)** Indicates the accompanying HFOs. **(a)** Shows that the HFO (both of the ripple and fast ripple components, labeled by black boxes) were restricted by the time course of sharp wave (the yellow translucent rectangle). **(b)** Shows that the HFO events (labeled by black boxes) were limited within the time course of SSC (the blue translucent rectangle). Note that the HFO events showed a “spindle” or “diamond” appearance. **(C)** All the HFO events were almost limited within the time course of the SSC, with the majority (116, 67.4%) restricted by the sharp wave. Note that the fast ripple component matched with sharp wave much well than the ripples.

According to our detailed visual observations, the typical HYP transients always stood out against the background and were marked by combinational features: (1) a sharp wave rode on a slow-wave proper inducing a downward arciform or a notched appearance (Figure 3A); (2) HFOs commonly superimposed on a single HYP transient (Figure 3B). Thus, the HYP transient was termed as the slow-sharp complex (SSC) in the present study. In addition, there was a post-slow component following each of the SSCs, the polarity of which was opposite to SSC (Figure 3A). The measured parameters of HYP transients are listed in Table 2.

**Table 2:** The measured parameters of HYP transients.

	Duration (ms)		Amplitude (μV)	
	Median	Range	Median	Range
Slow-wave proper	409.0	64.5 ~ 1423.5	511.1	39.1 ~ 2842.7
Sharp wave	62.5	15.0 ~ 243.5	369.9	23.0 ~ 3670.5
Post-slow component	–	–	120.7	774.7 ~ 1026.1

A total of 172 out of the 176 HYP transients were combined with HFOs, among which 165 had ripple component (80–250 Hz) and 168 had fast ripple component (> 250 Hz). All the HFOs (duration: 12.6 ~ 588.0 ms) were restricted within the time course of the SSCs, with the majority (116, 67.4%) superimposed on the sharp waves (Figures 3B,C). Most of the HFOs showed a spindle shape: the amplitude of sequential oscillatory cycles reached the peak within milliseconds to hundreds of milliseconds and then decreased equally quickly (Figure 3B). The median HFO amplitude was 49.6 μV (range 10.2 ~ 676.5 μV).

### 3.3 Morphological evolution of HYP transients along the preictal-ictal axis

All the 956 SSCs obtained from the preictal epochs of the 60 HYP seizures were incorporated for observation and analysis of the evolutionary features. The morphology of SSCs was significantly modified during the seizure evolution, deriving from which the transitional patterns could be classified into two types.

**Figure 4:**
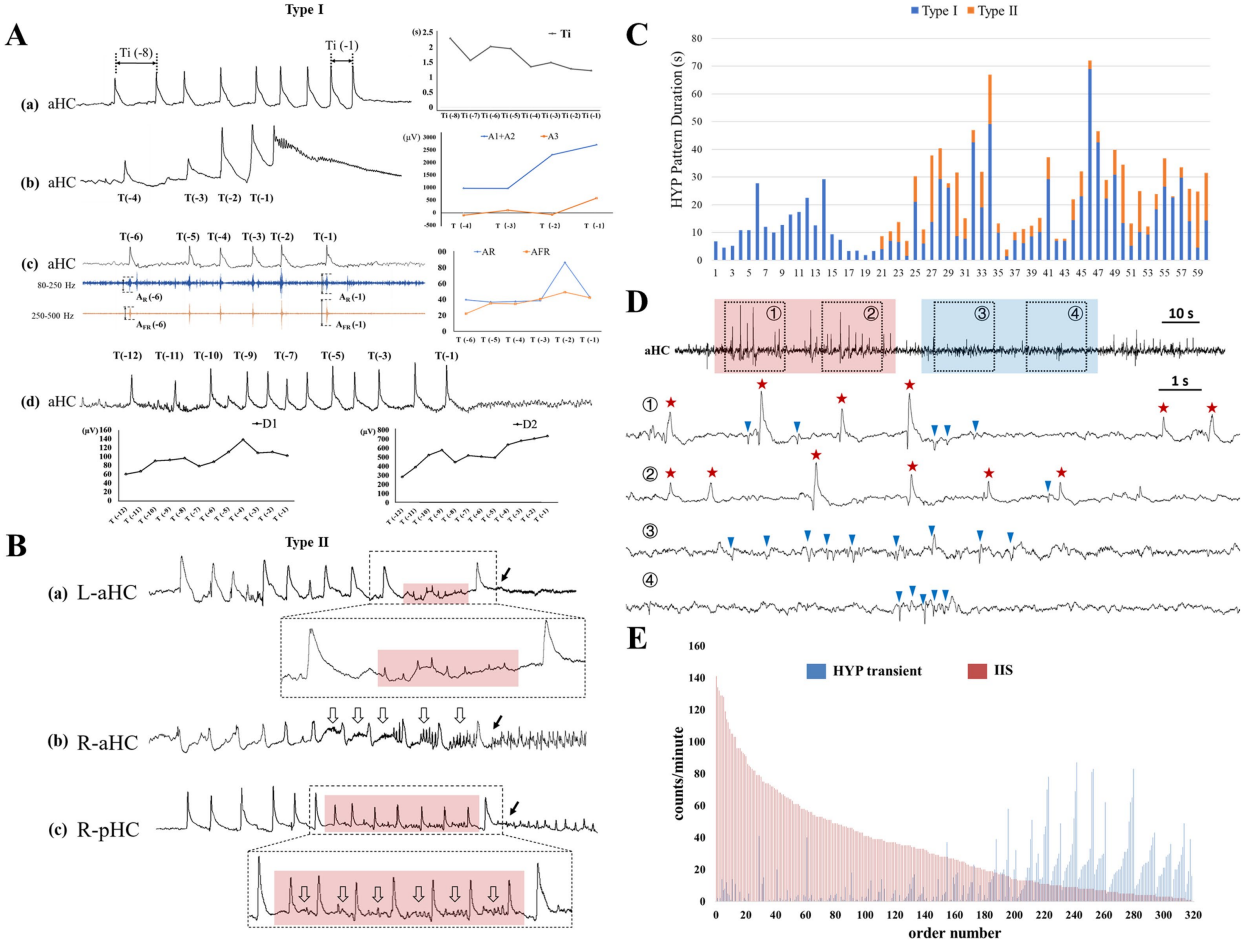
The existence and evolution of HYP pattern during the preictal and interictal stages. **(A)** Shows the example of the smooth modification of HYP pattern (type I) within the preictal epoch, including **(a)** gradual shortening of the inter-transient interval (Ti), progressive amplitude increases of **(b)** SSC, and **(c)** accompanying ripple (AR) and fast ripple (AFR), **(d)** broadening of sharp wave (D1) and slow proper (D2). **(B)** Exhibits additional components inserted between HYP transients and electrical onset (labeled by the solid arrows): **(a)** extreme low amplitude spike (transparent red box); **(b)** low amplitude rhythmic activities (hollow arrows); **(c)** combination of low amplitude spike and rhythmic activities (red transparent box plus hollow arrows). **(C)** Display the time duration of the HYP patterns. Note that there were 40 seizures possessing both of the two types. **(D)** Shows the two pathophysiological “states” during interictal stages. ① and ② labeled by transparent blue box was featured by frequent occurrence of typical IISs and absence of HYP transients. ③ and ④ labeled by transparent red box was characterized by emergence of HYP with avoidance of typical IIS. In the lower extended graph, the HYP transients and IIS were labeled by red stars and blue triangles. **(E)** Indicates the counts of both HYP transient and IIS in each 1-min epoch. Note that the epochs sorted in descending order of IIS number, and the order reveal the mutual “repelling” between the two types of epileptic discharges: the more interictal “HYP transients,” the less IISs, and vice versa.

Type I was featured as gradual (smooth) modification of SSCs within the preictal epoch (Figure 4A). Fifty-nine (98.3%) out of 60 HYP seizures presented this type of transition pattern except that one seizure presented only one typical HYP transient. Using linear analysis, the manners of smooth transition along the preictal-ictal axis were revealed, including gradual shortening of the inter-transient interval (*n* = 42, 70.0%), increase of amplitude and time duration of slow-wave proper (*n* = 47, 78.3%; *n* = 44, 73.3% respectively) and sharp wave (*n* = 37, 61.7%; *n* = 31, 51.7% respectively), and amplitude decrease of the post-slow component (*n* = 51, 85.0%). Amplitude increases of ripple (*n* = 44, 73.3%) and fast ripple (*n* = 47, 78.3%) was also confirmed. The detailed results are indicated in Supplementary material. The mean duration of type I was 15.4 ± 12.8 s.

Along the preictal-ictal axis, 40 out of the 60 HYP seizures exhibited some more complex components inserted between the smooth transitional pattern (type I) and the electrical onset (Figure 4C). All of them were categorized into type II (HYP plus pattern) in the present study. In 8 out of 40 seizures, the prominent feature of these transients was minor or extreme reduction in amplitude with decrease of inter-transient intervals (Figure 4B). In 16 out of 40 seizures, with preservation in morphology compared with typical SSC, the transients were always superimposed or followed by the emergent low amplitude rhythmic activities ranging from theta to gamma bands (Figure 4B). The remaining 16 seizures manifested both the aforementioned patterns (Figure 4B). A noticeable finding was that the emergent rhythmic activities could be considered as the precursor of the subsequent rhythms during the ictal periods. The mean duration of type II was 8.1 ± 6.2 s.

### 3.4 Temporal incompatibility between interictal “HYP transients” and IISs

A total of 320 1-min interictal epochs were included and exhibited 14,891 epileptiform discharges (interictal “HYP” transients, 4,164, 28.0%; IISs, 10,727, 72.0%). Our inspection uncovered that (1) the morphology of interictal “HYP” transients resembled that of SSCs; (2) the interictal “HYP” transients often occurred in cluster or were concentrated in some certain epochs; (3) A significant negative correlation was found between the rate of interictal “HYP transients” and that of IISs (Spearman correlation coefficient = −0.688, *p* < 0.001) in these interictal epochs, indicating that within a certain epoch, the more interictal “HYP transients,” the less IISs, and vice versa (Figures 4D,E).

## 4 Discussion

The main findings of the present study can be summarized as follows: (1) as the major pattern in preictal epochs, HYP trended toward evolving into rhythmic activity (in *β*-*γ* band) rather than low voltage fast activity; (2) HYP transients showed a constant combinational feature along which a slow proper was always superimposed by a sharp wave (termed as slow-sharp complex) and followed by a post-slow component; HFOs superimposed on SSCs in general and were restricted by the sharp waves in majority; (3) in keeping with ictal discharges, the HYP pattern during preictal epoch also exhibited evolutionary change along the time axis; and (4) typical IISs and rudimentary “HYP transients” were mutually “repelling” during interictal periods.

### 4.1 HYP as a specific preictal pattern in the mesial temporal seizure

Although the HYP pattern had been recognized as a specific seizure onset pattern to mesial temporal seizure (4, 14), we are inclined to view it as a transitional pattern from interictal state to seizure, which is supported by the facts revealed by our study.

Firstly, the HYP patterns could evolve into two distinct tonic patterns—RA and LVFA, even in the same epileptogenic lesion. This maybe reflect the fact that the HYP pattern, to some extent, was independent from the consequent ictal tonic pattern. Nonetheless, HYP patterns were more inclined to evolve into tonic RA (71 out of 93 seizures) reflecting the specific structure–function relation in mesial temporal regions. In addition, the anterior hippocampus, which was substantially more active than the amygdala and posterior hippocampus, recorded 65 (63.1%) of the 103 tonic discharges with RA. It is concluded that the evolution of HYP into RA may be related to more focal pathological changes, such as hippocampal sclerosis or focal cortical dysplasia.

Secondly, all the HYP patterns *per se* showed the specific unidirectional and irreversible evolutionary attributes in morphology along preictal-ictal axis. The principal transient deforming manner (type I) showed a build-up feature, probably corresponding to “preictal spiking acceleration” in some *in vitro* studies (7), manifesting amplitude and duration increase and inter-transient interval decrease of SSC. The gradual deformation may reflect some kind of quantitative changes during ictogenesis (15). In the majority of HYP seizures (40 from 60 seizures), furthermore, continuing the build-up pattern (type I), transients presenting abrupt deformation or combining with low amplitude *β*-*γ* activities (type II) were also shown. Interestingly, the emergent rhythmic activities in type II seem to be the precursor of the subsequent rhythms in tonic ictal period. The findings further underscore the progressive evolutionary feature in the mesial temporal seizures.

Thirdly, some HYP transients emerged in cluster during interictal epochs in the mesial temporal structures from which the HYP seizures arose. Importantly, the typical IISs and HYP transients were found to mutually “avoid” each other, indicating that within a certain epoch, the more interictal “HYP transients,” the less the typical interictal spikes, and vice versa (Figures 4D,E). This implies that there may be different underlying neural mechanisms at play. Thus, we hypothesized that these HYP pattern represented the ictogenic trend and their emergence in cluster during interictal period should reflect the failure of “summit” (achieve the threshold of seizure).

According to our clinical observation via SEEG, although not all in the seizure, there was always a conspicuous transitional phase between the foregoing interictal phase and the contiguous ictal phase (Figure 1A). It is interesting that, in contrast to interictal spikes with a high variability in morphology, the morphology of preictal discharges in a certain patient was more conserved across distinct preictal phases. Therefore, we postulated that preictal discharges could be utilized as a detectable indicator for impending electrical seizures in future research and the electrical stimulation applied during “preictal stage” could help for improving the ability to prevent the onset of real seizures and further increasing the direct effects of responsive neuromodulation.

### 4.2 HYP transient reflected the interaction between excitation and inhibition

The HYP pattern well defined in clinical observations has been approximatively reproduced in *in vitro* studies (14, 16–18). The laboratory HYP seizure could be induced by administration of the GABA_A_ antagonist picrotoxin, emphasizing that excitatory signaling could play an important role in the initiation of HYP pattern. On the other hand, the HYP pattern could be abolished by the GABA_A_ receptor antagonist bicuculline leading to the recognition that the HYP transients were GABA-mediated potentials (16–18). These two seemingly contradictory arguments may reflect the sophisticated interaction between interneurons and principal cells during the process of HYP under distinct experimental environments. In contrast to the “fast” and glutamatergic-dependent interictal spikes, HYP transients were mediated by GABAergic signal and corresponded to synchronous long-lasting depolarization (LLD, 300 ~ 1,200 ms in 4AP epilepsy model) of pyramidal neurons (16, 17). From our observations, the duration of HYP transients determined by that of slow-wave proper (median 409.0 ms) were very close to the duration of LLD, which led us to suggest that the slow-wave proper corresponds to the LLD which reflects the depolarizing effect of GABAergic signal. Similarly, the duration of the sharp wave (median 62.5 ms) riding on the slow-wave proper was also very close to the duration of typical IISs (13, 19), maybe reflecting the strong synchrony of glutamatergic intracellular events, termed by paroxysmal depolarizing shift (PDS), in pyramidal neurons (20, 21). Furthermore, the fact that HFOs were more dominantly superimposing on the sharp waves emphasized the contribution of glutamatergic signals to the emergence of HFOs (22).

It has been deeply known that the excitatory effects of GABAergic signal relay on the increase of extracellular potassium concentration ([K^+^]_O_) can be induced by the massive activation of the GABA_A_ receptor and further cause positive shifts of the reversal potential of GABA_A_-mediated postsynaptic events (23, 24). The *vitro* 4AP epileptic modal had shown that significant accumulations of [K^+^]_O_ were associated with the recurrent long-lasting field potentials correlated with LLD of principal cells in the hippocampus (16, 25). We inferred further that during the slow-wave proper, rapidly increasing [K^+^]_O_ may provide an excitatory drive of pyramidal neurons and eventually contributed to synchrony of PDS (mirrored by sharp wave) and action potential (mirrored by HFOs) (20–22). The post-slow component likely reflects both of the depolarization block of pyramidal cells and inhibitory effects of interneurons (26).

### 4.3 The gradual evolution of preictal and ictal discharges reflects the dynamic imbalance of glutamatergic and GABAergic mechanisms

The present study underscored the dynamic evolution of HYP transients in morphology during preictal period in clinical conditions, including increase in amplitude and time duration of slow-wave proper and sharp wave, shortening of the inter-transient interval, and amplitude decrease in the post-slow component. All these matched the findings of *in vitro* and *in vivo* studies of the rodent mesial temporal epileptic model (7). As concluded in experimental studies, the increase of [K^+^]_O_ increased the excitability of interneurons at first and shifted the reversal potential of the GABA_A_ receptor to the positive level (23, 24, 27). The consequent excitatory GABA-mediated postsynaptic currents further recruited more interneurons into the epileptic network step by step (28, 29). Thus, in the clinical condition, the increase of wave width and amplitude of slow-wave proper may mirror the gradual “self-enhancement” of the GABAergic excitatory effect during ictogenesis. The gradual enhancement of GABAergic excitatory effect triggered massive pyramidal, including adjacent and distant pyramidal populations, depolarization of which may be a causative factor for the concomitant shape change of sharp wave and the superimposing HFOs. The amplitude decrement of the post-slow components also represented weakening of GABAergic inhibitory effects.

As mentioned above, the concept that extracellular potassium changes promote neural network excitability alterations associated with seizures has been verified in detail in experimental and computational studies (18, 24, 30). Computational model studies have shown that the size and rise time of [K^+^]_O_ increase lead to the evolution to a pathological condition (30). *In vitro* evidence supports the suggestion that [K^+^]_O_ increasing both in a short time (hundreds of milliseconds) and maintained over a prolonged period (more than several seconds) promote seizure generation (24). In our observation, HYP mostly evolved into RA (71 from 93 HYP seizures) and into LVFA in some cases (22 from 93 seizures). The facts led us to reason that in a gradual manner, [K^+^]_O_ was progressively summated to reach the critical threshold value under which adequate pyramidal cells activated and ictal tonic discharges in *β*-*γ* bands began. While the increment of [K^+^]_O_ in a gradual manner failed to trigger a seizure, [K^+^]_O_ accumulation in a bursting manner was enabled, represented by LVFA with SPS (GABA-dominant mechanism) and achieved the specific threshold value for pyramidal cell massive activation. In brief, from the viewpoint of extracellular potassium accumulation, the effects of HYP pattern in the “acceleration” manner were equivalent to LVFA with SPS.

## 5 Conclusion

We observed in detail the morphological features and temporal evolutional manners of HYP pattern in human MTLE and tried to translate experimental findings to the clinical condition. Although it is difficult to confirm the neurobiological mechanism *in vivo* under clinical conditions, consistency of findings between animal experiments and the clinical practice suggests that ictogenesis of epileptic brain slice and intact brain share common neural mechanisms. Briefly, HYP, consisting of phasic combinational epileptic components, is a specific type of cross-frequency coupling phenomenon in epileptogenic mesial temporal structures and firmly demonstrates the transitional trend from the interictal to ictal phase (8, 31).

## Data Availability

The original contributions presented in the study are included in the article/Supplementary material, further inquiries can be directed to the corresponding authors.

## References

[1] Fisher RS Scharfman HE deCurtis M. How can we identify ictal and interictal abnormal activity? Adv Exp Med Biol. (2014) 813:3–23. doi: 10.1007/978-94-017-8914-1_1, PMID: 25012363 PMC4375749

[2] Mormann F Andrzejak RG Elger CE Lehnertz K. Seizure prediction: the long and winding road. Brain. (2007) 130:314–33. doi: 10.1093/brain/awl241, PMID: 17008335

[3] Chvojka J Kudlacek J Chang WC Novak O Tomaska F Otahal J. The role of interictal discharges in ictogenesis - a dynamical perspective. Epilepsy Behav. (2021) 121:106591. doi: 10.1016/j.yebeh.2019.106591, PMID: 31806490

[4] Velasco AL Wilson CL Babb TL Engel J Jr. Functional and anatomic correlates of two frequently observed temporal lobe seizure-onset patterns. Neural Plast. (2000) 7:49–63. doi: 10.1155/NP.2000.49, PMID: 10709214 PMC2565365

[5] Bragin A Azizyan A Almajano J Wilson CL Engel J Jr. Analysis of chronic seizure onsets after intrahippocampal kainic acid injection in freely moving rats. Epilepsia. (2005) 46:1592–8. doi: 10.1111/j.1528-1167.2005.00268.x, PMID: 16190929

[6] Gnatkovsky V Librizzi L Trombin F de Curtis M. Fast activity at seizure onset is mediated by inhibitory circuits in the entorhinal cortex in vitro. Ann Neurol. (2008) 64:674–86. doi: 10.1002/ana.21519, PMID: 19107991

[7] Köhling R D'Antuono M Benini R de Guzman P Avoli M. Hypersynchronous ictal onset in the perirhinal cortex results from dynamic weakening in inhibition. Neurobiol Dis. (2016) 87:1–10. doi: 10.1016/j.nbd.2015.12.002, PMID: 26699817 PMC4878890

[8] Huberfeld G Menendez de la Prida L Pallud J Cohen I Le Van QM Adam C. Glutamatergic pre-ictal discharges emerge at the transition to seizure in human epilepsy. Nat Neurosci. (2011) 14:627–34. doi: 10.1038/nn.2790, PMID: 21460834

[9] Insausti R Tuñón T Sobreviela T Insausti AM Gonzalo LM. The human entorhinal cortex: a cytoarchitectonic analysis. J Comp Neurol. (1995) 355:171–98. doi: 10.1002/cne.903550203, PMID: 7541808

[10] Perucca P Dubeau F Gotman J. Intracranial electroencephalographic seizure-onset patterns: effect of underlying pathology. Brain. (2014) 137:183–96. doi: 10.1093/brain/awt299, PMID: 24176980

[11] Grinenko O Li J Mosher JC Wang IZ Bulacio JC Gonzalez-Martinez J. A fingerprint of the epileptogenic zone in human epilepsies. Brain. (2018) 141:117–31. doi: 10.1093/brain/awx306, PMID: 29253102 PMC5837527

[12] Jacobs J Staba R Asano E. High-frequency oscillations (HFOs) in clinical epilepsy. Prog Neurobiol. (2012) 98:302–15. doi: 10.1016/j.pneurobio.2012.03.001, PMID: 22480752 PMC3674884

[13] de Curtis M Avanzini G. Interictal spikes in focal epileptogenesis. Prog Neurobiol. (2001) 63:541–67. doi: 10.1016/S0301-0082(00)00026-5, PMID: 11164621

[14] Lévesque M Salami P Gotman J Avoli M. Two seizure-onset types reveal specific patterns of high-frequency oscillations in a model of temporal lobe epilepsy. J Neurosci. (2012) 32:13264–72. doi: 10.1523/JNEUROSCI.5086-11.2012, PMID: 22993442 PMC4878898

[15] Blauwblomme T Jiruska P Huberfeld G. Mechanisms of ictogenesis. Int Rev Neurobiol. (2014) 114:155–85. doi: 10.1016/B978-0-12-418693-4.00007-8, PMID: 25078502

[16] Perreault P Avoli M. 4-aminopyridine-induced epileptiform activity and a GABA-mediated long-lasting depolarization in the rat hippocampus. J Neurosci. (1992) 12:104–15. doi: 10.1523/JNEUROSCI.12-01-00104.1992, PMID: 1309571 PMC6575697

[17] Avoli M Barbarosie M Lücke A Nagao T Lopantsev V Köhling R. Synchronous GABA-mediated potentials and epileptiform discharges in the rat limbic system in vitro. J Neurosci. (1996) 16:3912–24. doi: 10.1523/JNEUROSCI.16-12-03912.1996, PMID: 8656285 PMC6578615

[18] Avoli M. GABA-mediated synchronous potentials and seizure generation. Epilepsia. (1996) 37:1035–42. doi: 10.1111/j.1528-1157.1996.tb01022.x, PMID: 8917052

[19] de Curtis M Radici C Forti M. Cellular mechanisms underlying spontaneous interictal spikes in an acute model of focal cortical epileptogenesis. Neuroscience. (1999) 88:107–17. doi: 10.1016/S0306-4522(98)00201-2, PMID: 10051193

[20] Kubista H Boehm S Hotka M. The paroxysmal depolarization shift: reconsidering its role in epilepsy, Epileptogenesis and beyond. Int J Mol Sci. (2019) 20:577. doi: 10.3390/ijms20030577, PMID: 30699993 PMC6387313

[21] Hotka M Kubista H. The paroxysmal depolarization shift in epilepsy research. Int J Biochem Cell Biol. (2019) 107:77–81. doi: 10.1016/j.biocel.2018.12.006, PMID: 30557621 PMC7116775

[22] Jefferys JG Menendez de la Prida L Wendling F Bragin A Avoli M Timofeev I. Mechanisms of physiological and epileptic HFO generation. Prog Neurobiol. (2012) 98:250–64. doi: 10.1016/j.pneurobio.2012.02.005, PMID: 22420980 PMC4873284

[23] Avoli M de Curtis M. GABAergic synchronization in the limbic system and its role in the generation of epileptiform activity. Prog Neurobiol. (2011) 95:104–32. doi: 10.1016/j.pneurobio.2011.07.003, PMID: 21802488 PMC4878907

[24] de Curtis M Uva L Gnatkovsky V Librizzi L. Potassium dynamics and seizures: why is potassium ictogenic? Epilepsy Res. (2018) 143:50–9. doi: 10.1016/j.eplepsyres.2018.04.005, PMID: 29660559

[25] Avoli M de Curtis M Köhling R. Does interictal synchronization influence ictogenesis? Neuropharmacology. (2013) 69:37–44. doi: 10.1016/j.neuropharm.2012.06.044, PMID: 22776544 PMC4878915

[26] Bikson M Hahn PJ Fox JE Jefferys JG. Depolarization block of neurons during maintenance of electrographic seizures. J Neurophysiol. (2003) 90:2402–8. doi: 10.1152/jn.00467.2003, PMID: 12801897

[27] Rutecki PA Lebeda FJ Johnston D. Epileptiform activity induced by changes in extracellular potassium in hippocampus. J Neurophysiol. (1985) 54:1363–74. doi: 10.1152/jn.1985.54.5.1363 2416891

[28] Ben-Ari Y. Excitatory actions of gaba during development: the nature of the nurture. Nat Rev Neurosci. (2002) 3:728–39. doi: 10.1038/nrn920, PMID: 12209121

[29] Pavlov I Kaila K Kullmann DM Miles R. Cortical inhibition, pH and cell excitability in epilepsy: what are optimal targets for antiepileptic interventions? J Physiol. (2013) 591:765–74. doi: 10.1113/jphysiol.2012.237958, PMID: 22890709 PMC3591695

[30] Gentiletti D Suffczynski P Gnatkovsky V de Curtis M. Changes of ionic concentrations during seizure transitions - a modeling study. Int J Neural Syst. (2017) 27:1750004. doi: 10.1142/S0129065717500046, PMID: 27802792

[31] Buzsáki G Watson BO. Brain rhythms and neural syntax: implications for efficient coding of cognitive content and neuropsychiatric disease. Dialogues Clin Neurosci. (2012) 14:345–67. doi: 10.31887/DCNS.2012.14.4/gbuzsaki, PMID: 23393413 PMC3553572

