# Supplementary material for: An ictogenic marker in the mesial temporal epilepsy and its temporal evolutionary features
Source: Front Neurol. 2025 Jan 10;15:1510108. doi: 10.3389/fneur.2024.1510108 (PMC11757140; doi:10.3389/fneur.2024.1510108)

The detailed statistical results of the 59 seizures presenting HYP type I.

Patient 1 SZ1

Time interval

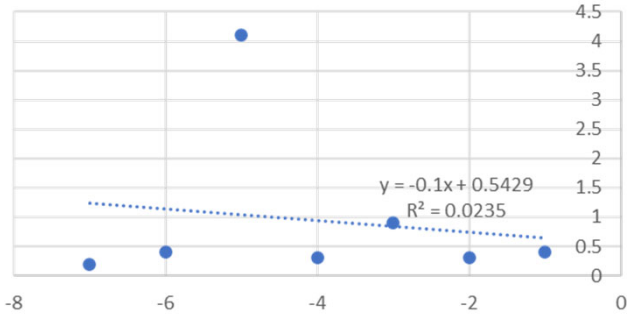

Amplitude of sharp wave

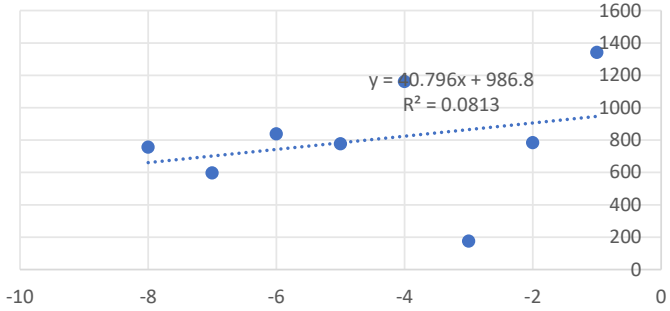

Duration of sharp wave

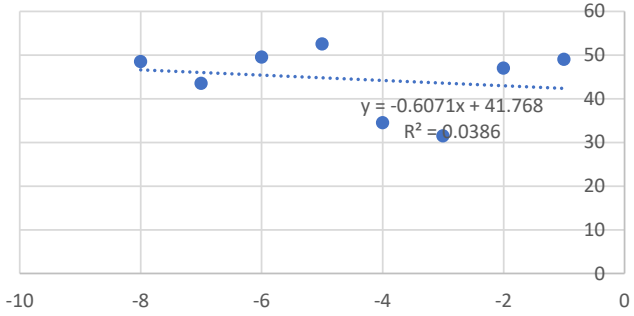

Amplitude of slow proper

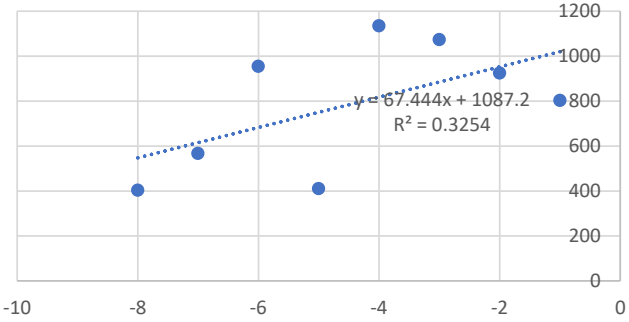

Duration of slow proper

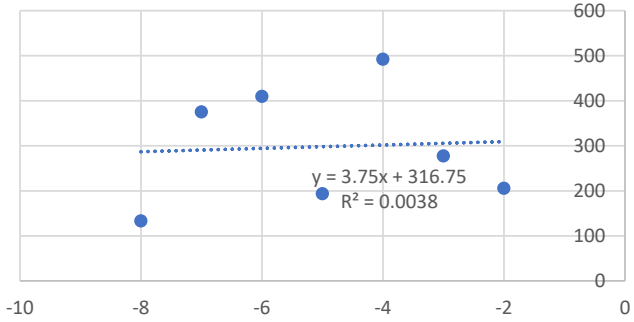

Amplitude of post-slow component

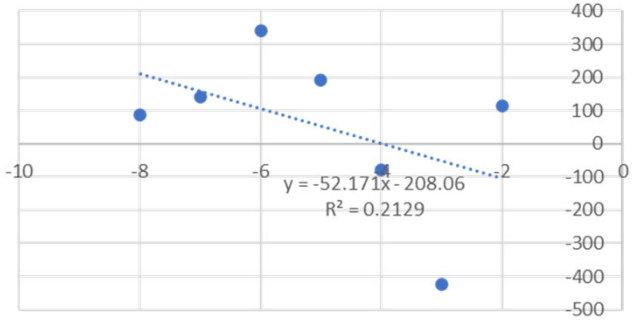

Patient 1 SZ1

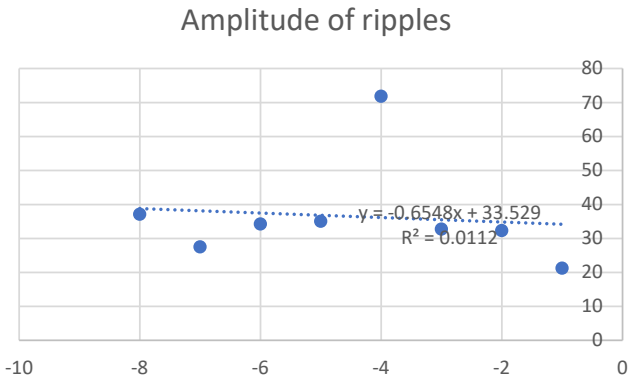

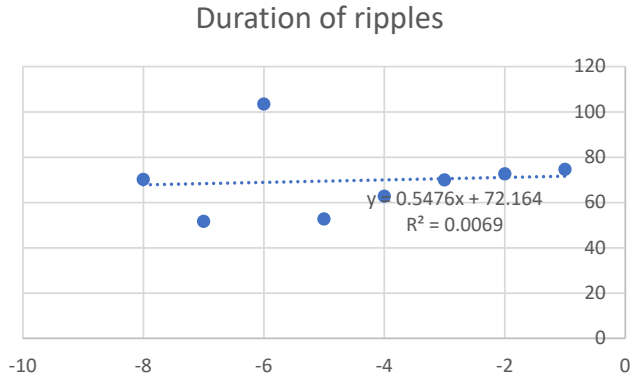

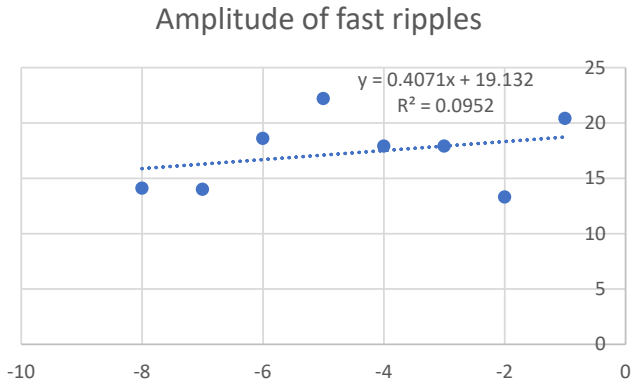

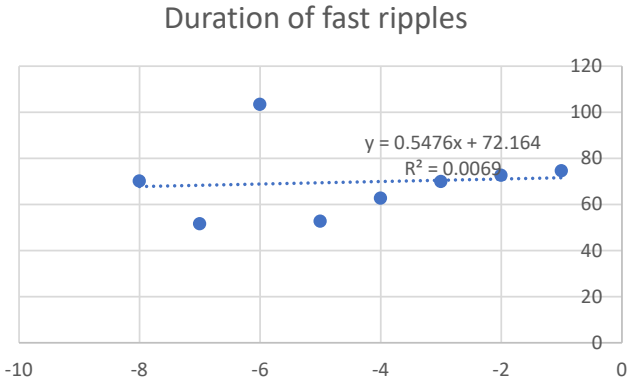

Patient 1 SZ2

Time interval

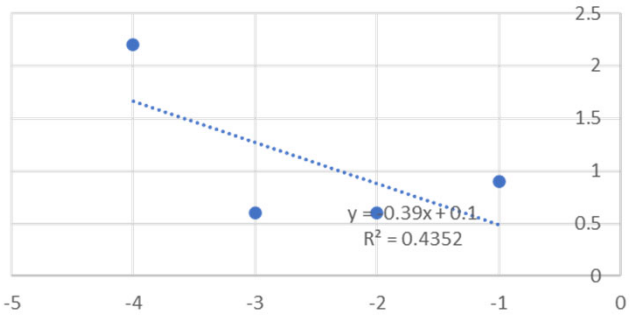

Amplitude of sharp wave

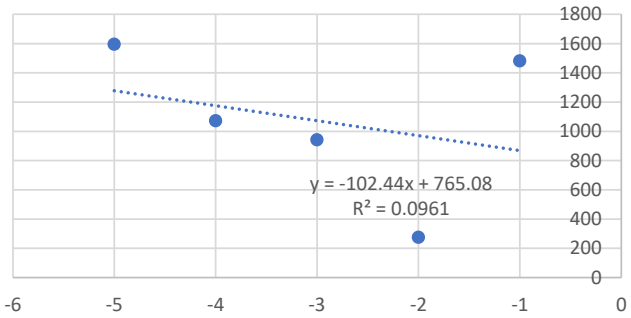

Duration of sharp wave

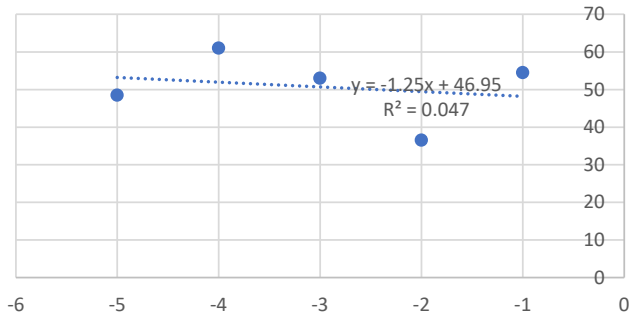

Amplitude of slow proper

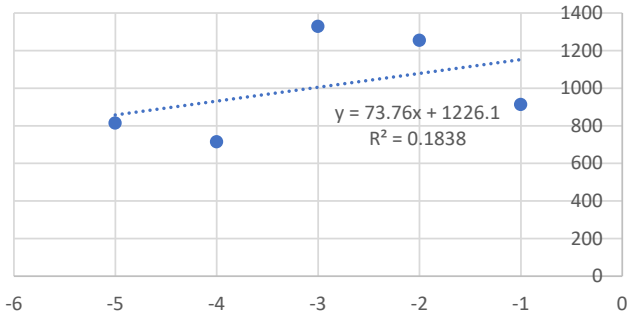

Duration of slow proper

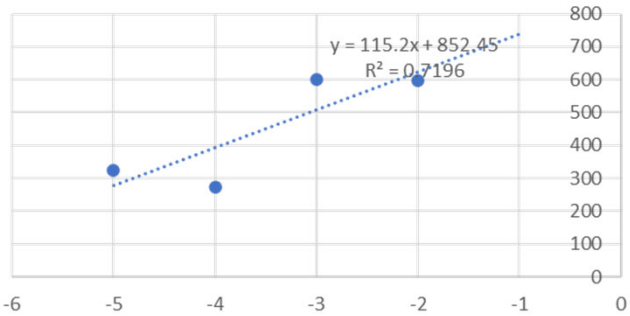

Amplitude of post-slow component

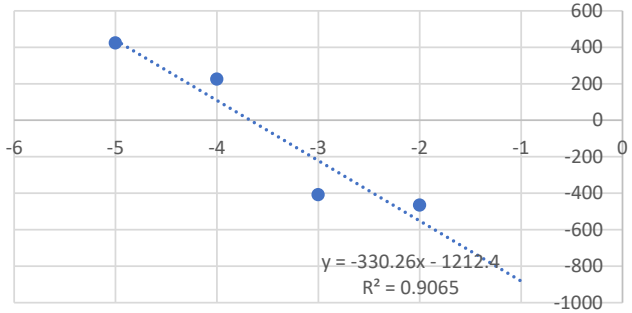

Patient 1 SZ2

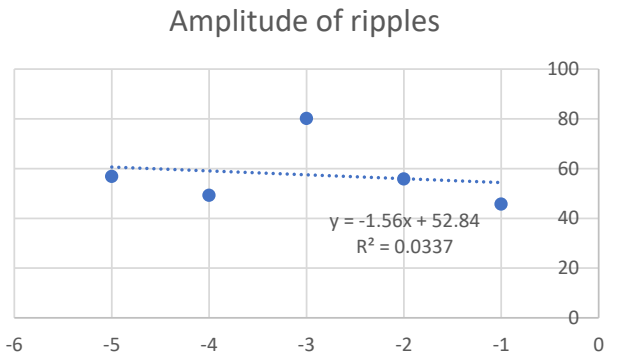

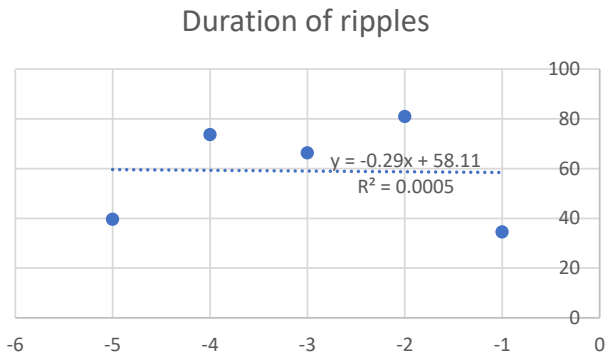

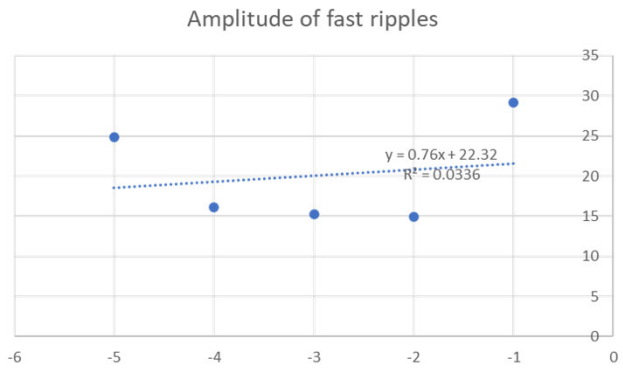

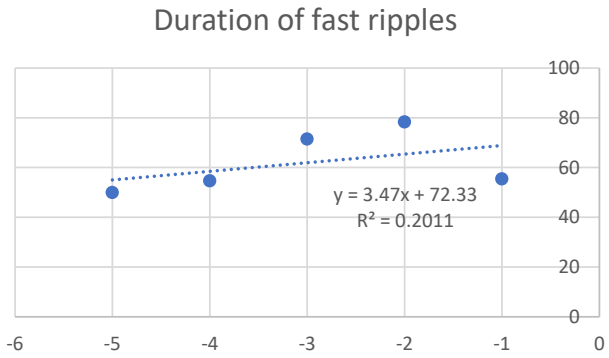

Patient 2 SZ1

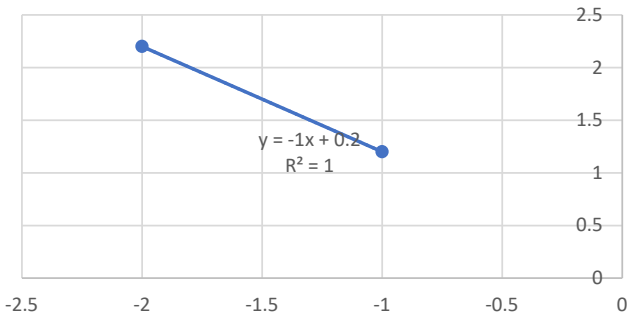

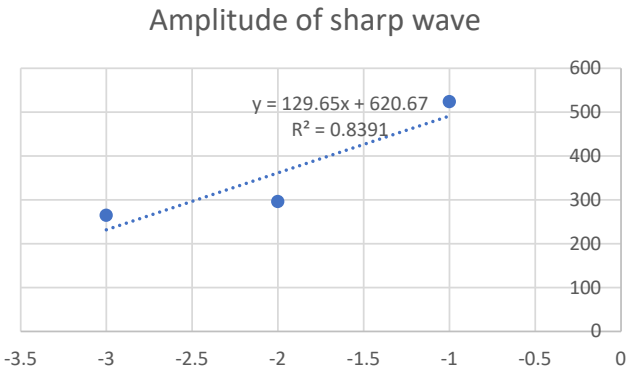

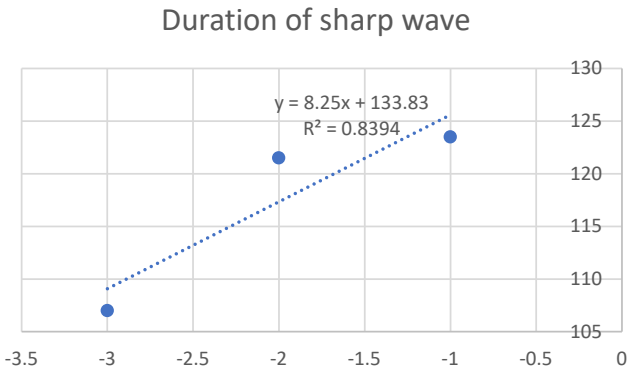

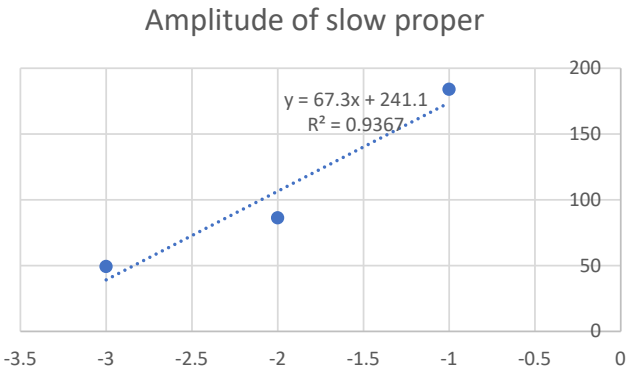

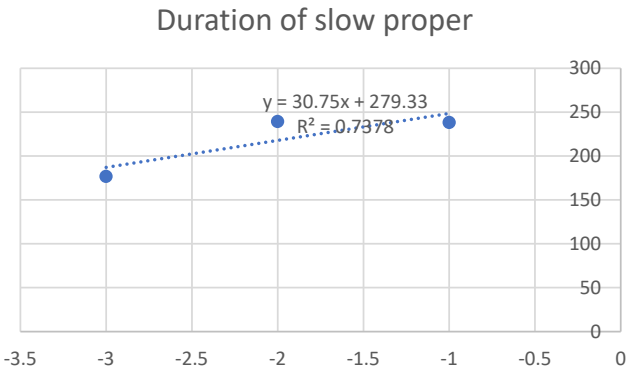

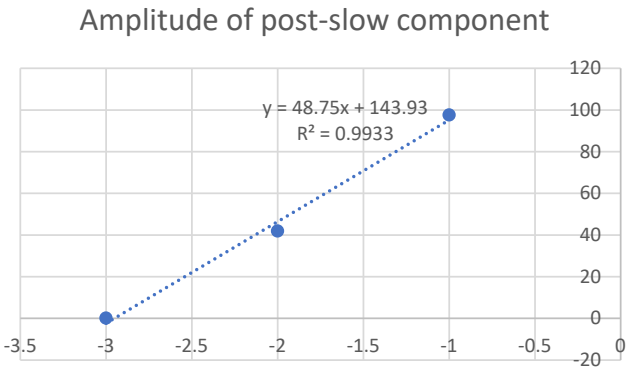

Patient 2 SZ1

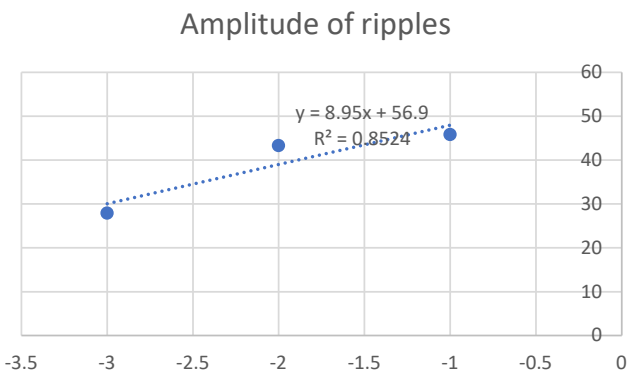

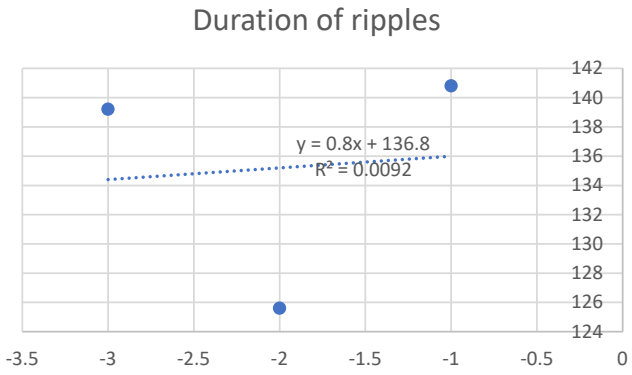

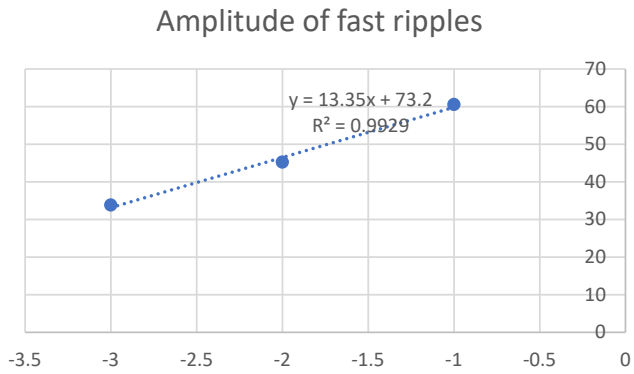

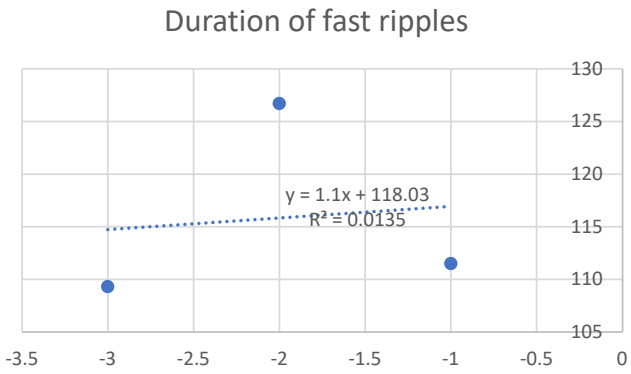

Patient 2 SZ2

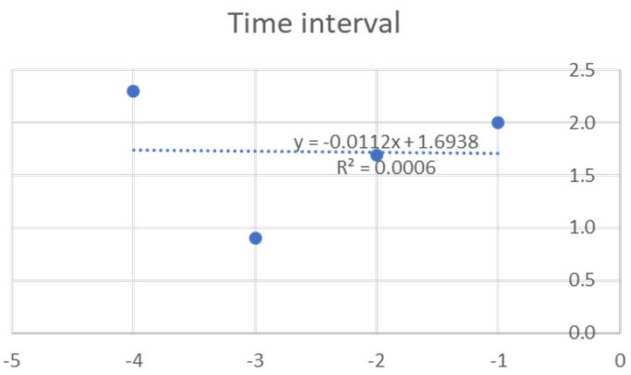

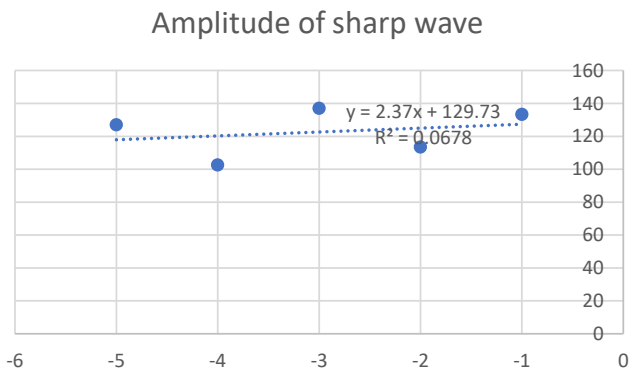

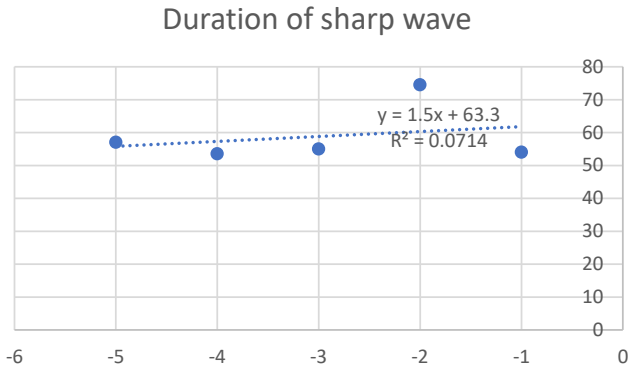

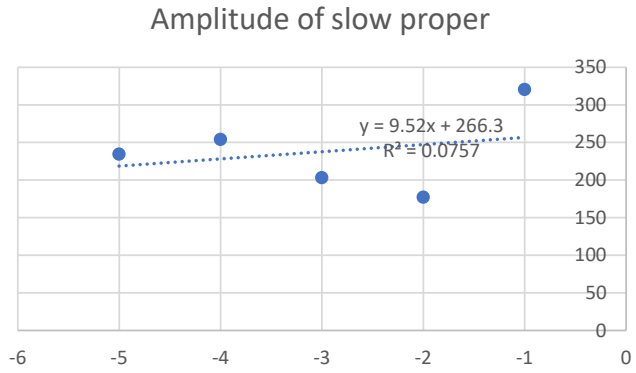

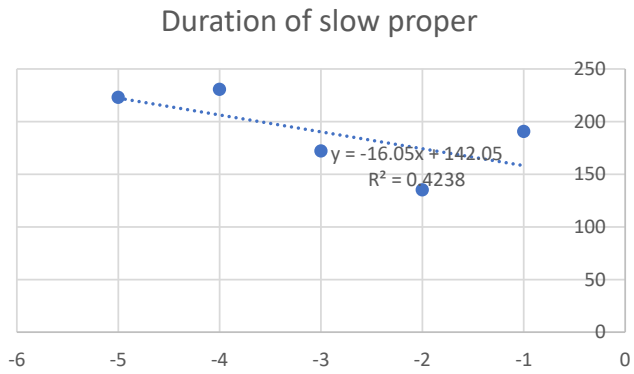

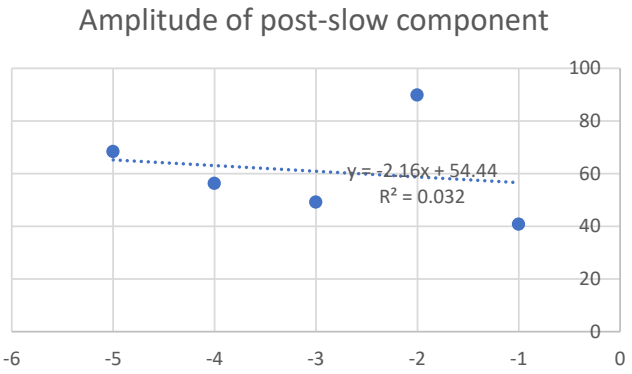

Patient 2 SZ2

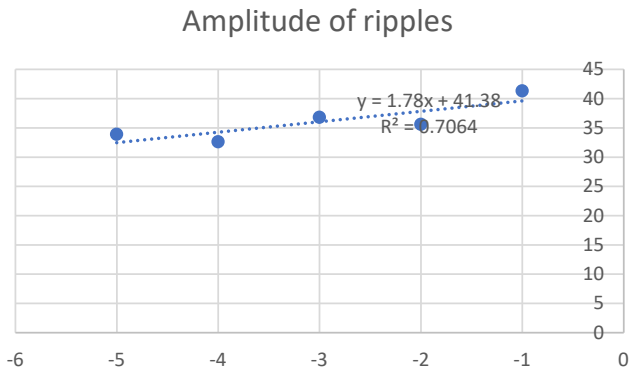

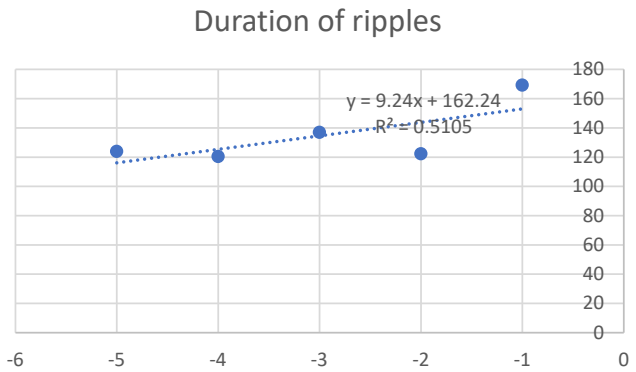

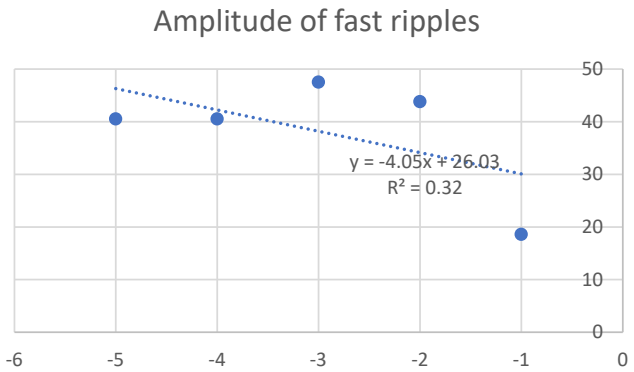

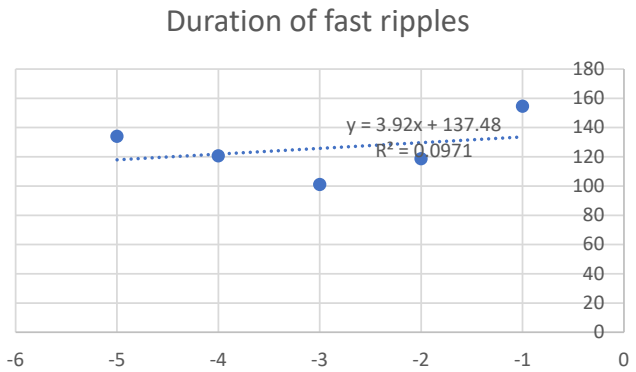

Patient 2 SZ3

Time interval

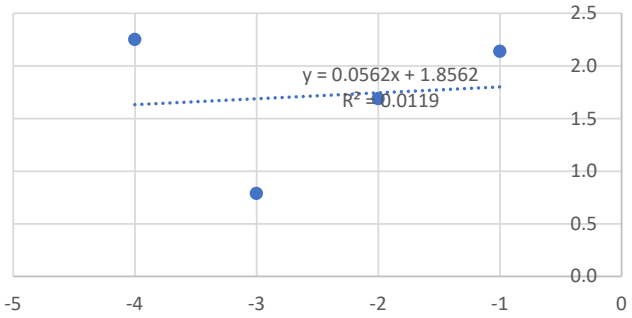

Amplitude of sharp wave

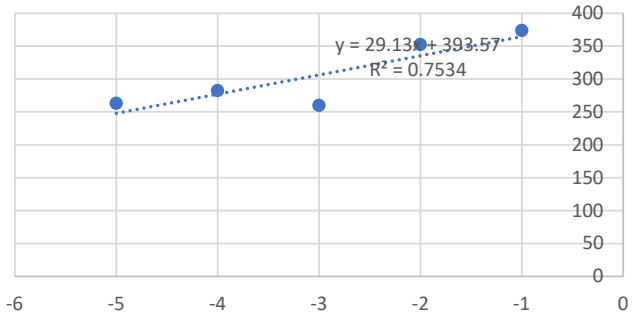

Duration of sharp wave

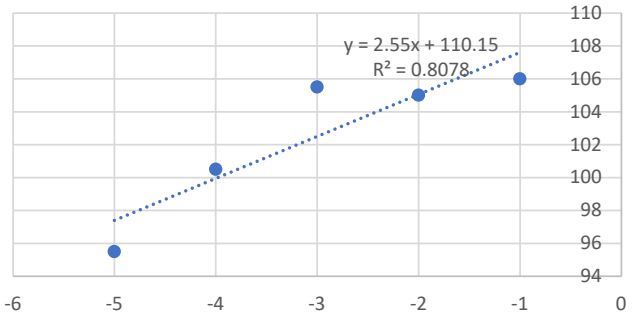

Amplitude of slow proper

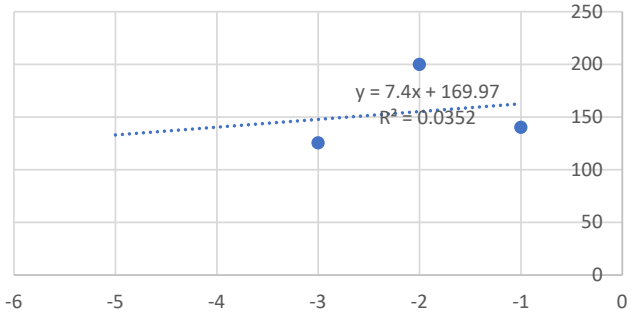

Duration of slow proper

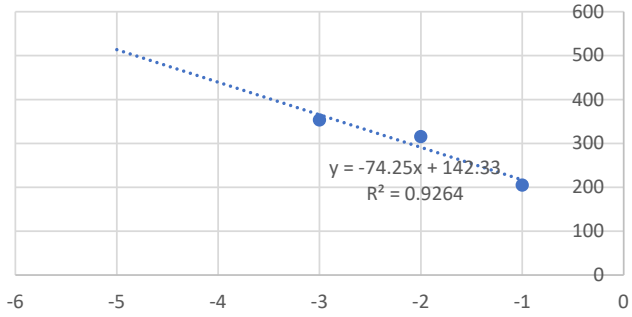

Amplitude of post-slow component

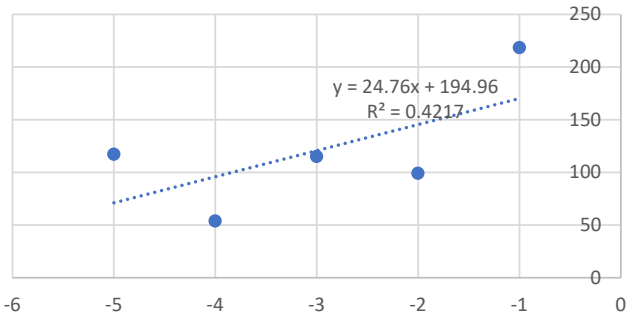

Patient 2 SZ3

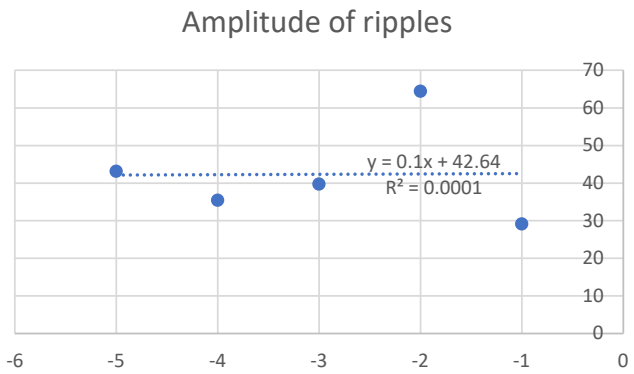

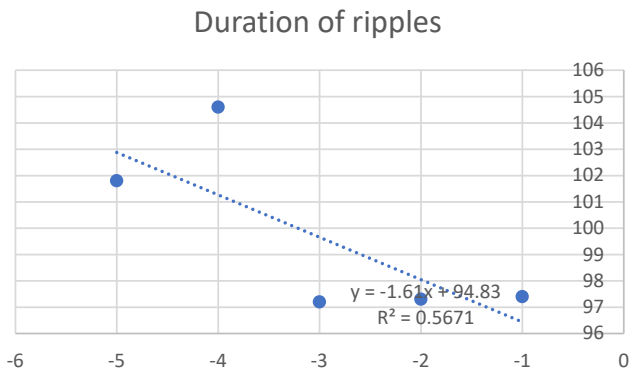

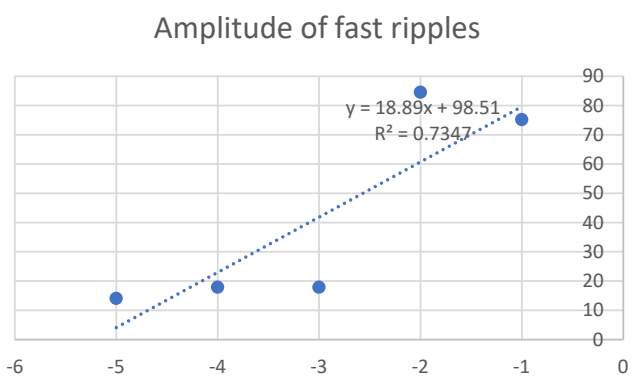

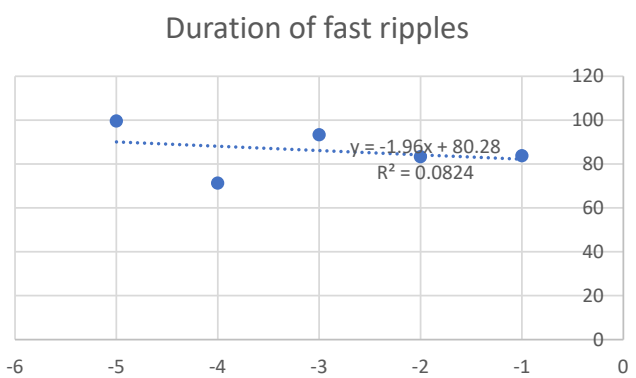

Patient 2 SZ4

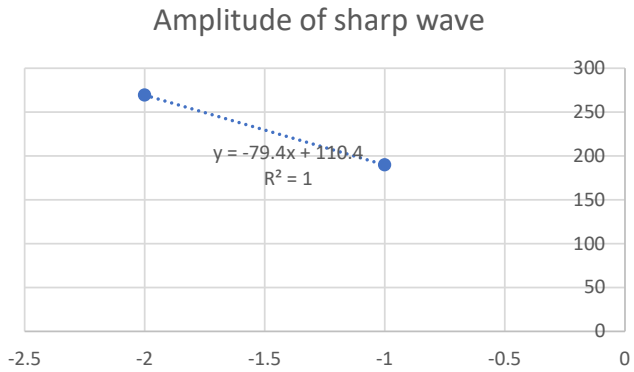

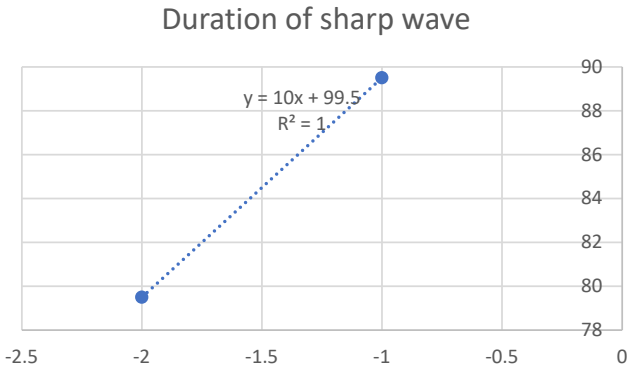

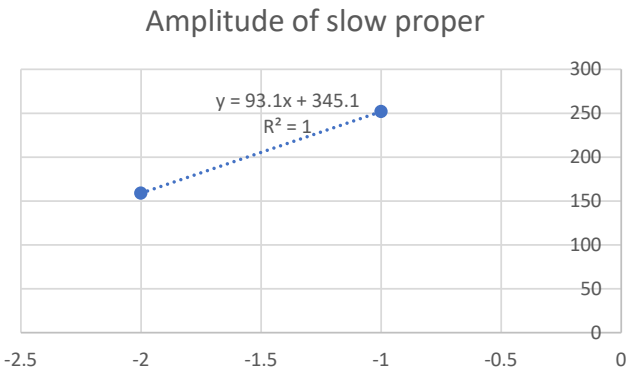

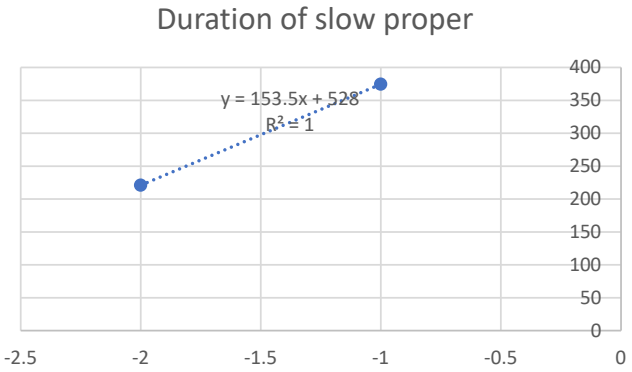

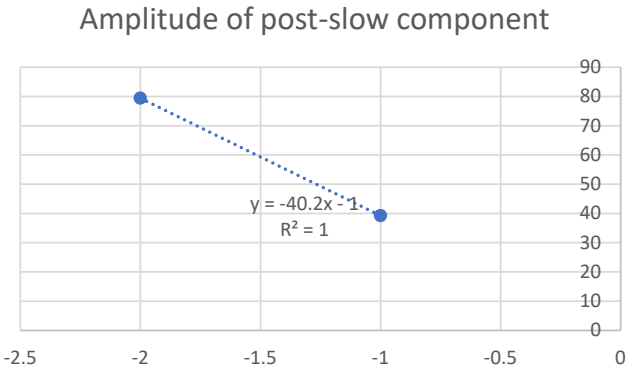

Patient 2 SZ4

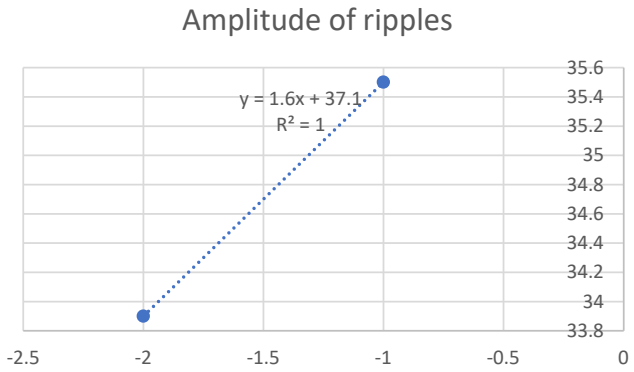

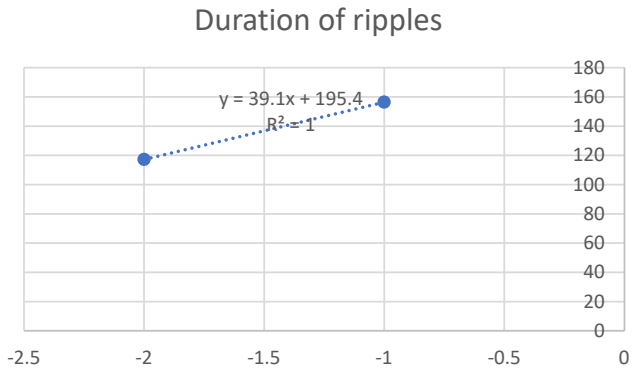

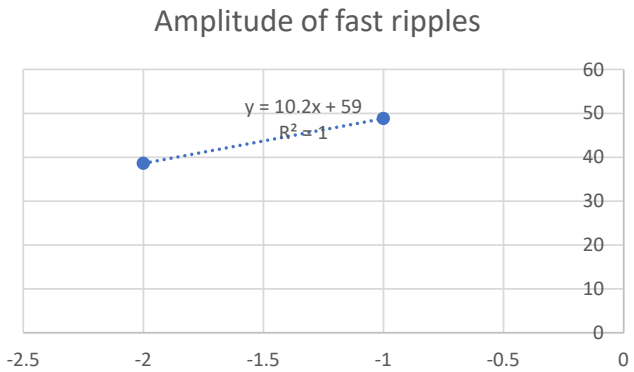

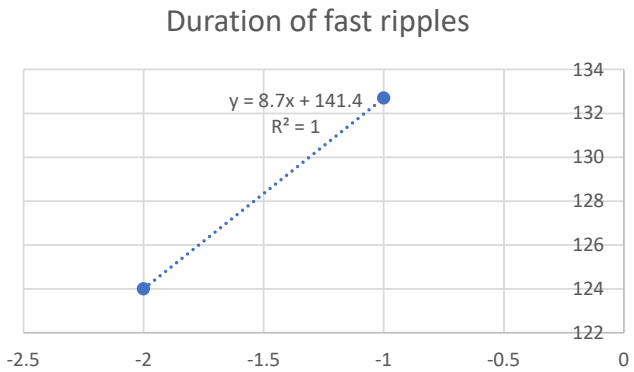

Patient 3 SZ1

Time interval

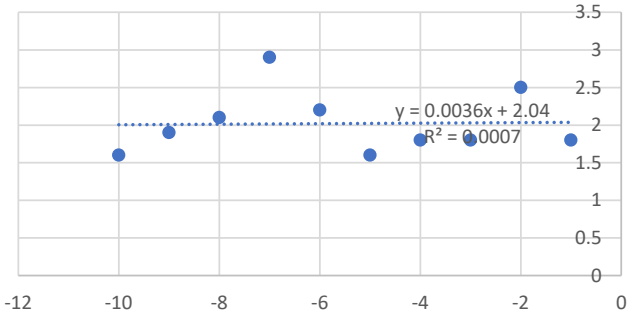

Amplitude of sharp wave

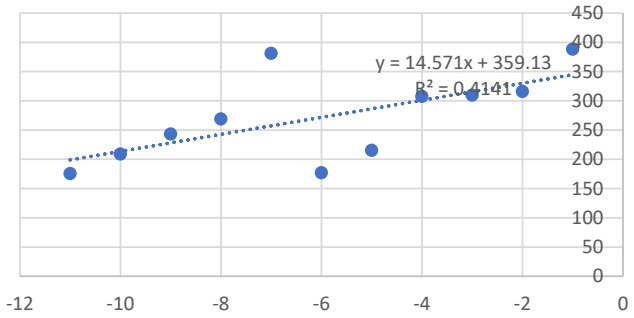

Duration of sharp wave

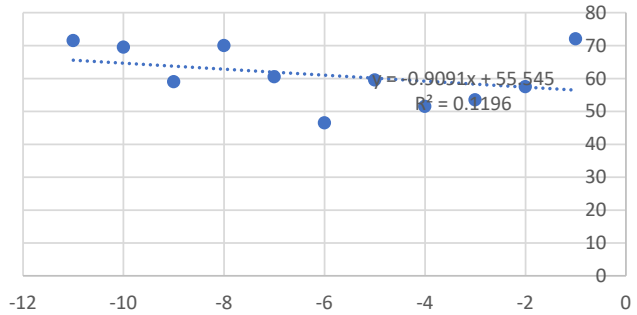

Amplitude of slow proper

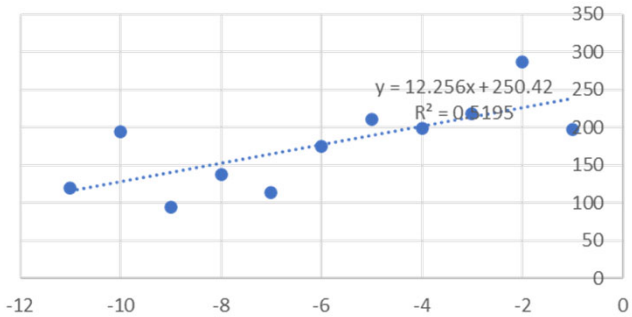

Duration of slow proper

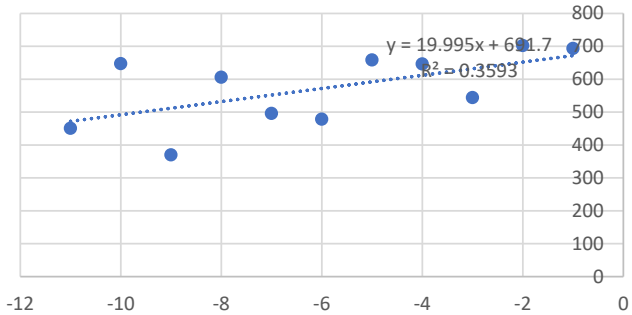

Amplitude of post slow component

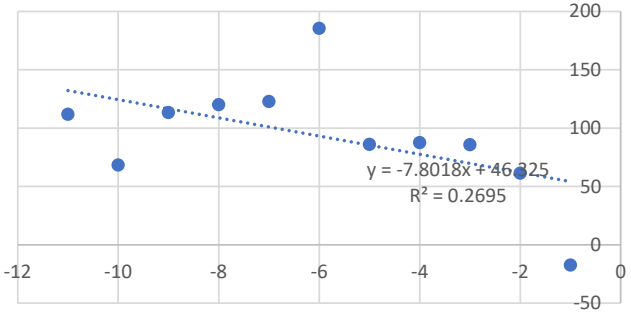

Patient 3 SZ1

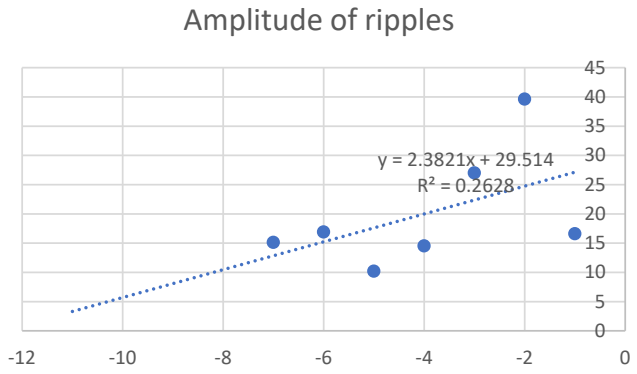

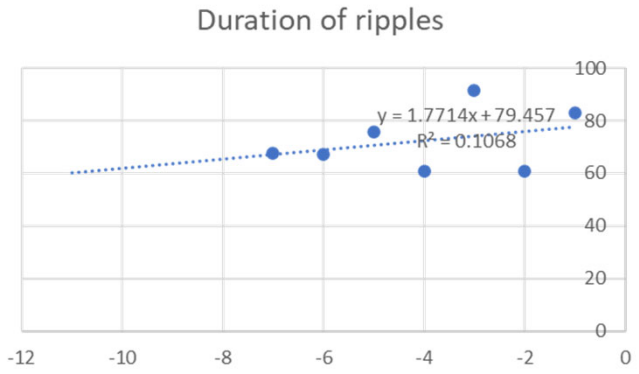

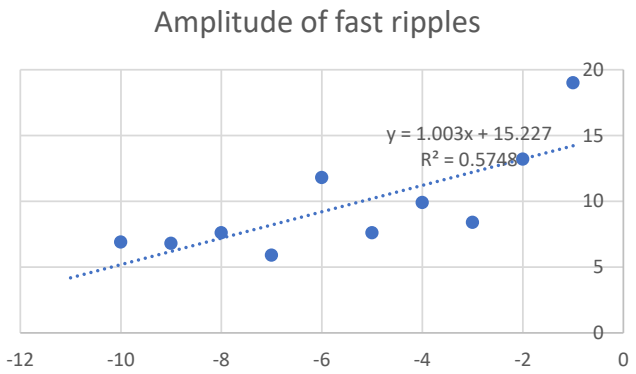

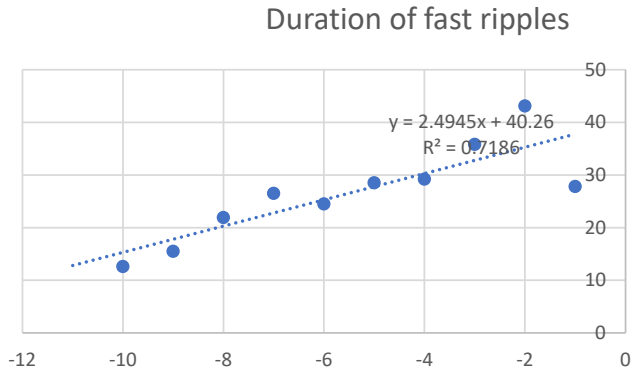

Patient 3 SZ2

Time interval

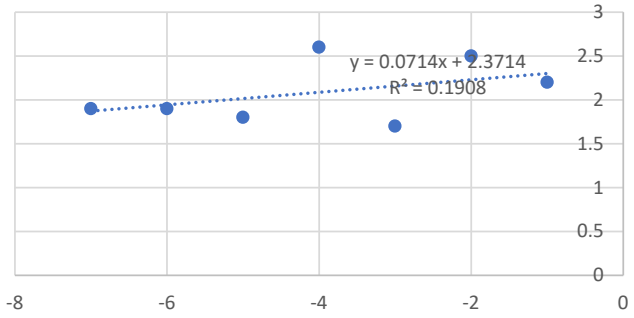

Amplitude of sharp wave

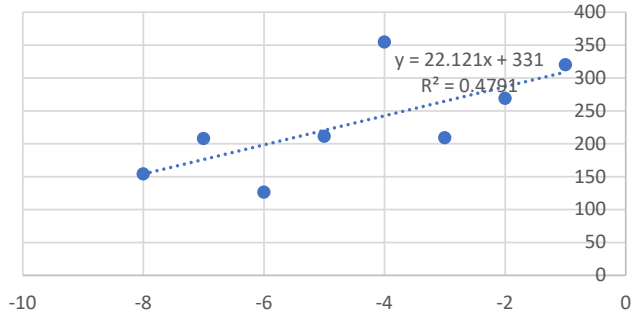

Duration of sharp wave

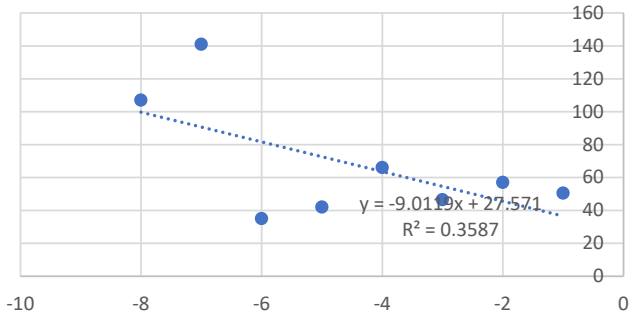

Amplitude of slow proper

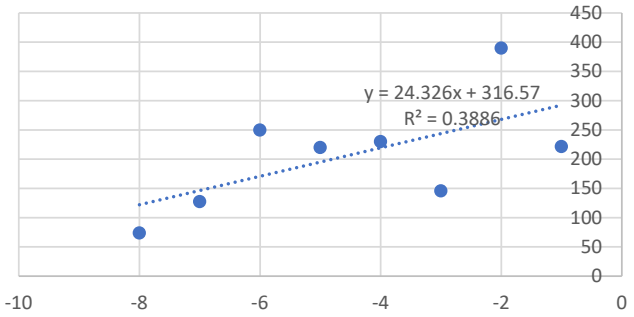

Duration of slow proper

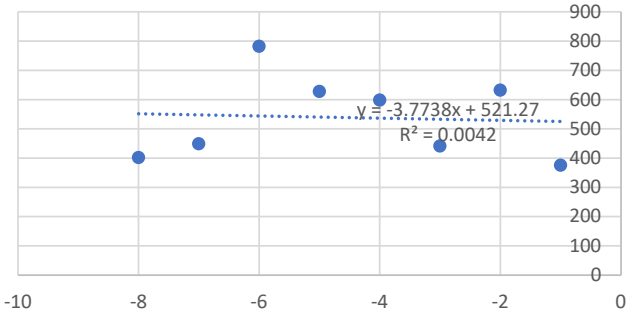

Amplitude of post-slow component

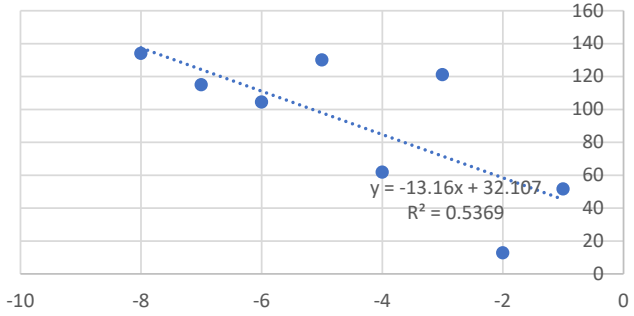

Patient 3 SZ2

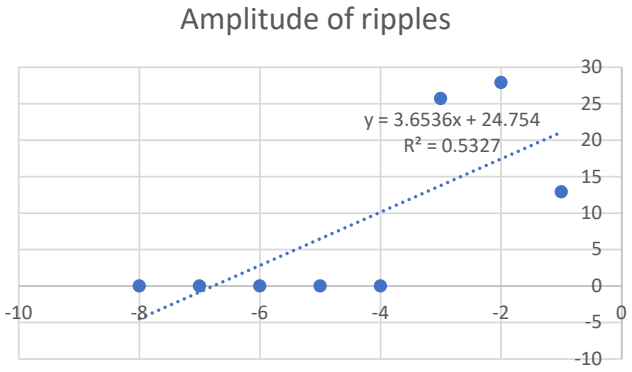

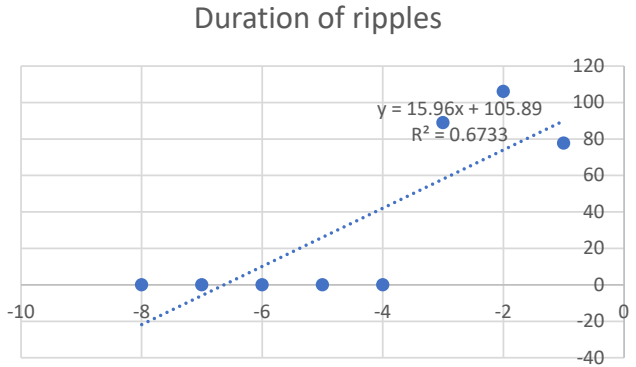

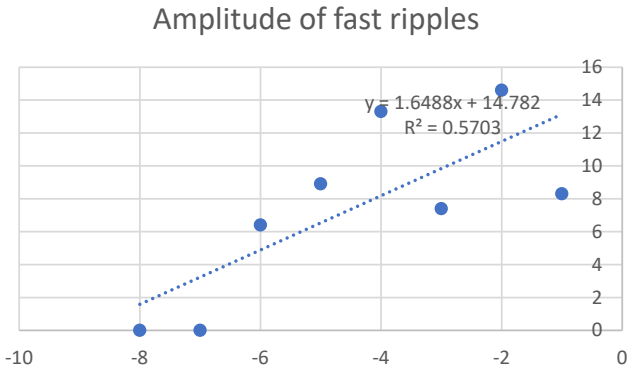

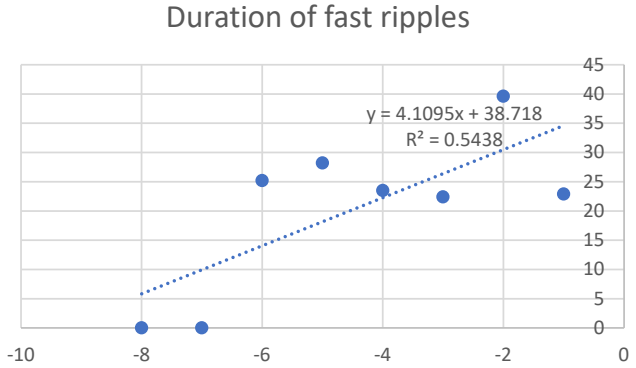

Patient 3 SZ3

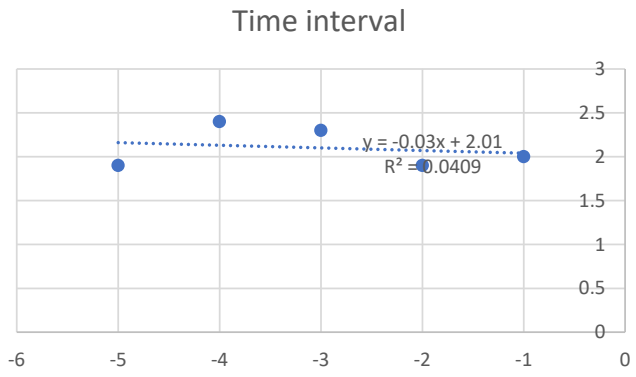

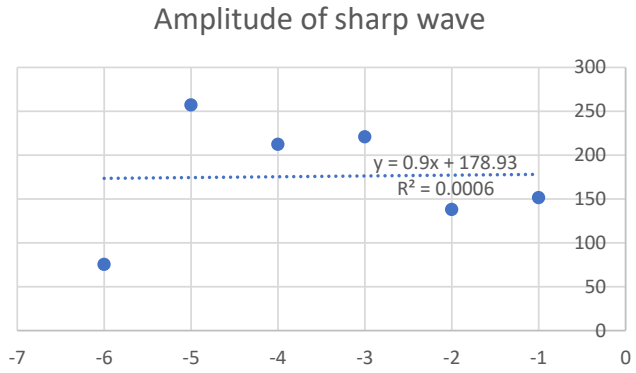

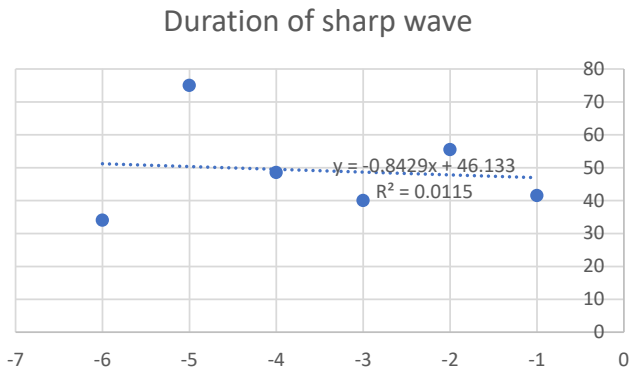

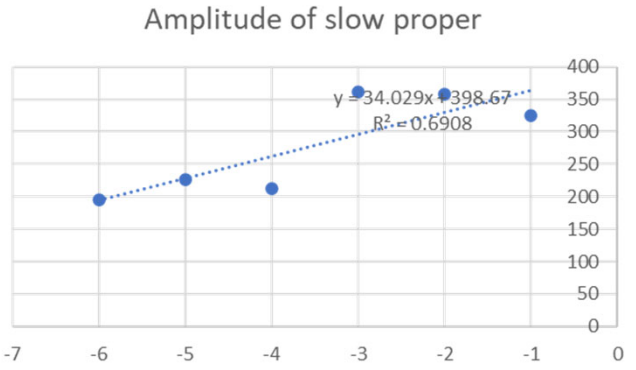

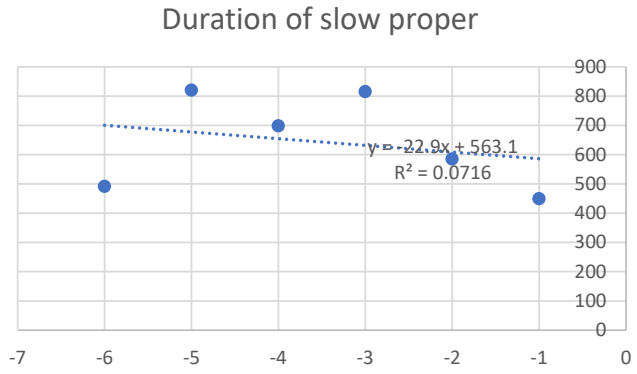

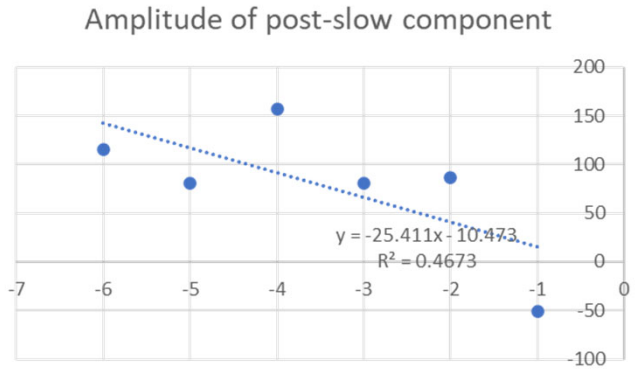

Patient 3 SZ3

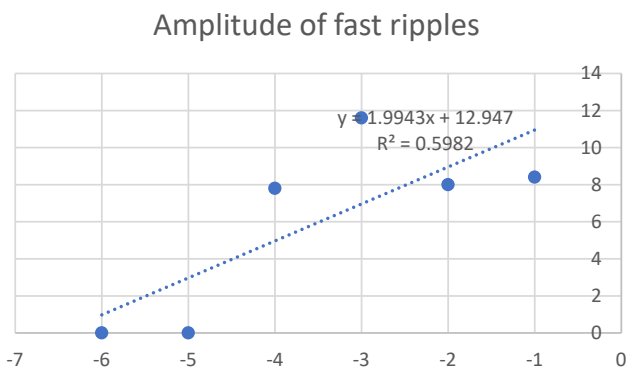

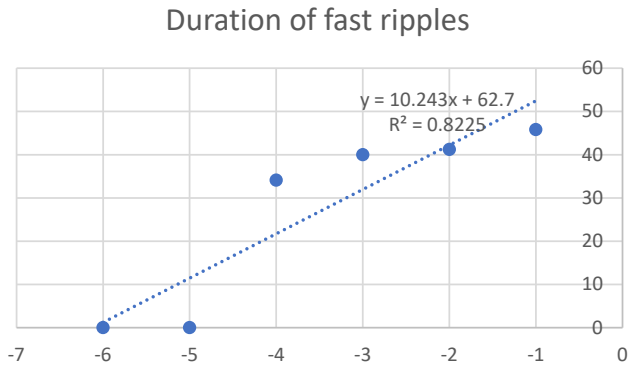

Patient 3 SZ4

Time interval

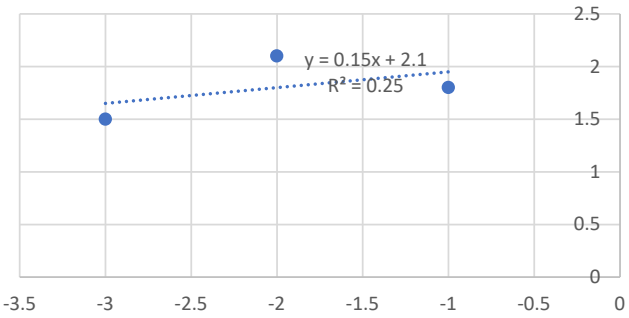

Amplitude of sharp wave

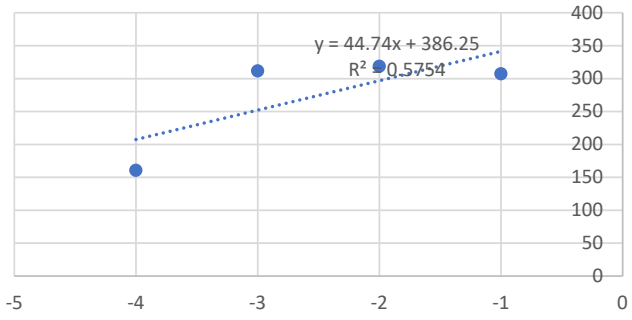

Duration of sharp wave

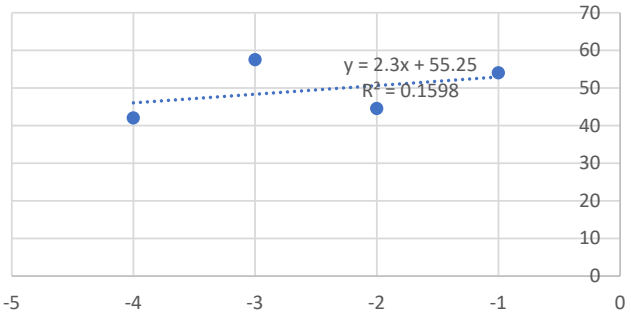

Amplitude of slow proper

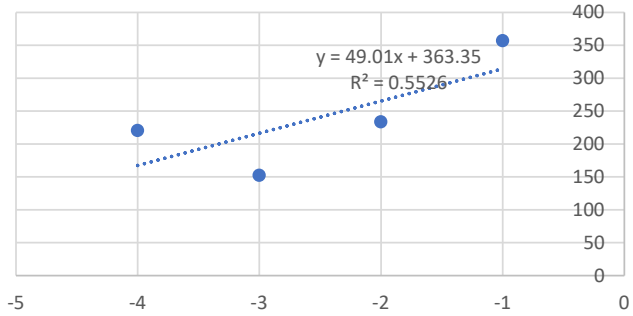

Duration of slow proper

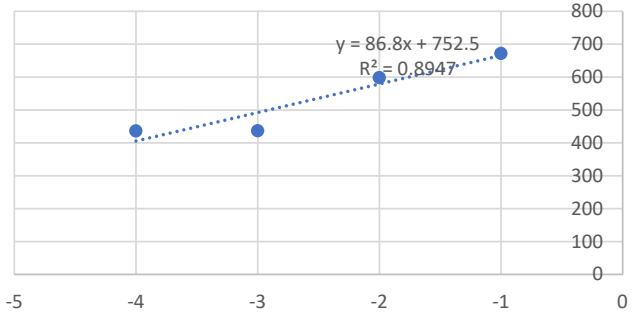

Amplitude of post-slow component

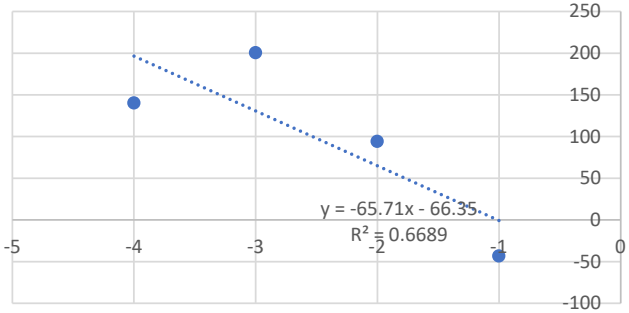

Patient 3 SZ4

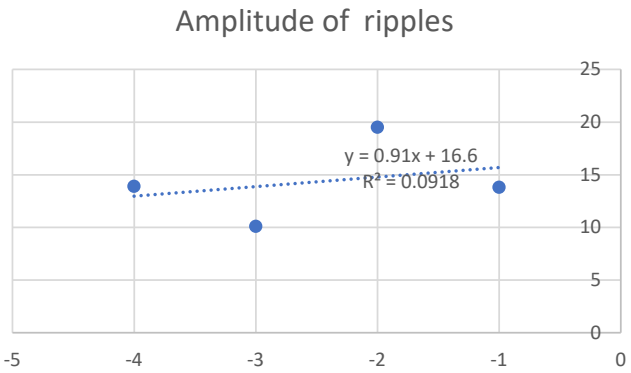

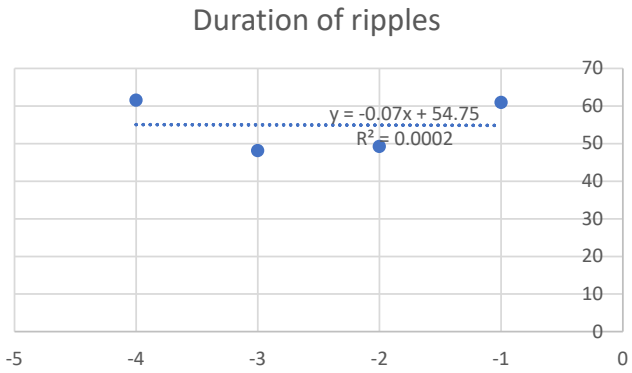

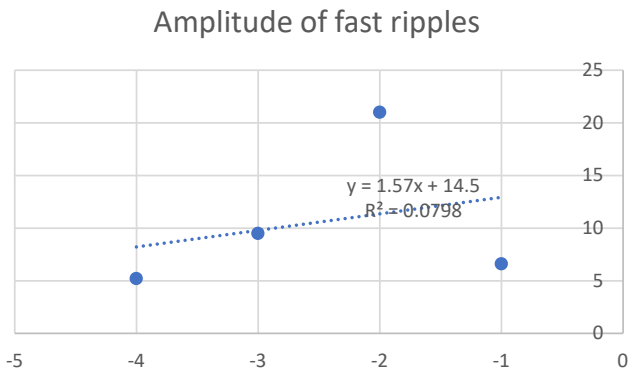

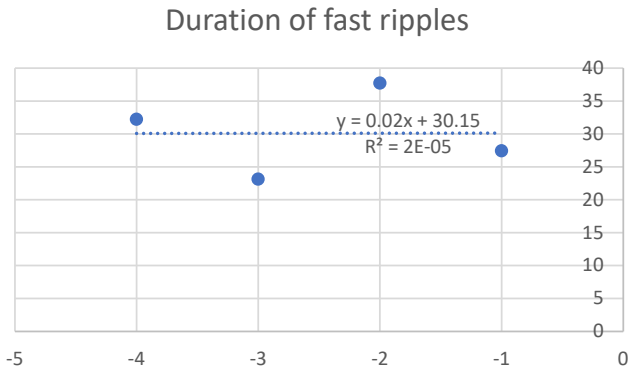

Patient 3 SZ5

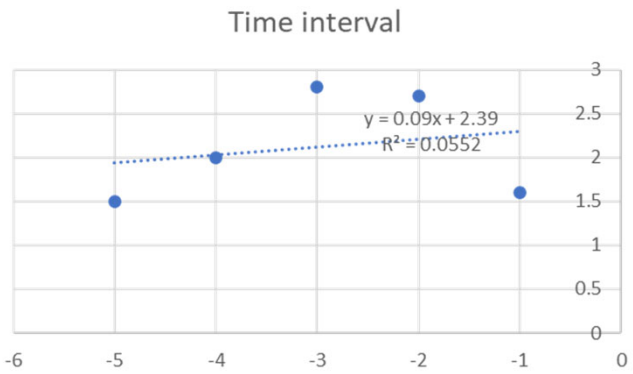

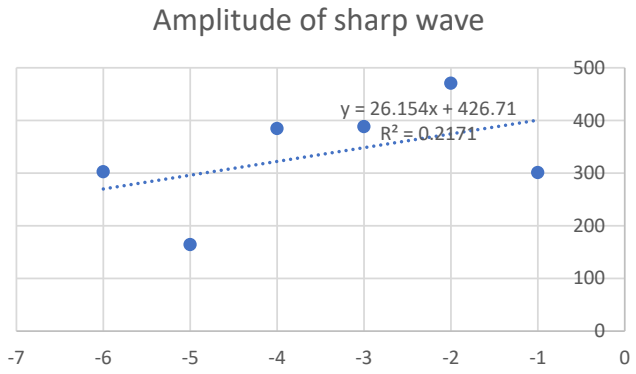

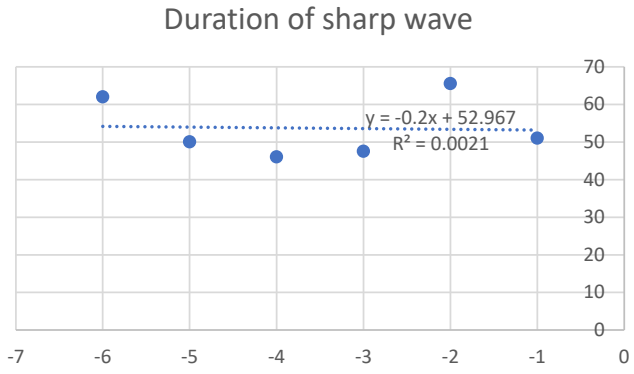

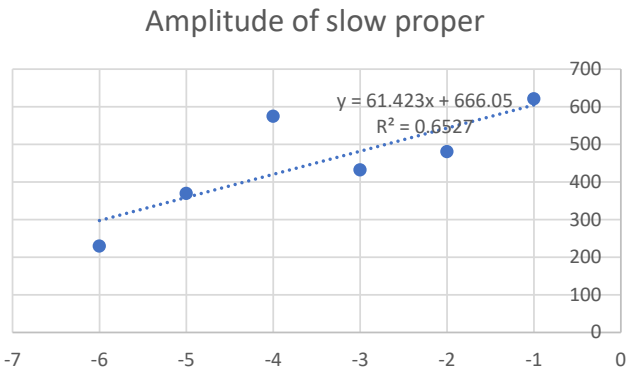

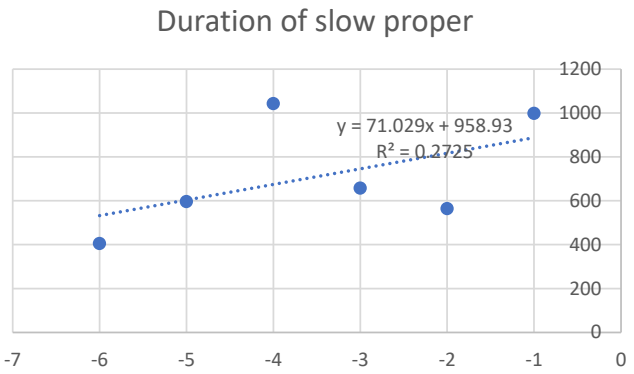

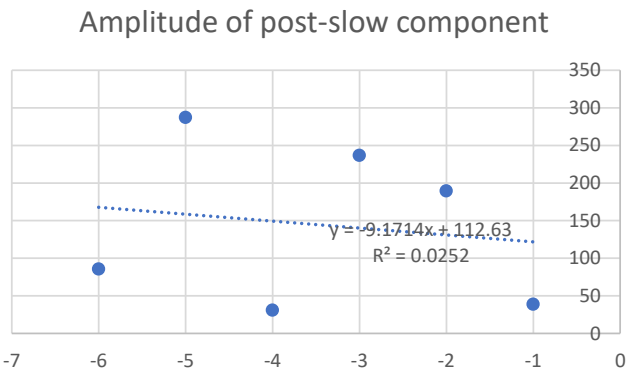

Patient 3 SZ5

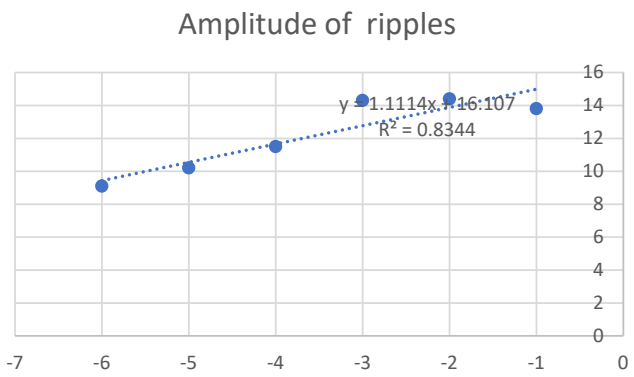

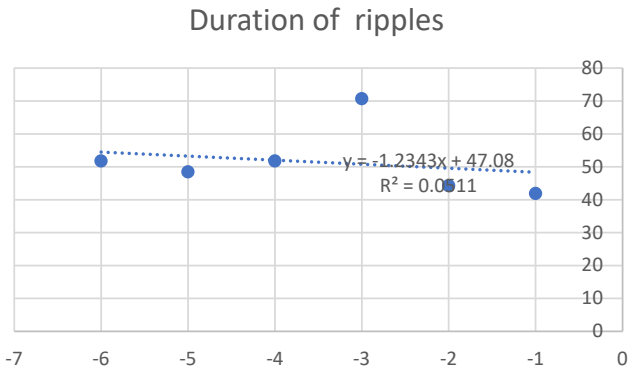

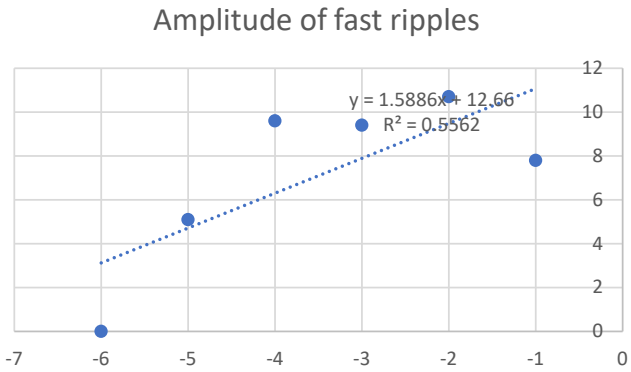

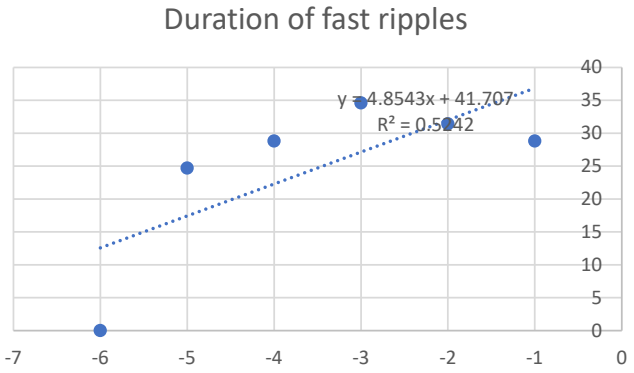

Patient 4 SZ1

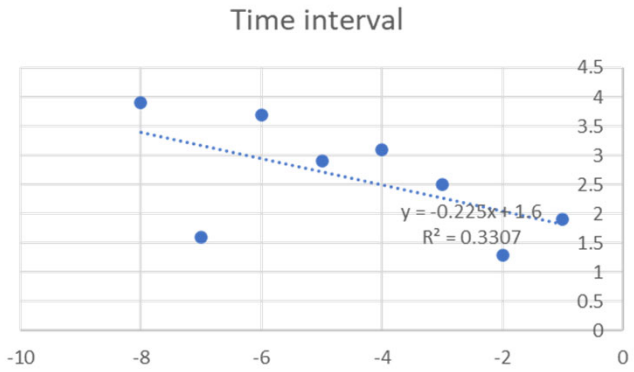

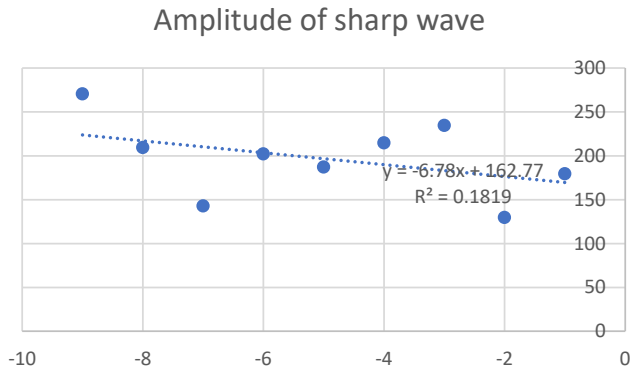

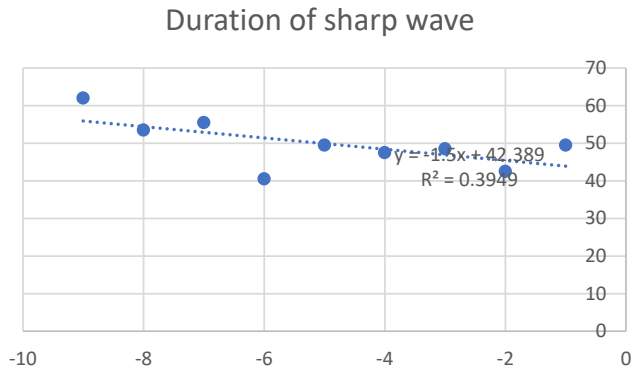

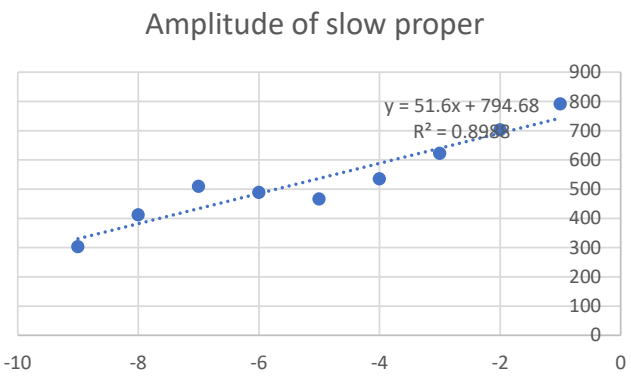

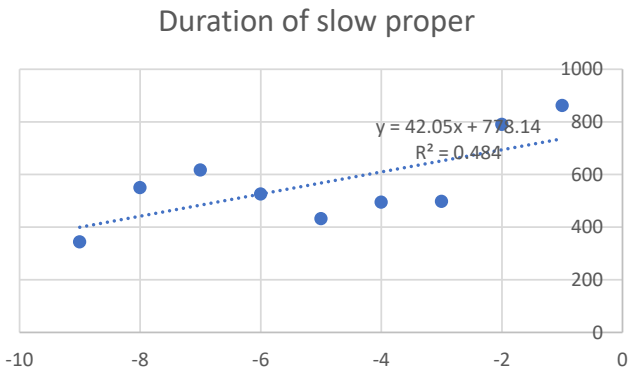

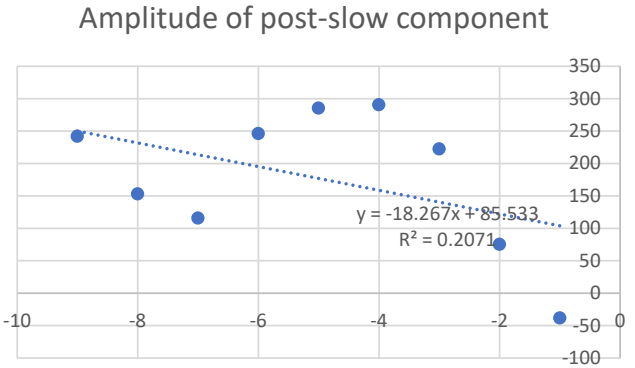

Patient 4 SZ1

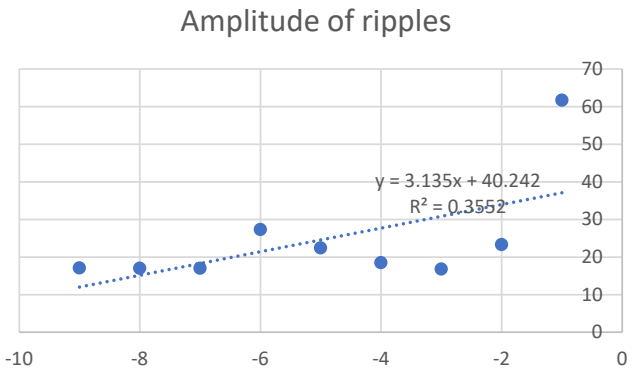

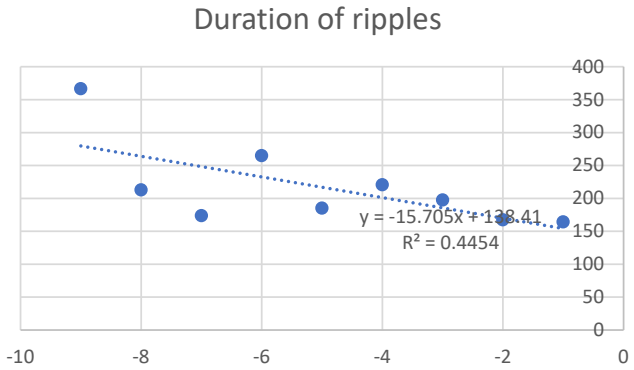

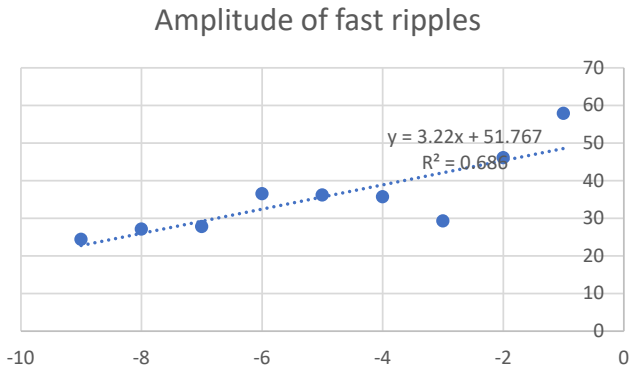

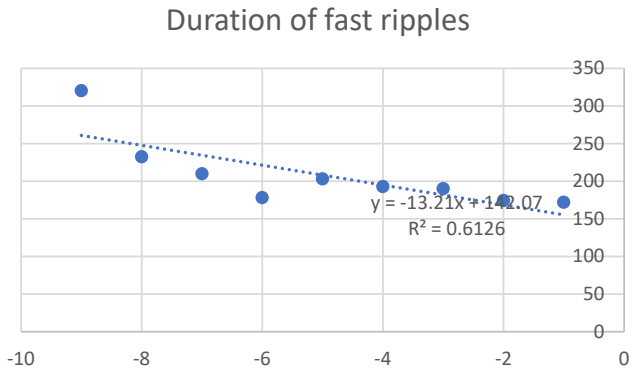

Patient 4 SZ2

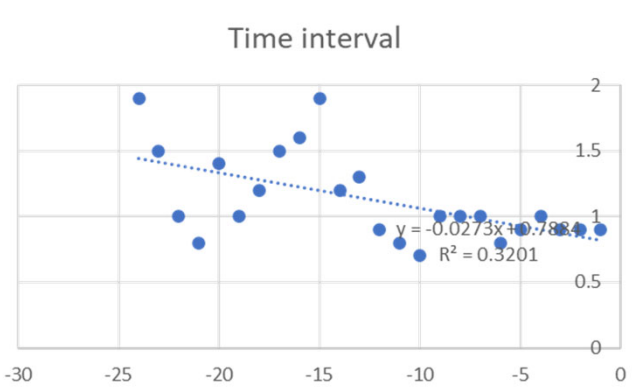

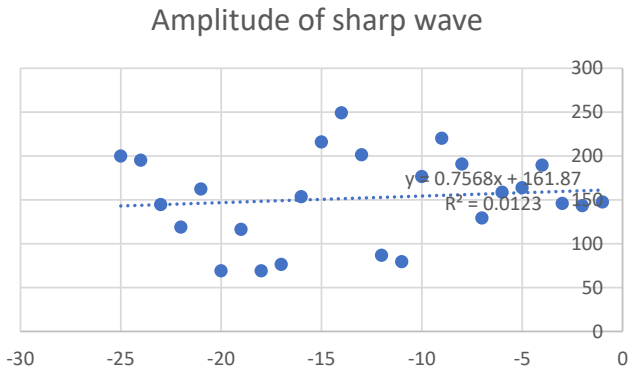

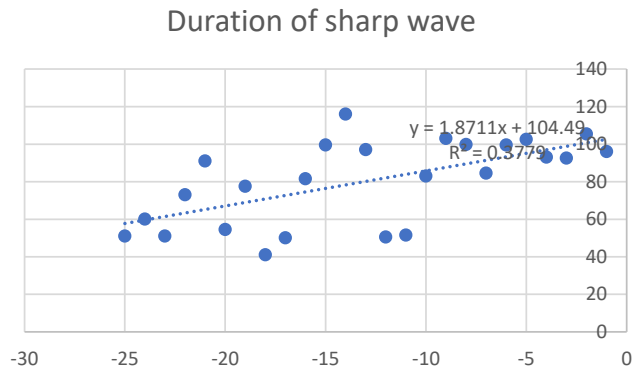

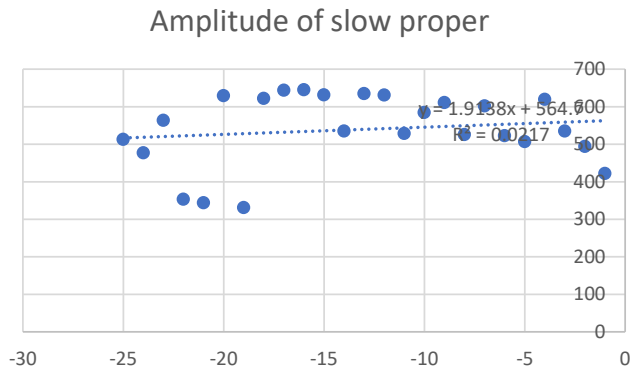

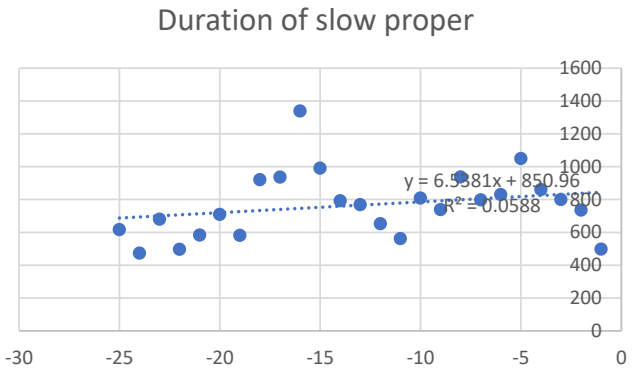

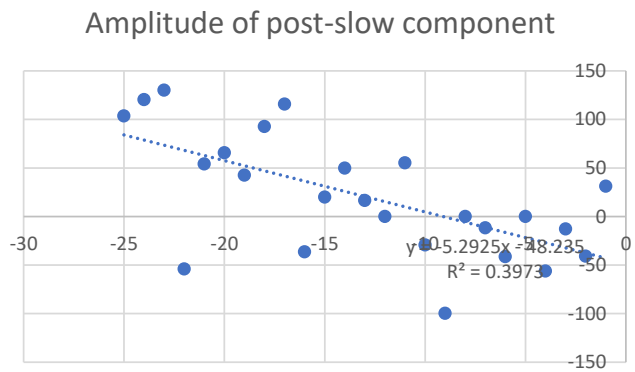

Patient 4 SZ2

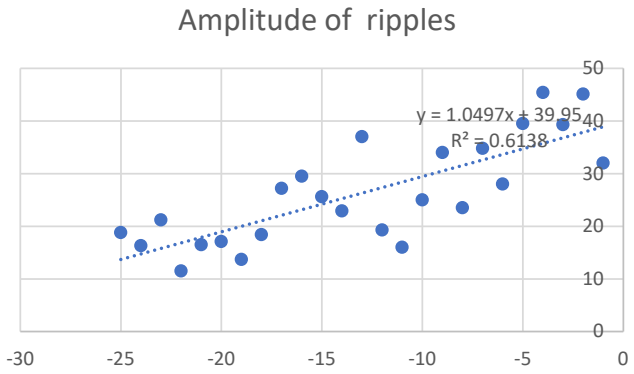

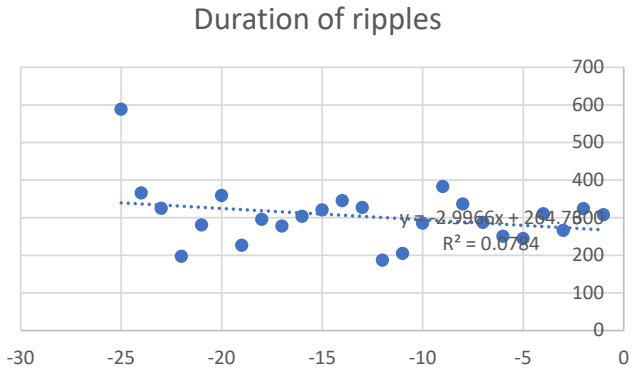

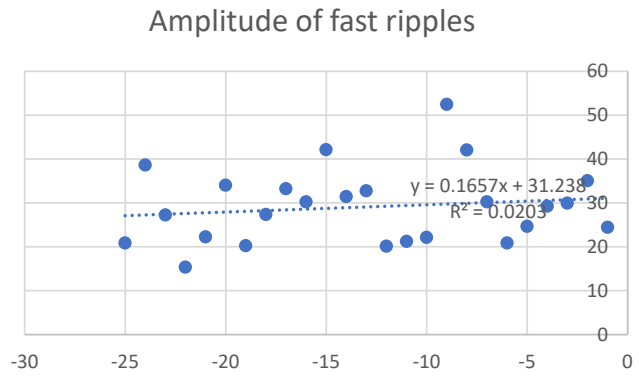

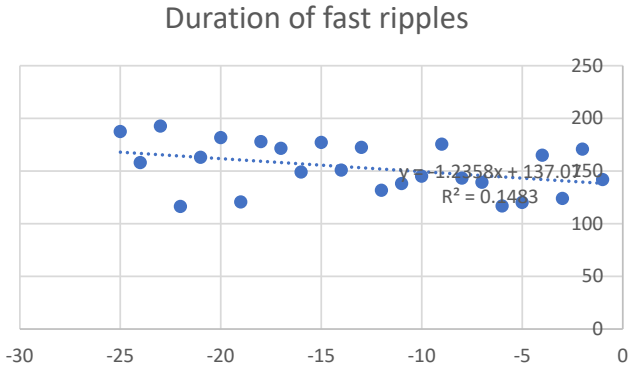

Patient 4 SZ3

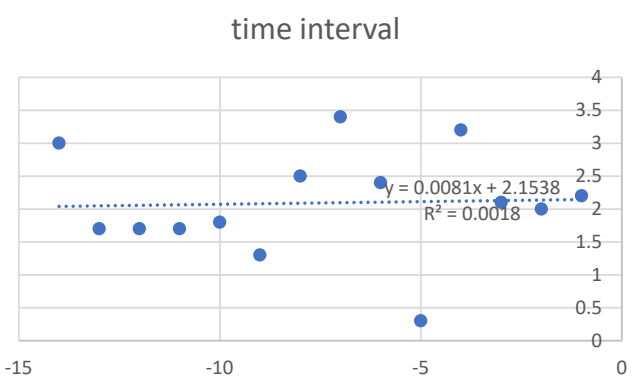

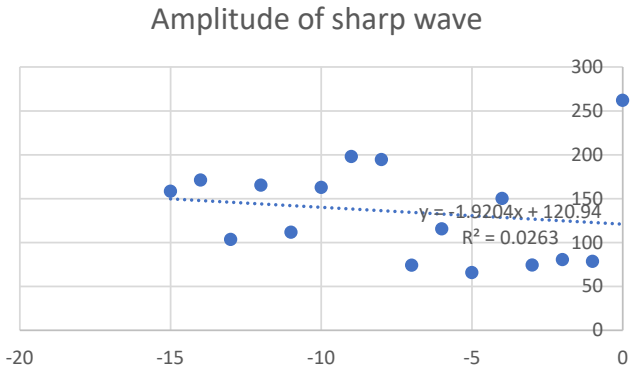

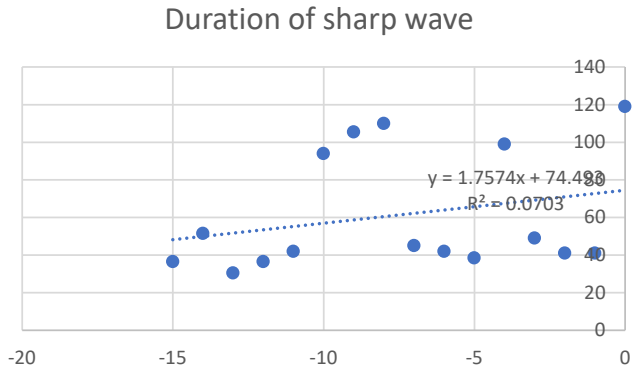

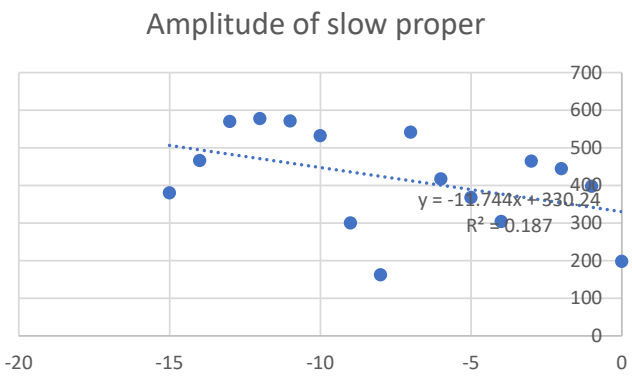

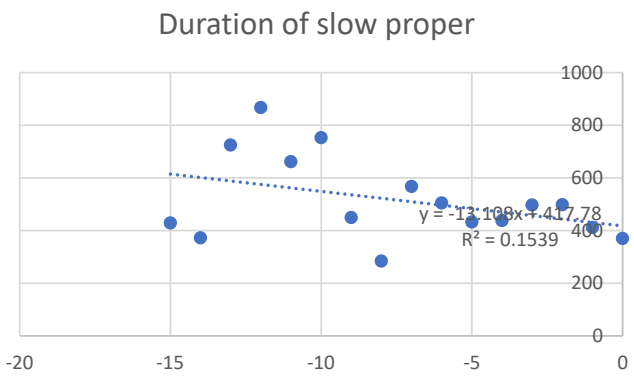

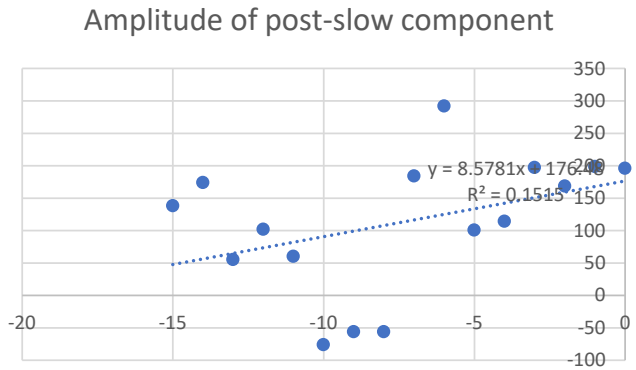

Patient 4 SZ3

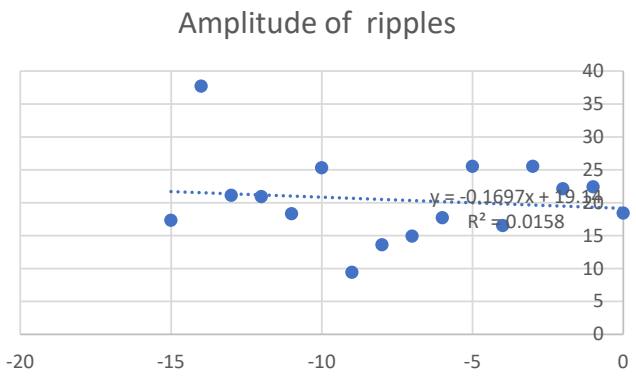

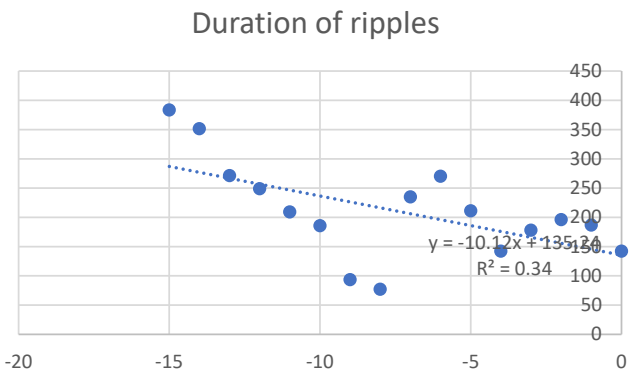

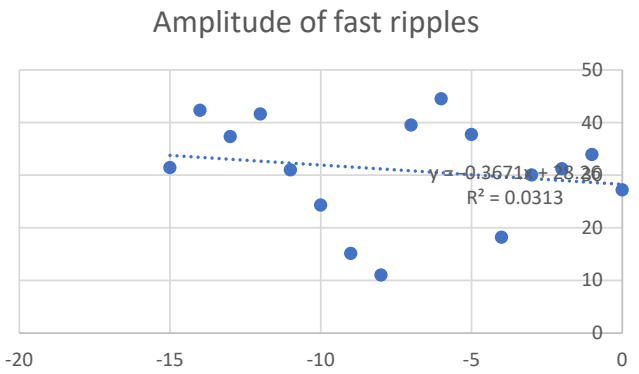

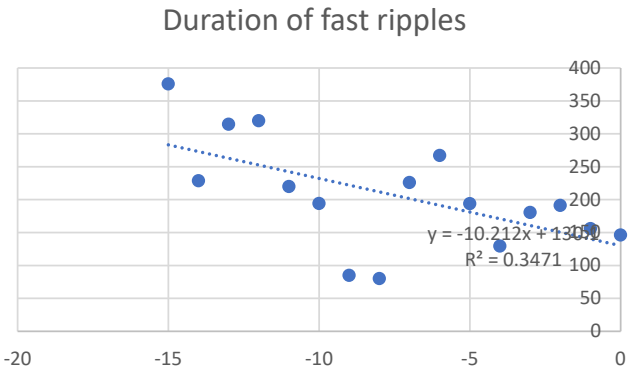

Patient 4 SZ4

Time interval

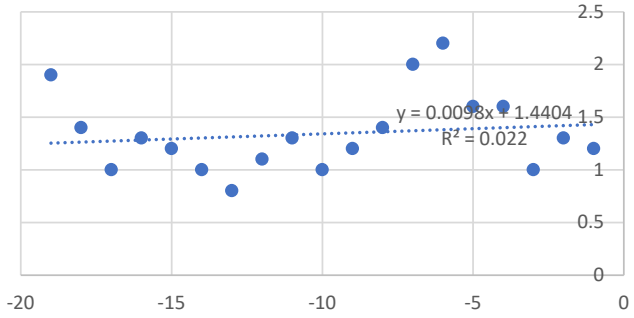

Amplitude of sharp wave

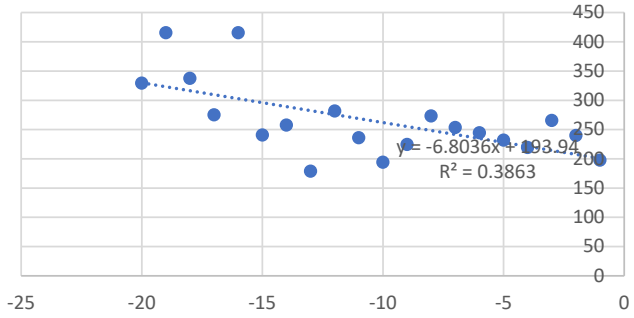

Duration of sharp wave

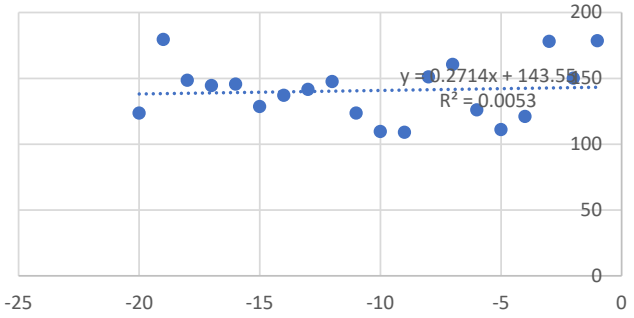

Amplitude of slow proper

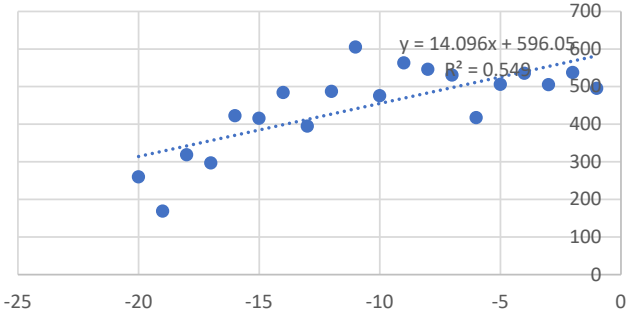

Duration of slow proper

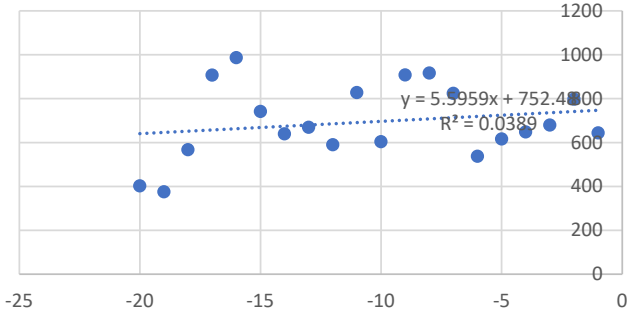

Amplitude of post-slow component

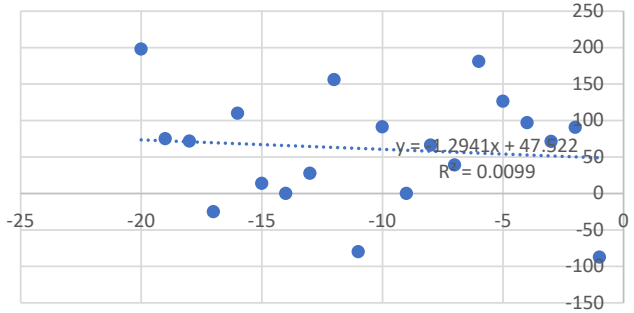

Patient 4 SZ4

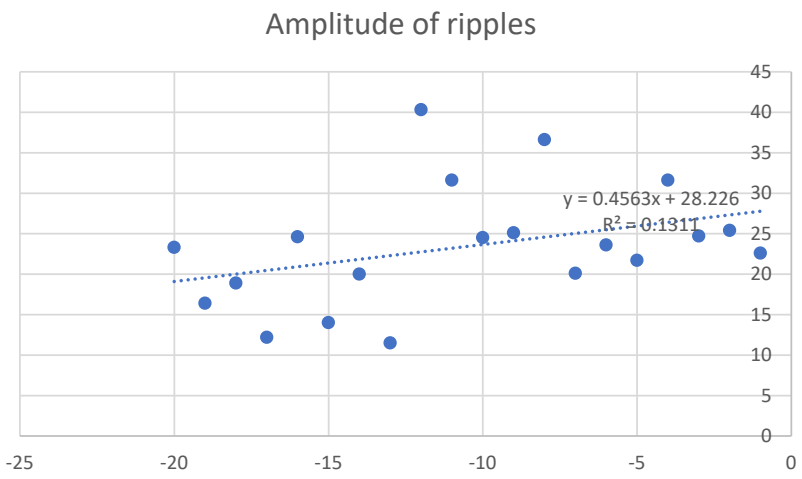

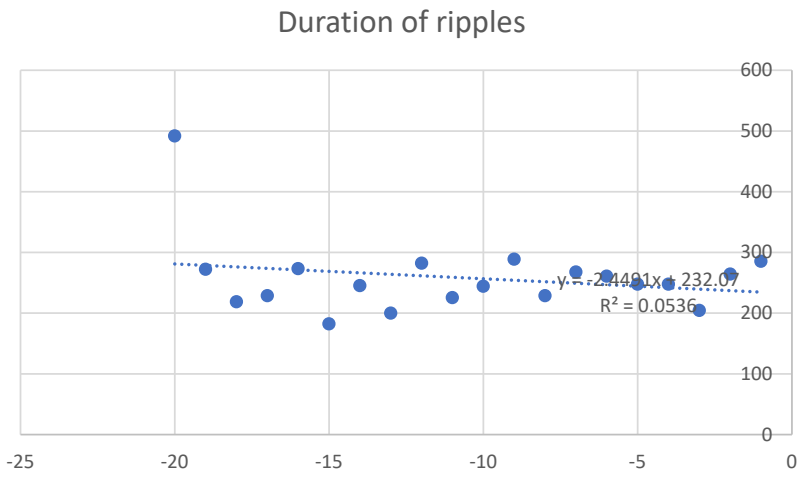

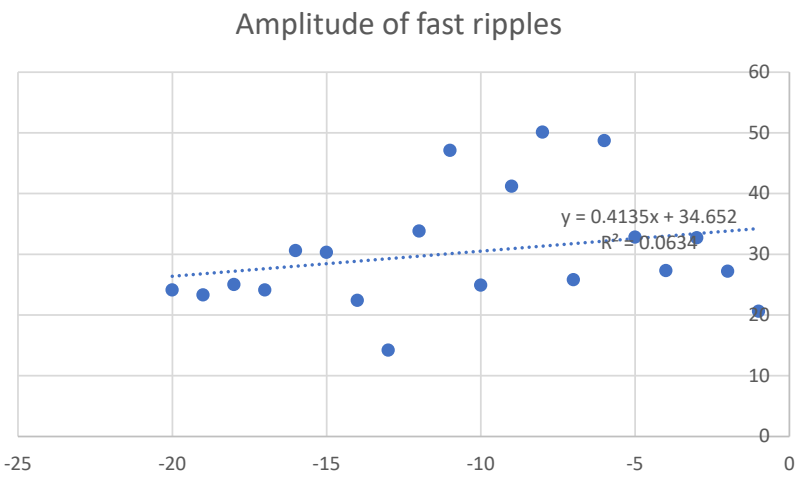

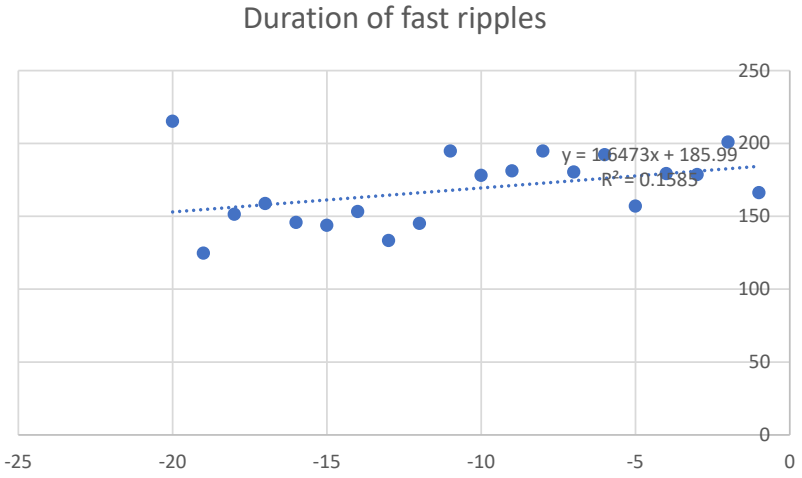

Patient 4 SZ5

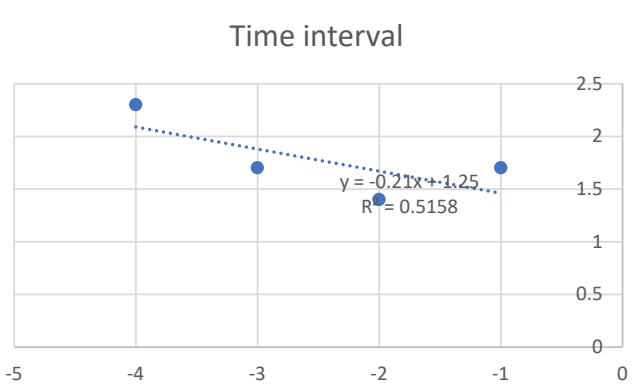

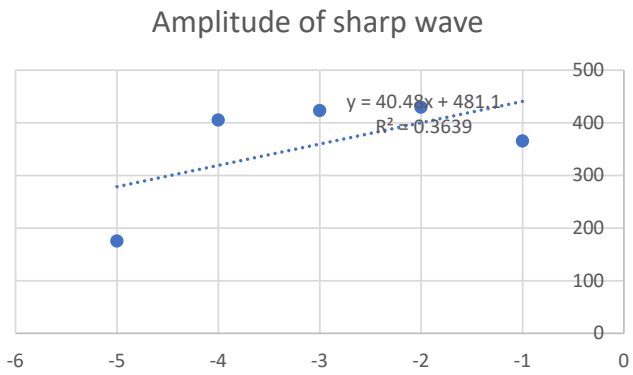

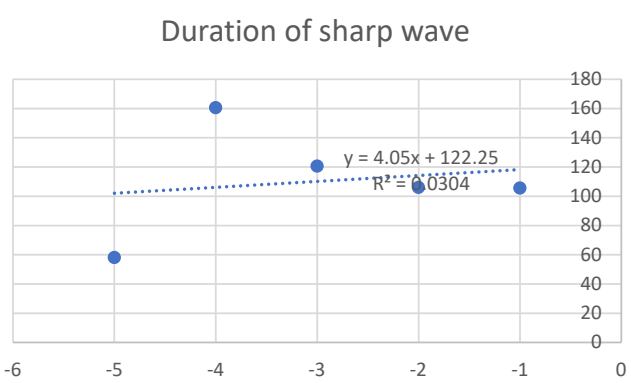

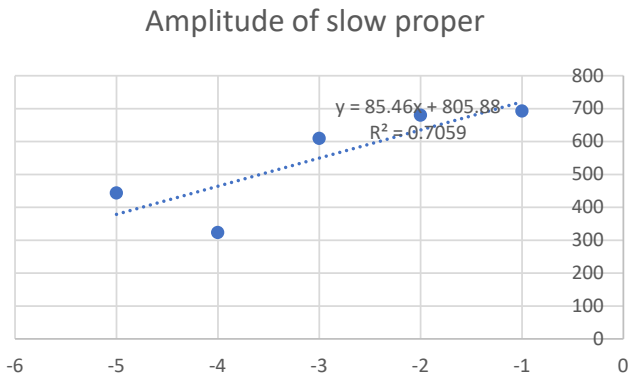

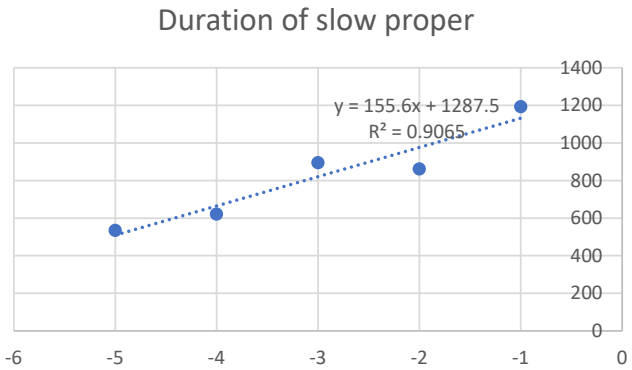

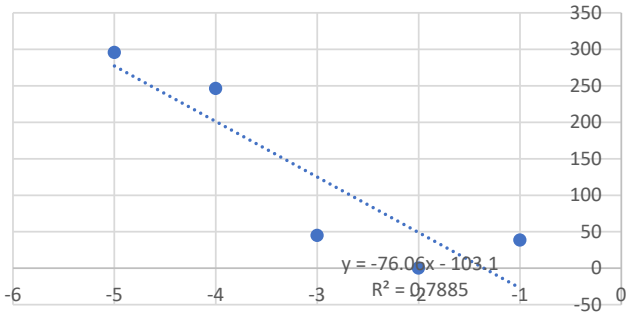

Patient 4 SZ5

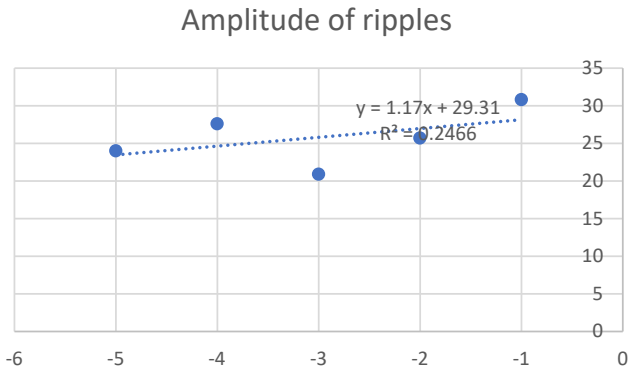

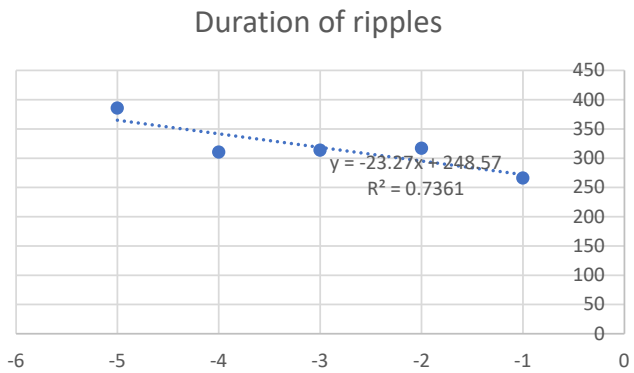

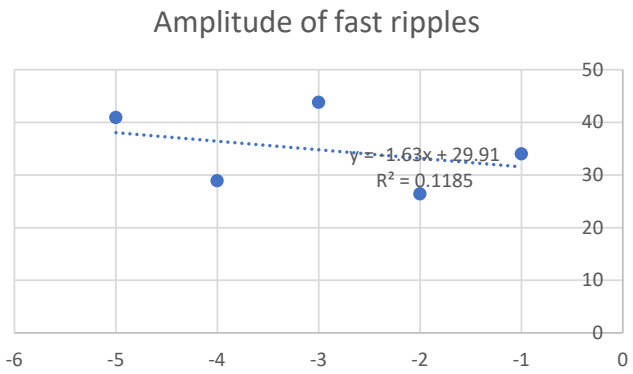

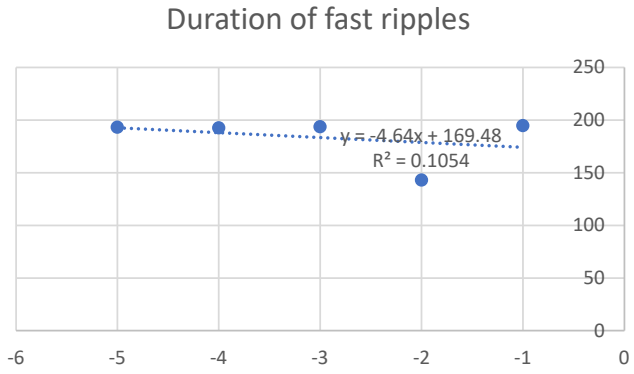

Patient 5 SZ1

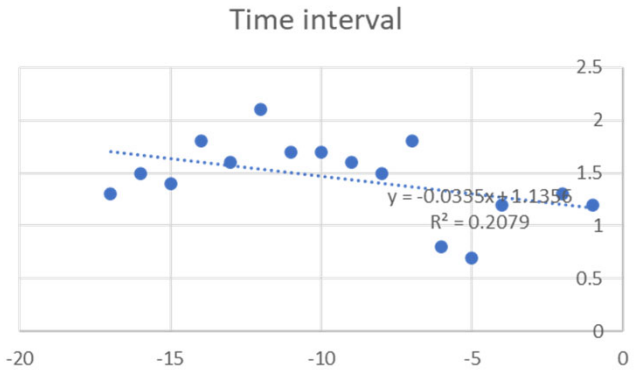

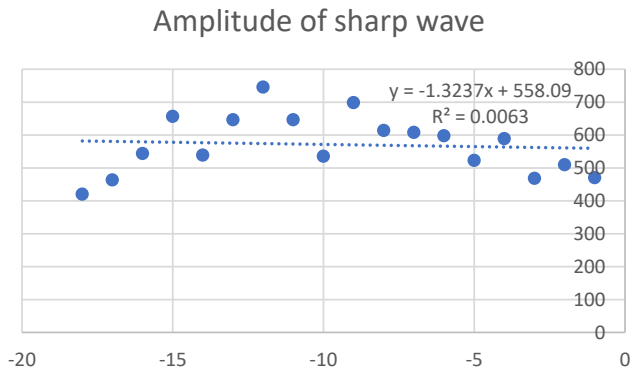

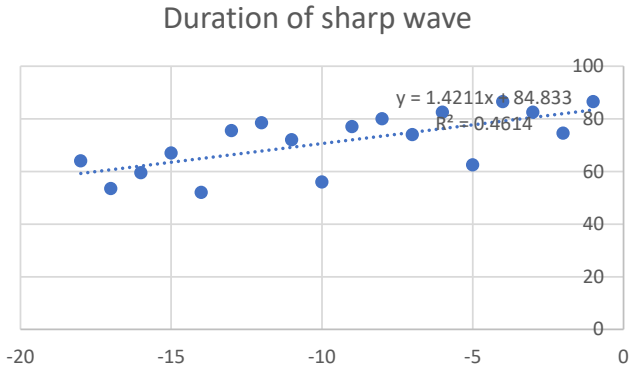

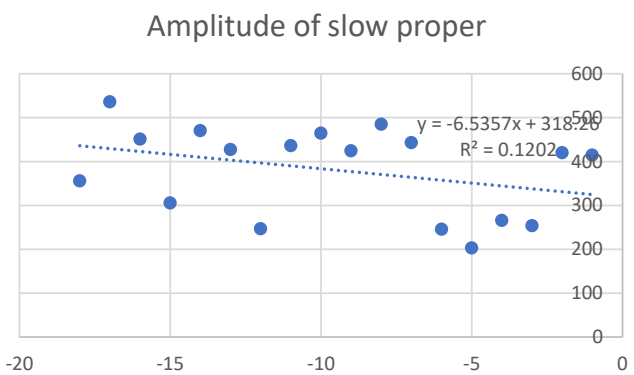

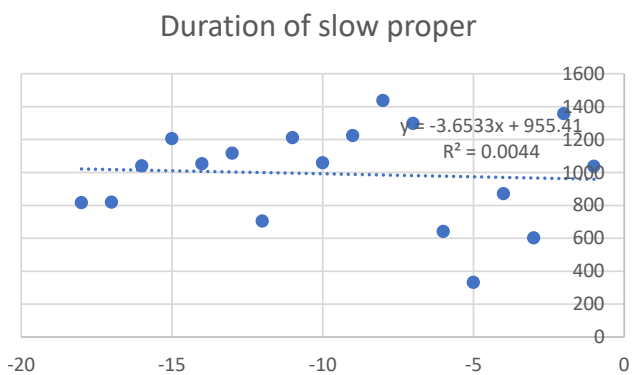

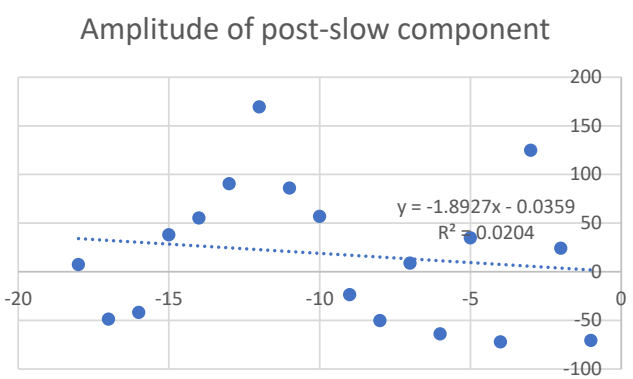

Patient 5 SZ1

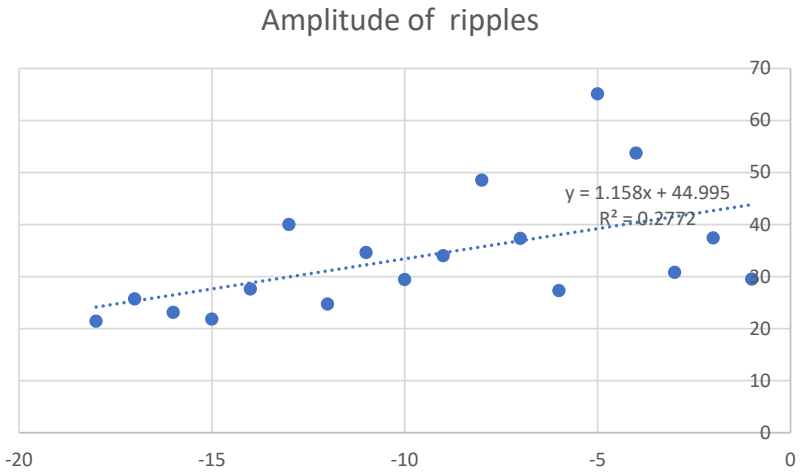

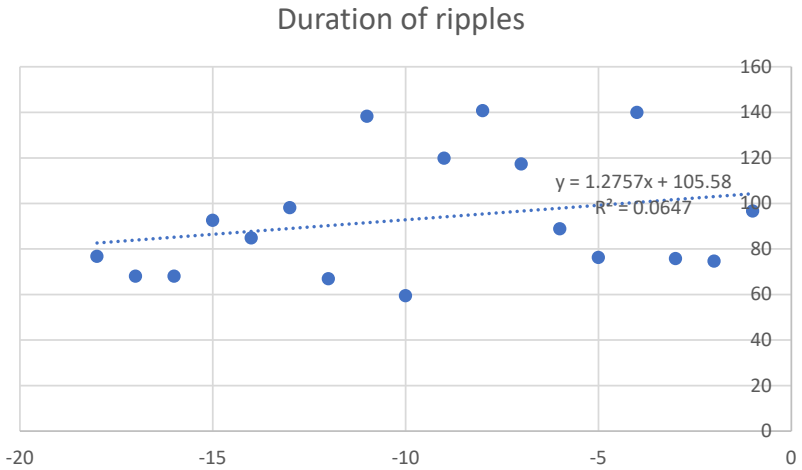

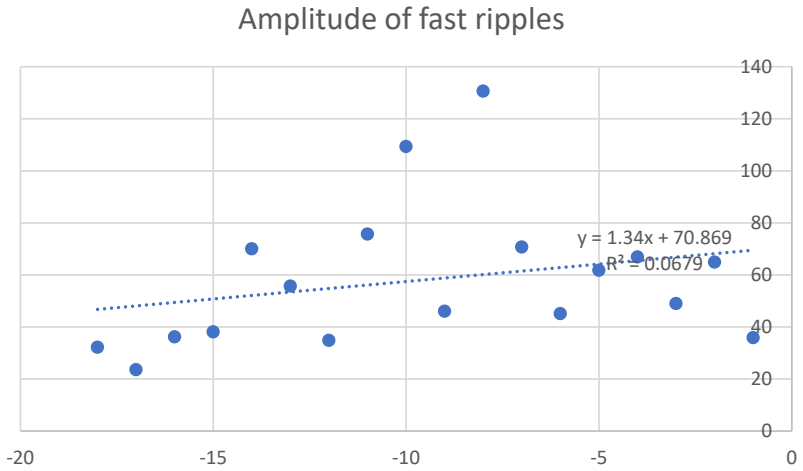

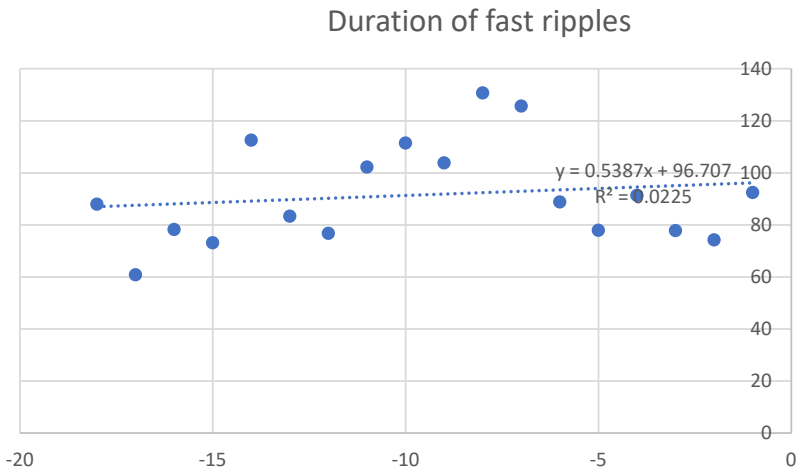

Patient 5 SZ2

Time interval

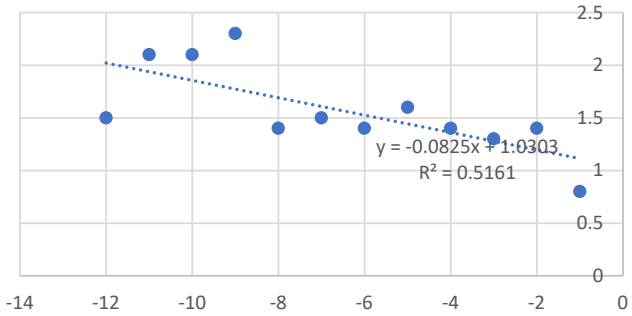

Amplitude of sharp wave

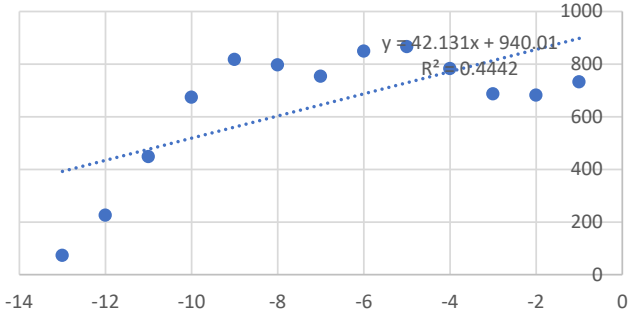

Duration of sharp wave

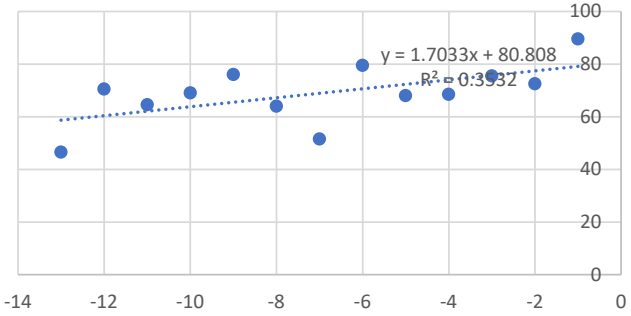

Amplitude of slow proper

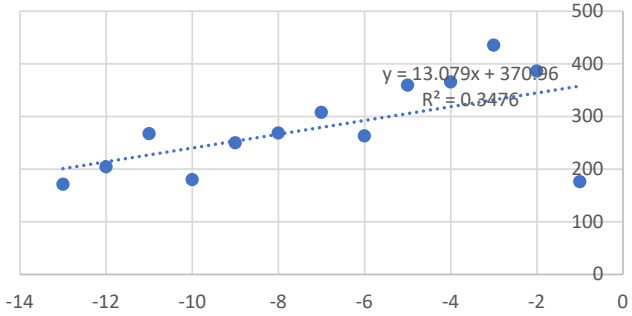

Duration of slow proper

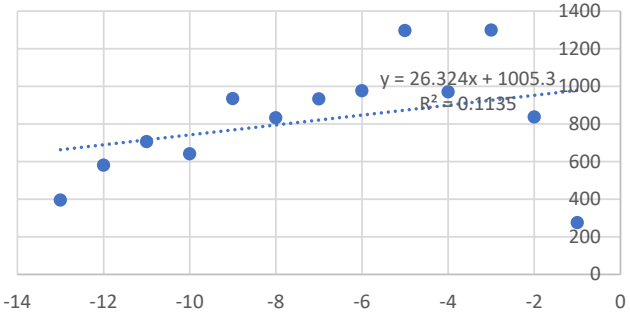

Amplitude of post-slow component

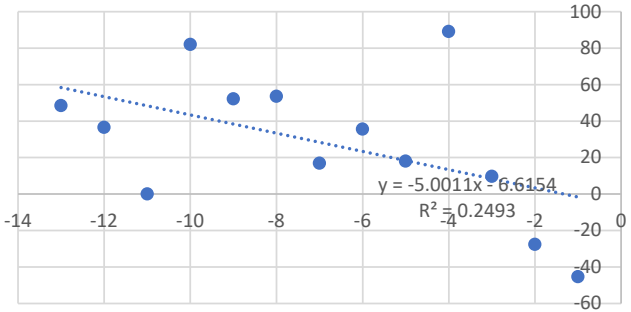

Patient 5 SZ2

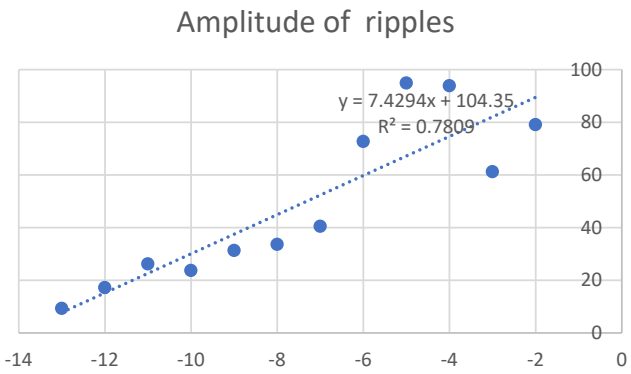

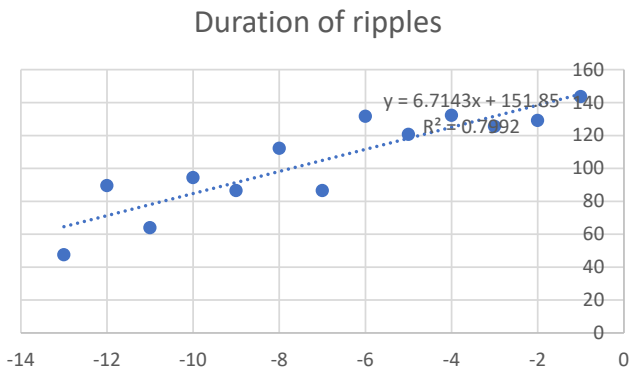

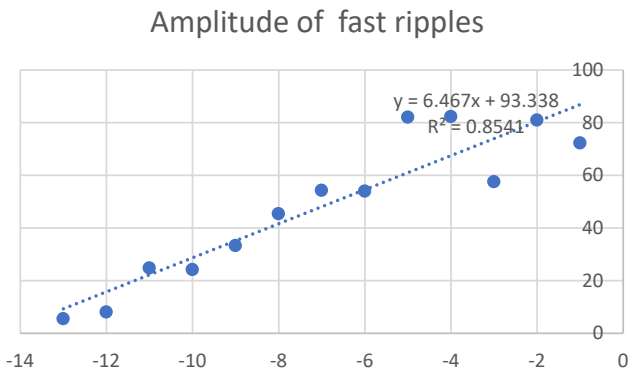

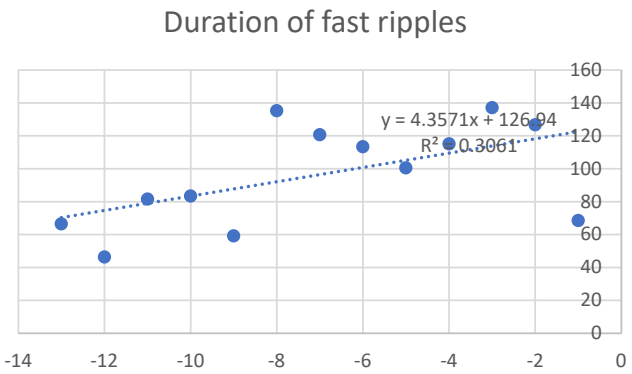

Patient 5 SZ3

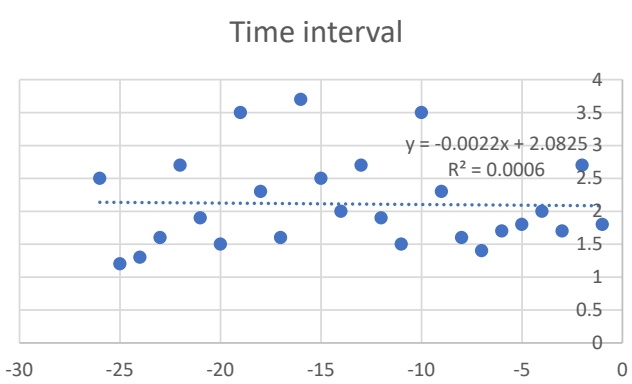

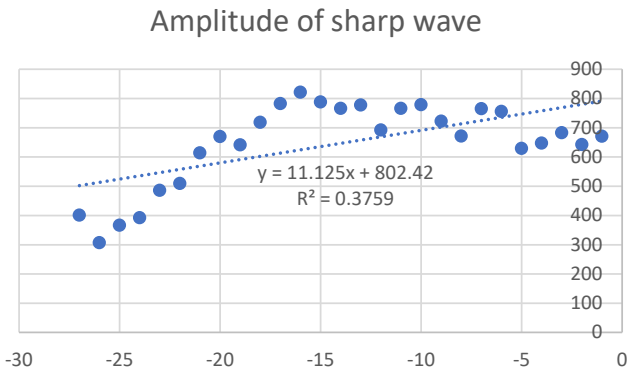

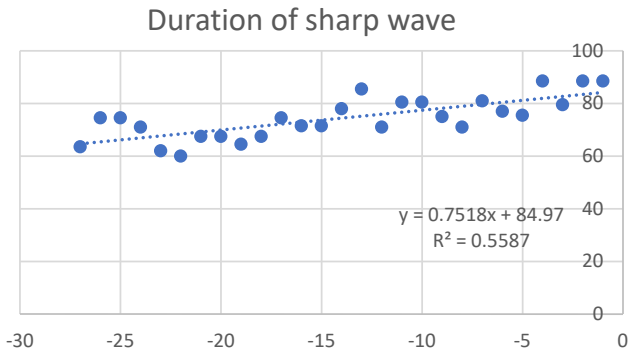

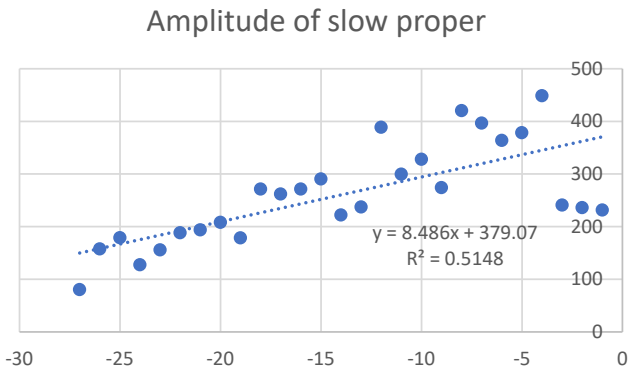

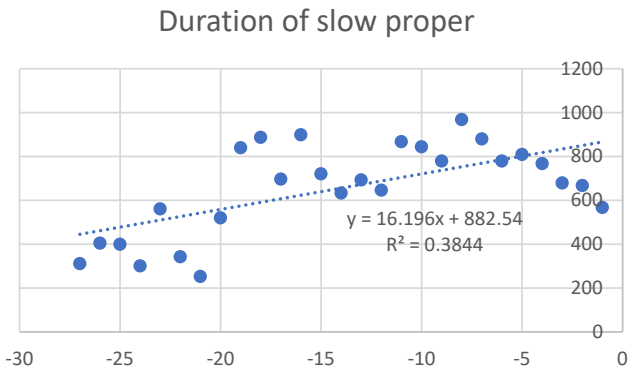

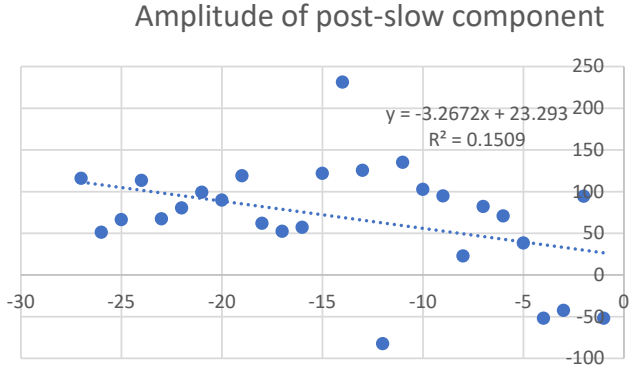

Patient 5 SZ3

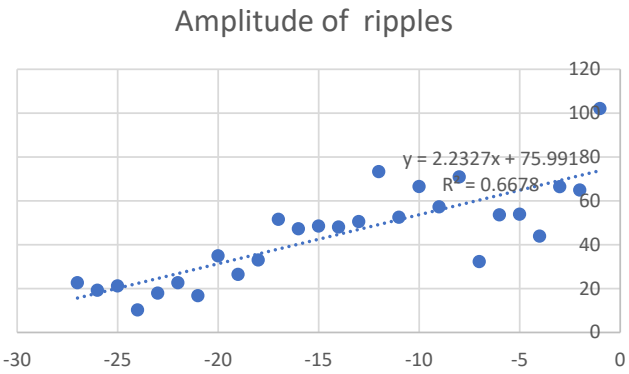

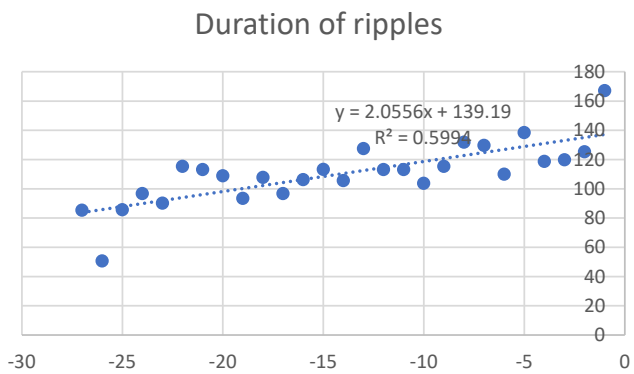

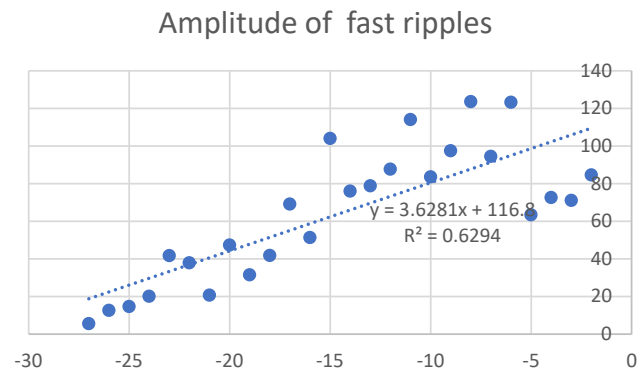

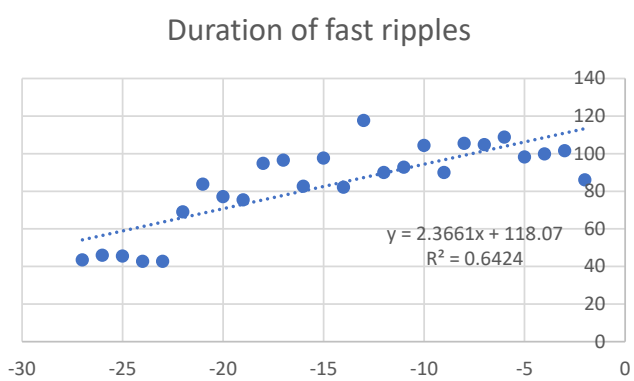

Patient 6 SZ1

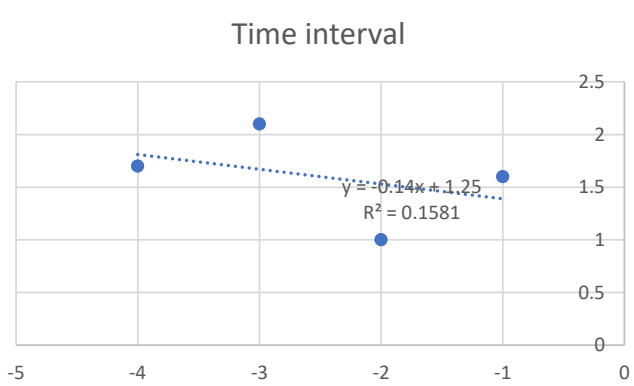

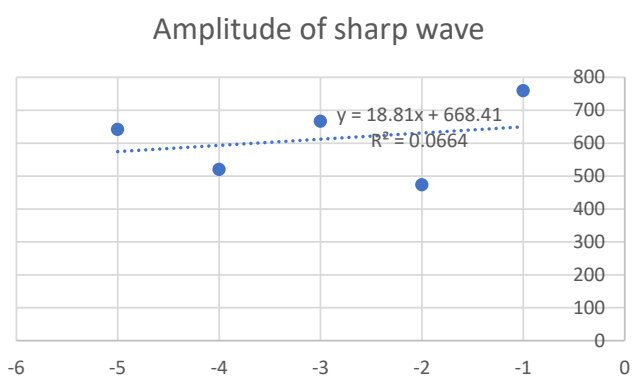

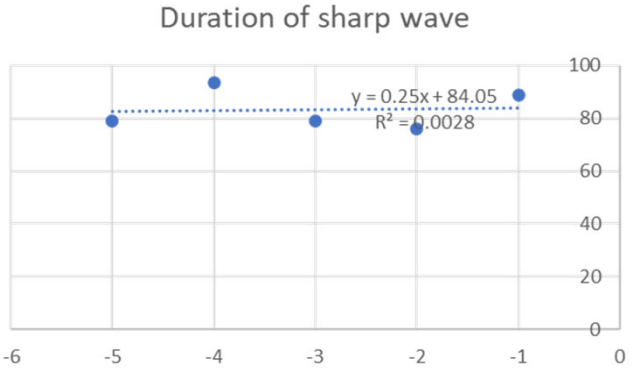

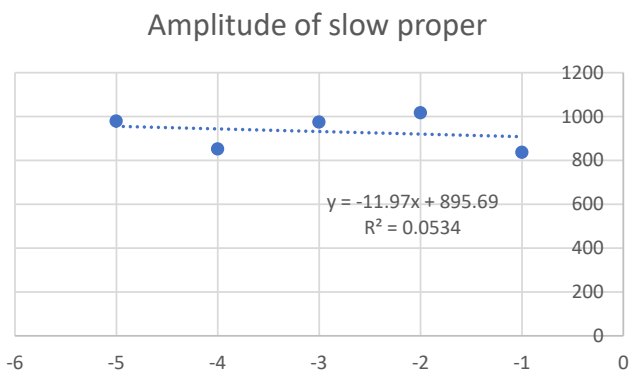

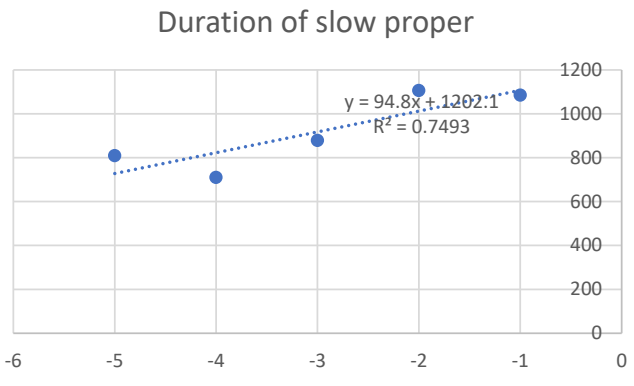

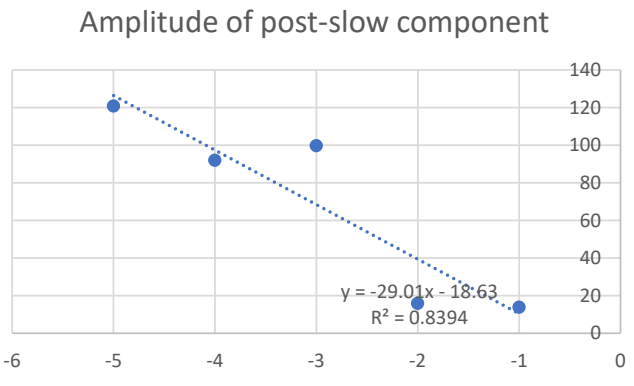

Patient 6 SZ1

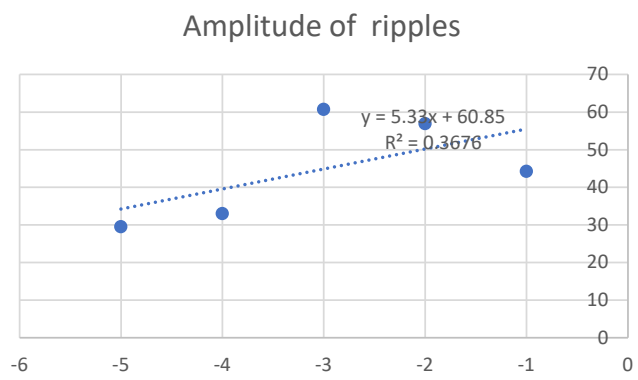

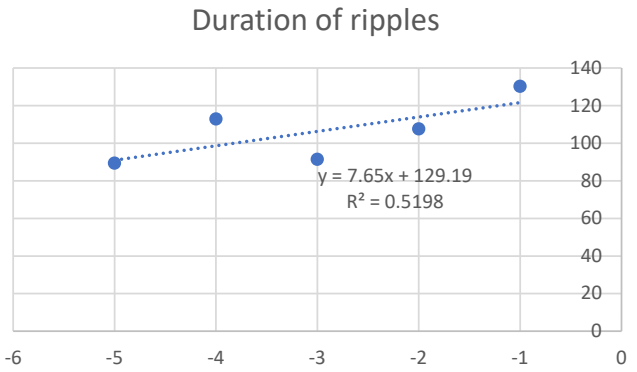

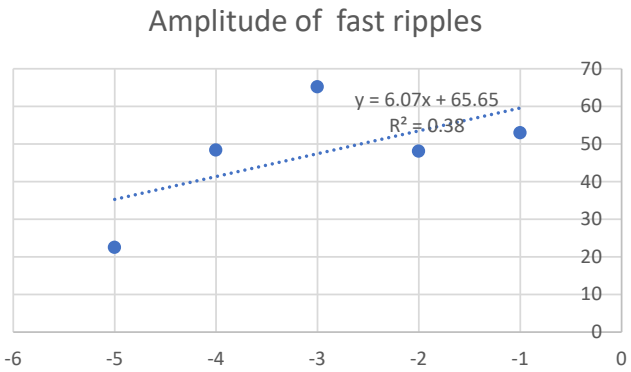

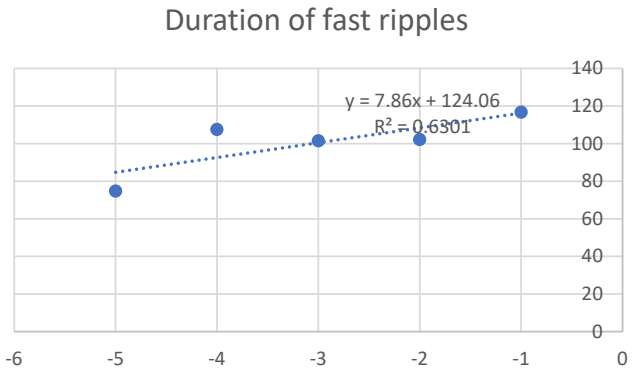

Patient 6 SZ2

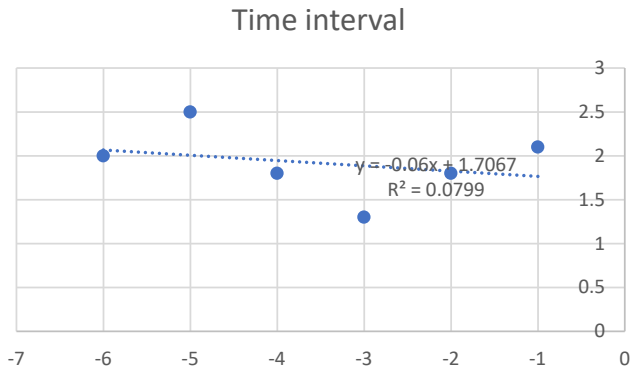

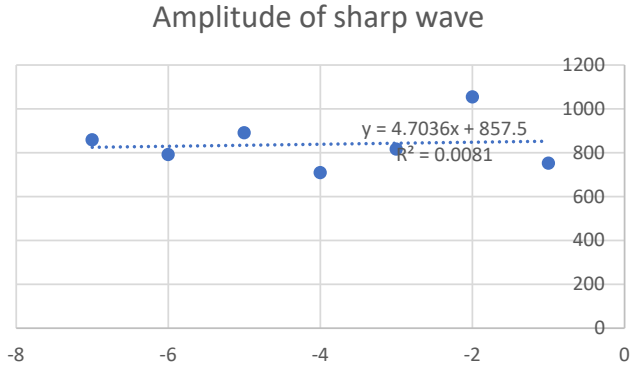

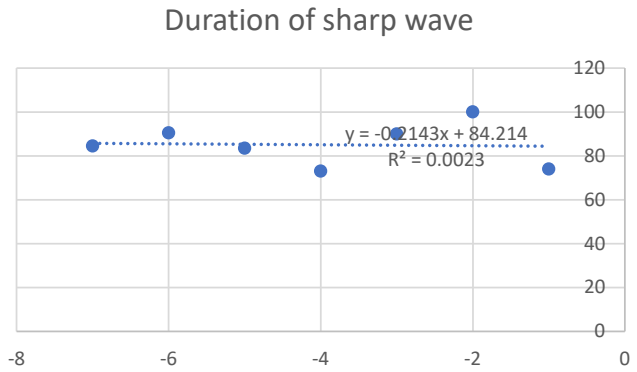

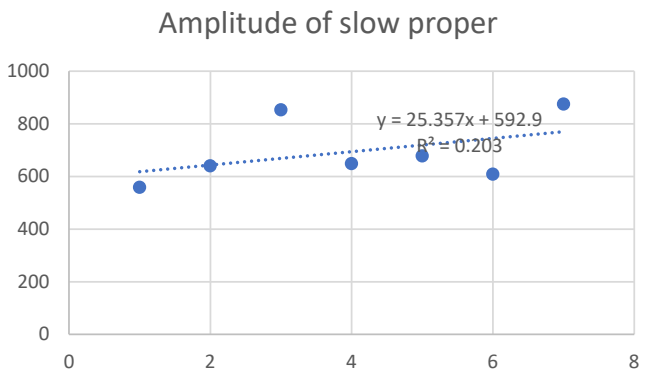

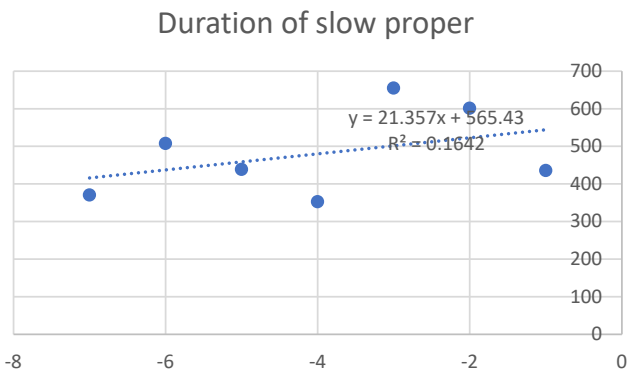

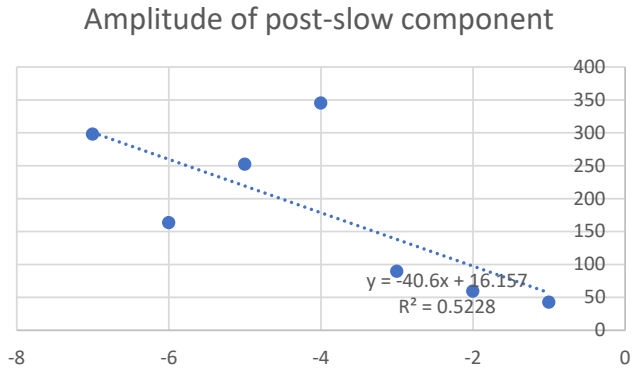

Patient 6 SZ2

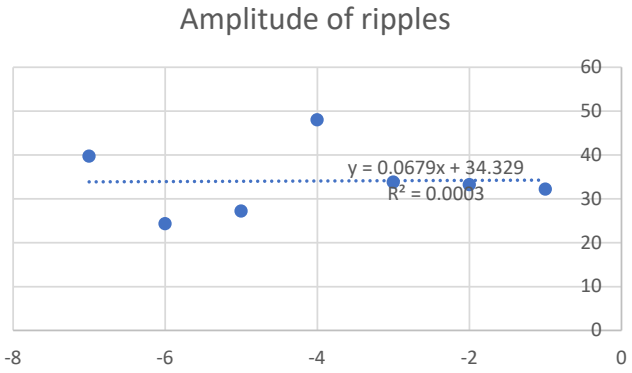

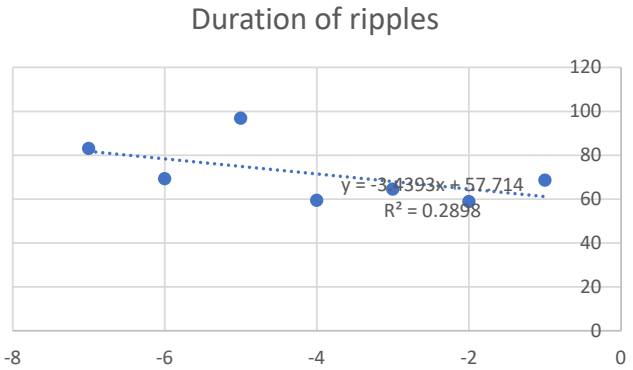

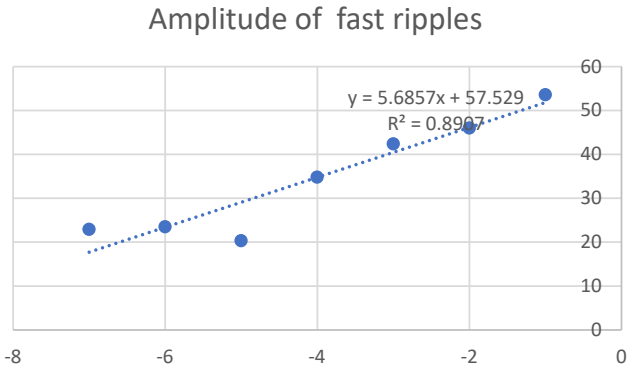

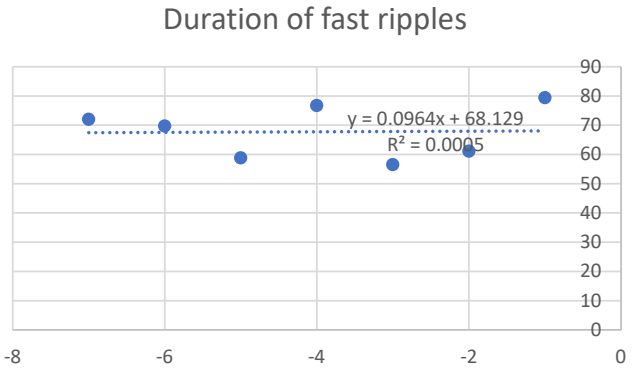

Patient 6 SZ3

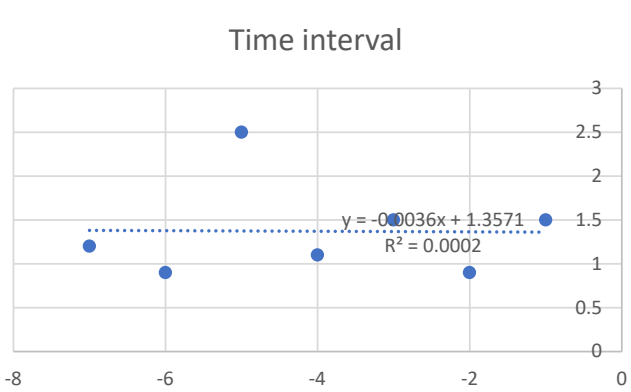

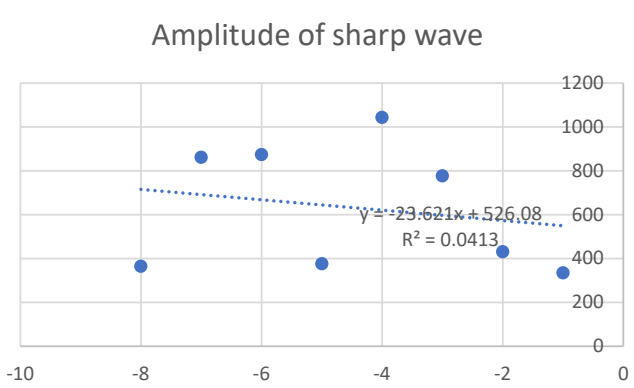

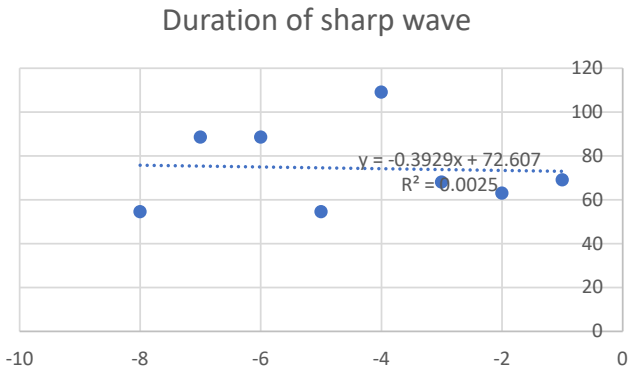

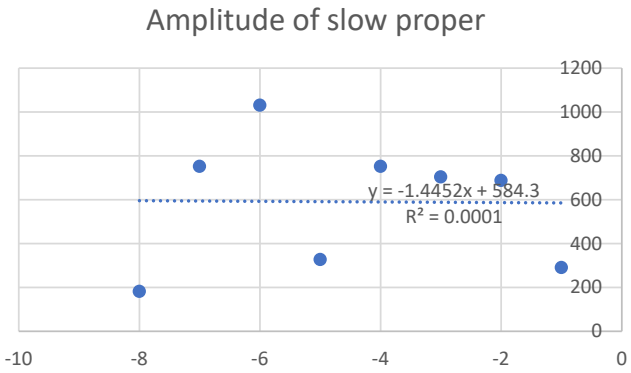

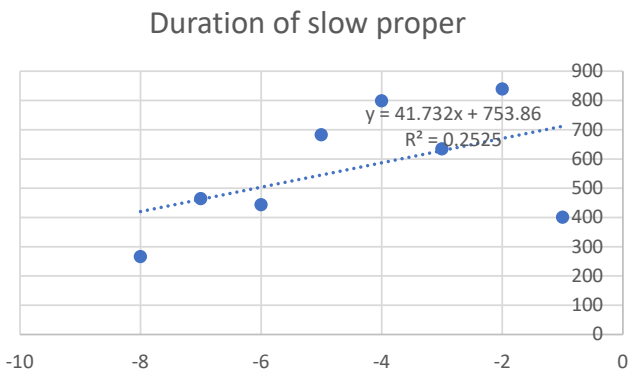

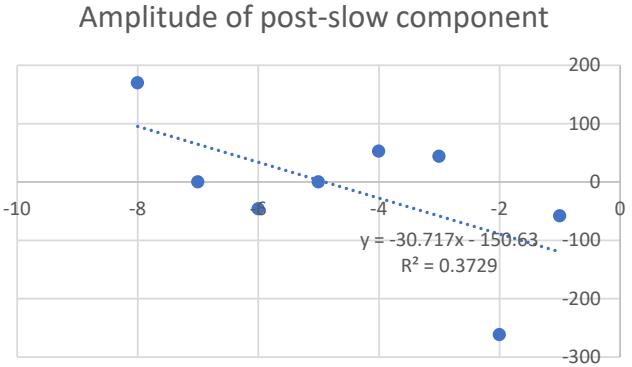

Patient 6 SZ3

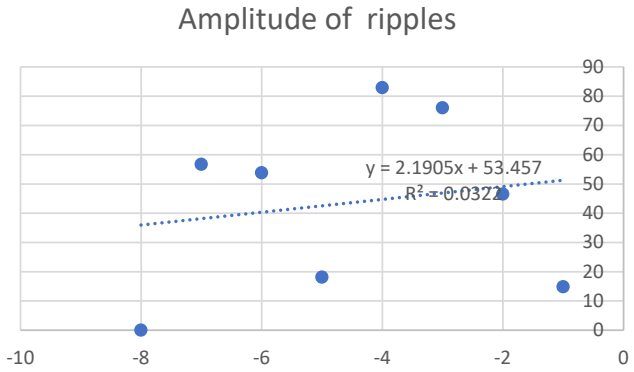

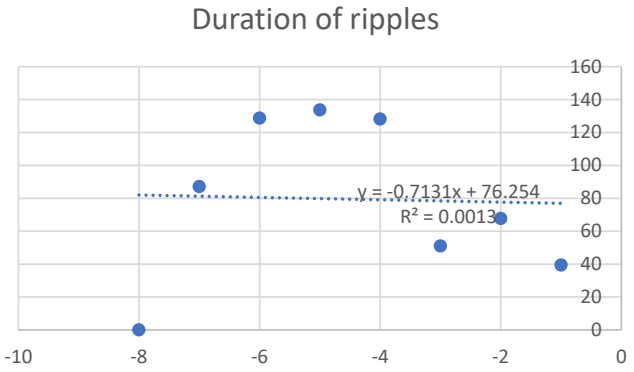

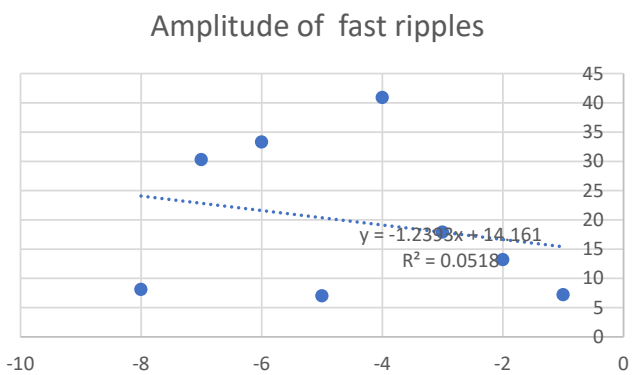

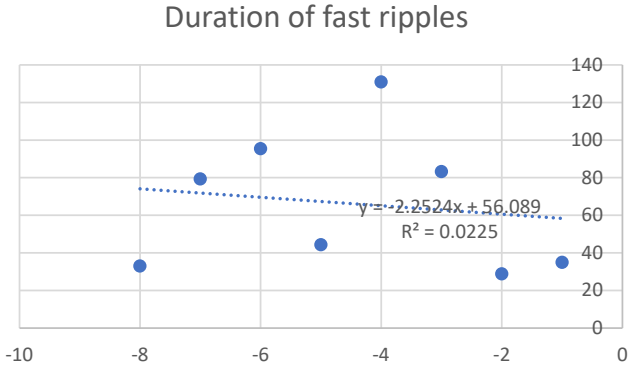

Patient 7 SZ1

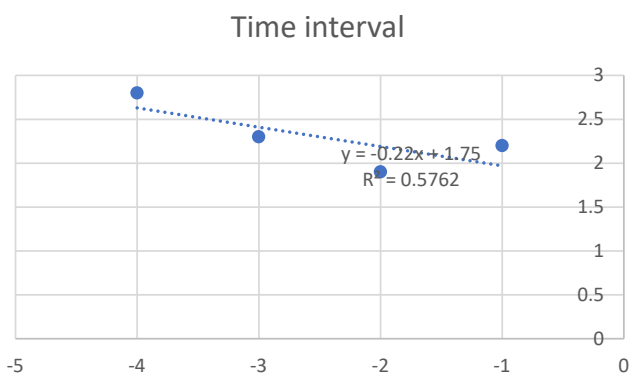

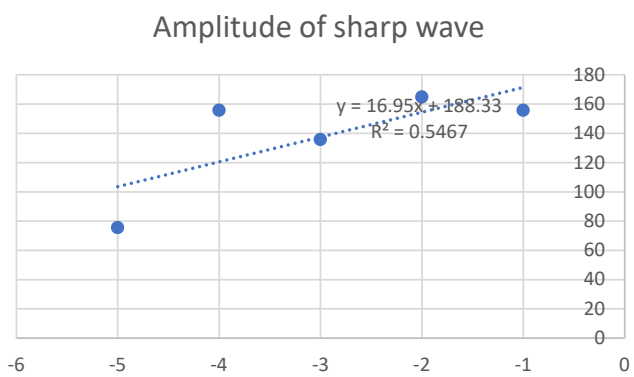

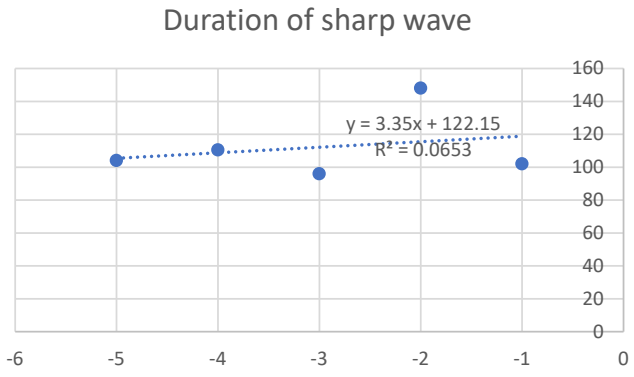

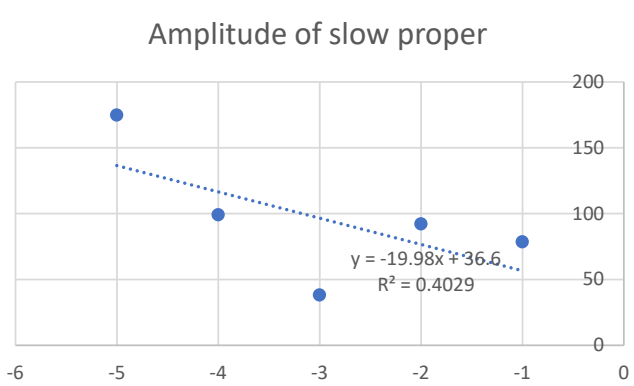

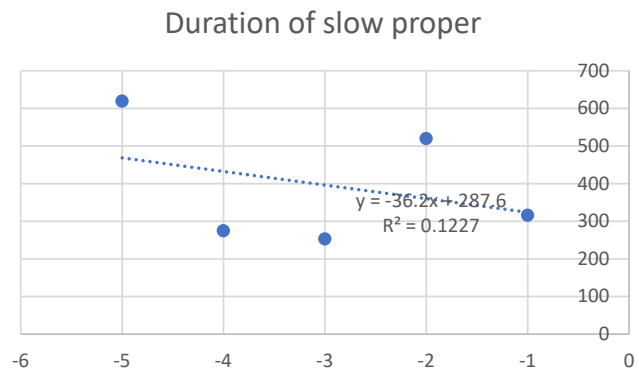

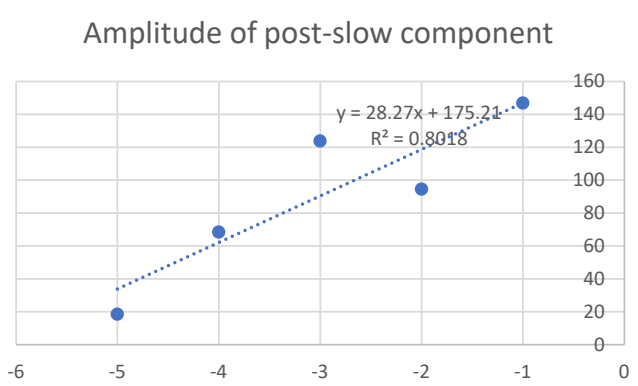

Patient 7 SZ1

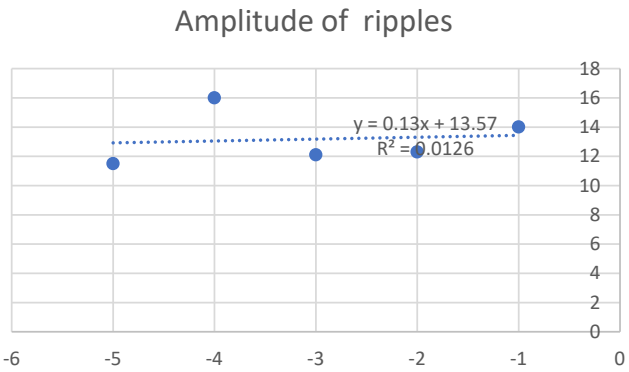

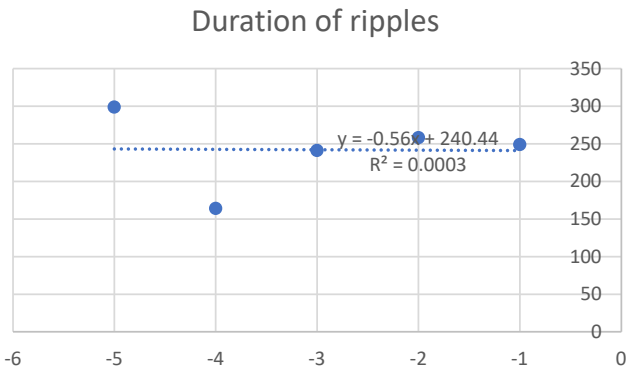

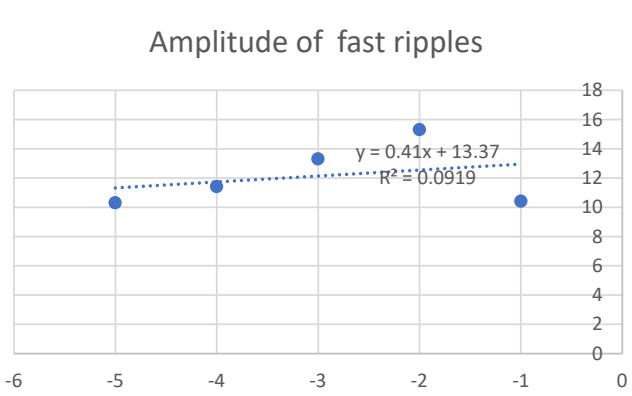

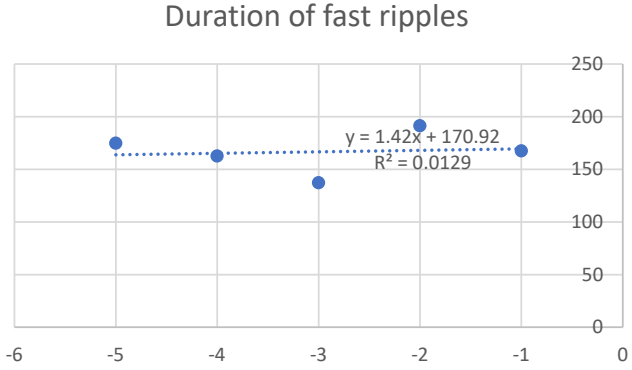

Patient 7 SZ2

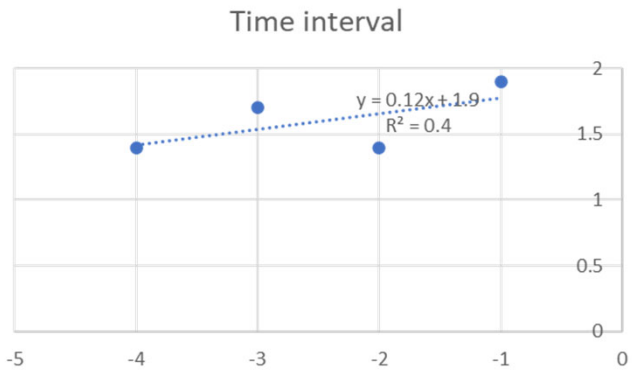

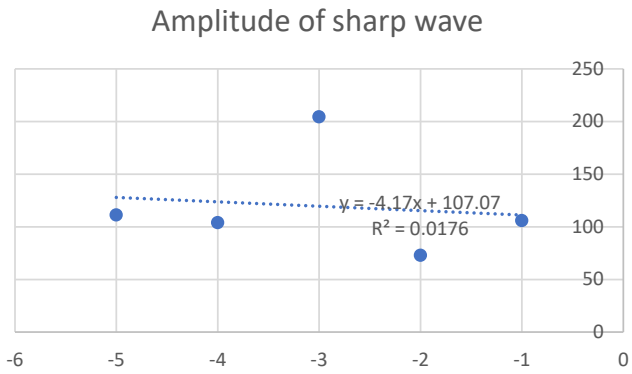

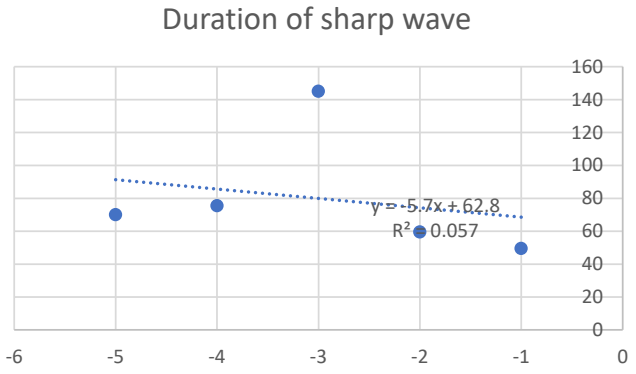

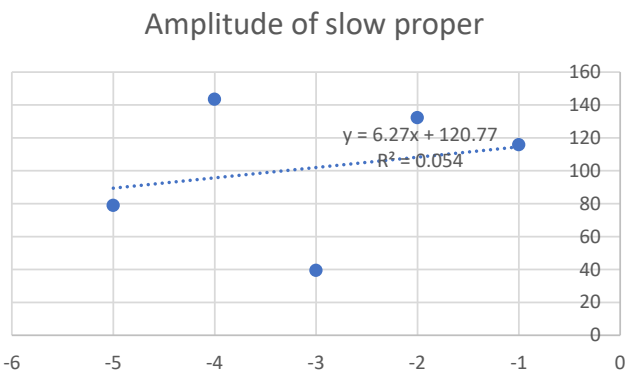

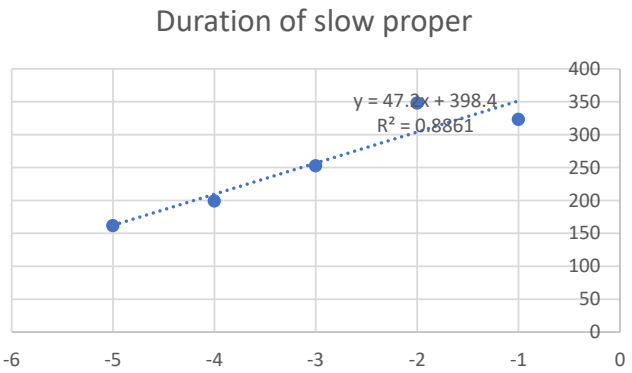

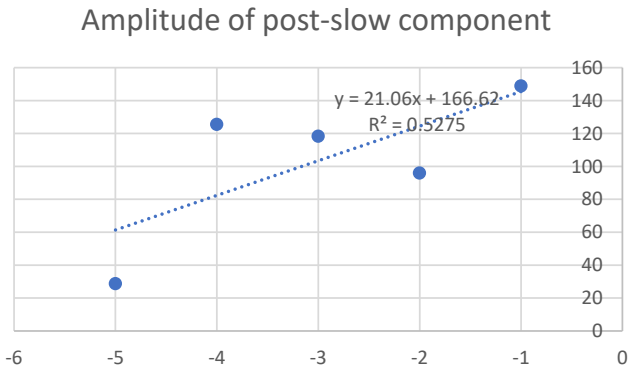

Patient 7 SZ2

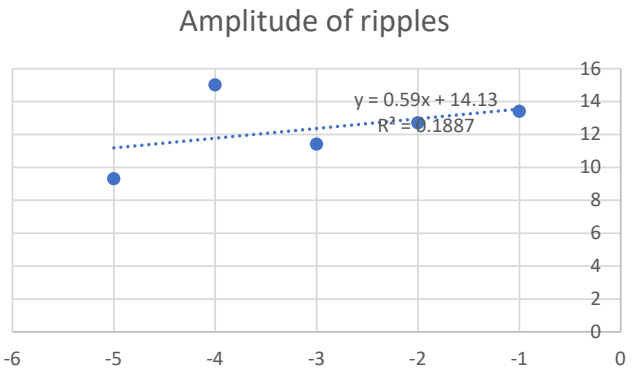

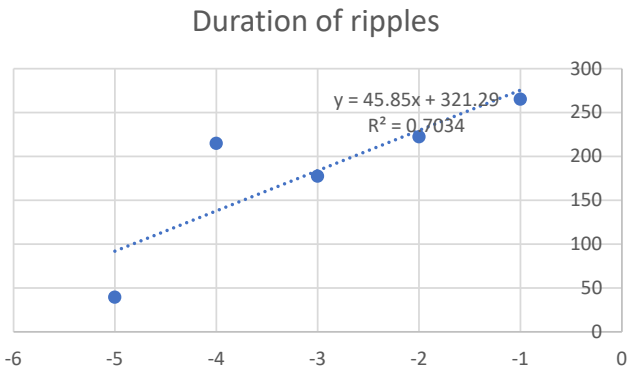

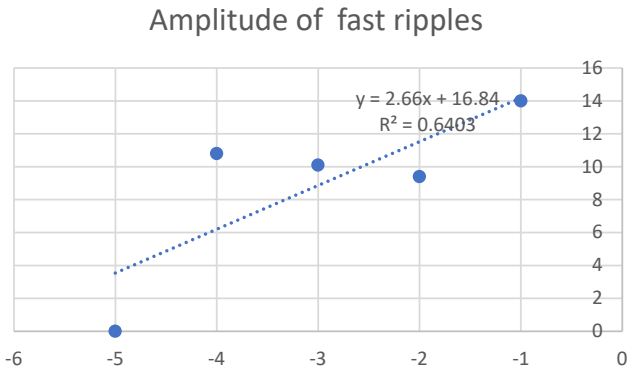

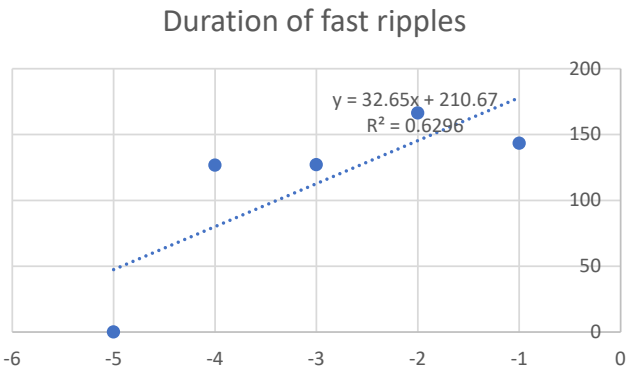

Patient 8 SZ1

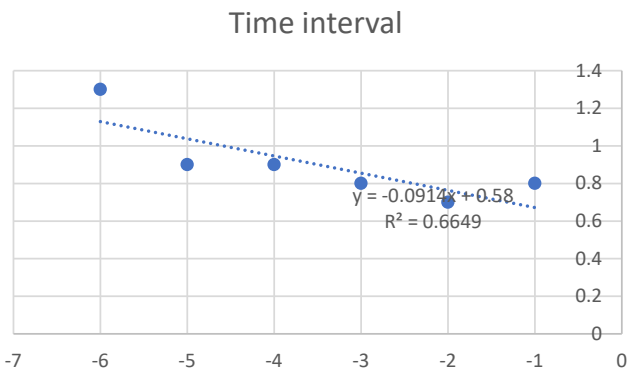

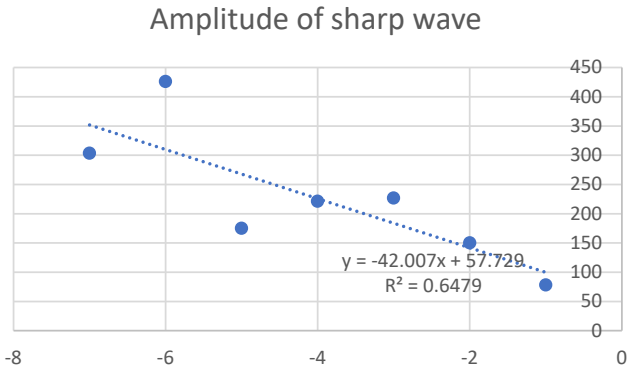

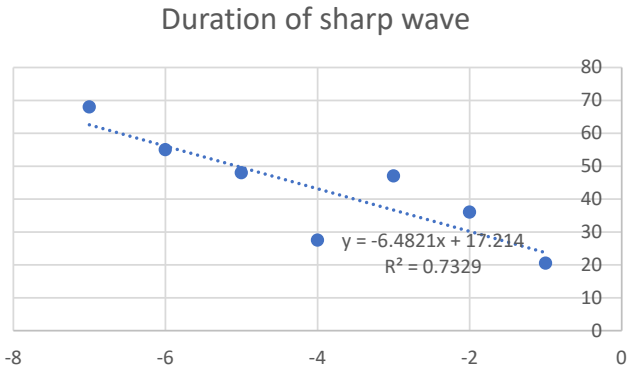

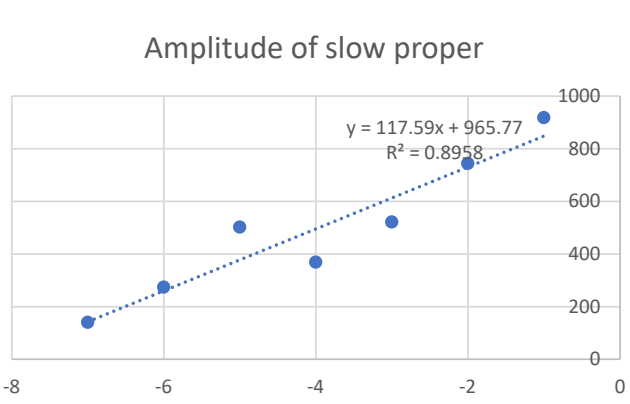

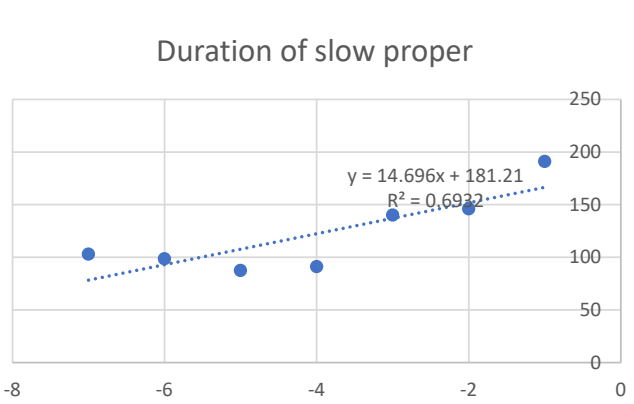

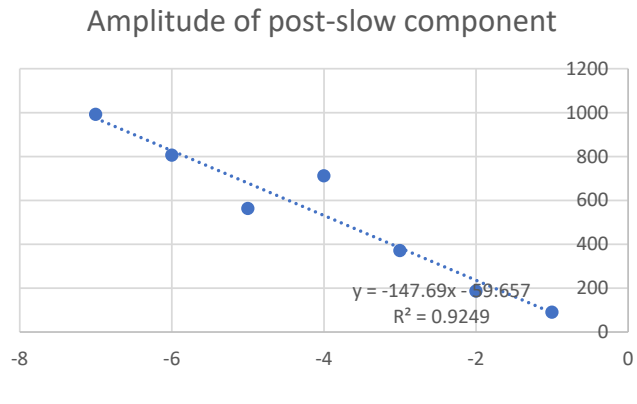

Patient 8 SZ1

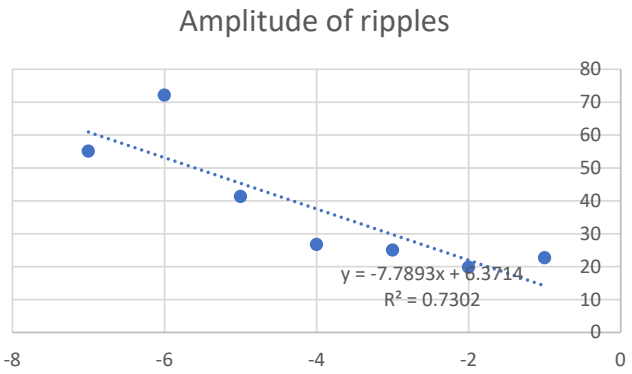

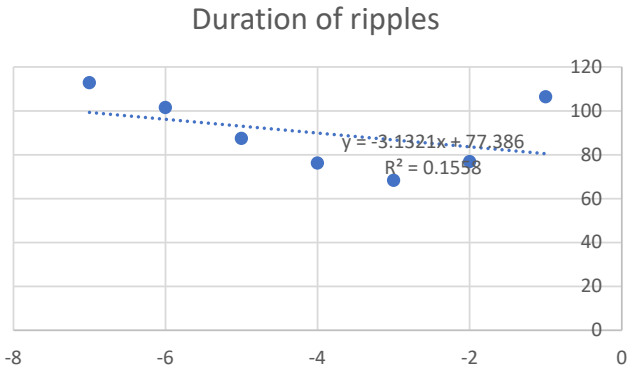

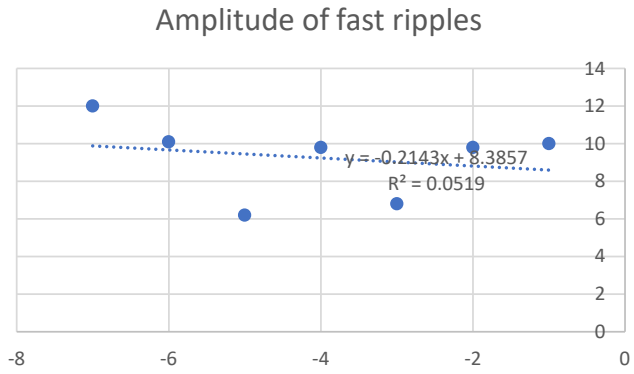

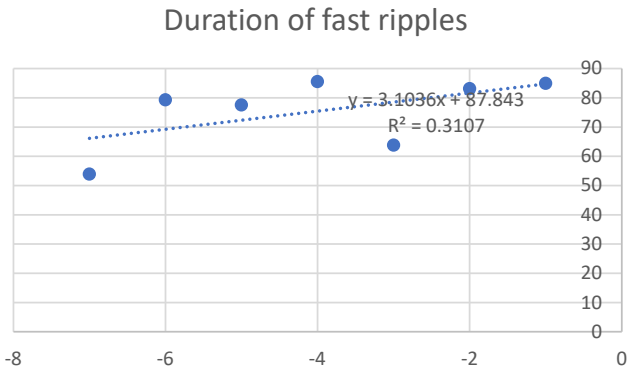

Patient 8 SZ2

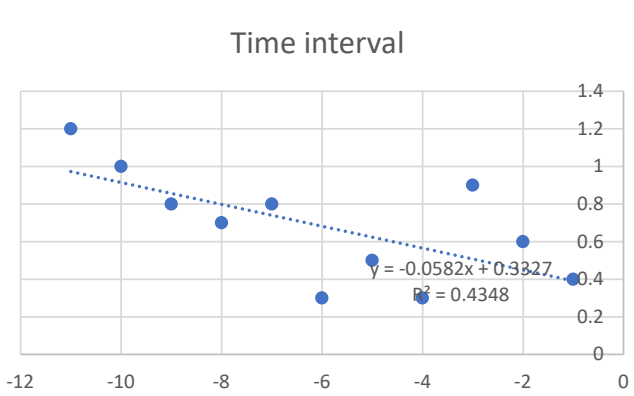

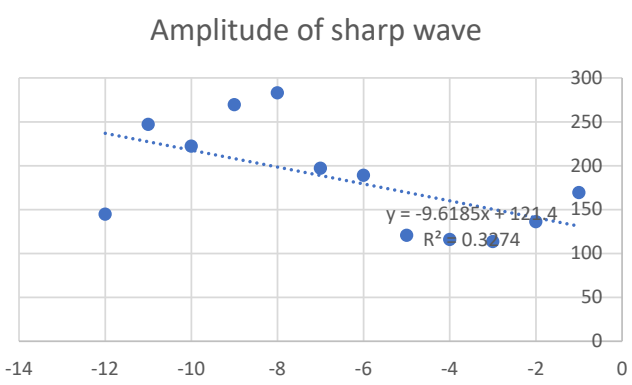

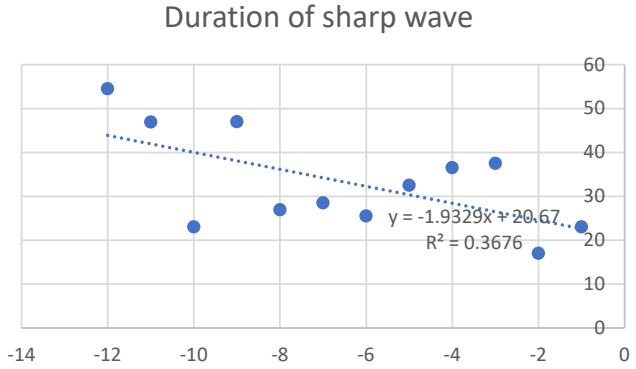

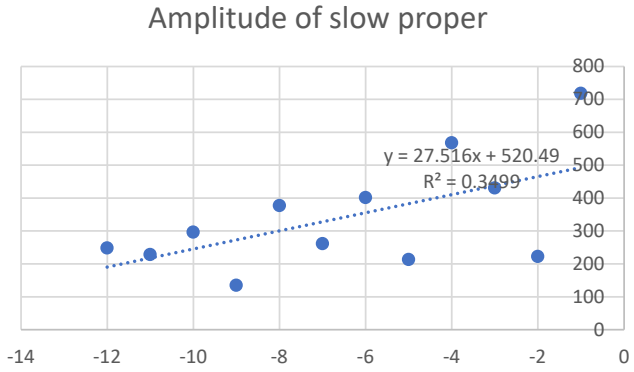

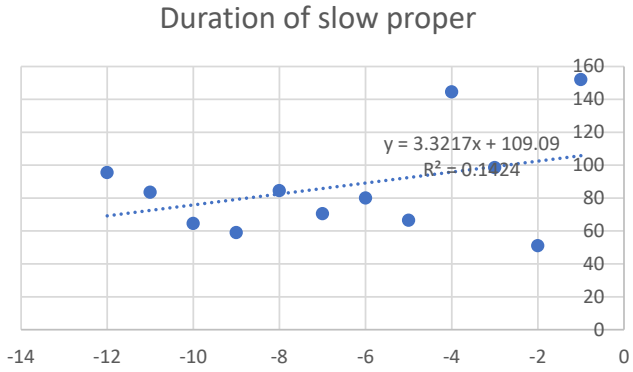

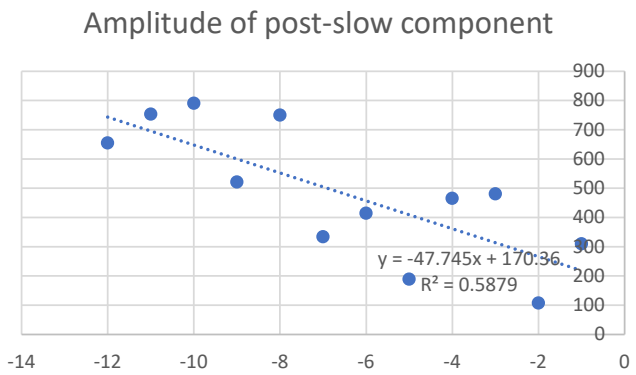

Patient 8 SZ2

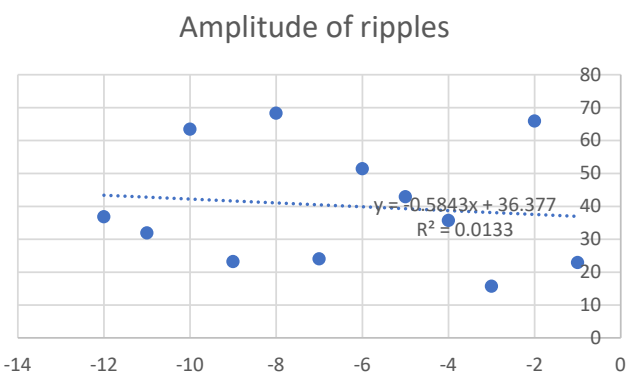

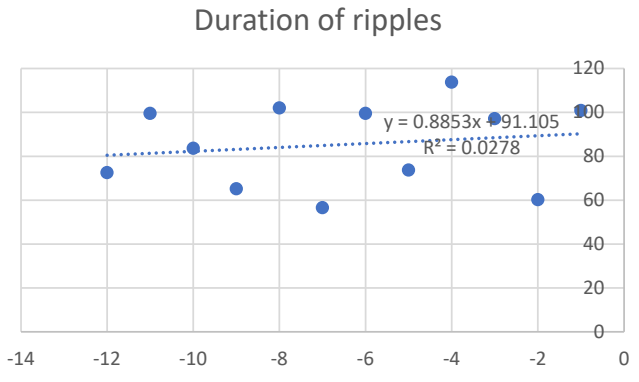

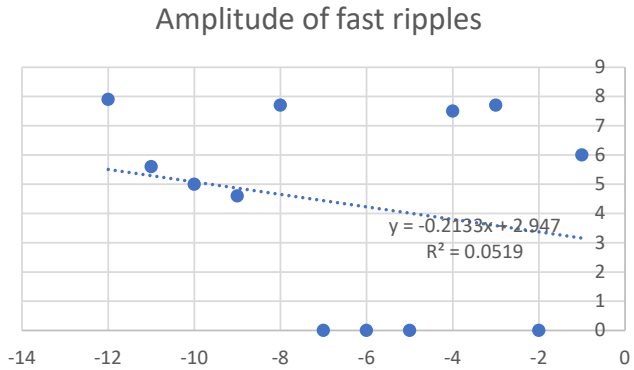

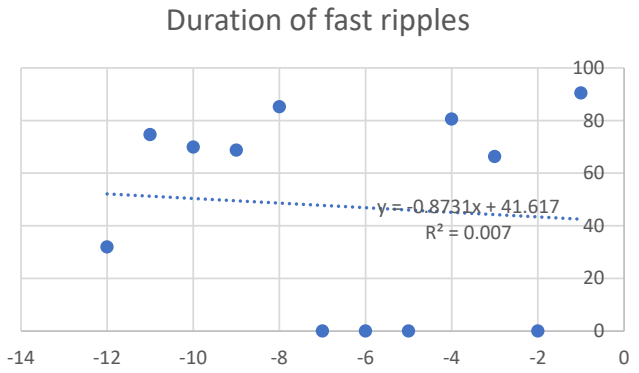

Patient 8 SZ3

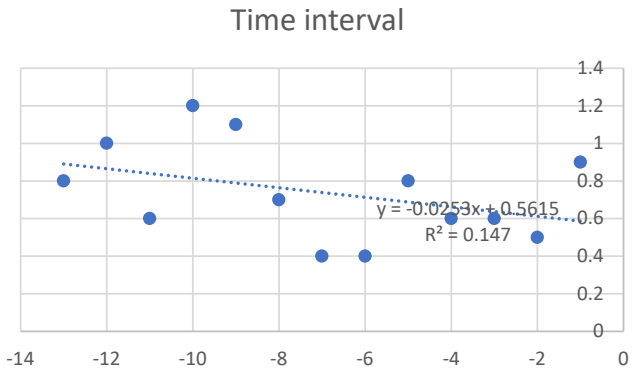

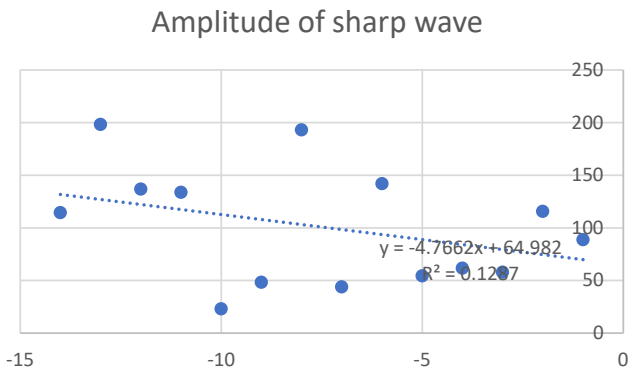

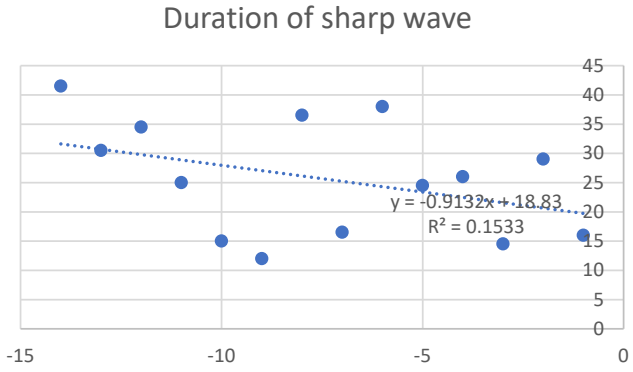

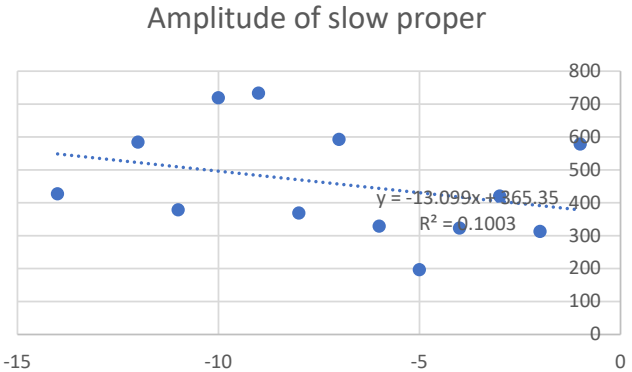

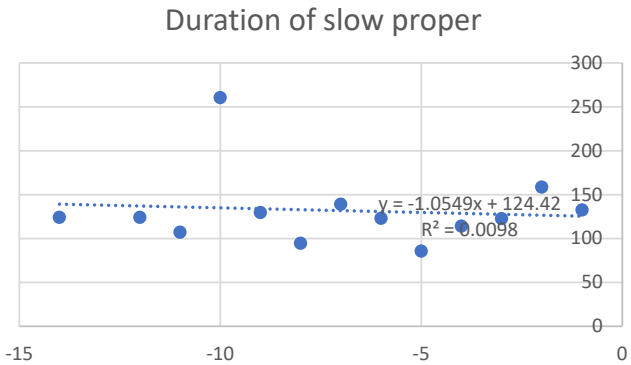

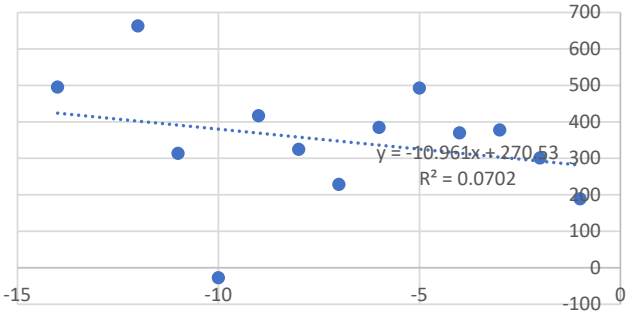

Patient 8 SZ3

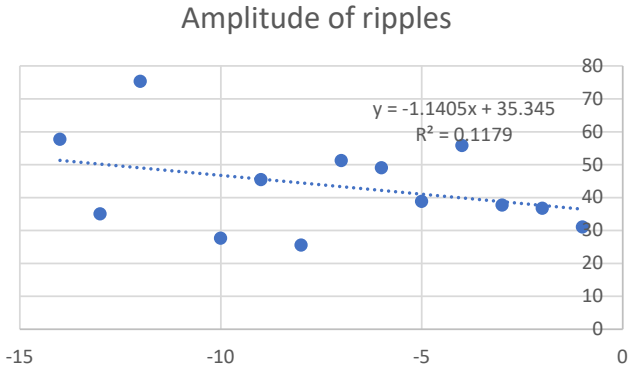

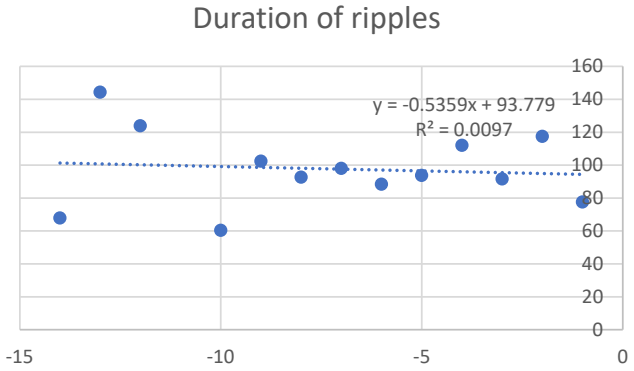

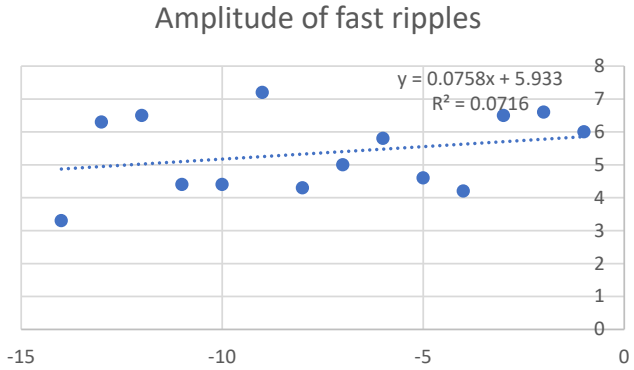

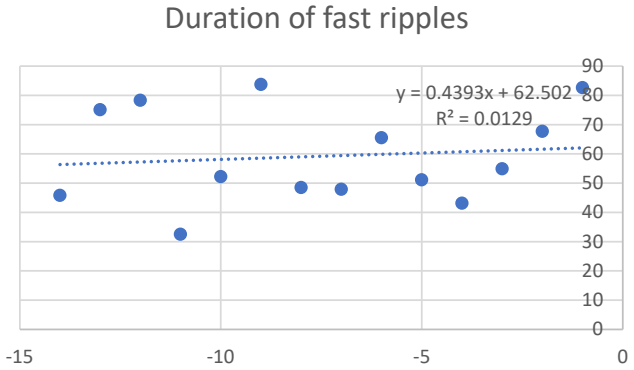

Patient 9 SZ1

Time interval

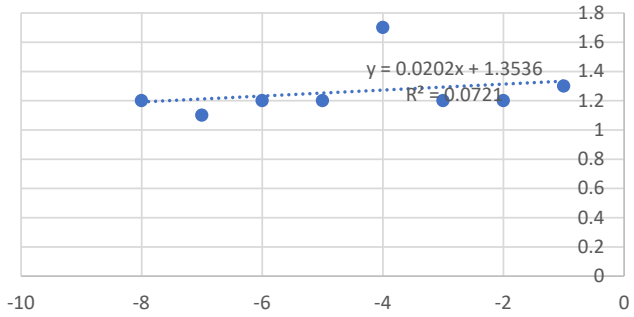

Amplitude of sharp wave

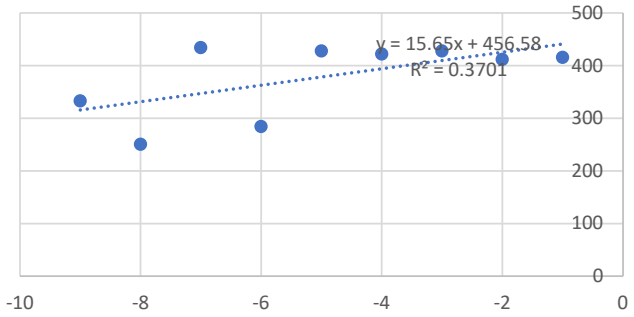

Duration of sharp wave

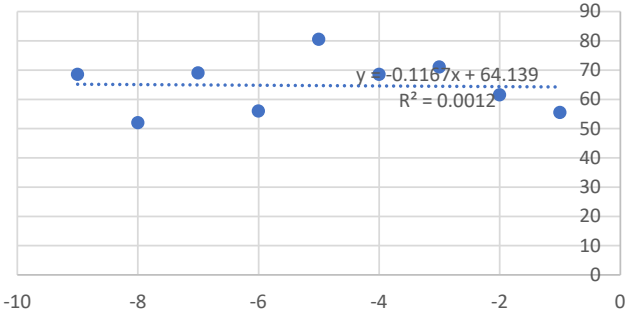

Amplitude of slow proper

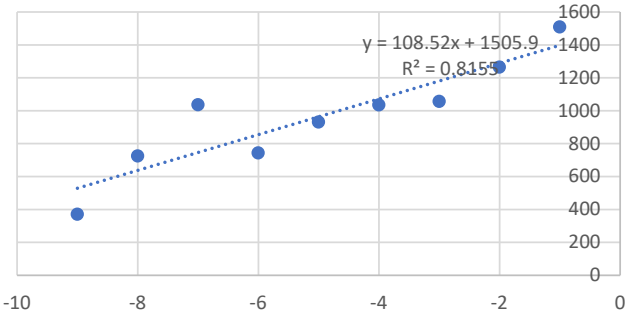

Duration of slow proper

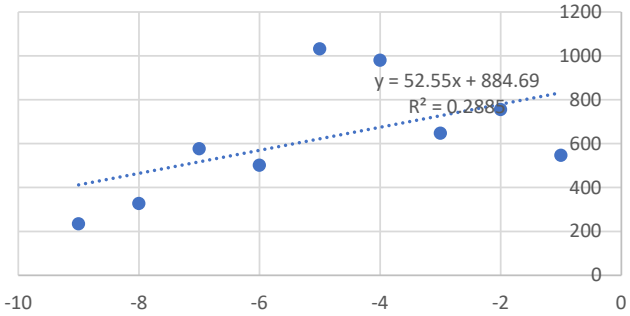

Amplitude of post-slow component

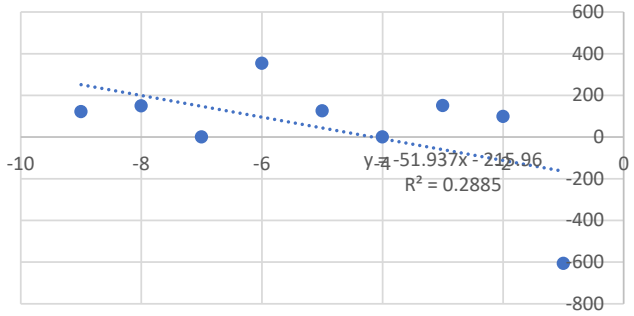

Patient 9 SZ1

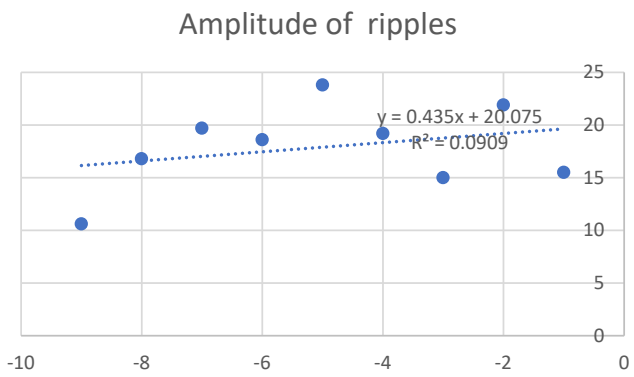

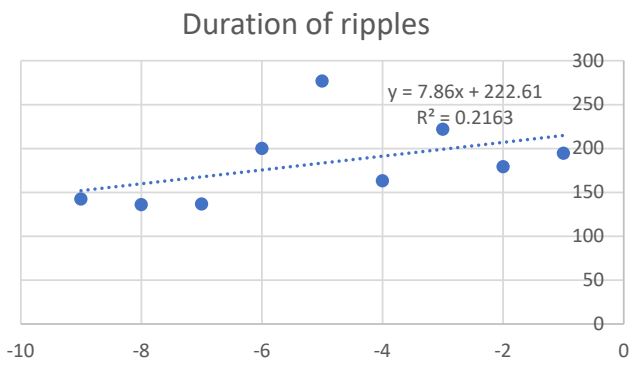

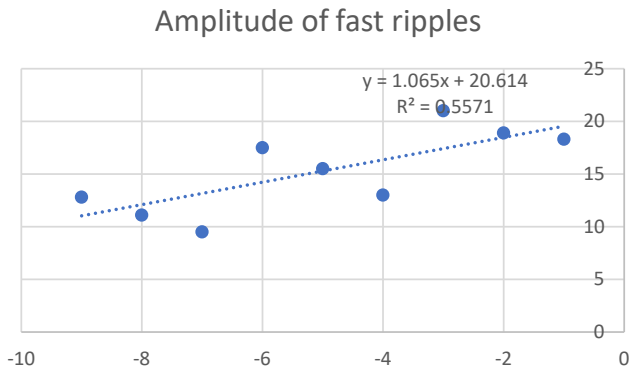

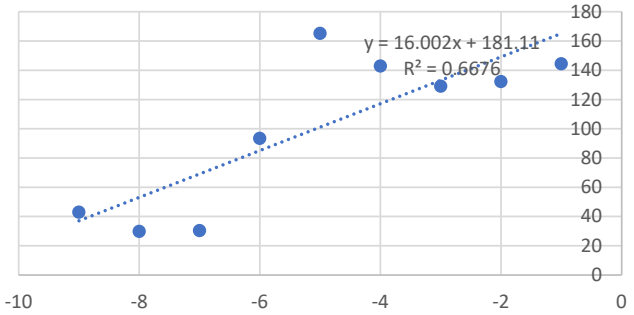

Patient 9 SZ2

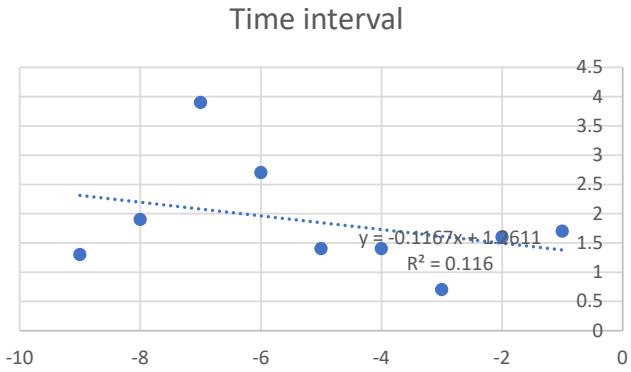

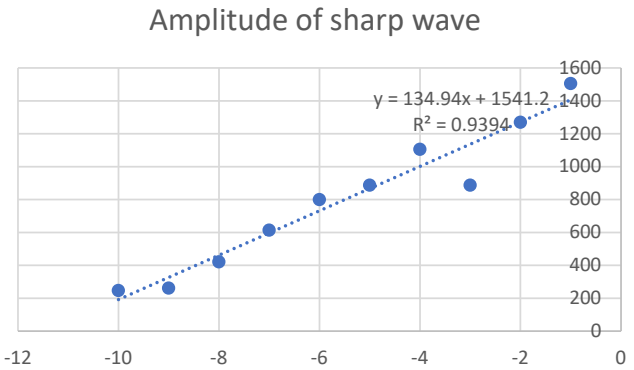

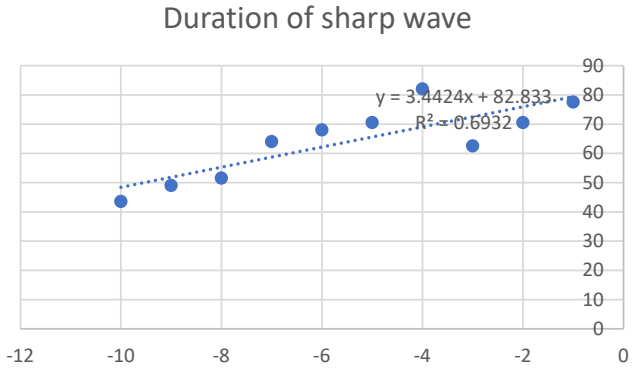

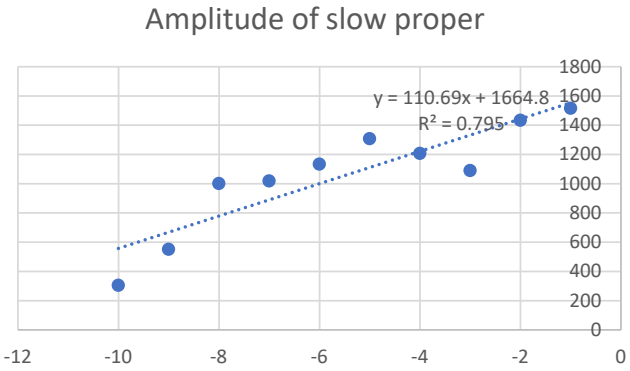

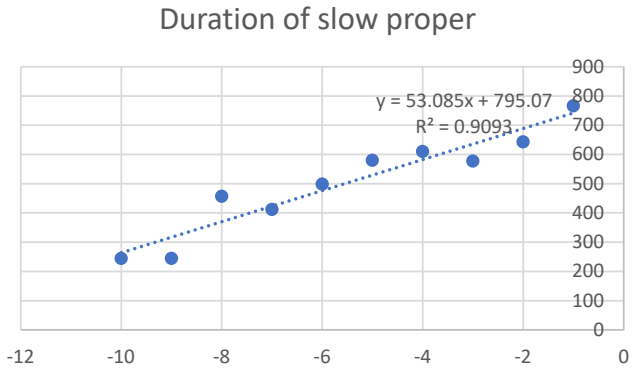

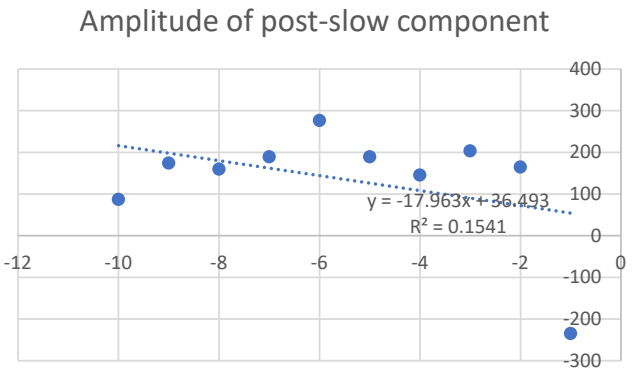

Patient 9 SZ2

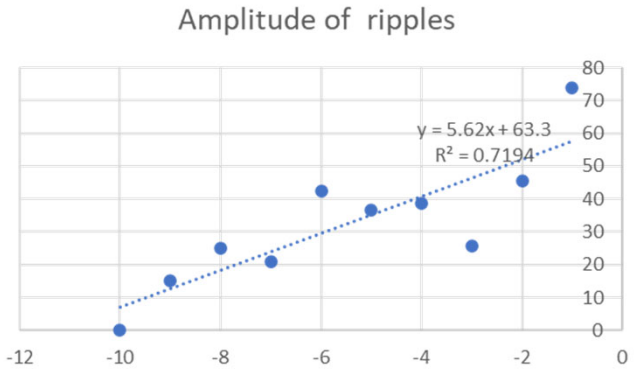

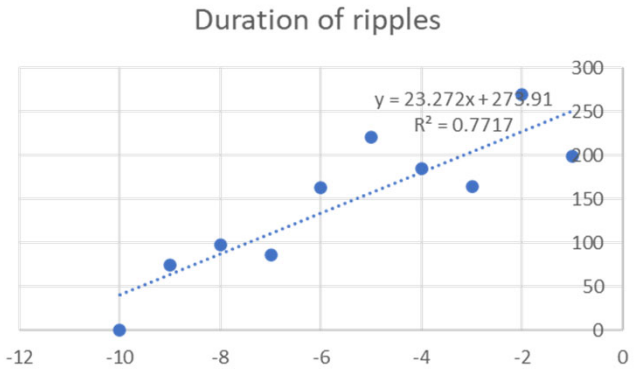

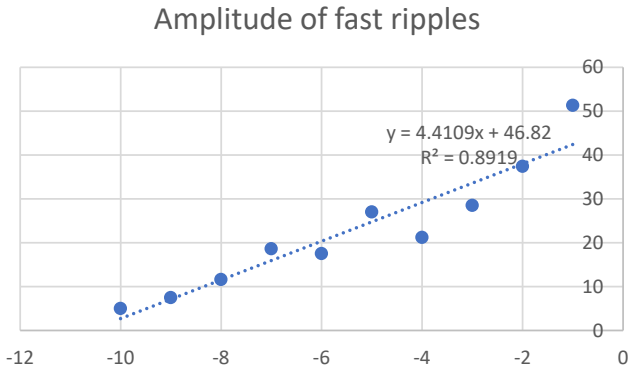

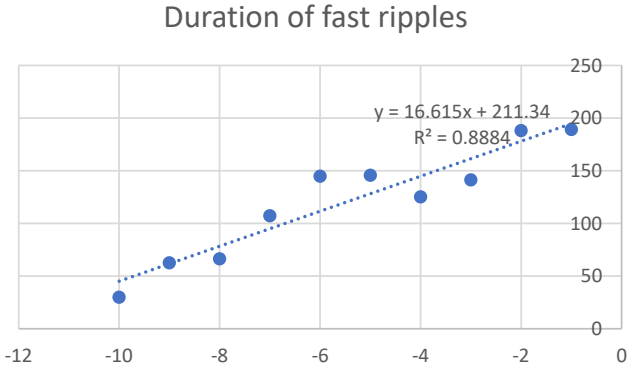

Patient 9 SZ3

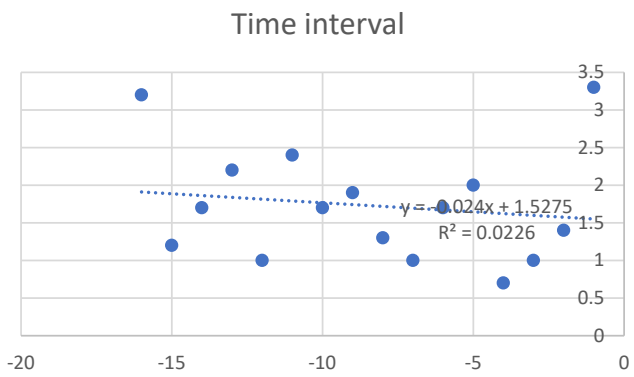

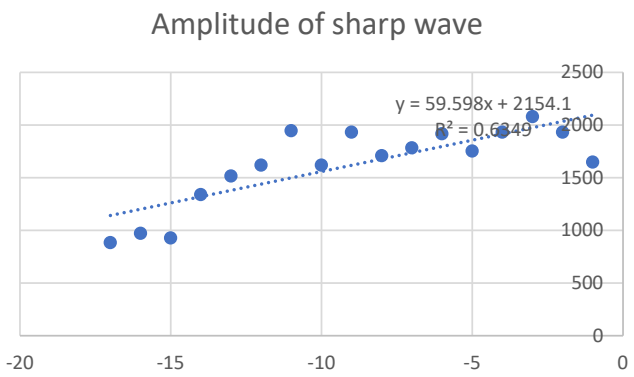

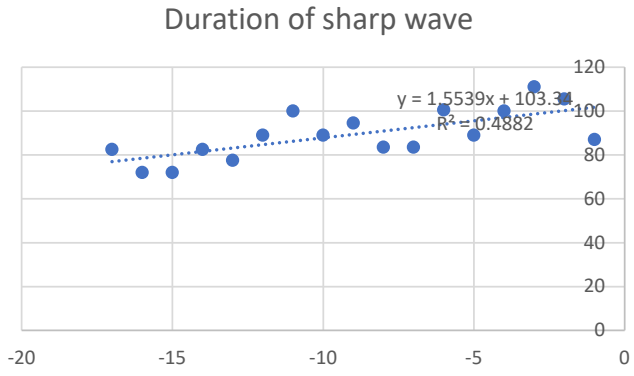

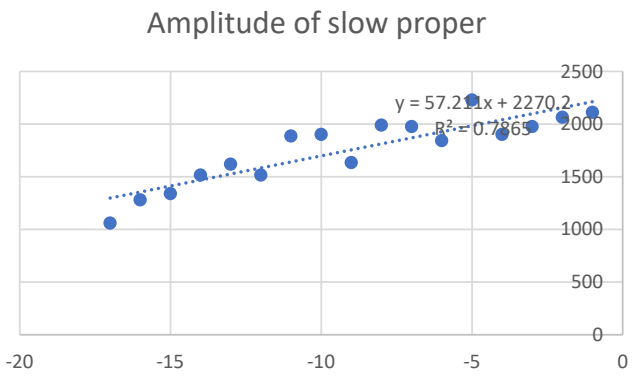

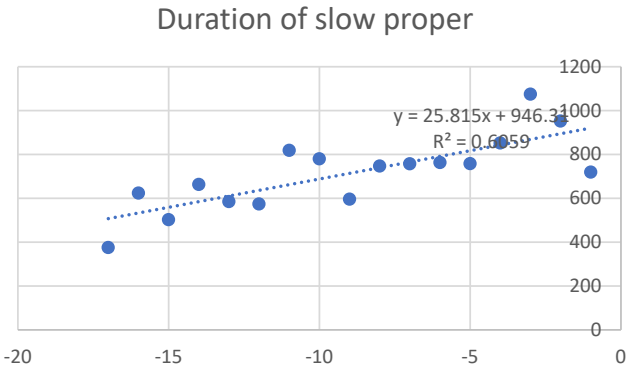

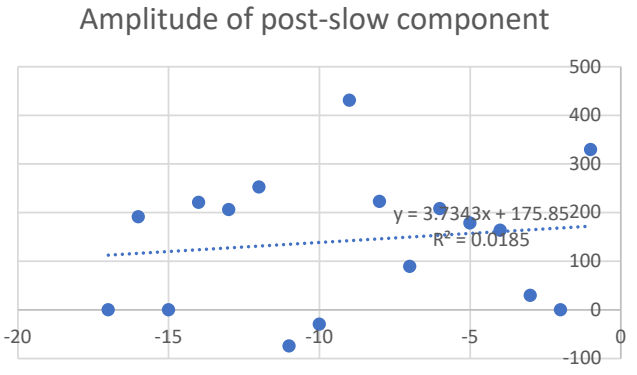

Patient 9 SZ3

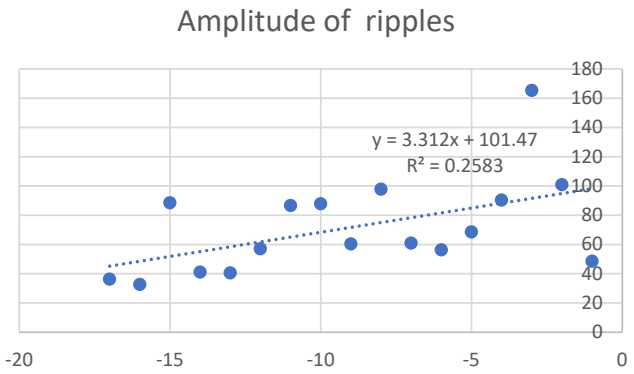

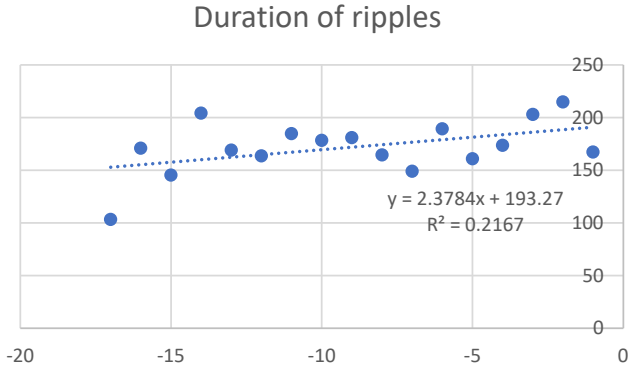

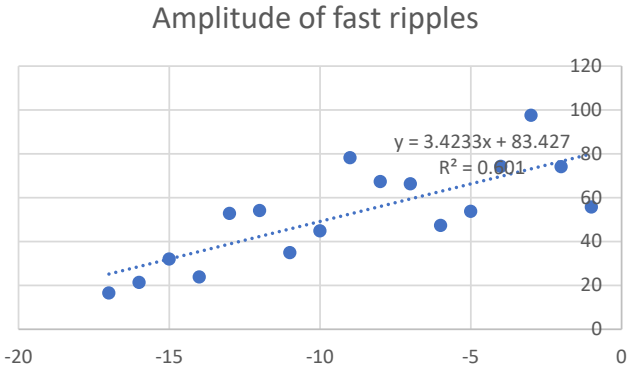

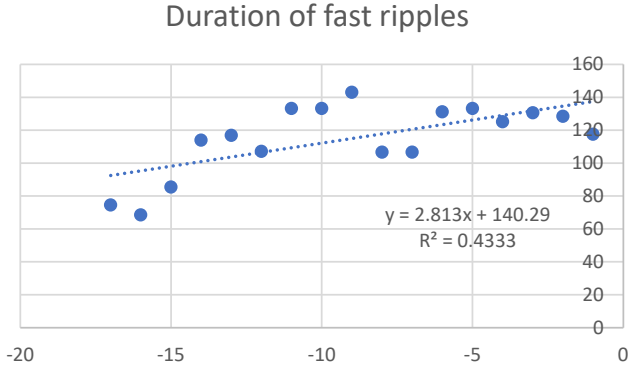

Patient 9 SZ4

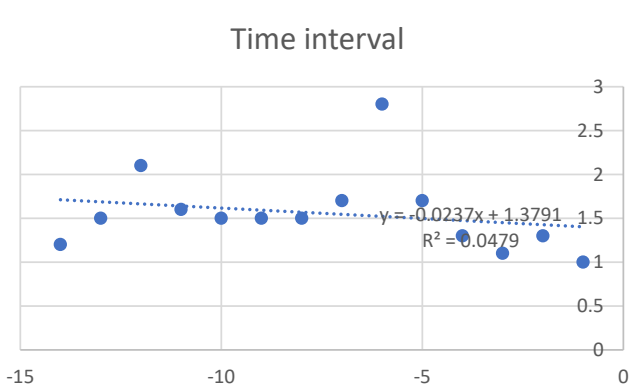

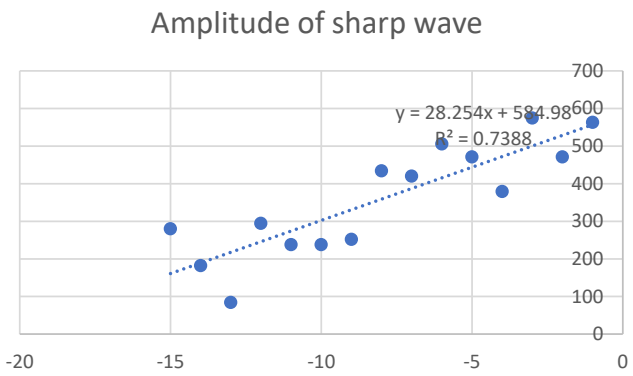

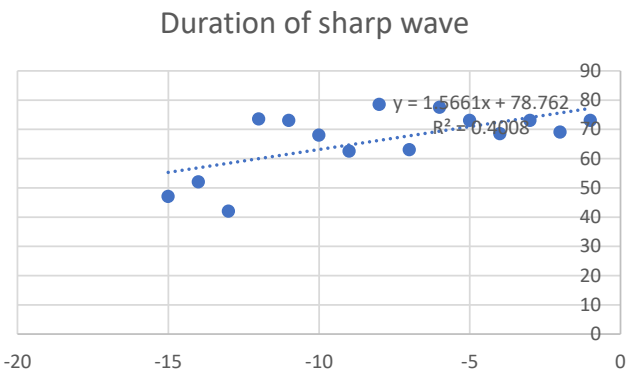

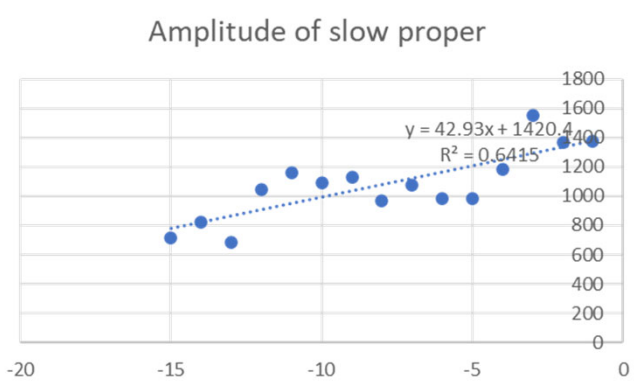

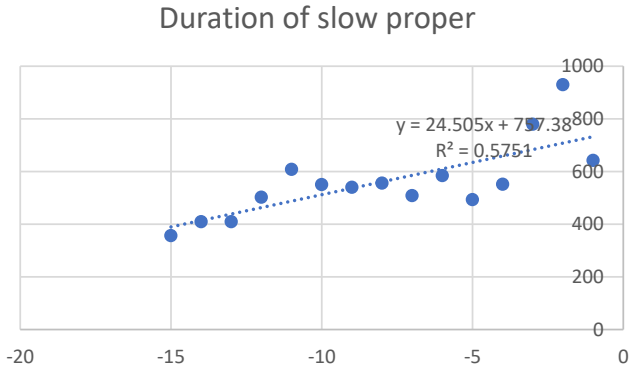

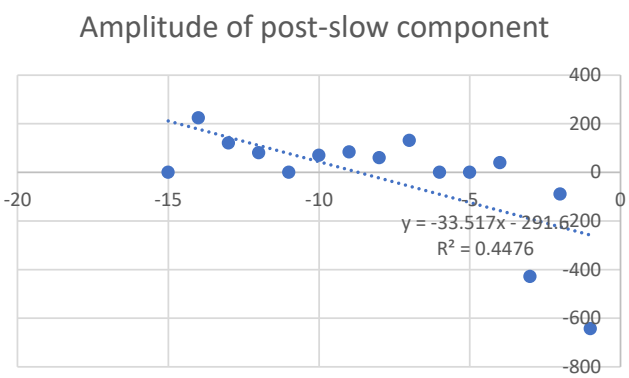

Patient 9 SZ4

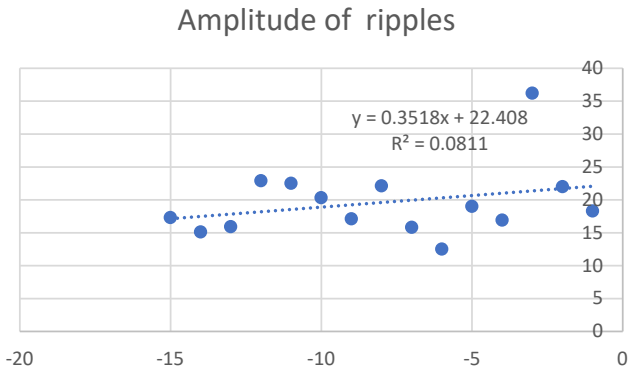

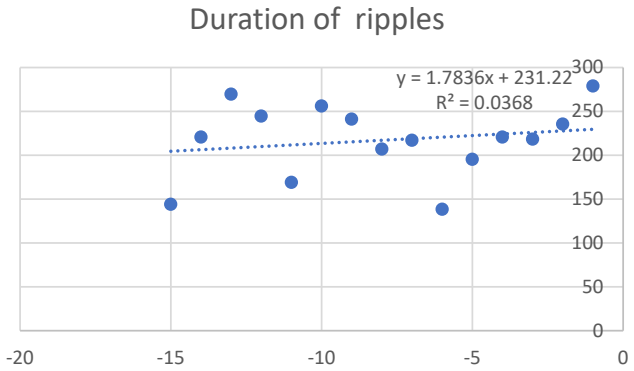

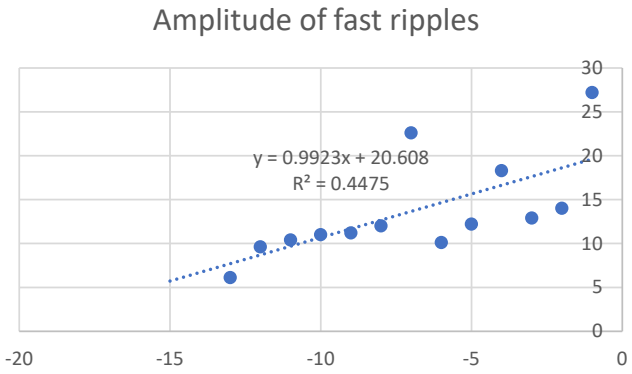

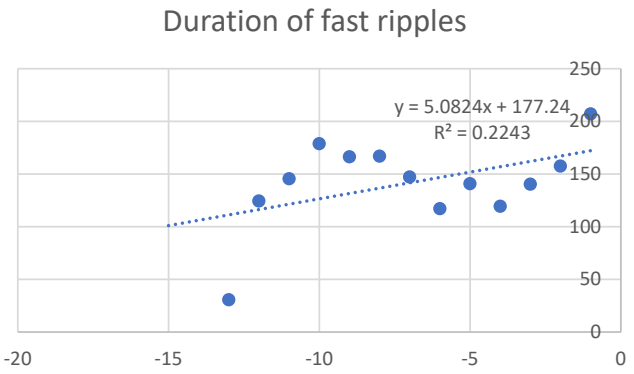

Patient 9 SZ5

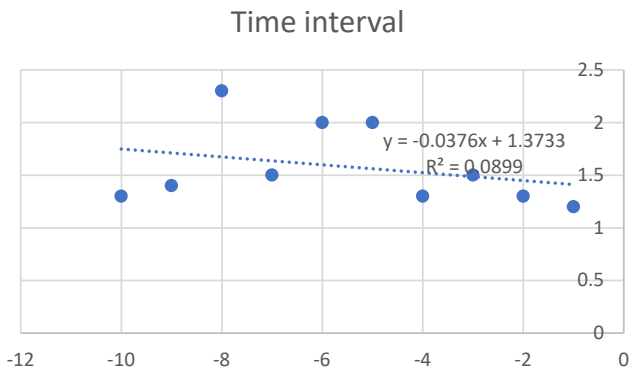

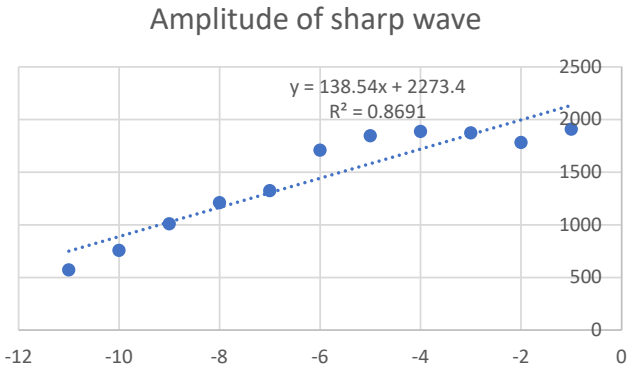

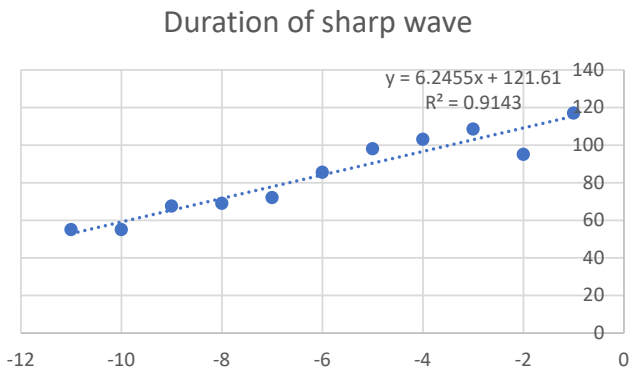

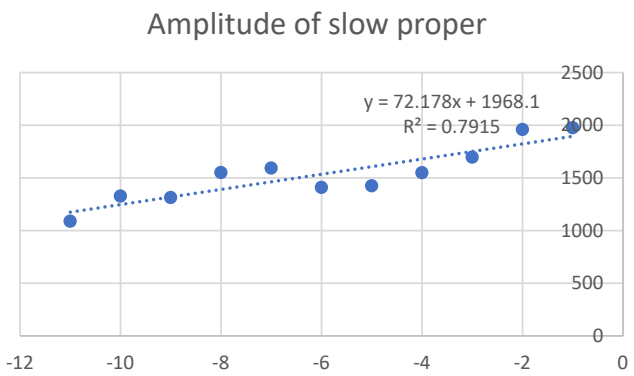

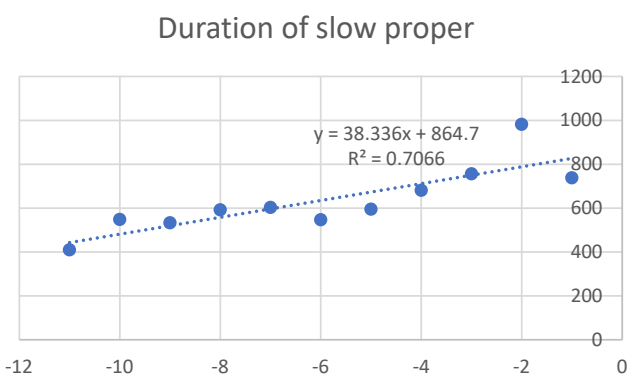

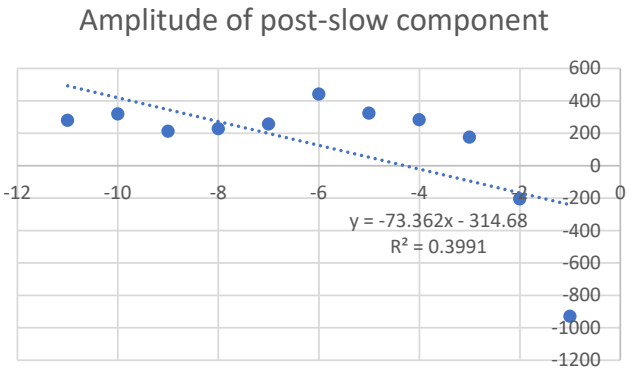

Patient 9 SZ5

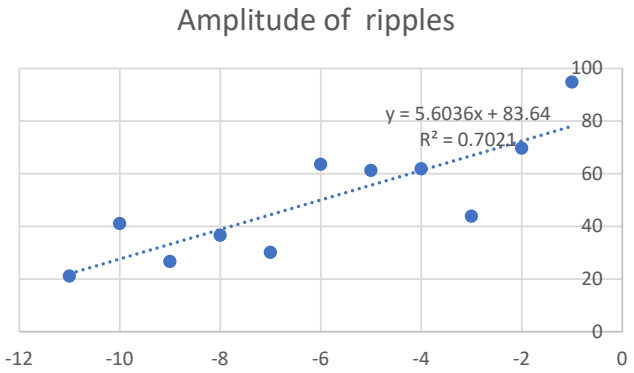

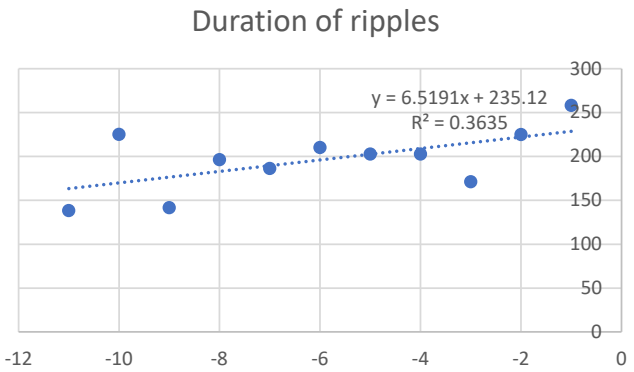

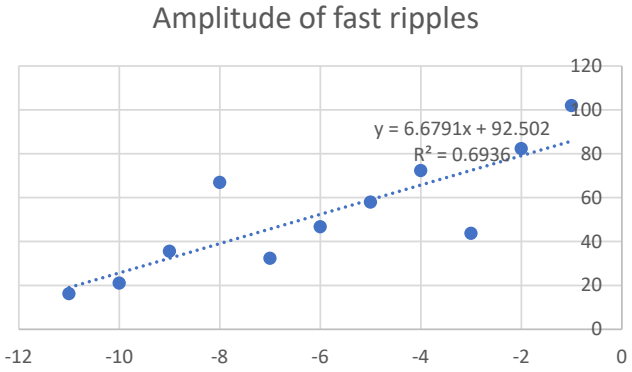

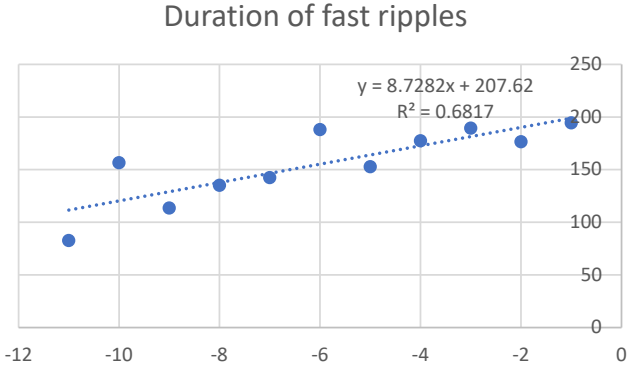

Patient 10 SZ1

Time interval

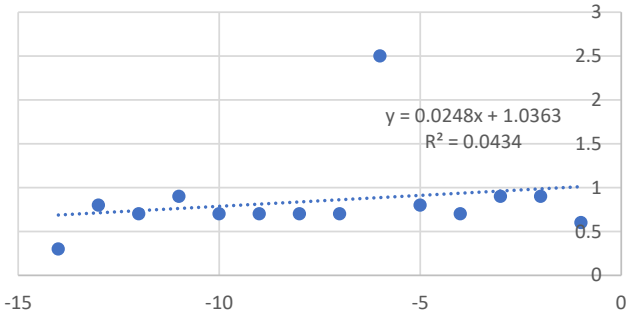

Amplitude of sharp wave

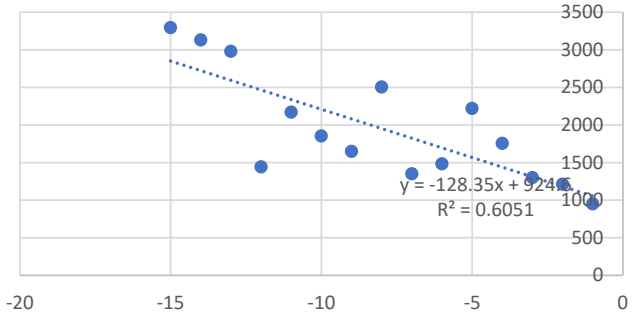

Duration of sharp wave

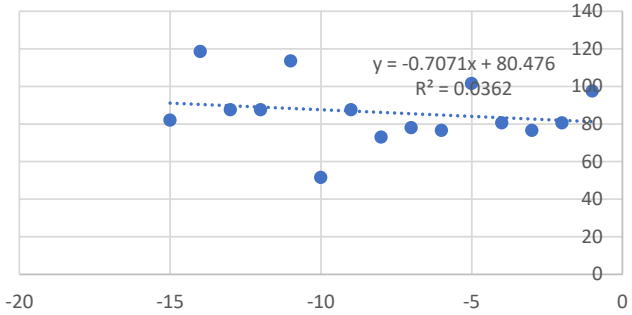

Amplitude of slow proper

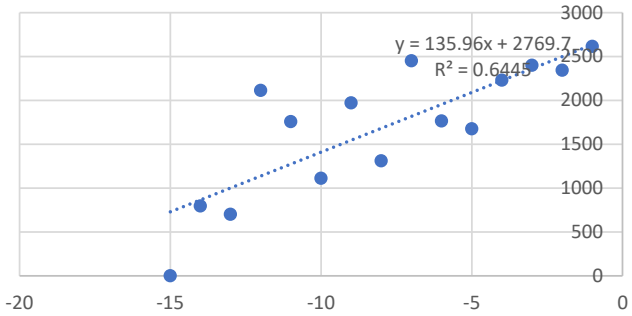

Duration of slow proper

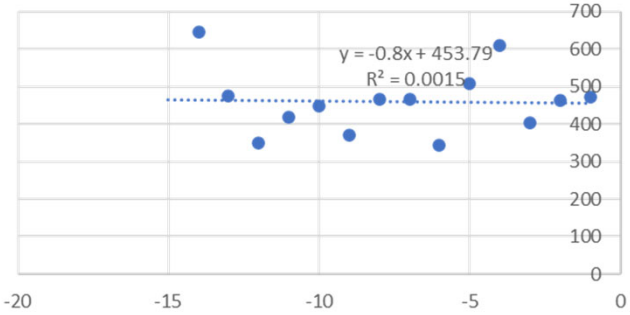

Amplitude of post-slow component

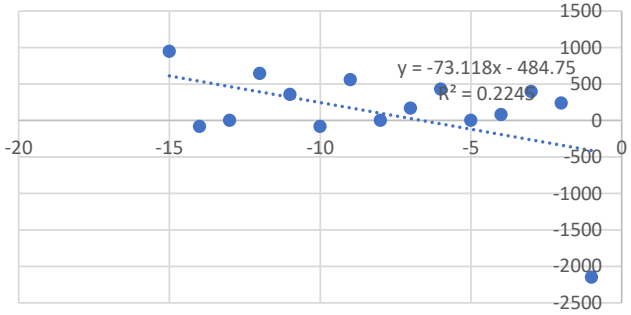

Patient 10 SZ1

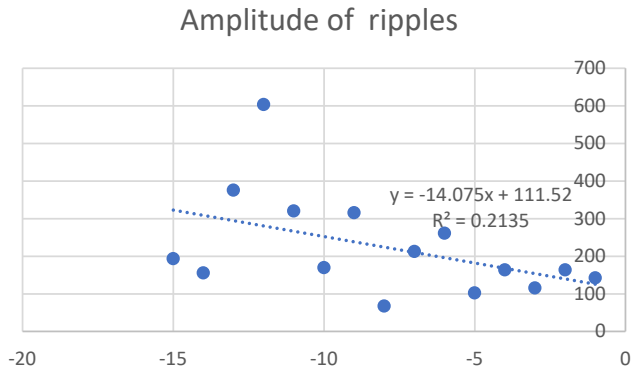

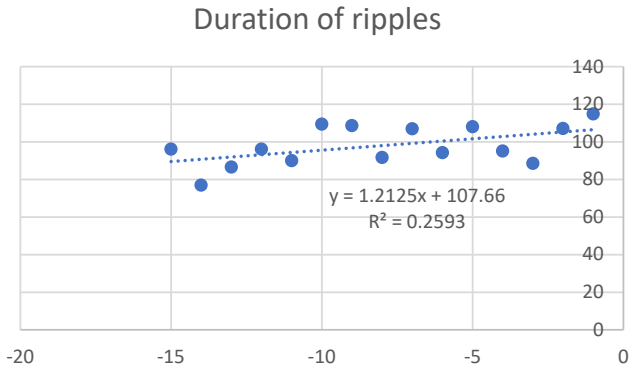

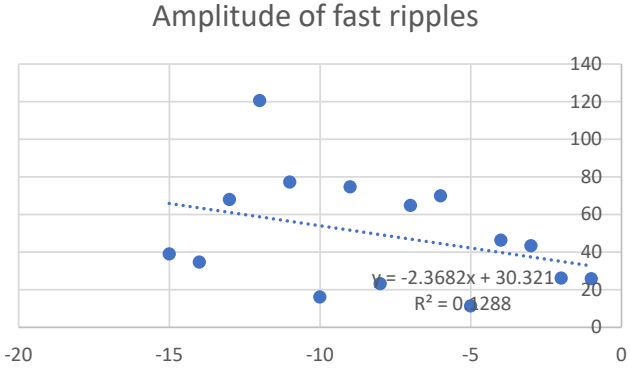

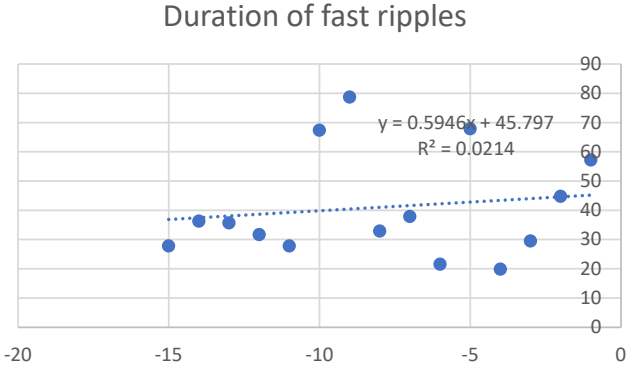

Patient 10 SZ2

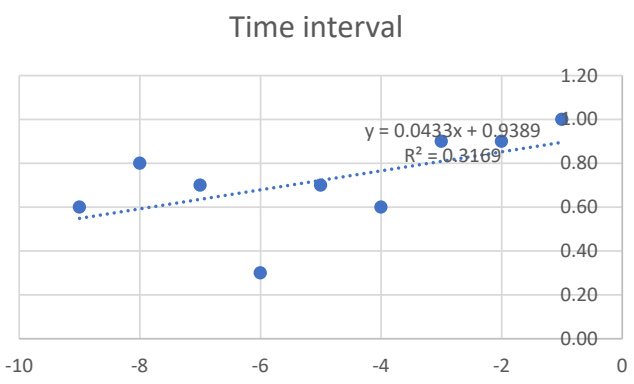

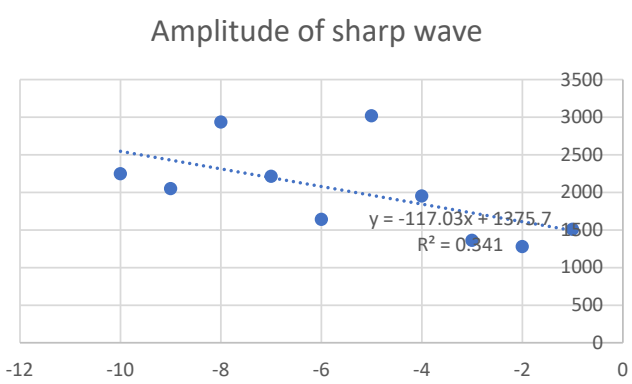

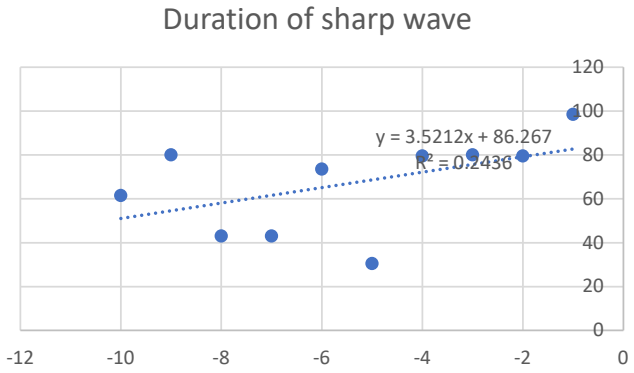

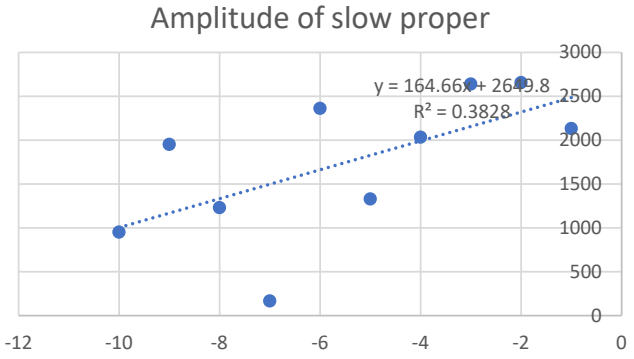

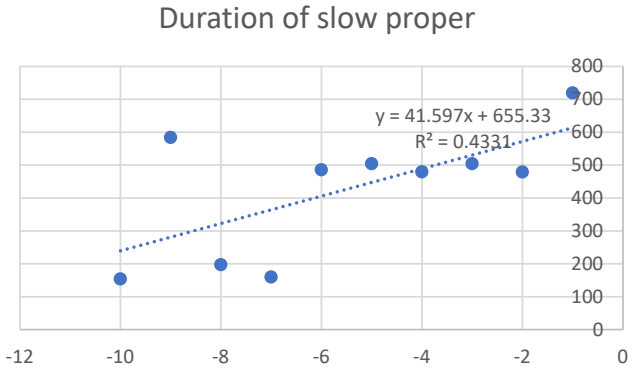

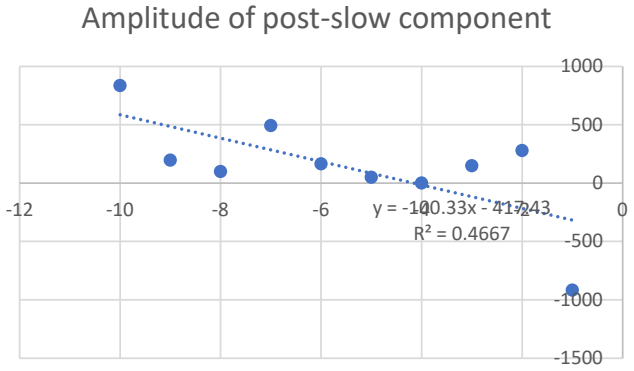

Patient 10 SZ2

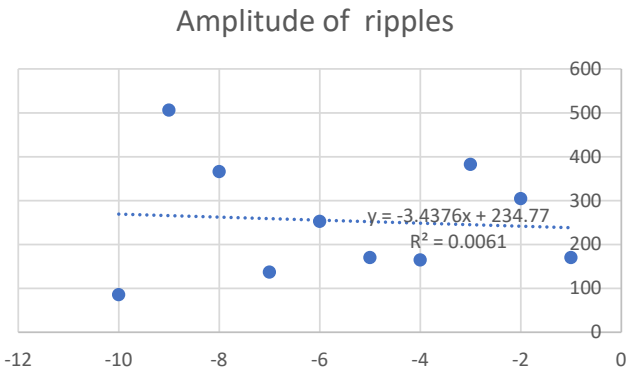

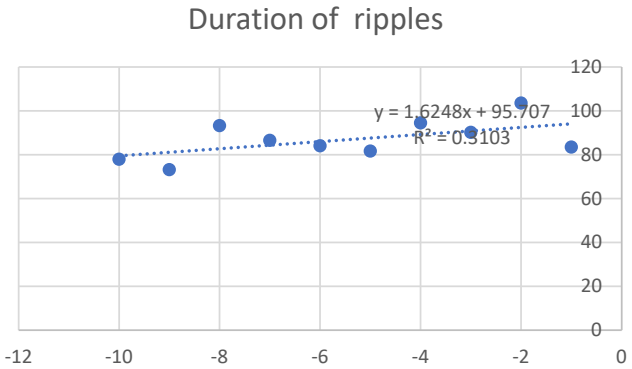

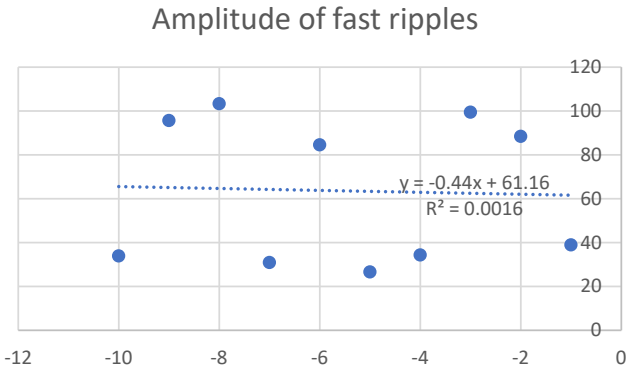

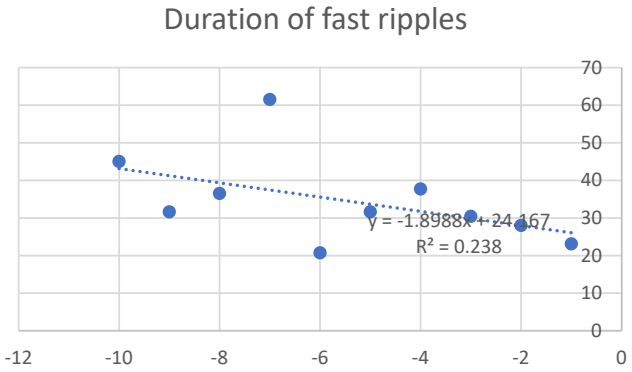

Patient 10 SZ3

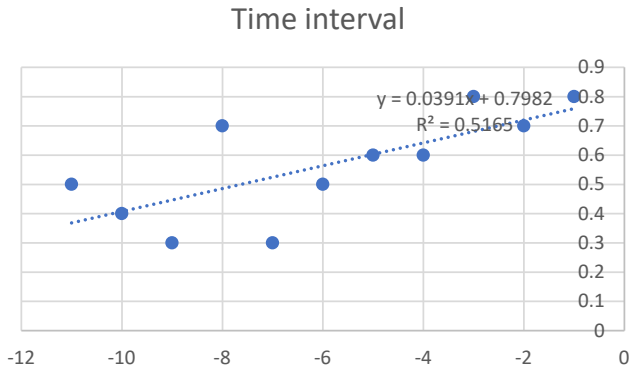

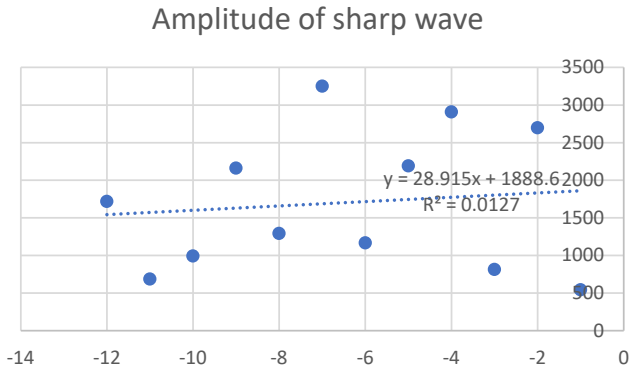

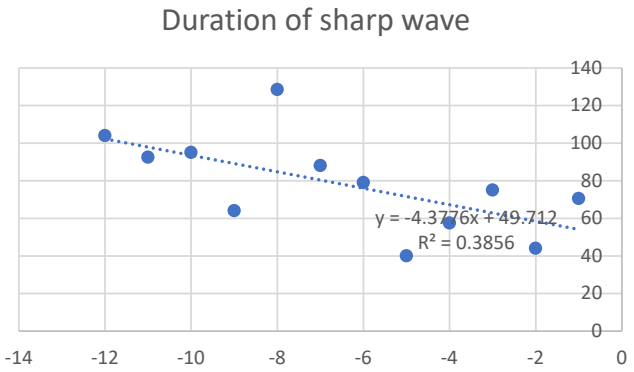

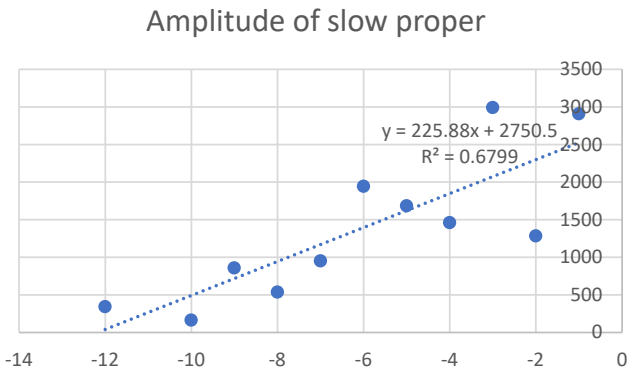

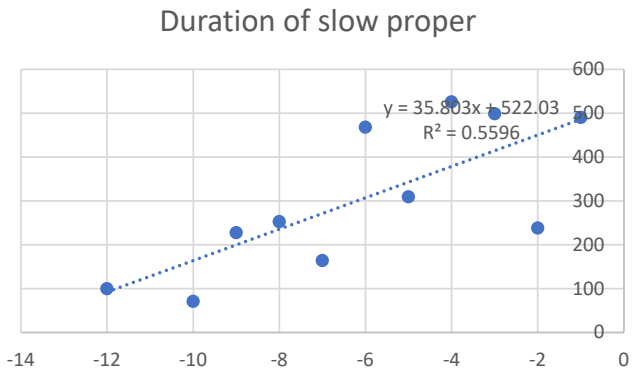

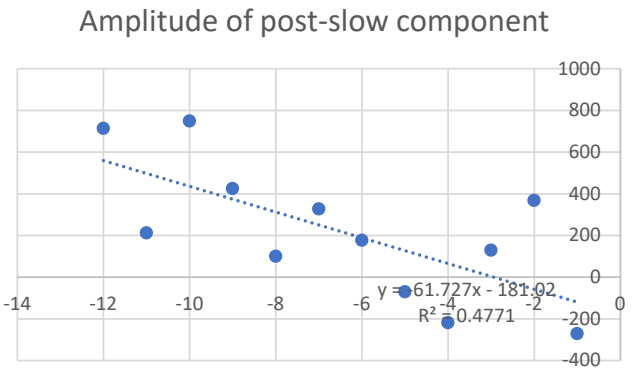

Patient 10 SZ3

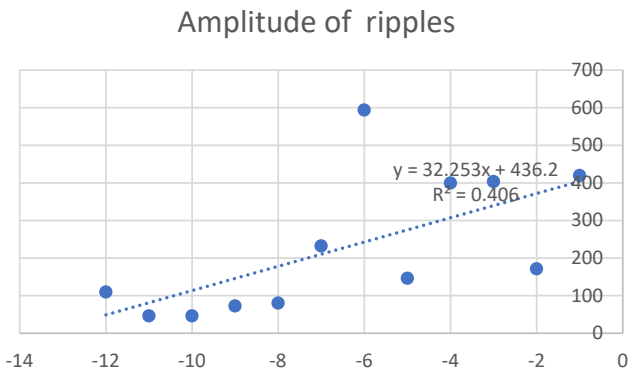

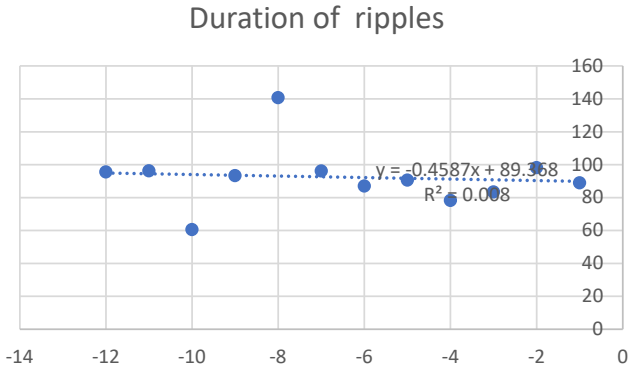

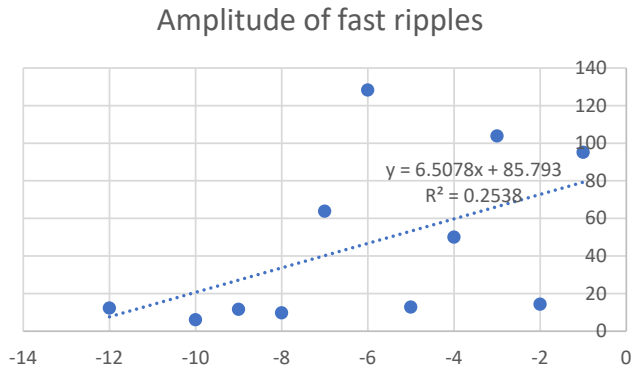

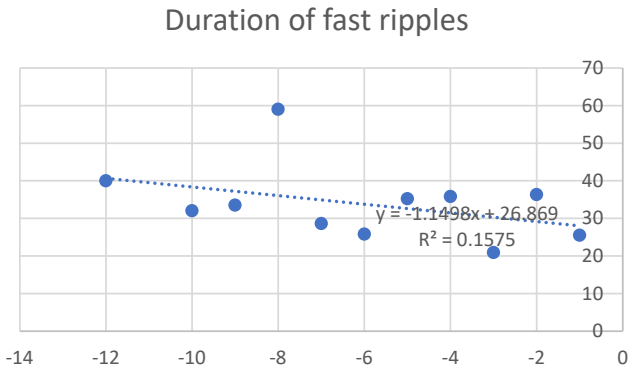

Patient 10 SZ4

Time interval

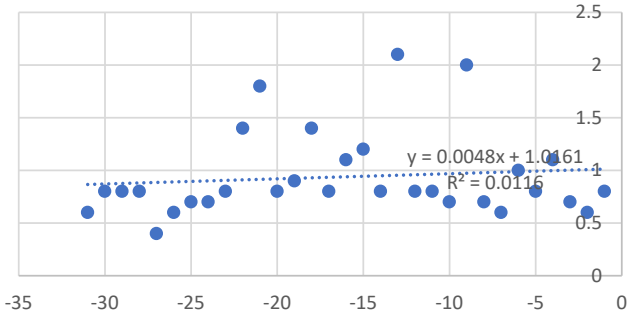

Amplitude of sharp wave

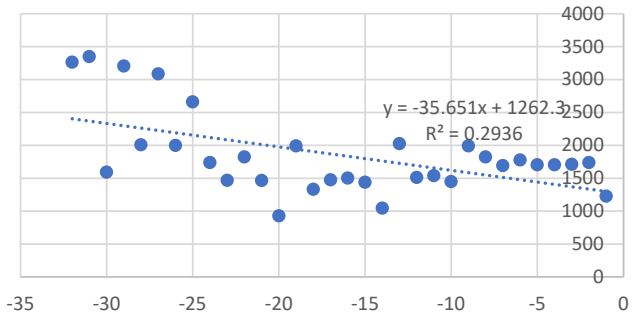

Duration of sharp wave

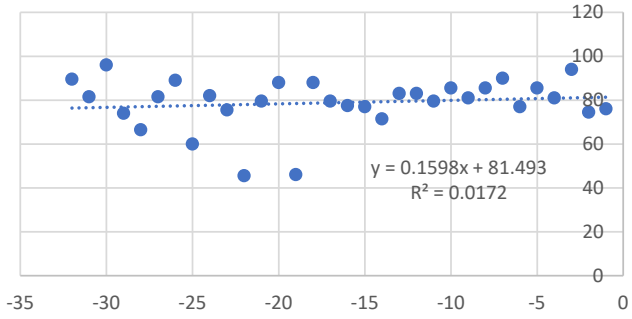

Amplitude of slow proper

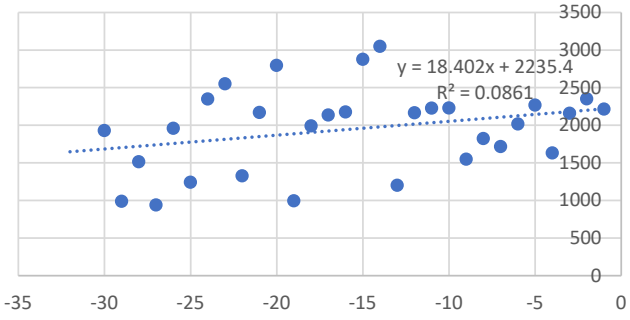

Duration of slow proper

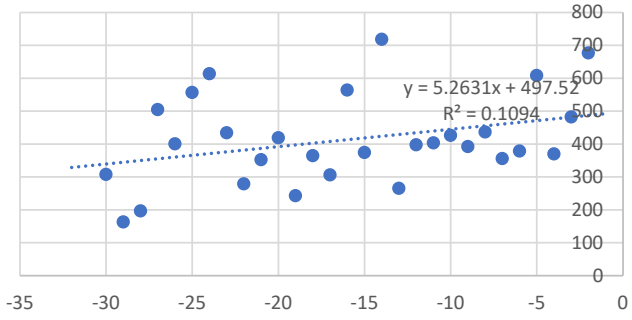

Amplitude of post-slow component

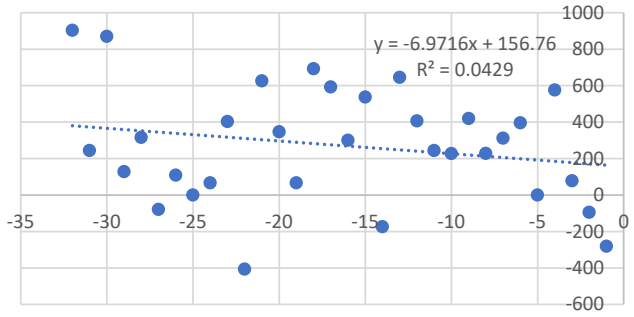

Patient 10 SZ4

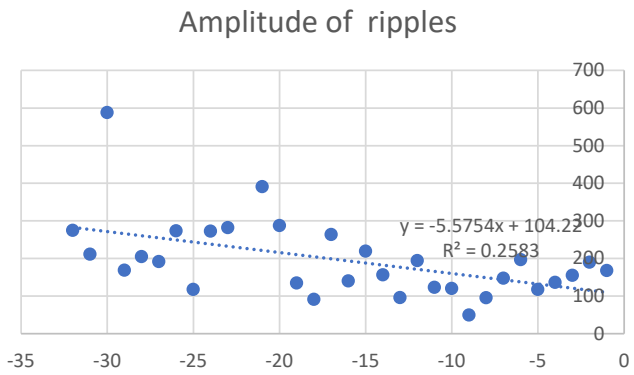

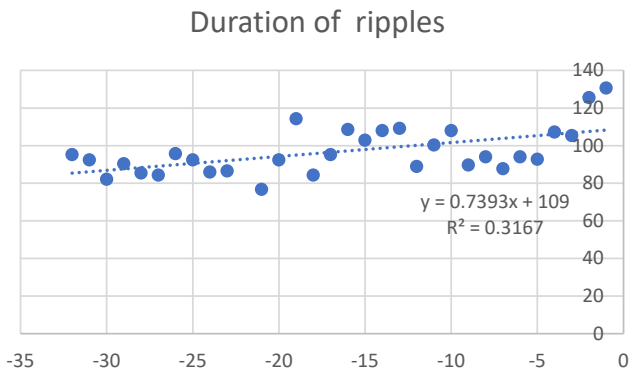

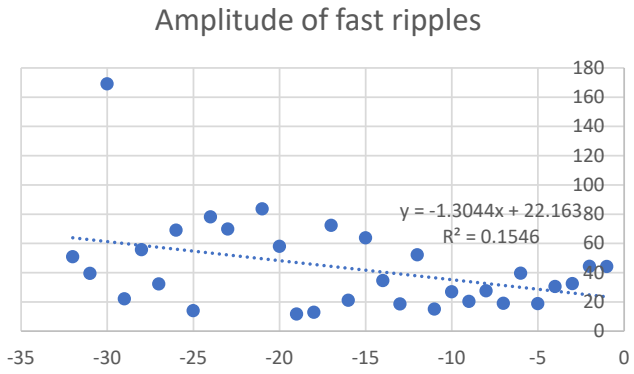

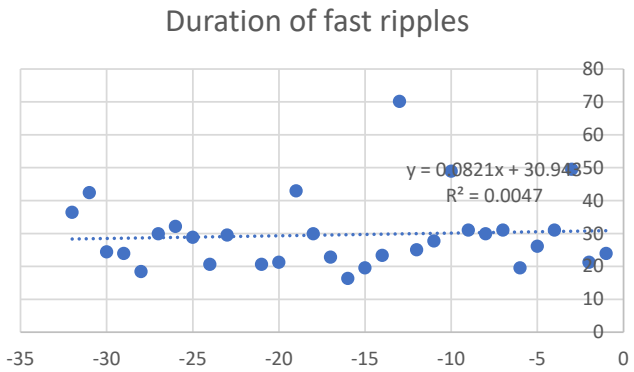

Patient 10 SZ5

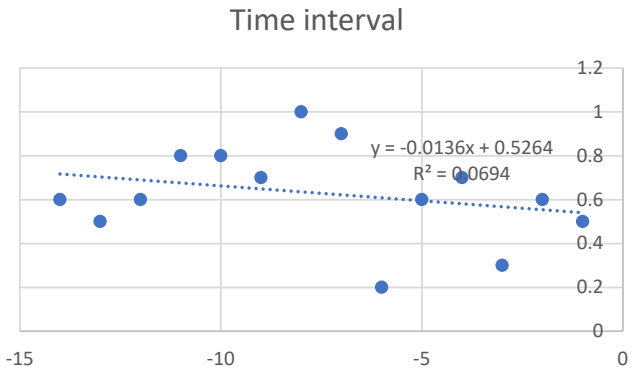

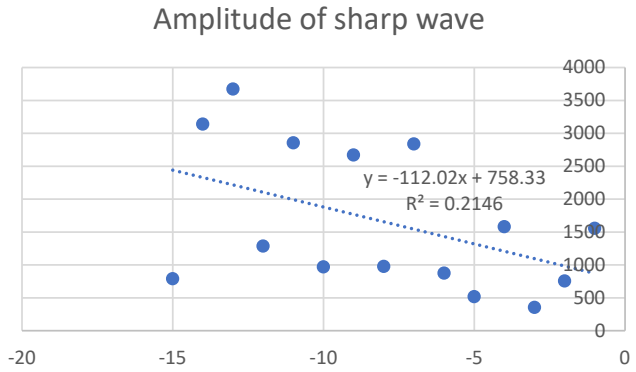

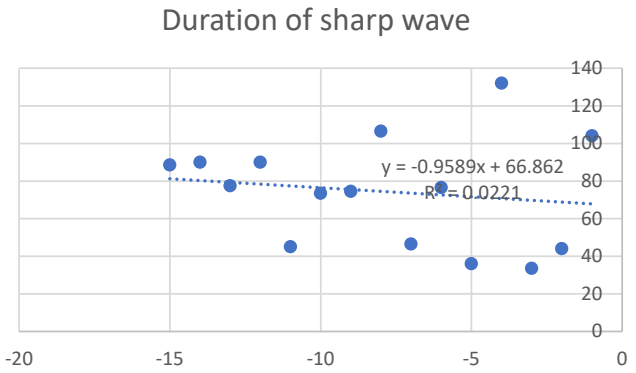

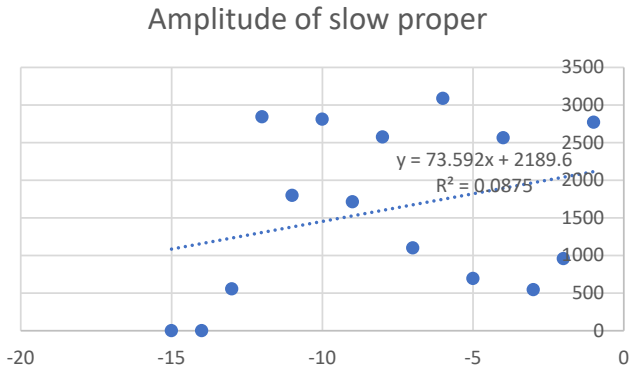

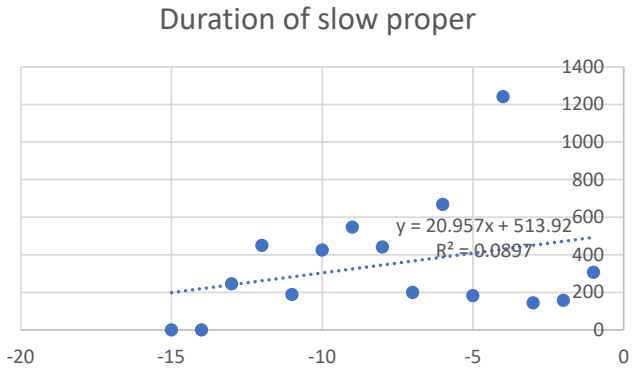

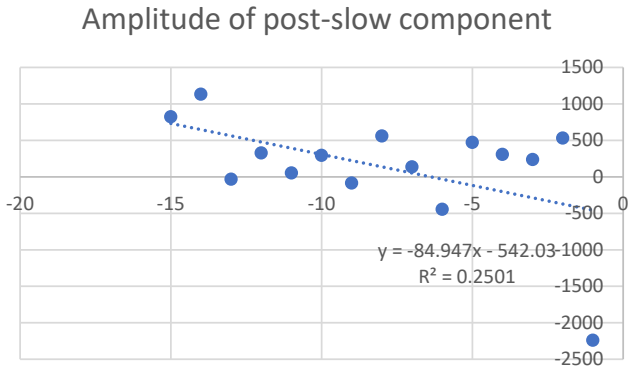

Patient 10 SZ5

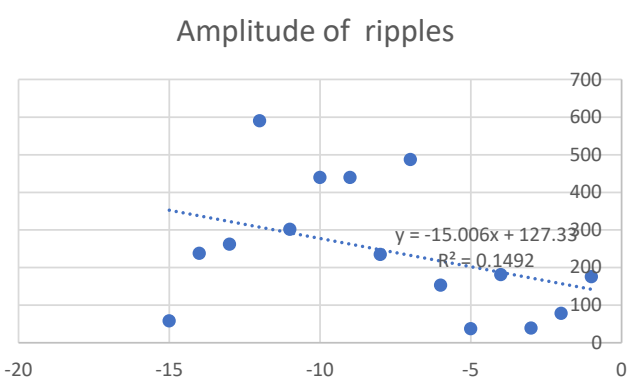

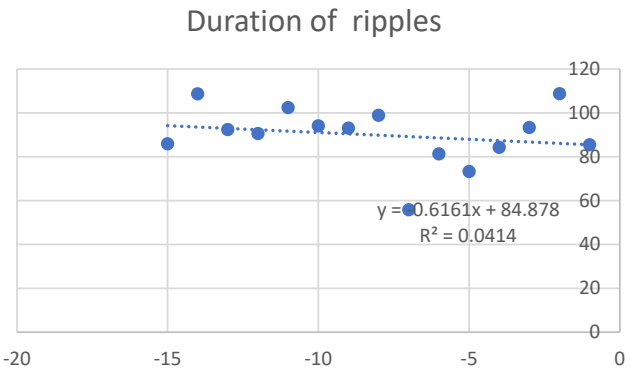

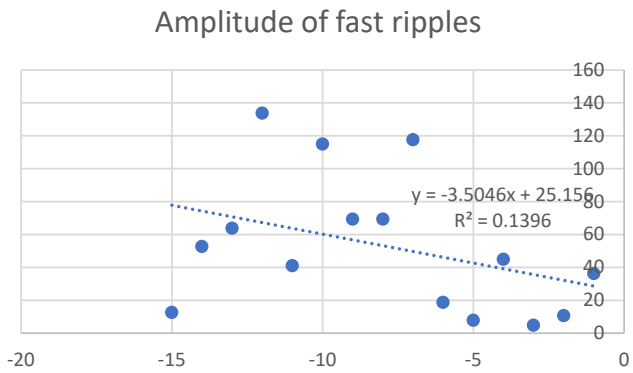

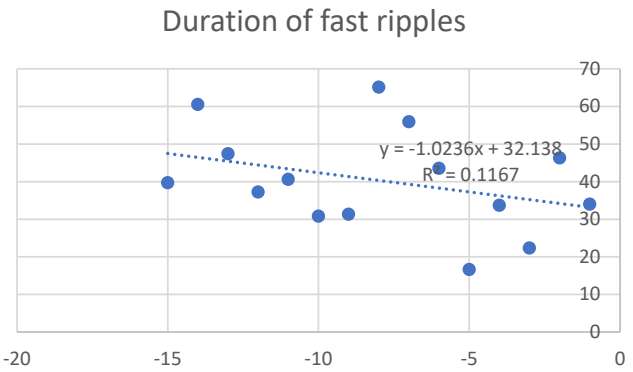

Patient 11 SZ1

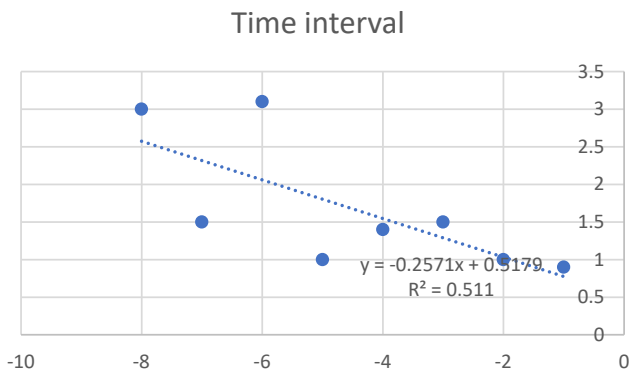

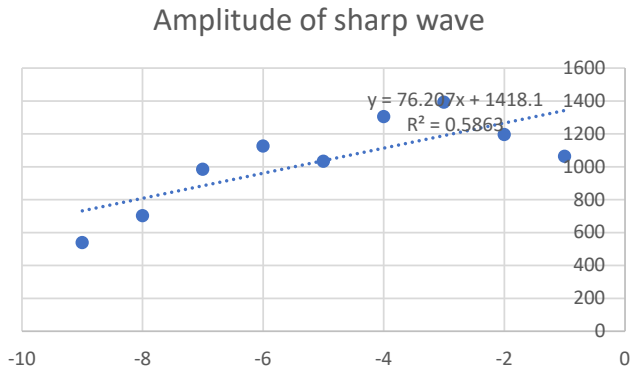

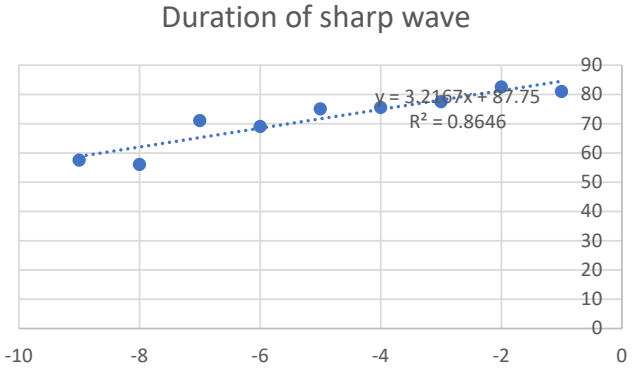

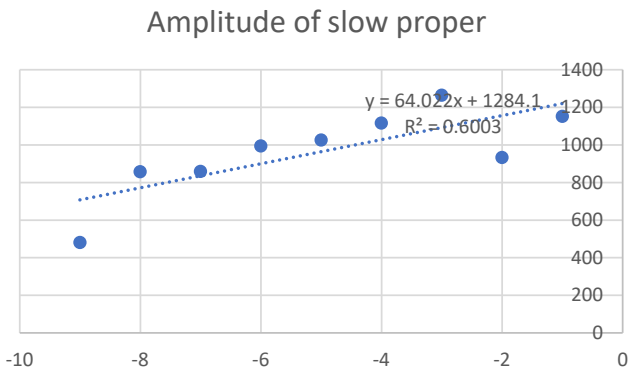

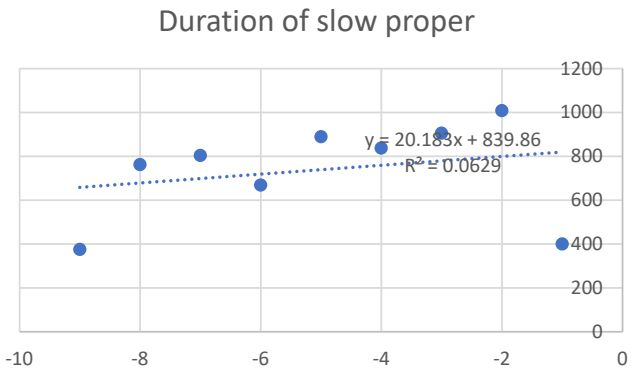

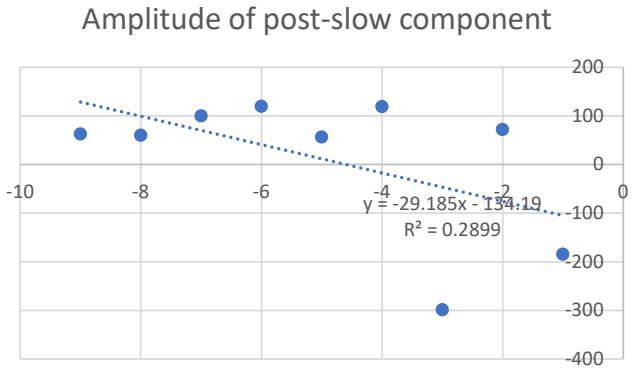

Patient 11 SZ1

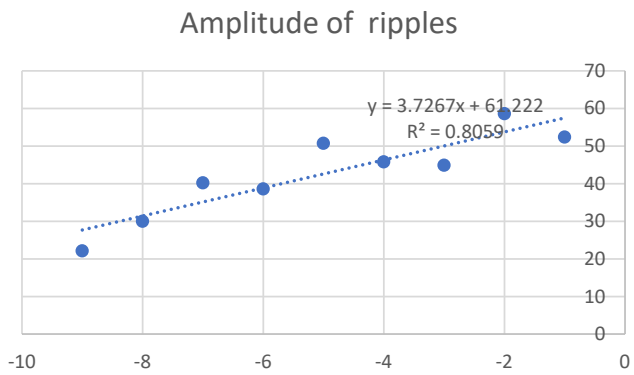

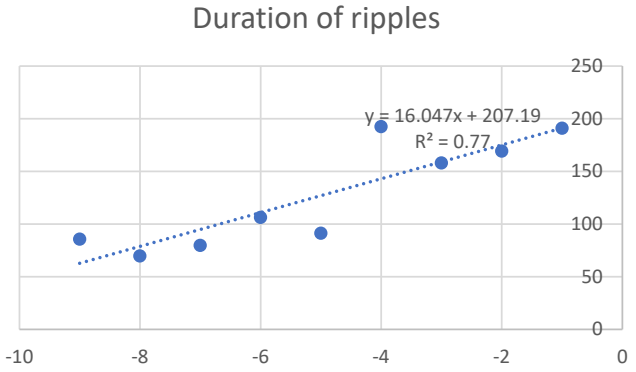

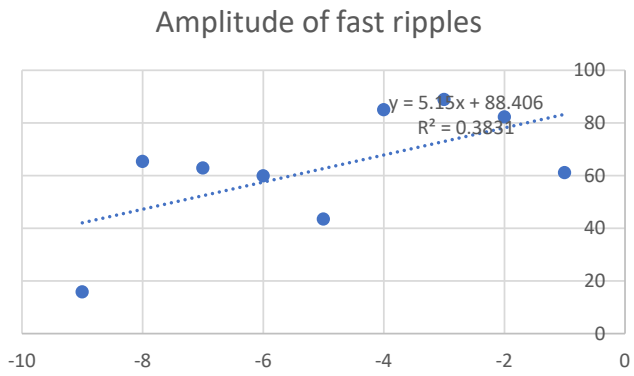

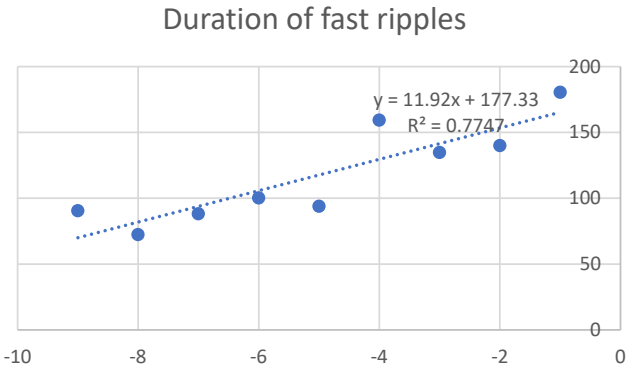

Patient 11 SZ2

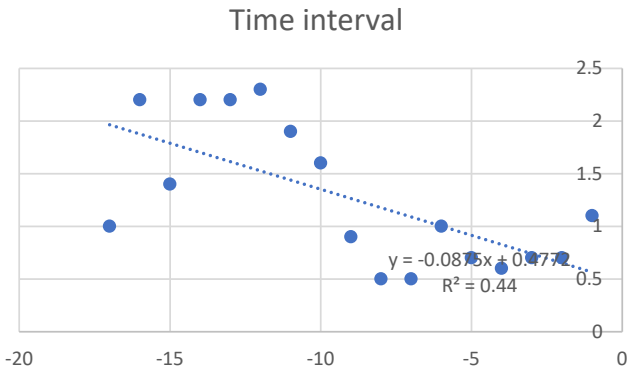

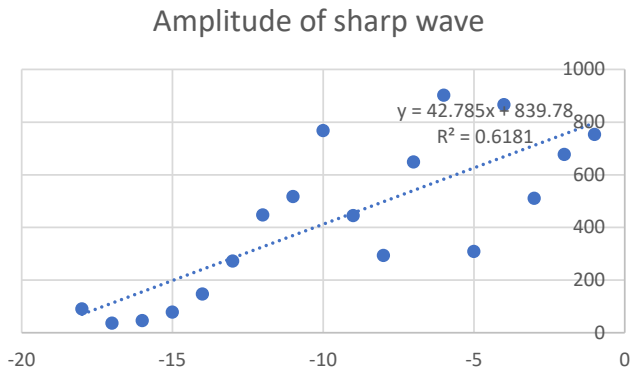

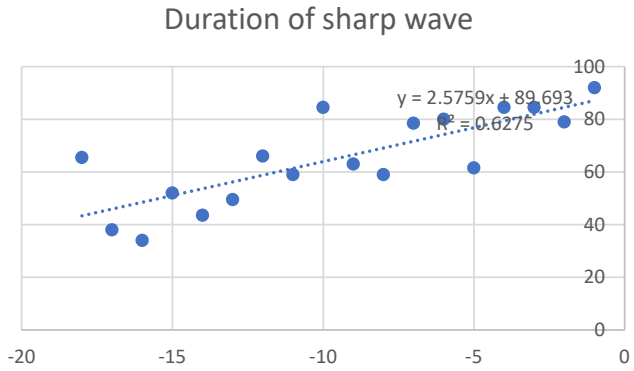

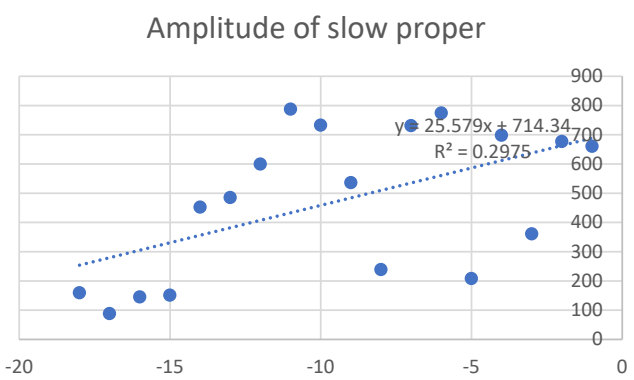

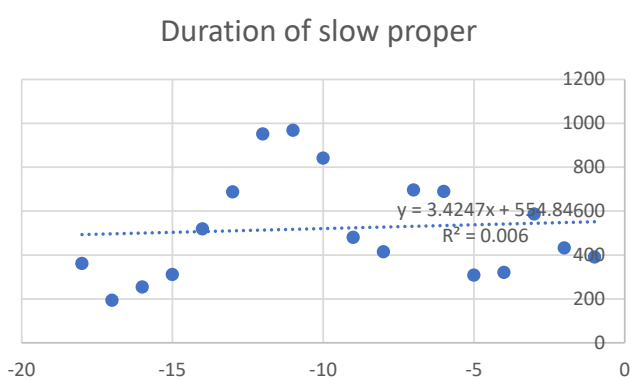

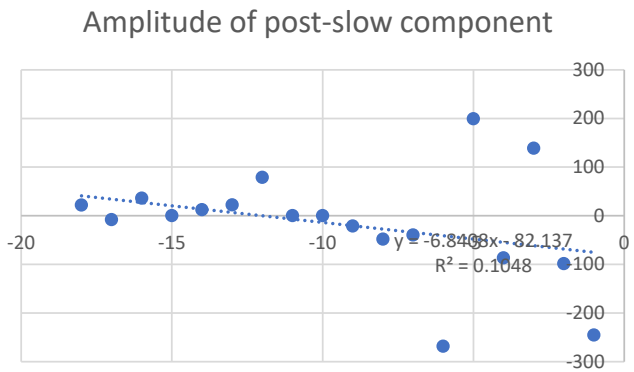

Patient 11 SZ2

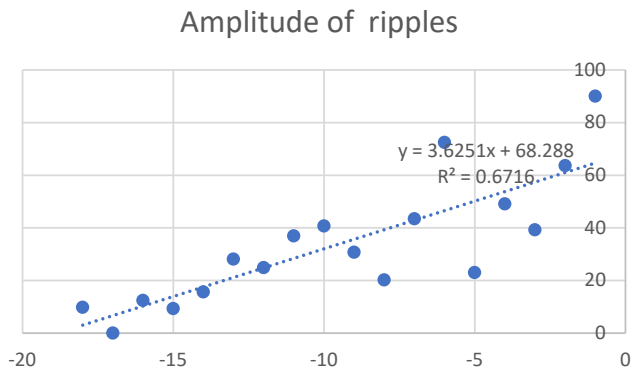

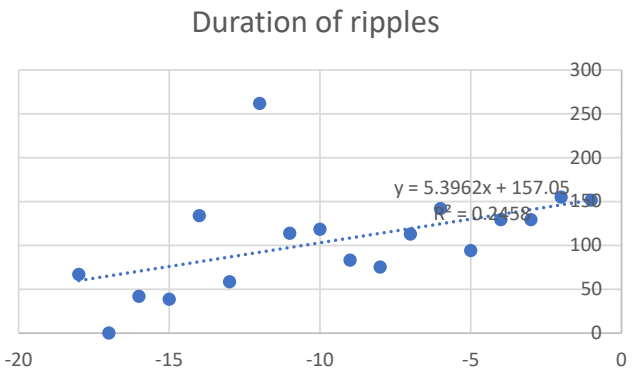

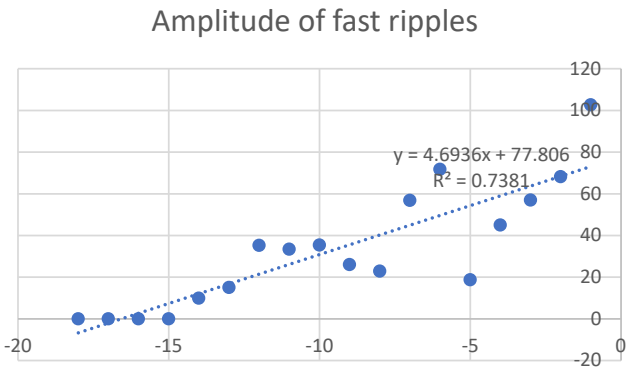

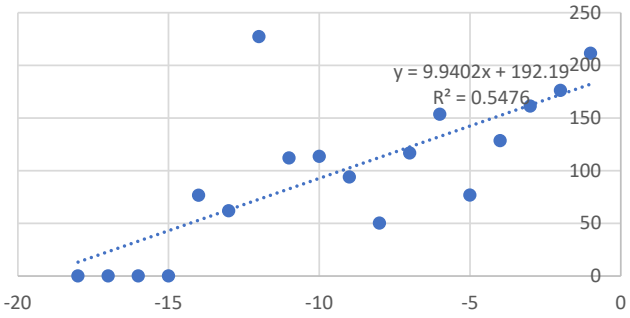

Patient 12 SZ1

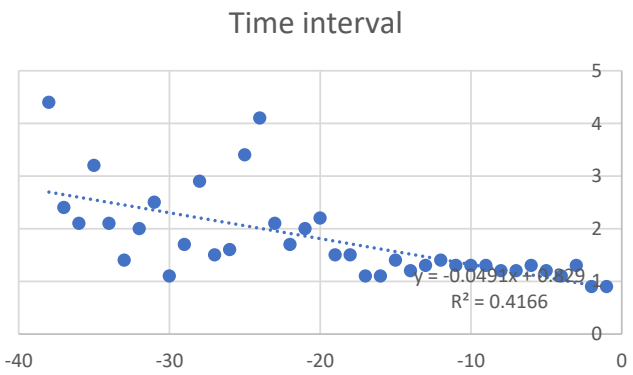

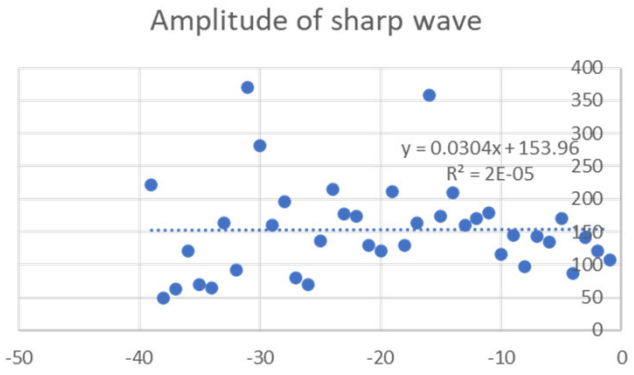

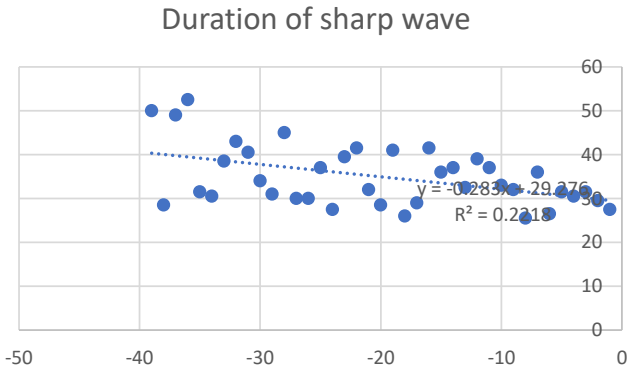

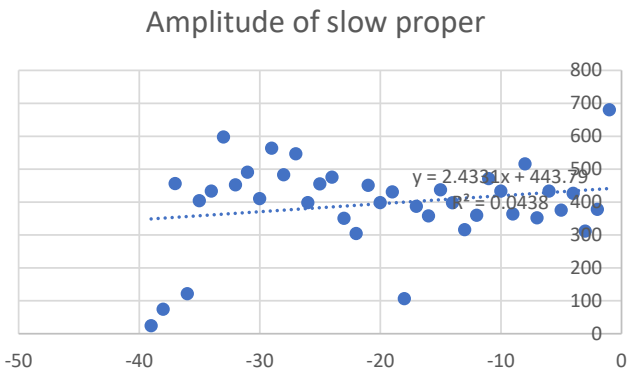

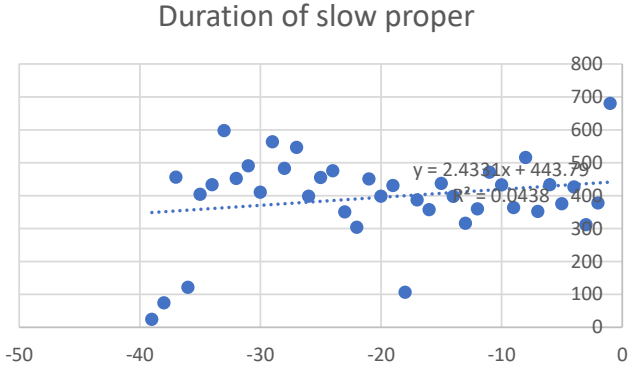

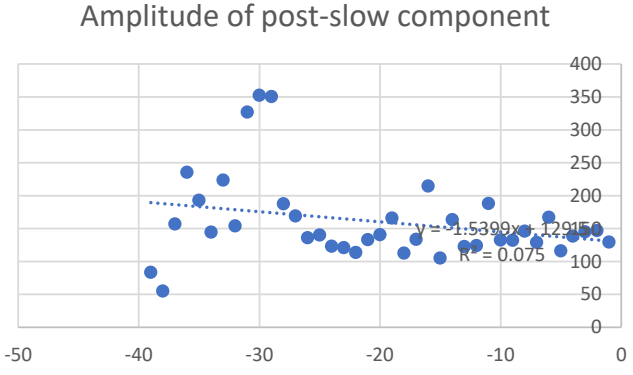

Patient 12 SZ1

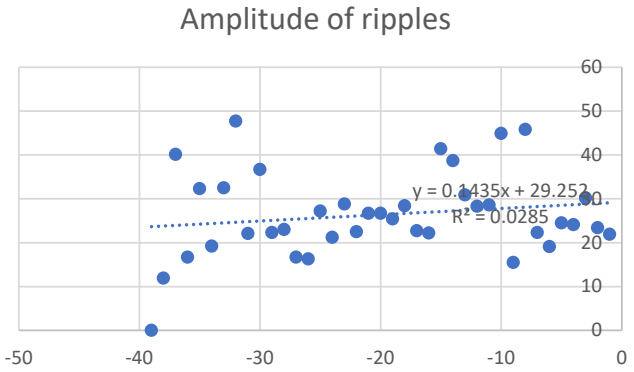

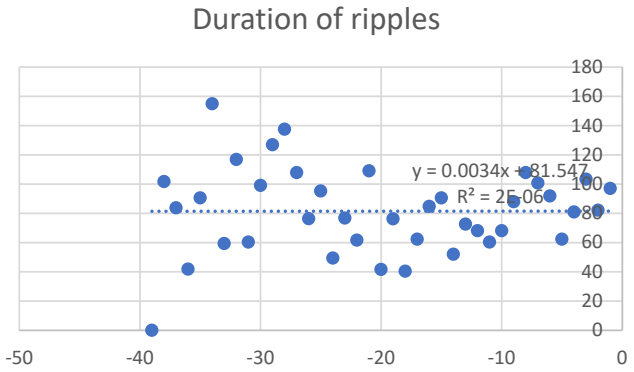

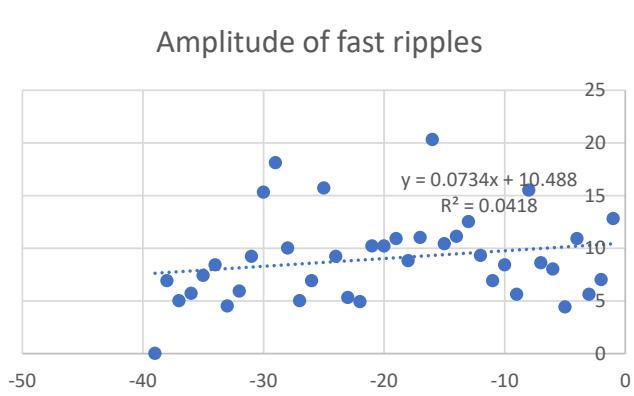

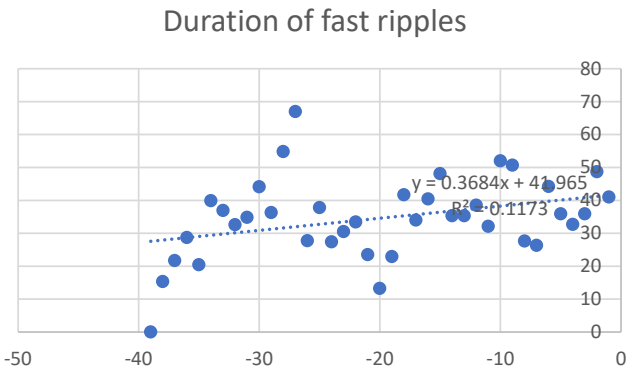

Patient 12 SZ2

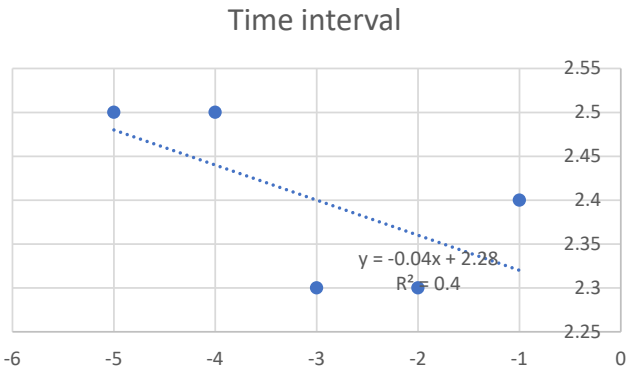

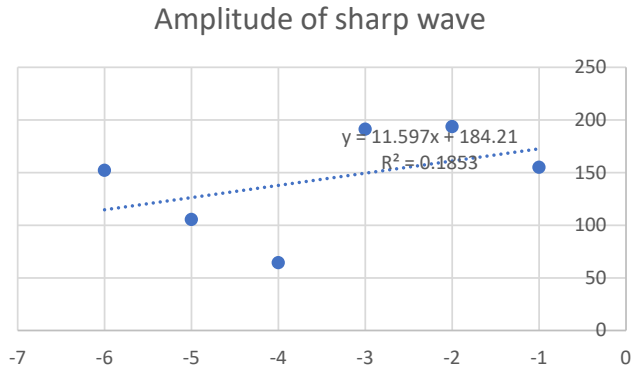

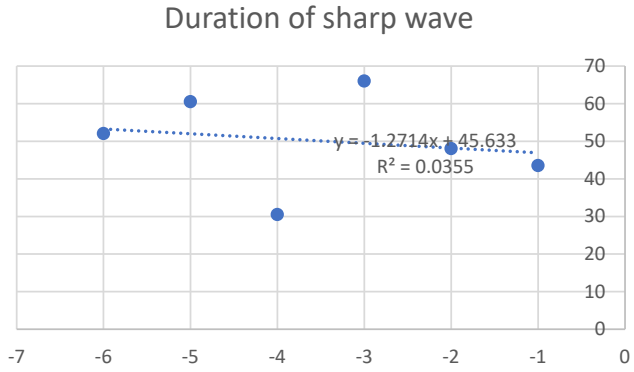

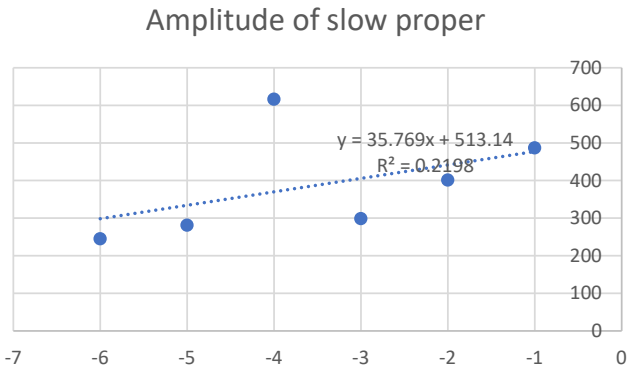

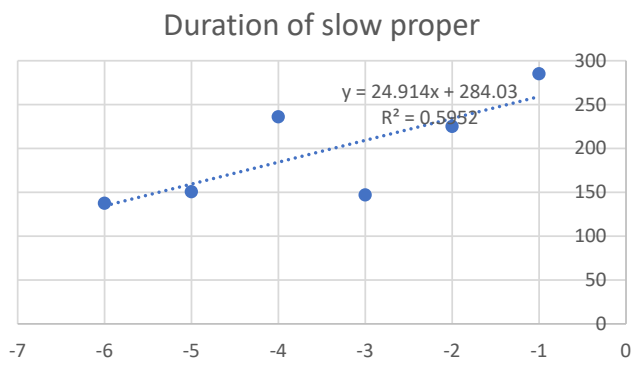

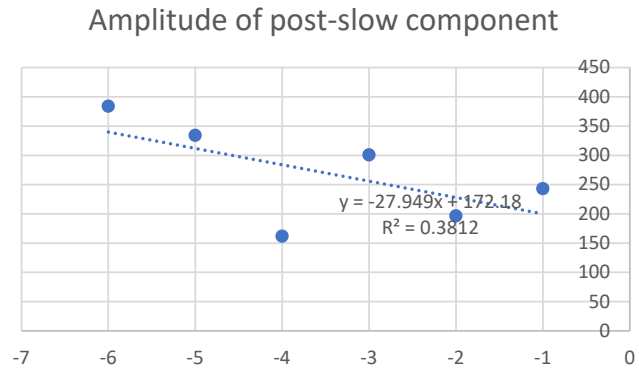

Patient 12 SZ2

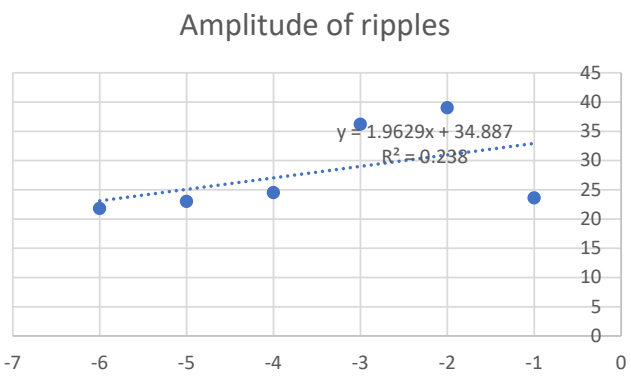

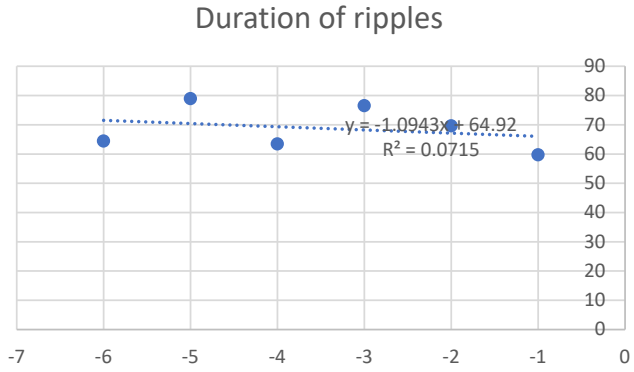

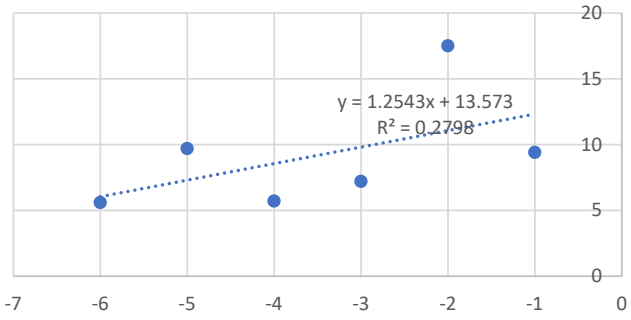

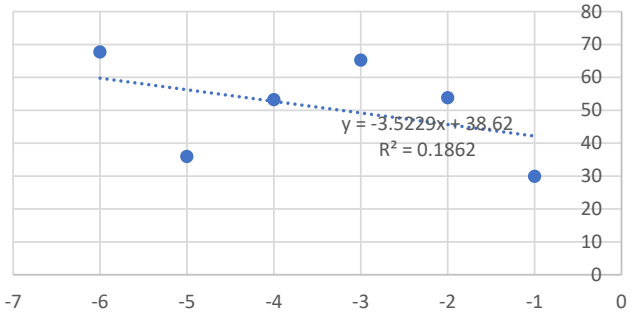

Patient 12 SZ3

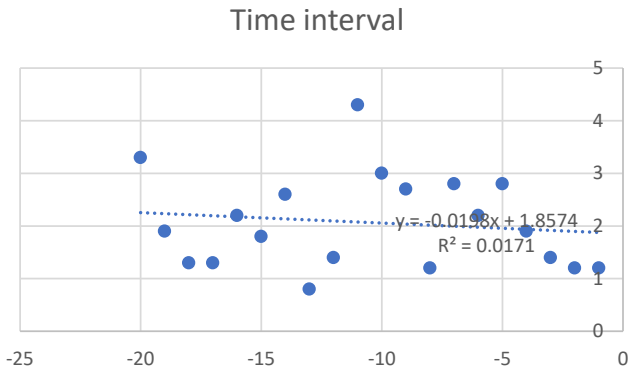

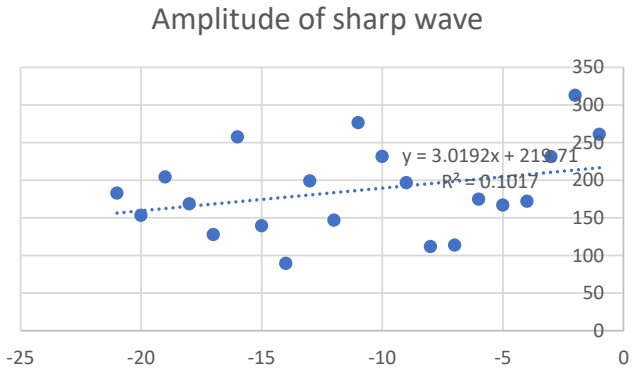

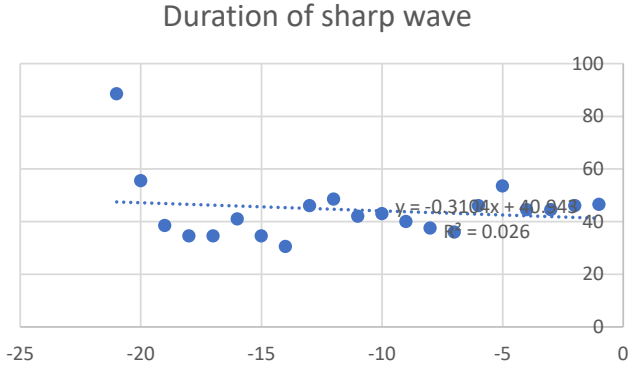

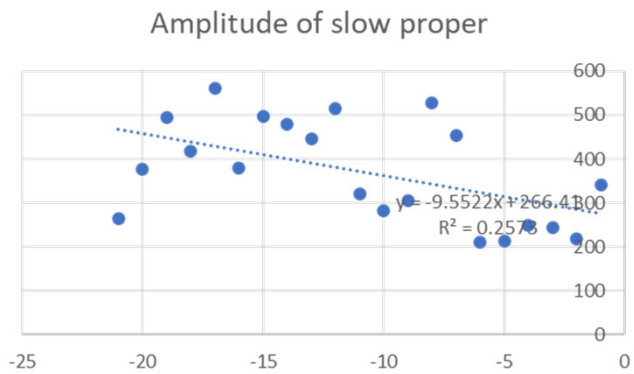

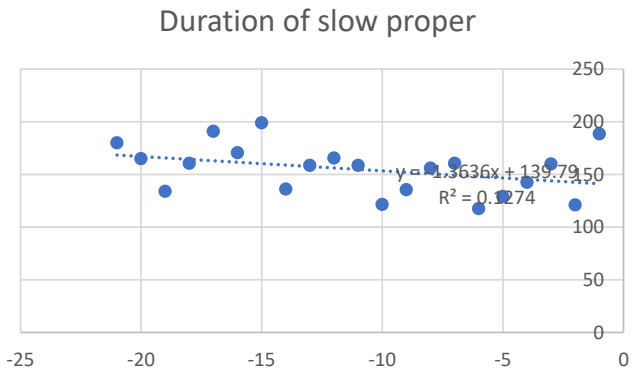

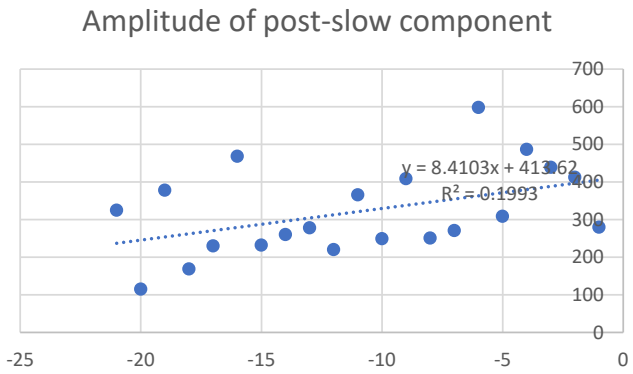

Patient 12 SZ3

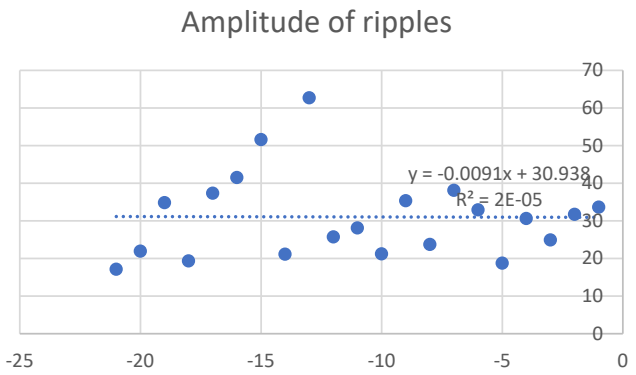

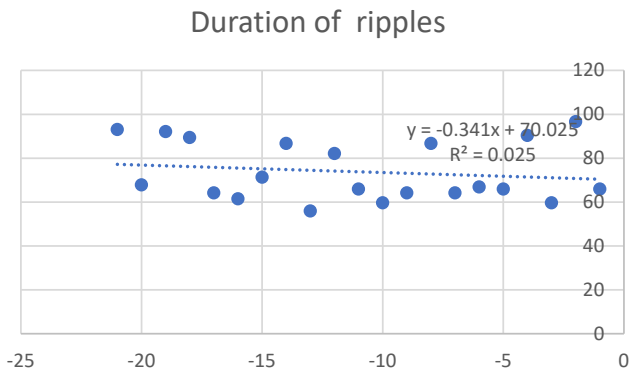

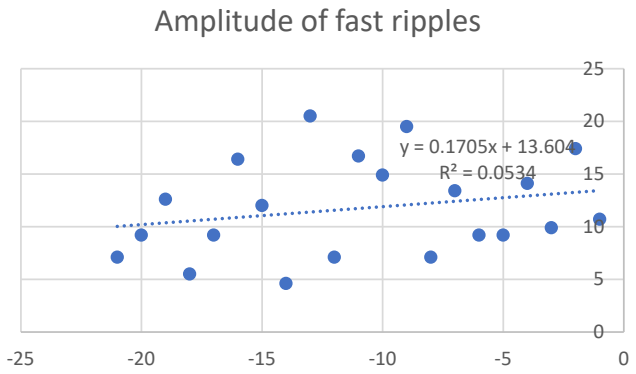

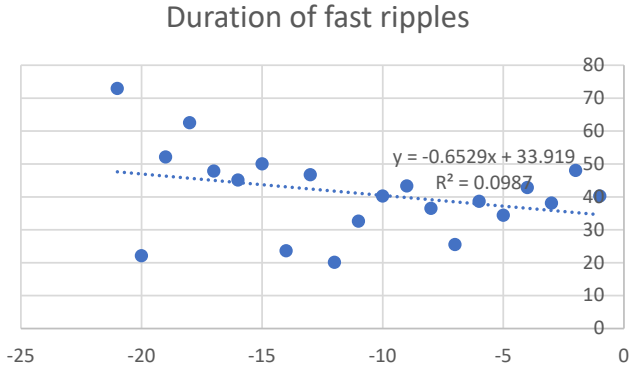

Patient 12 SZ4

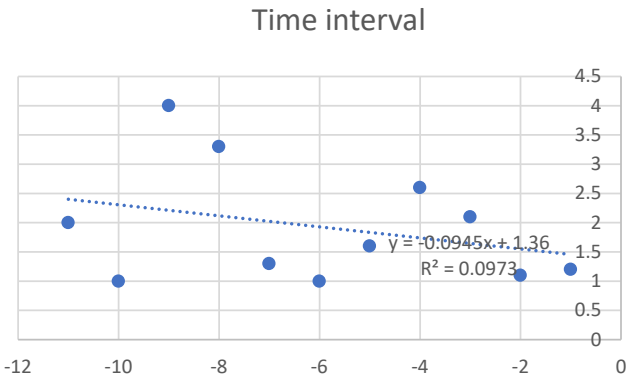

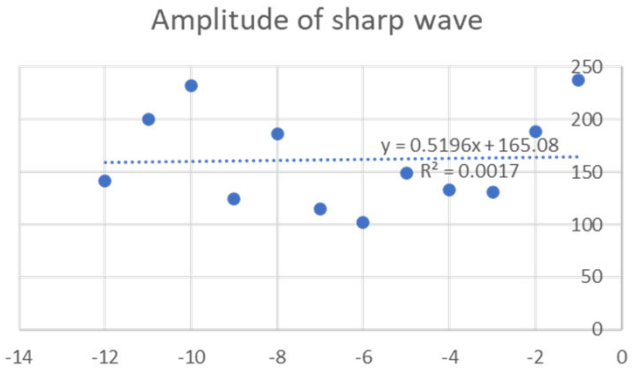

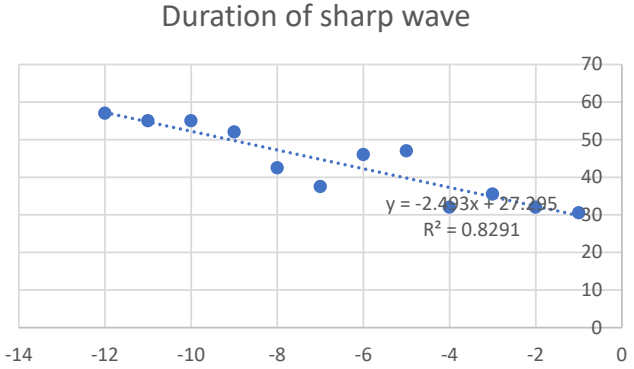

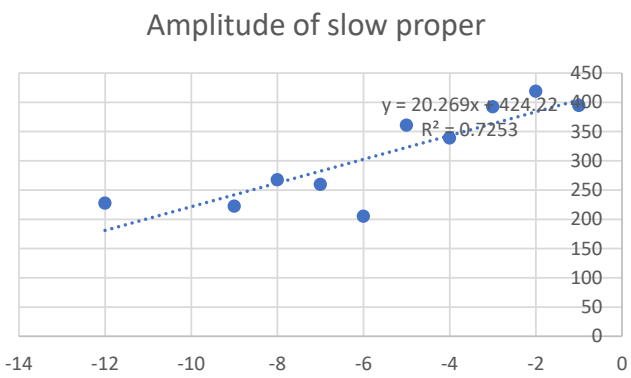

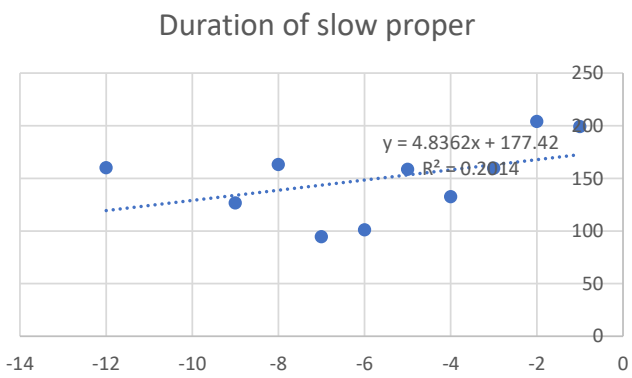

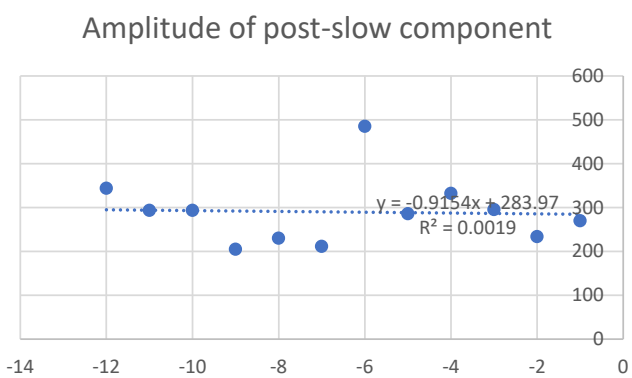

Patient 12 SZ4

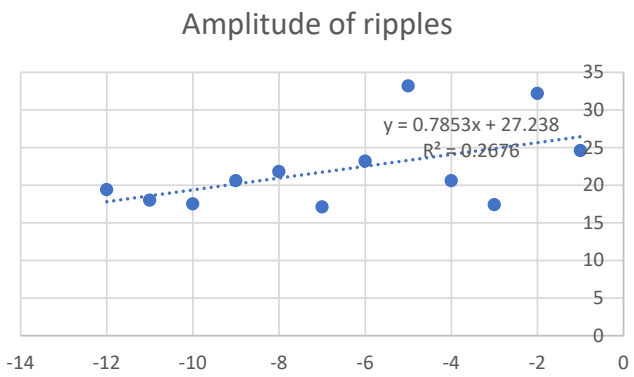

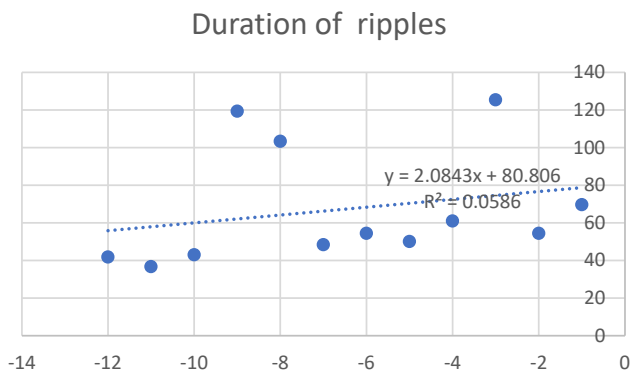

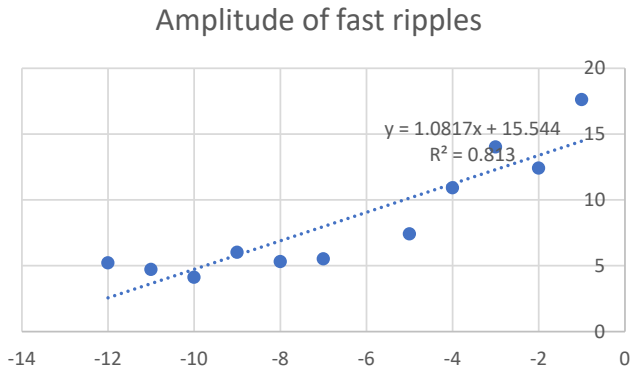

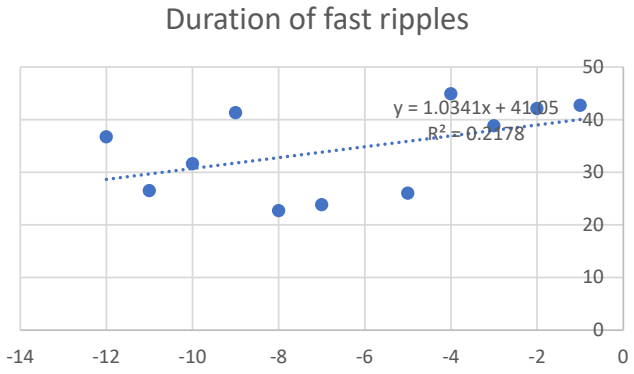

Patient 12 SZ5

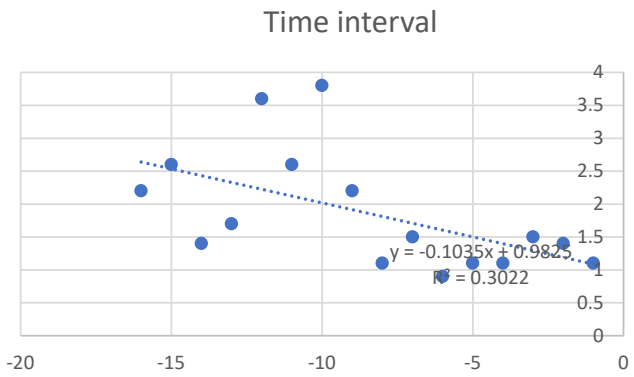

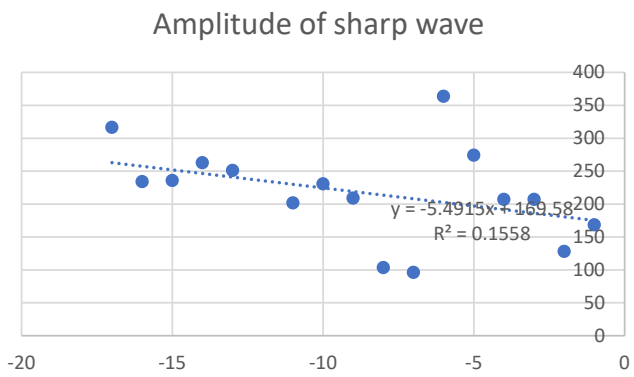

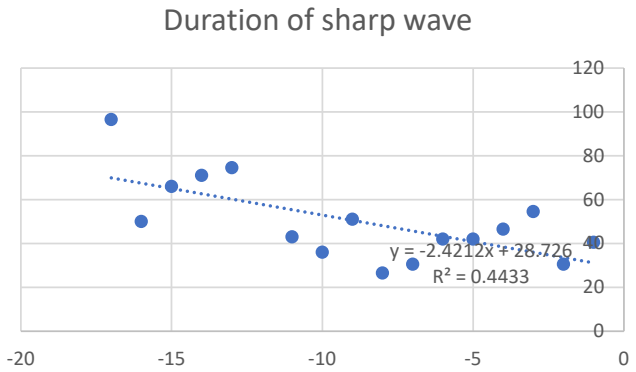

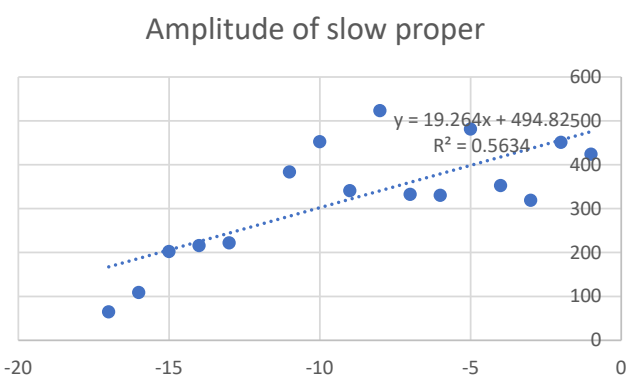

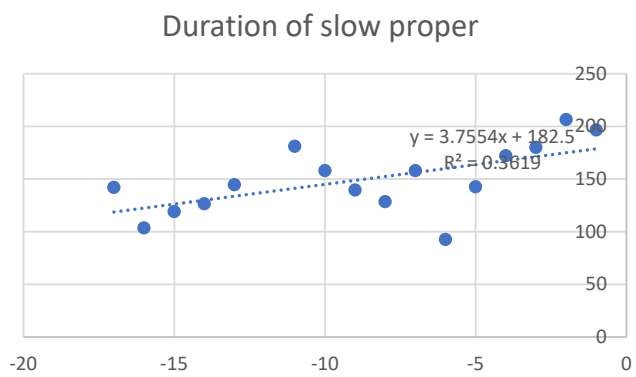

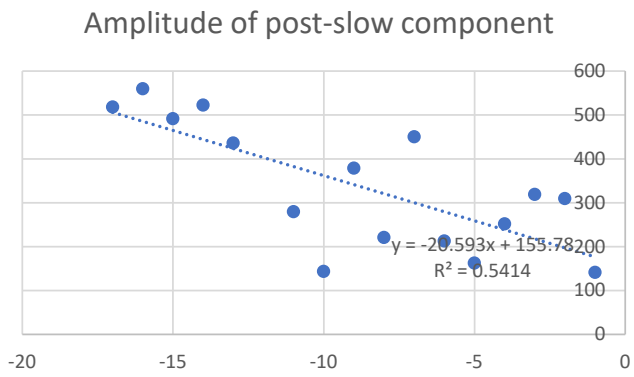

Patient 12 SZ5

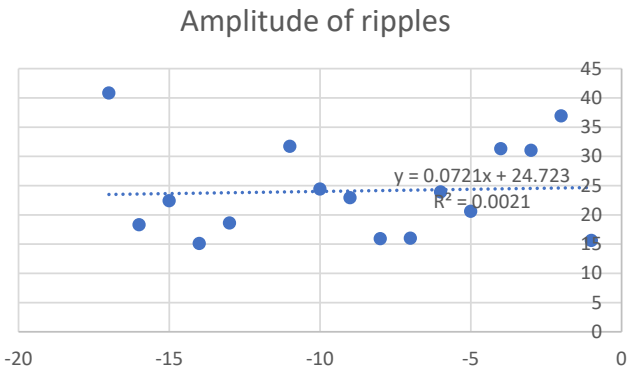

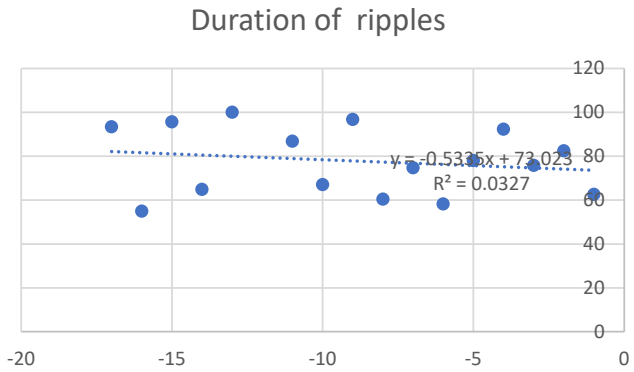

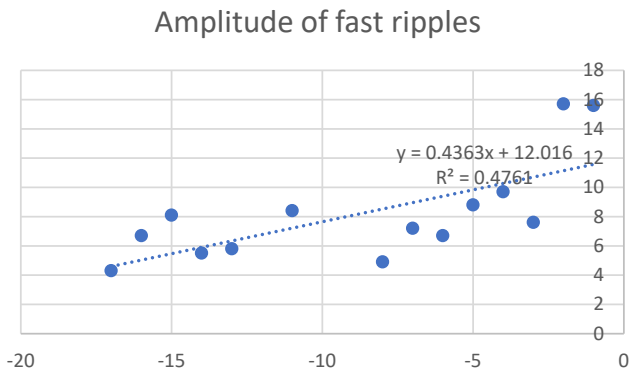

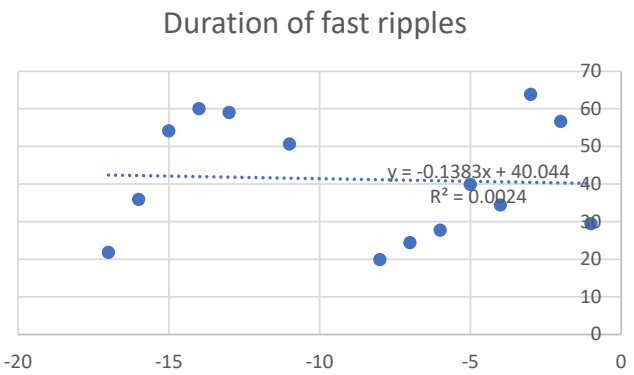

Patient 13 SZ1

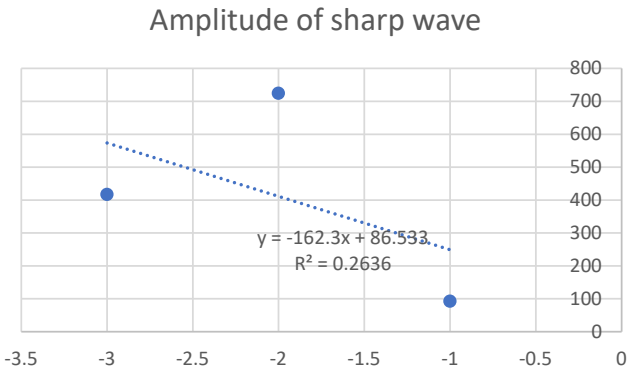

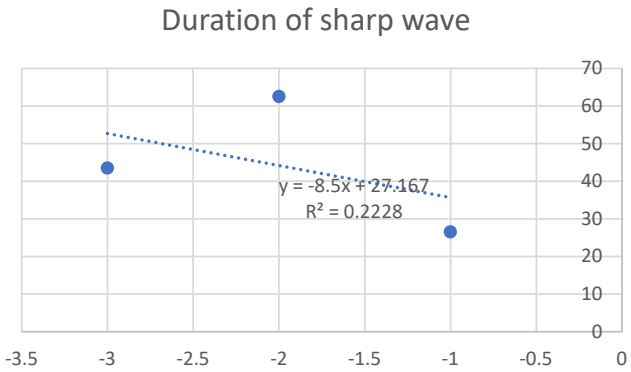

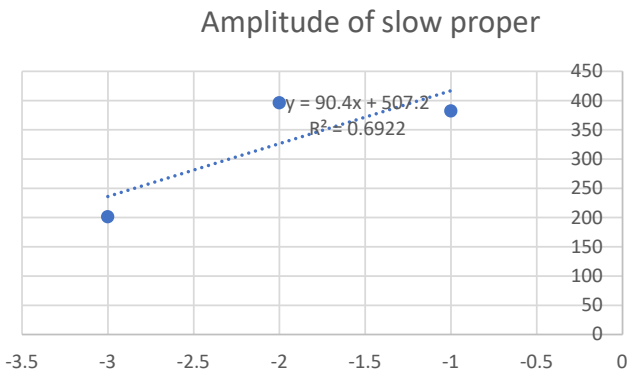

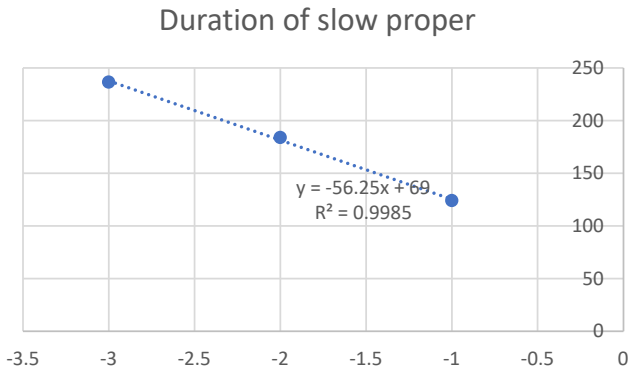

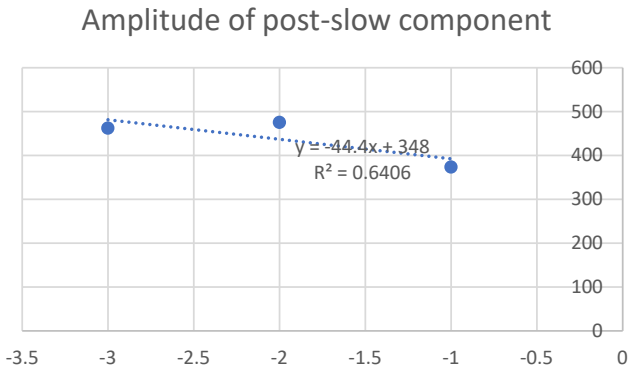

Patient 13 SZ1

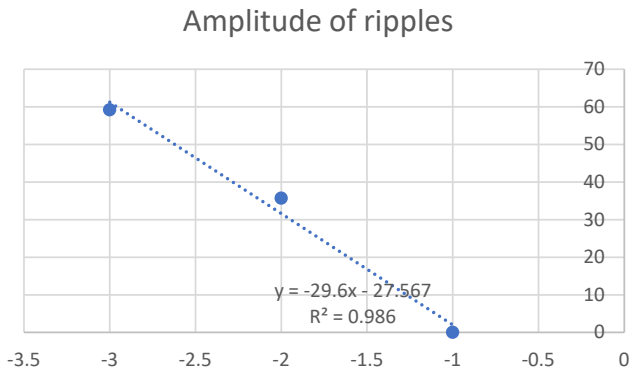

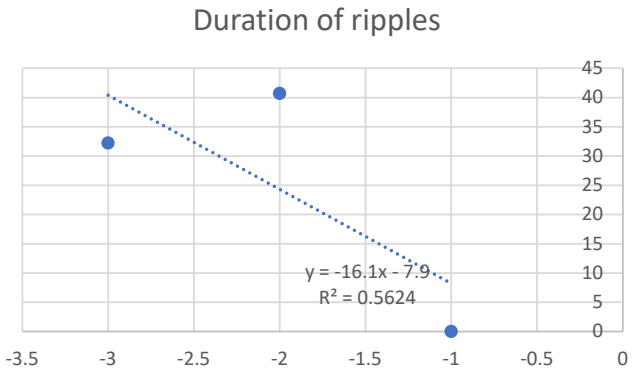

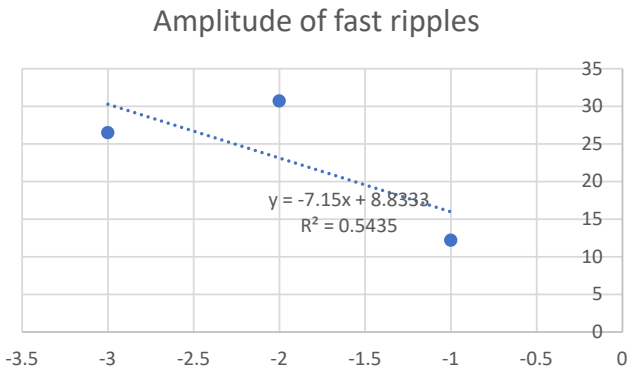

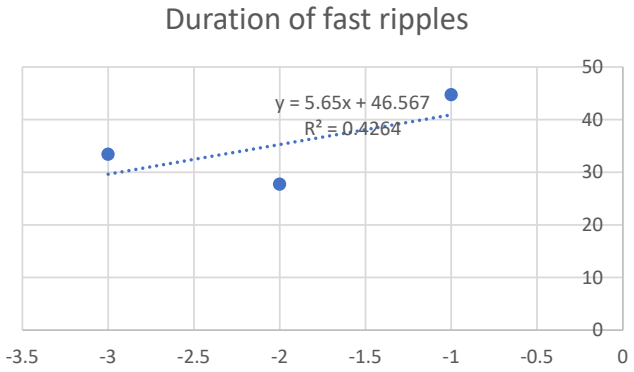

Patient 13 SZ2

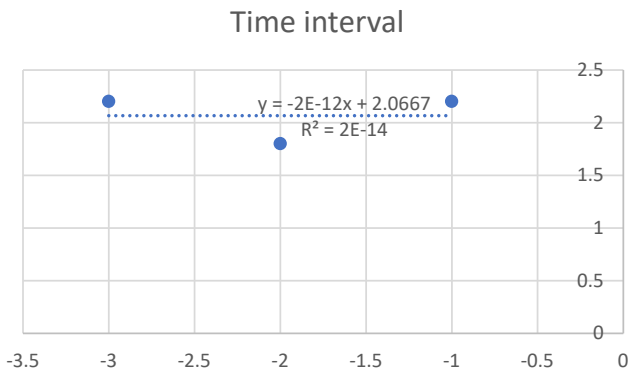

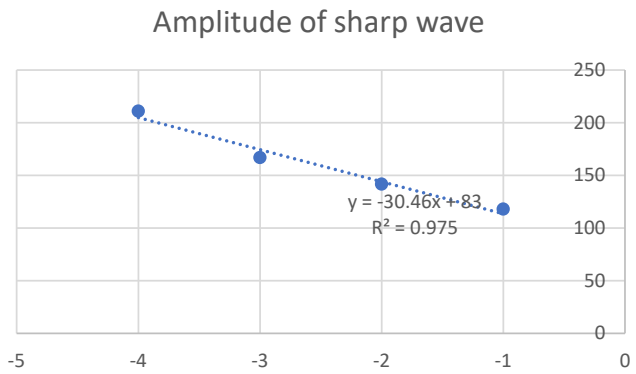

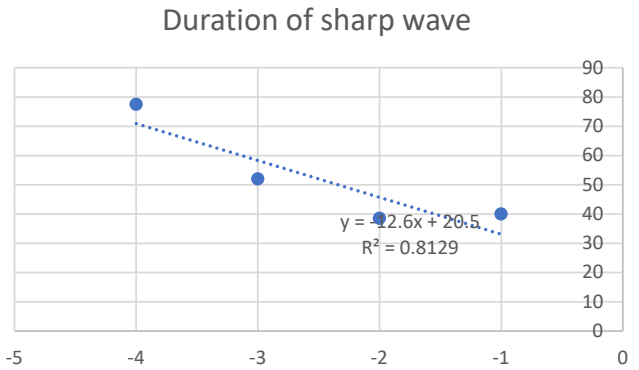

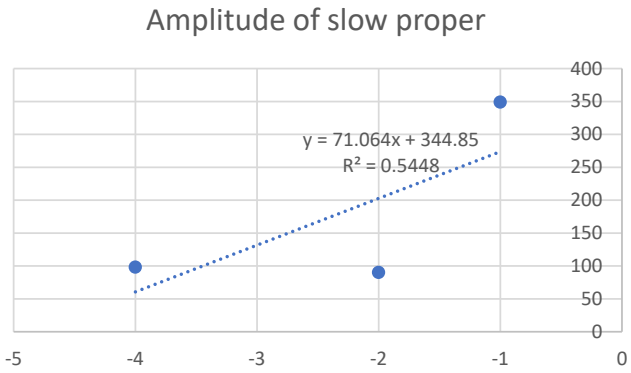

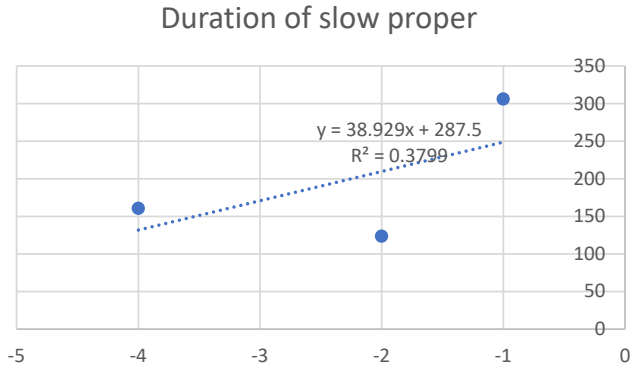

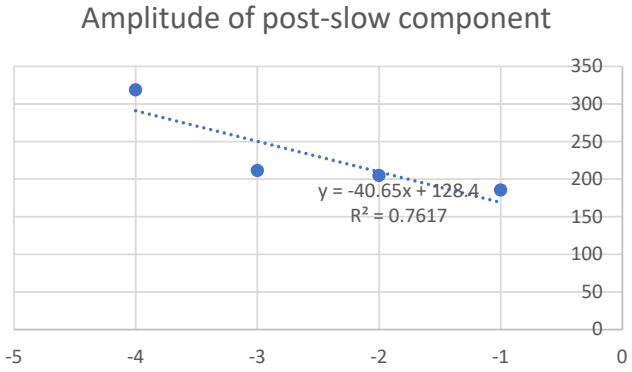

Patient 13 SZ2

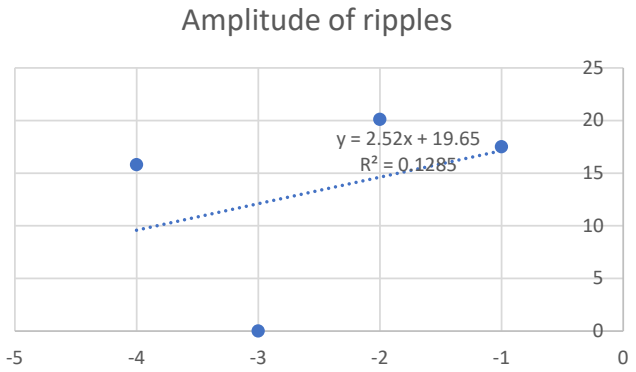

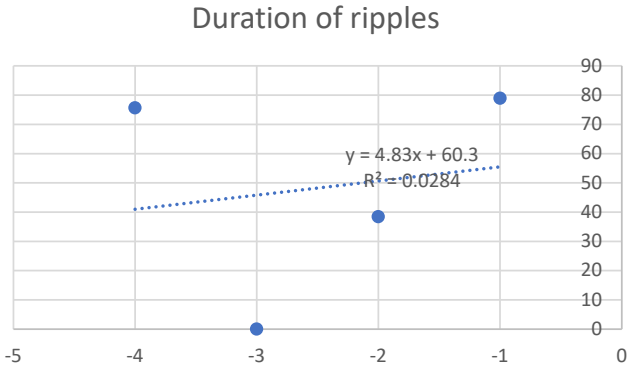

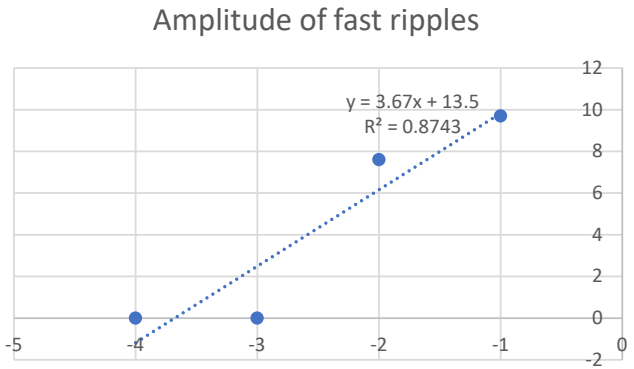

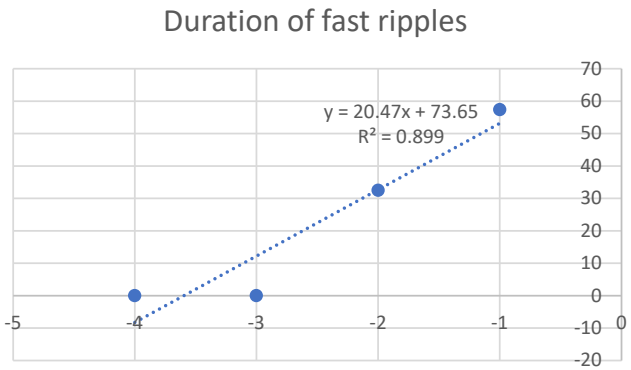

Patient 14 SZ1

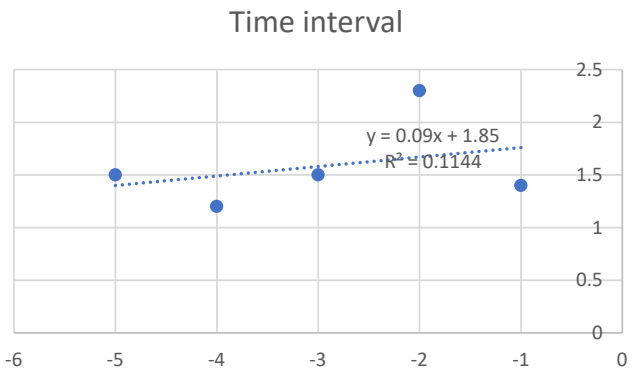

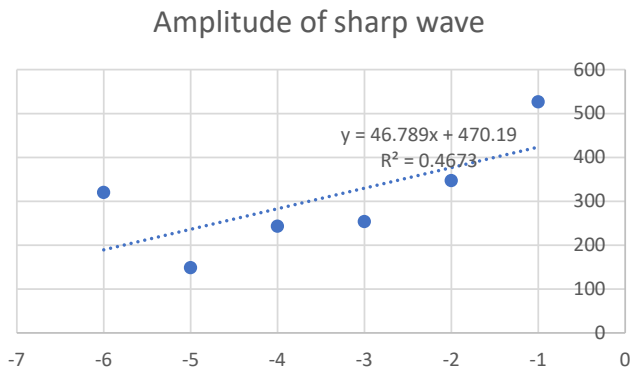

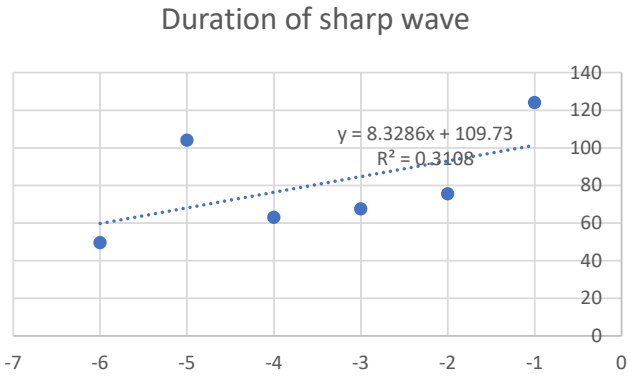

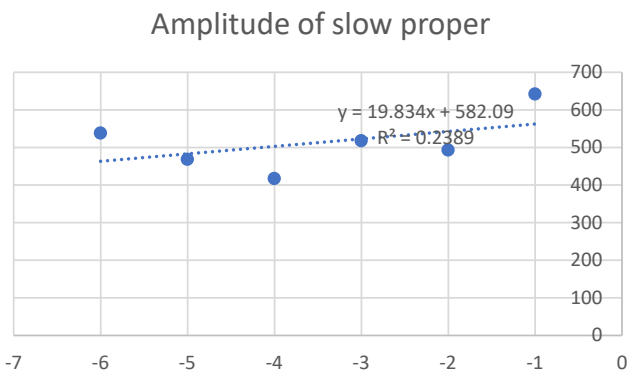

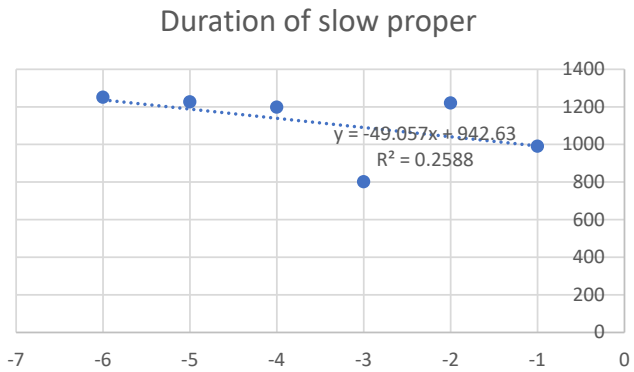

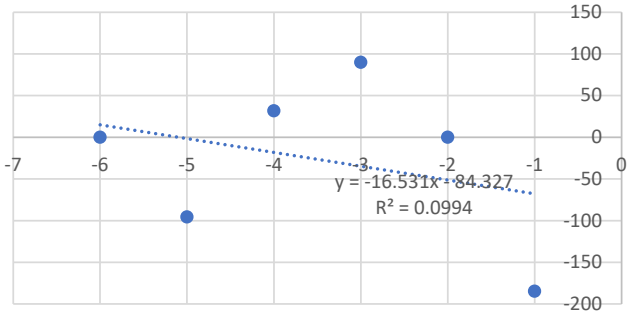

Patient 14 SZ1

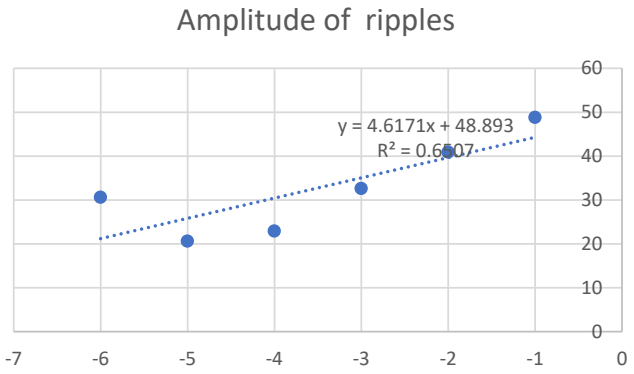

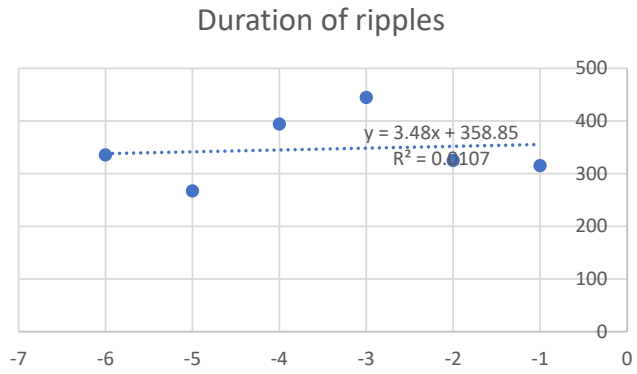

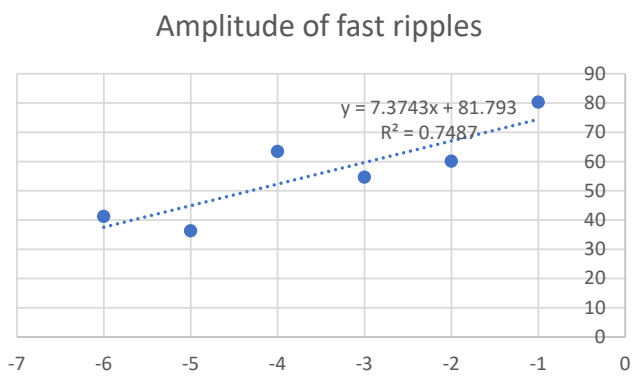

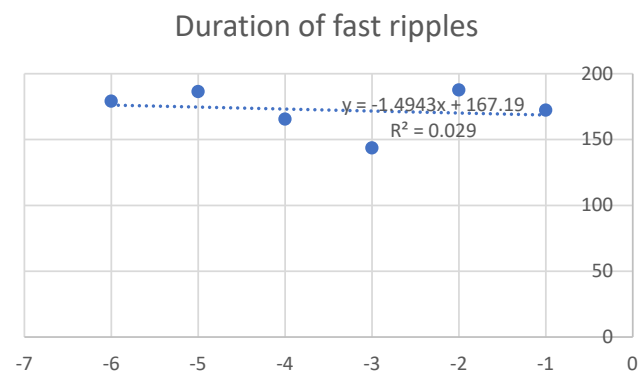

Patient 14 SZ2

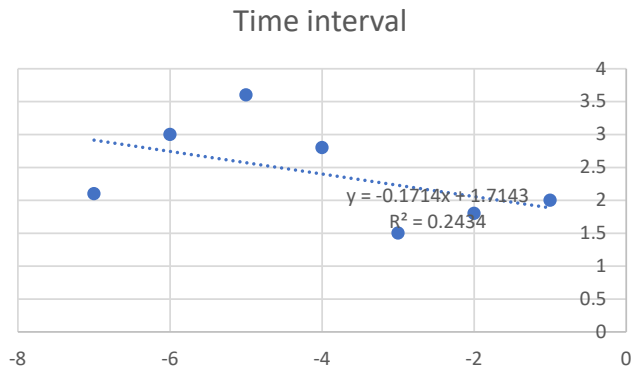

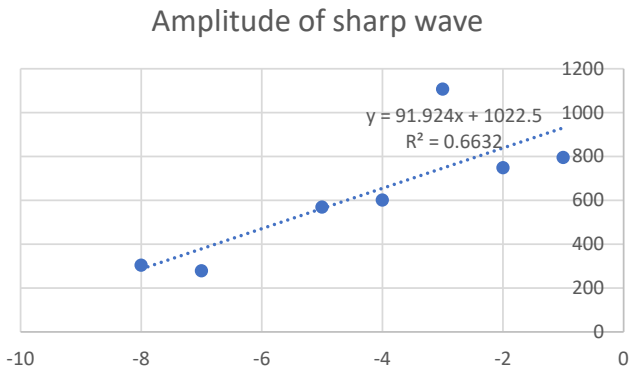

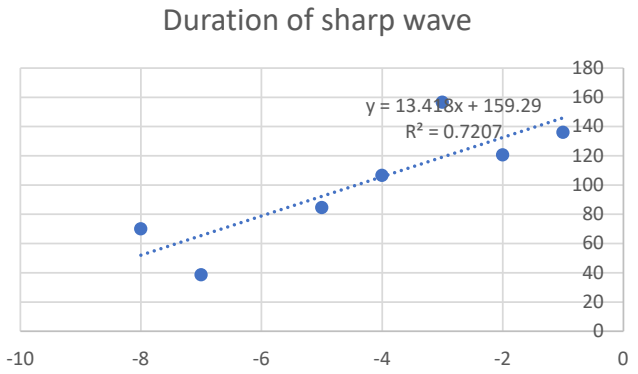

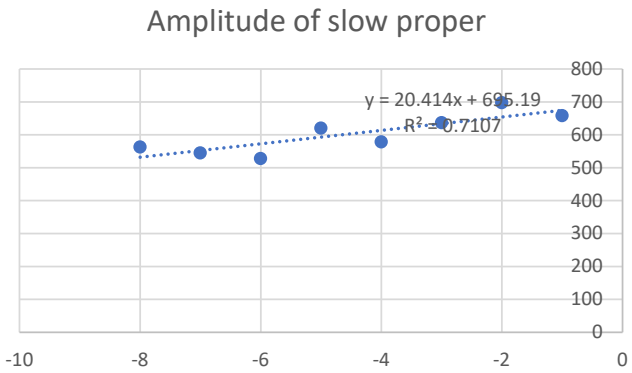

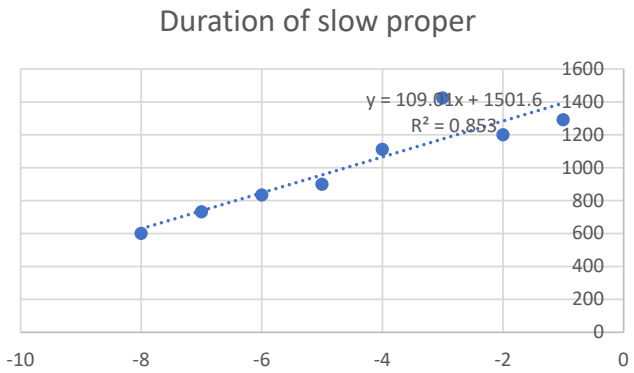

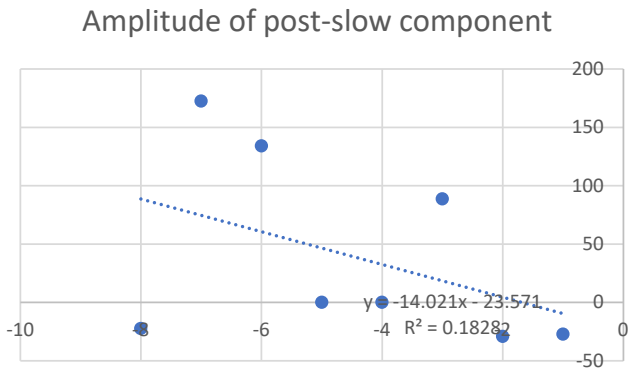

Patient 14 SZ2

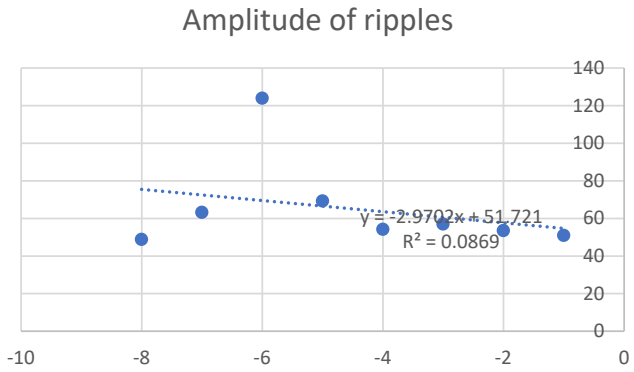

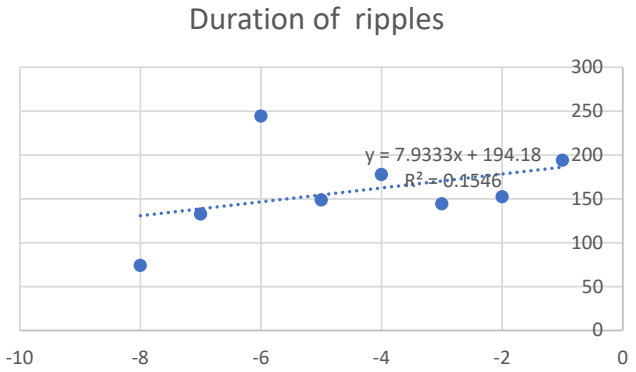

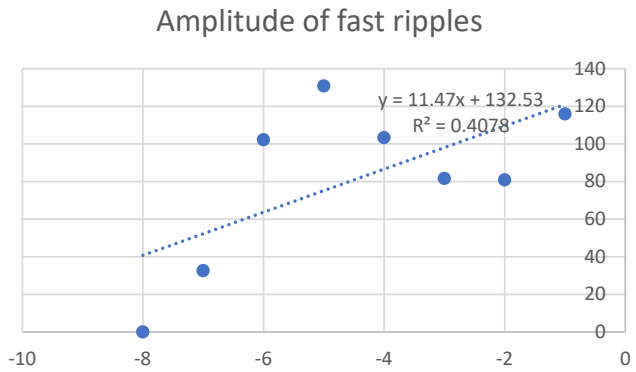

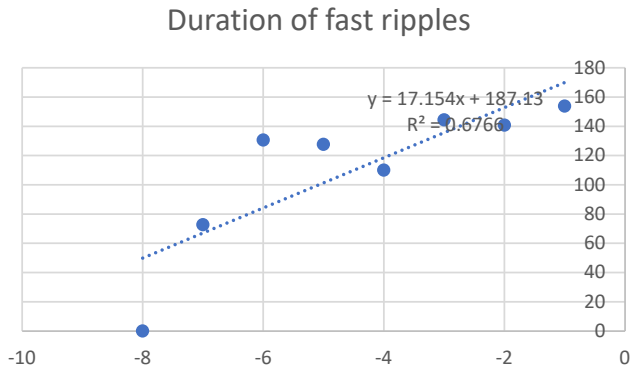

Patient 14 SZ3

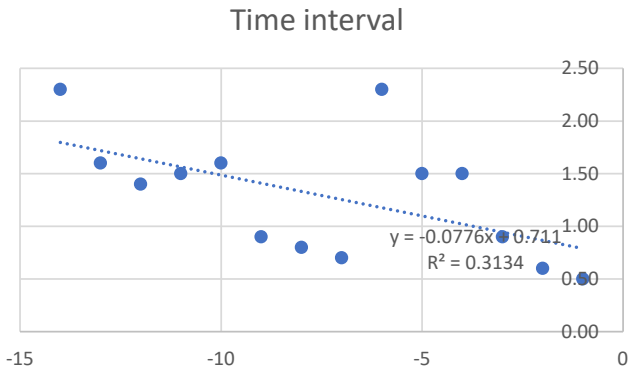

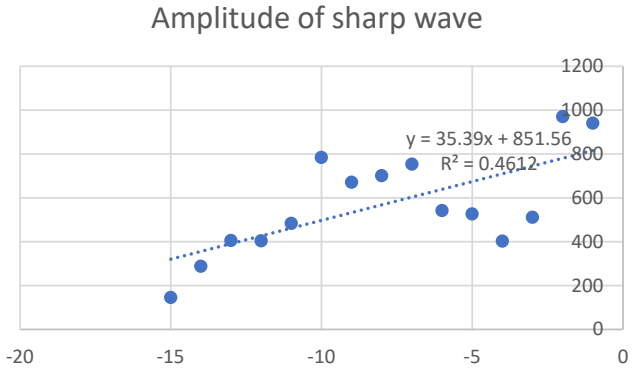

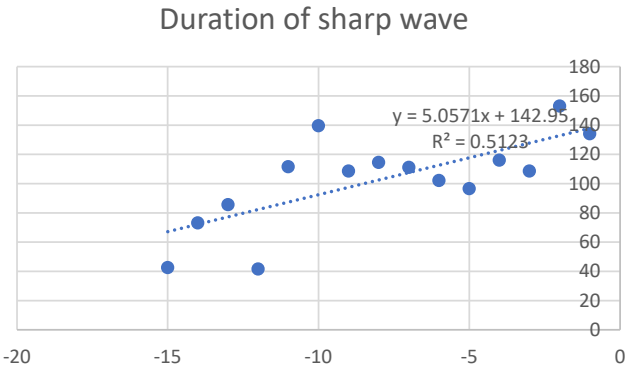

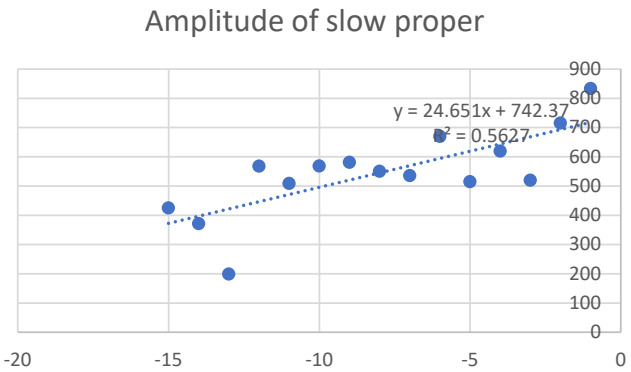

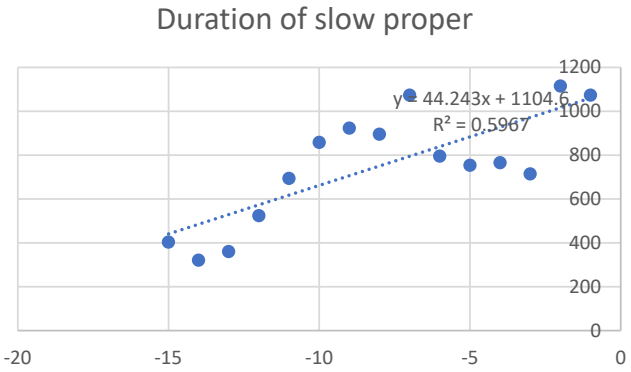

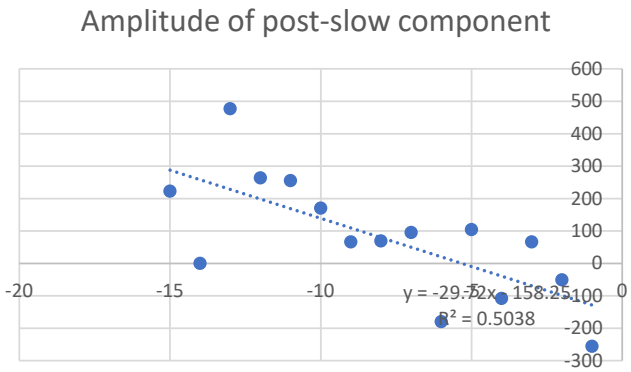

Patient 14 SZ3

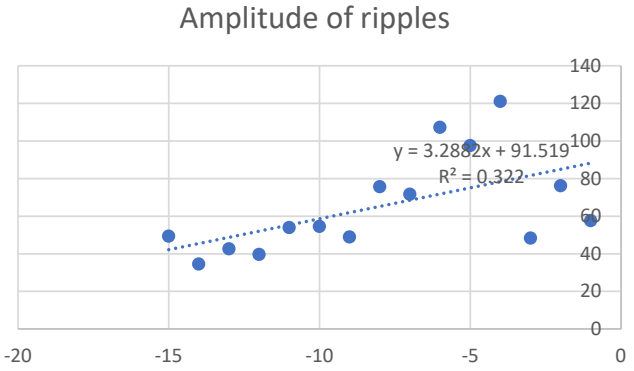

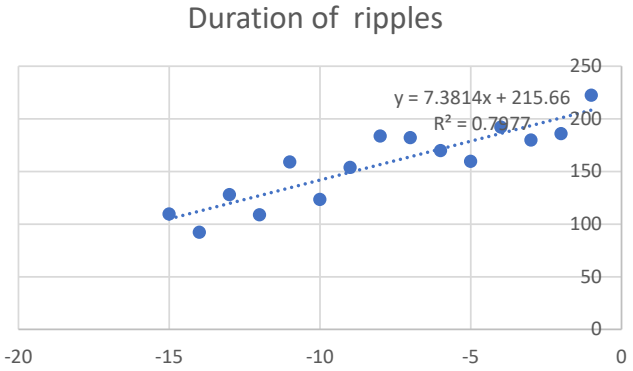

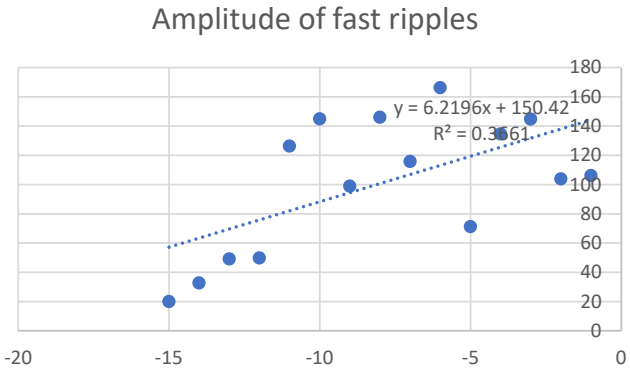

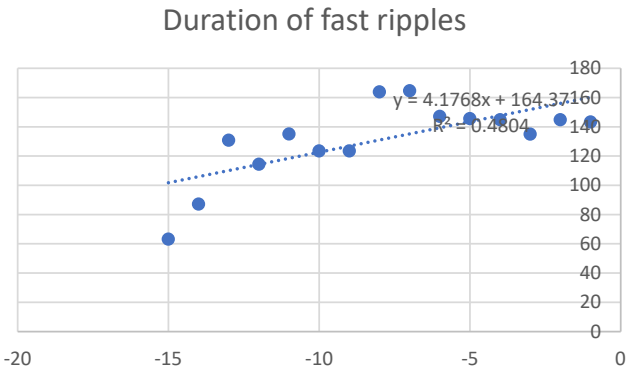

Patient 15 SZ1

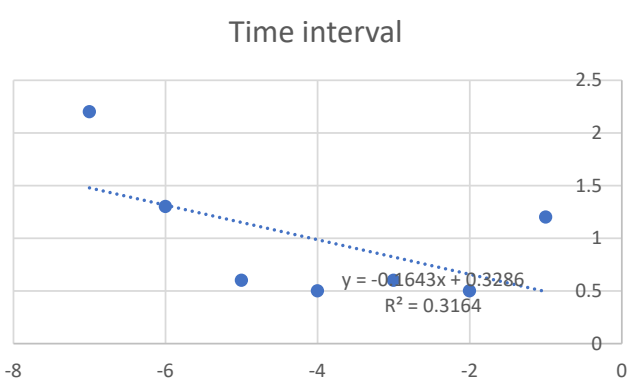

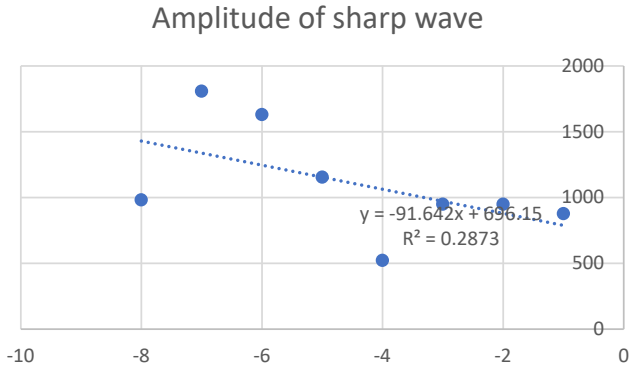

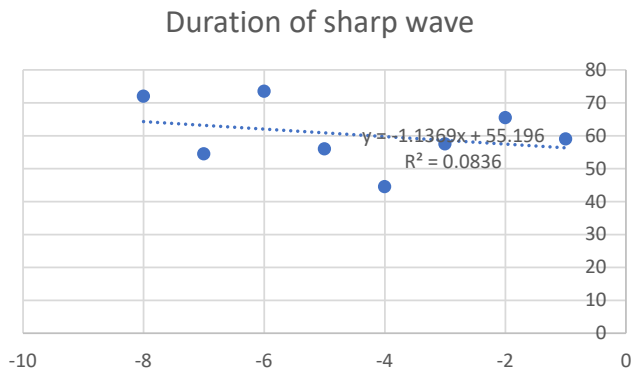

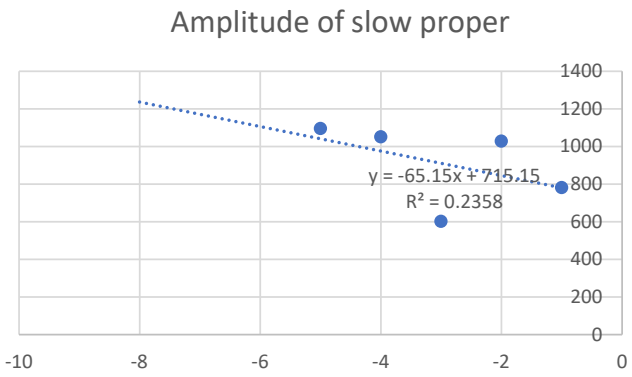

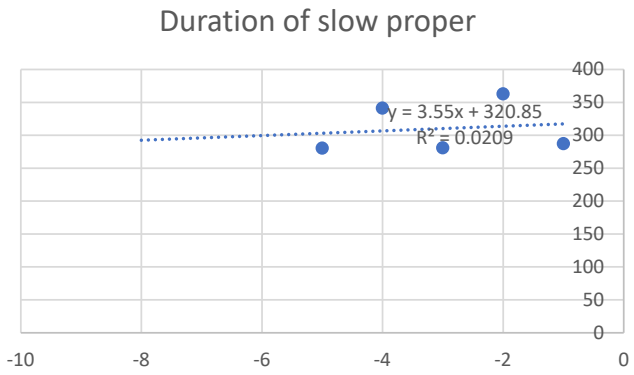

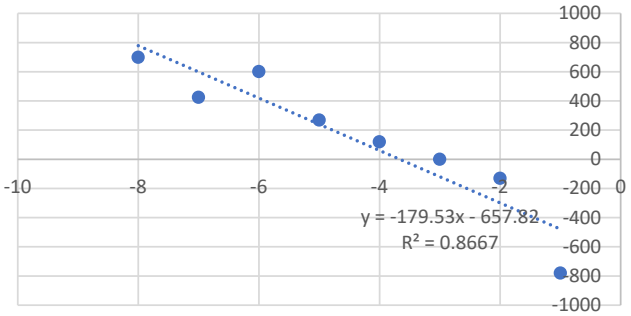

Patient 15 SZ1

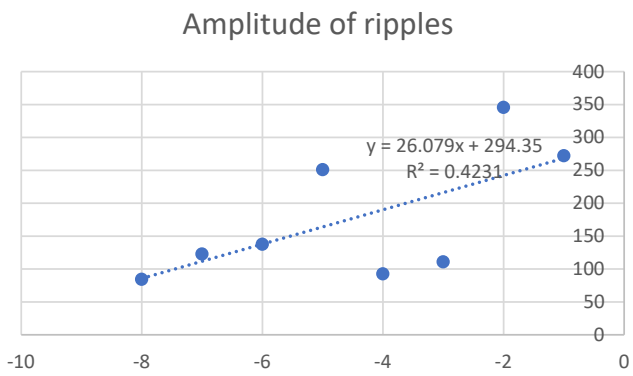

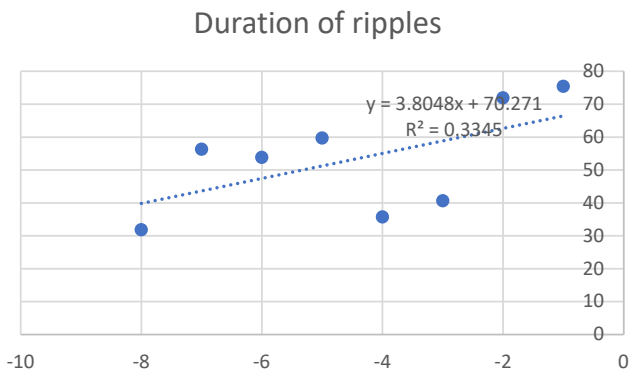

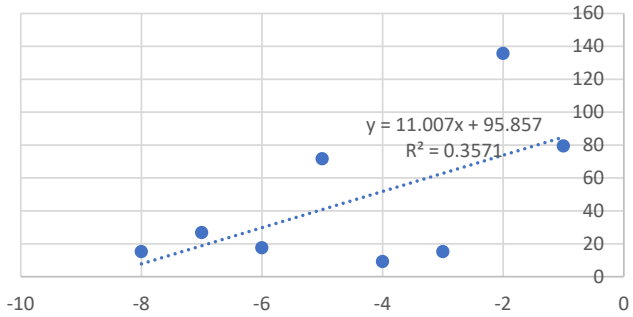

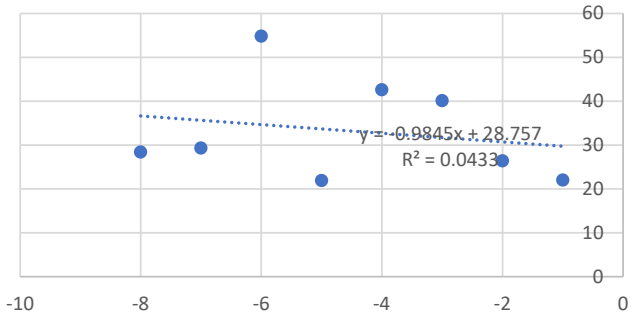

Patient 15 SZ2

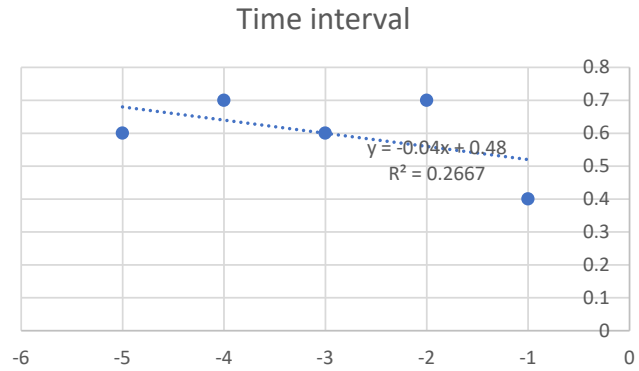

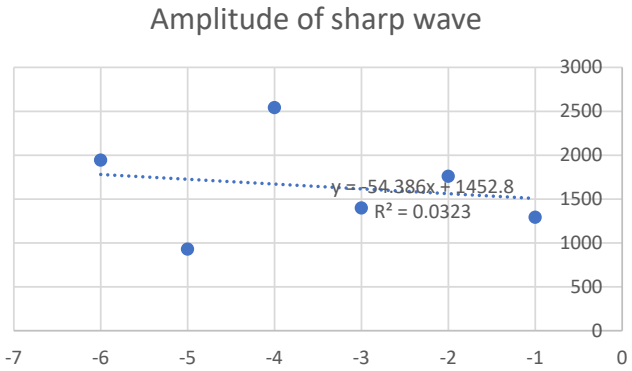

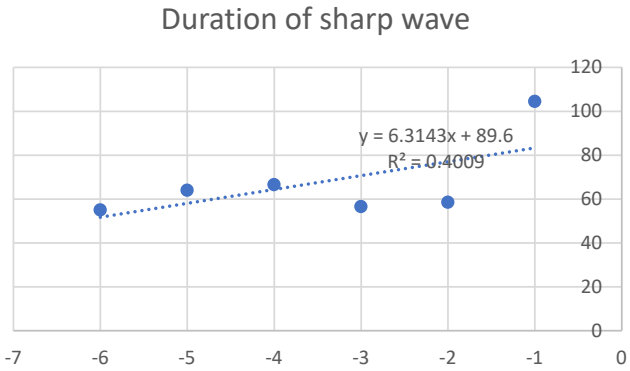

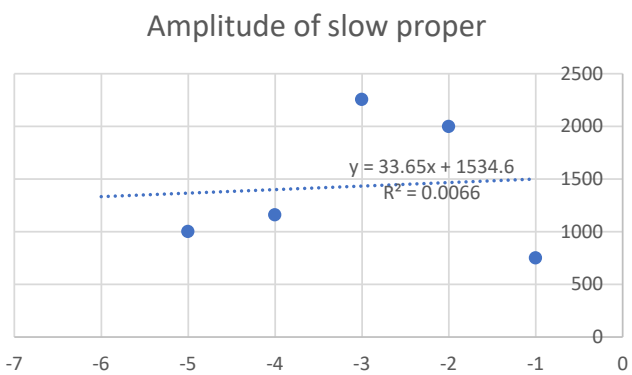

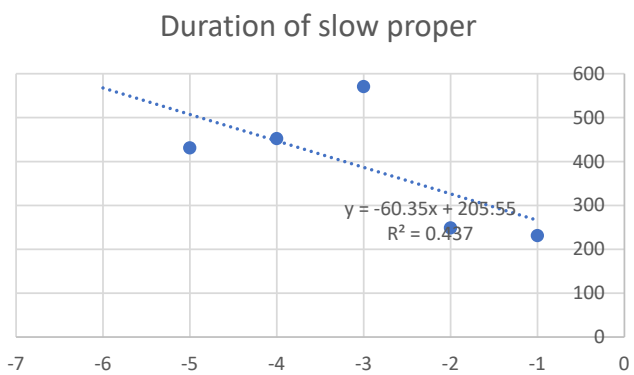

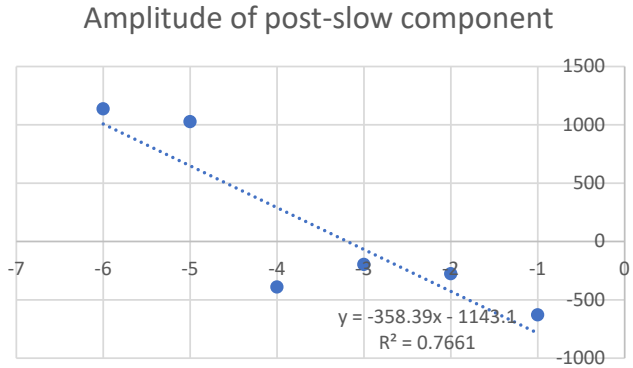

Patient 15 SZ2

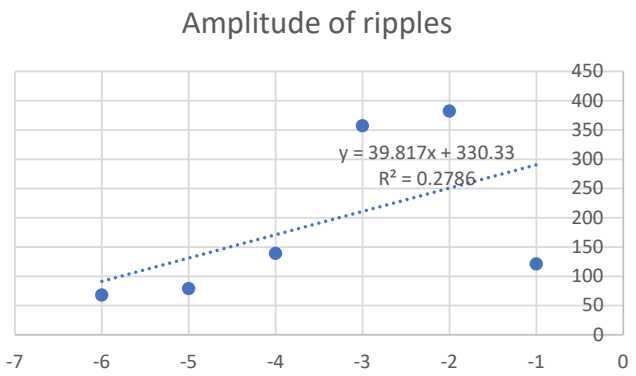

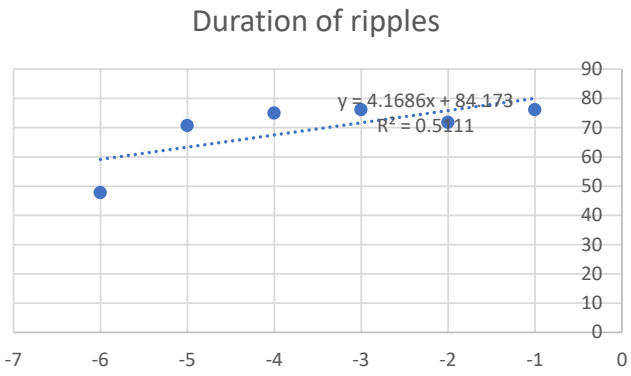

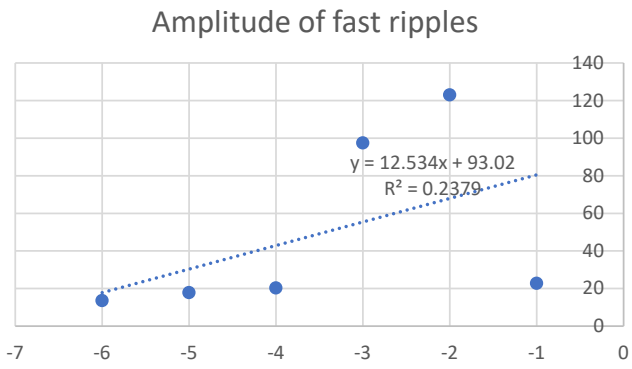

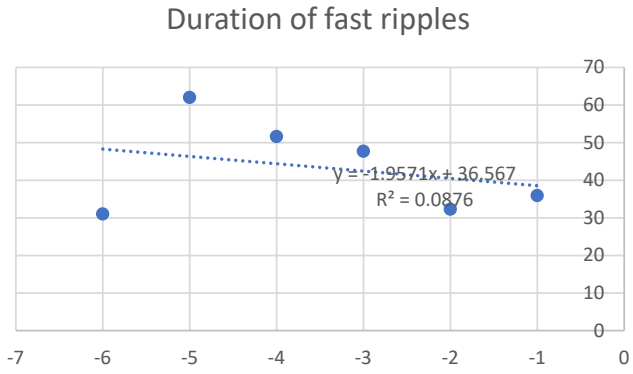

Patient 15 SZ3

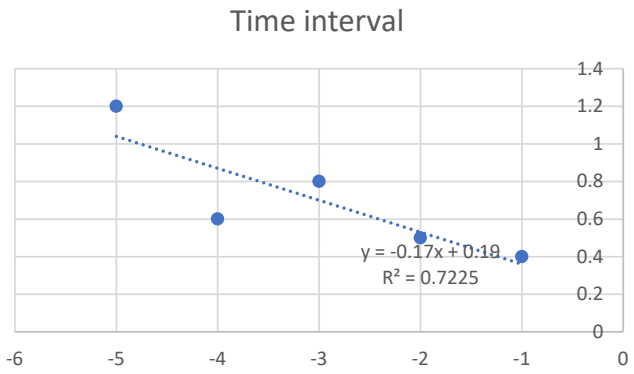

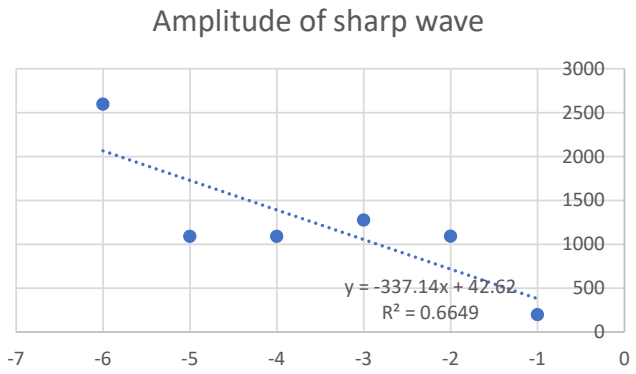

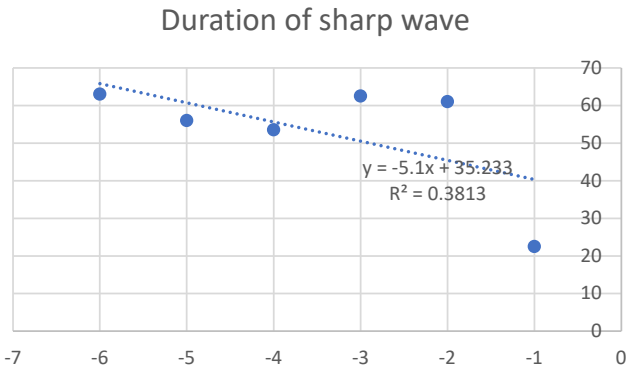

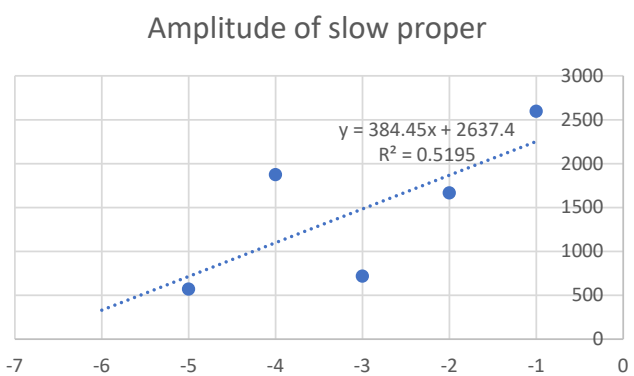

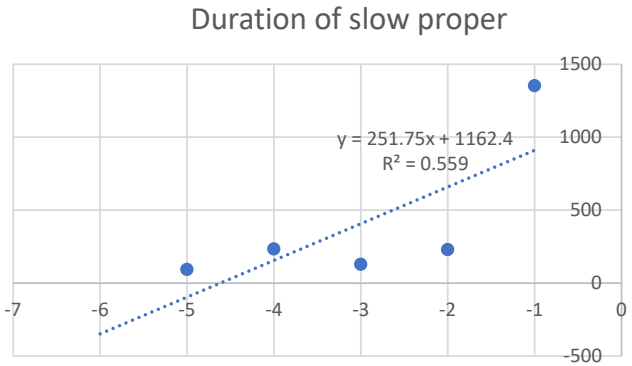

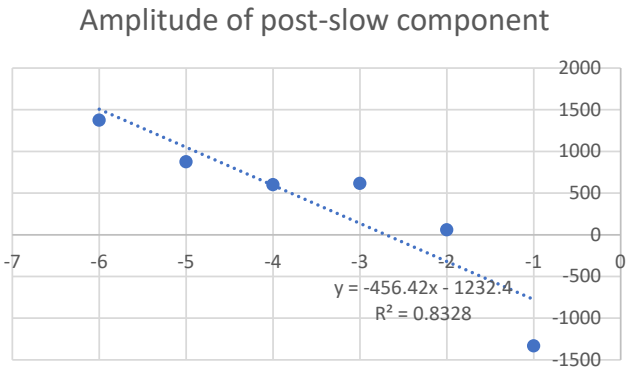

Patient 15 SZ3

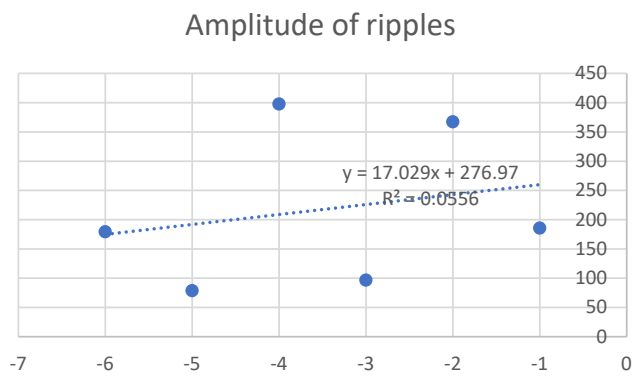

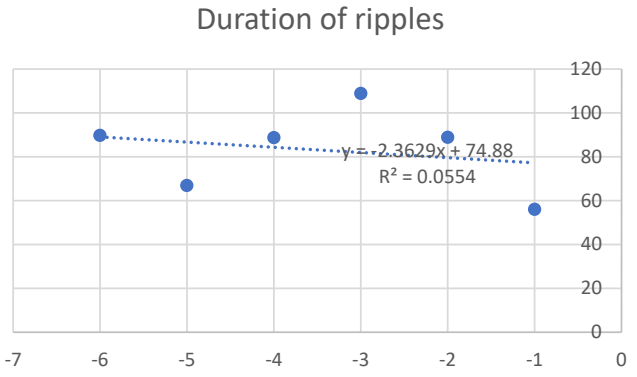

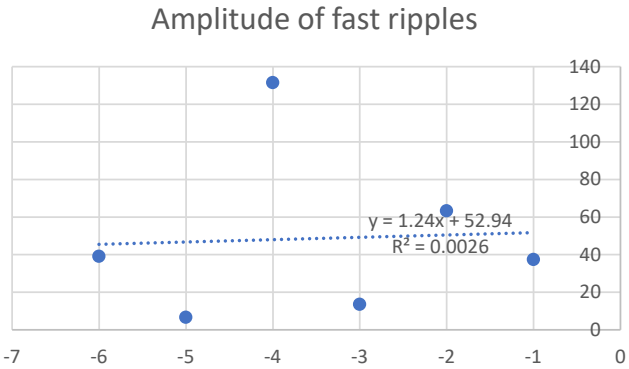

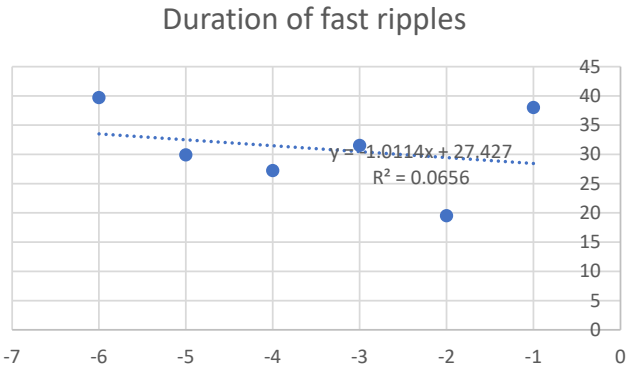

Patient 15 SZ4

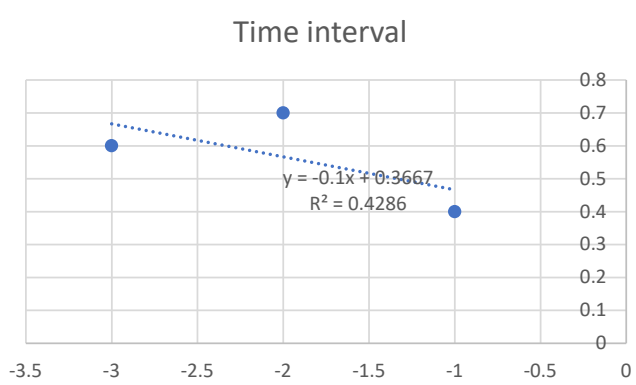

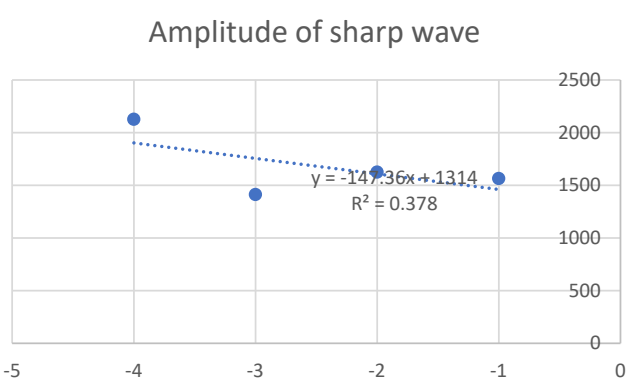

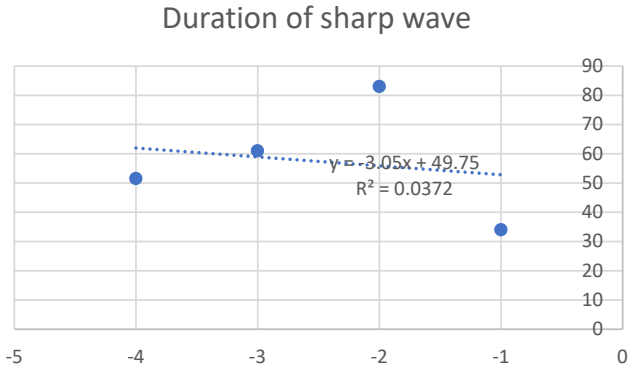

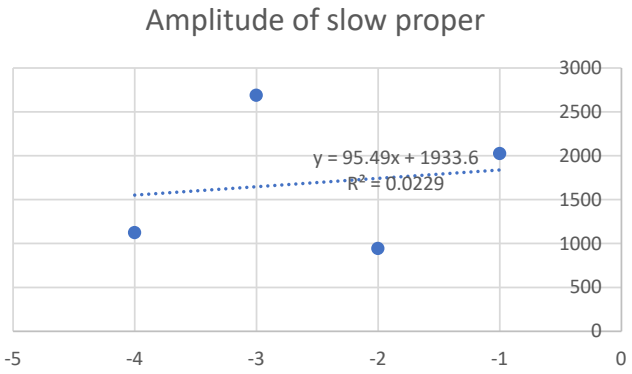

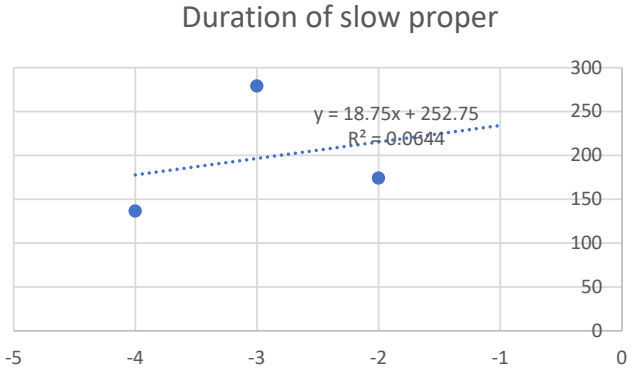

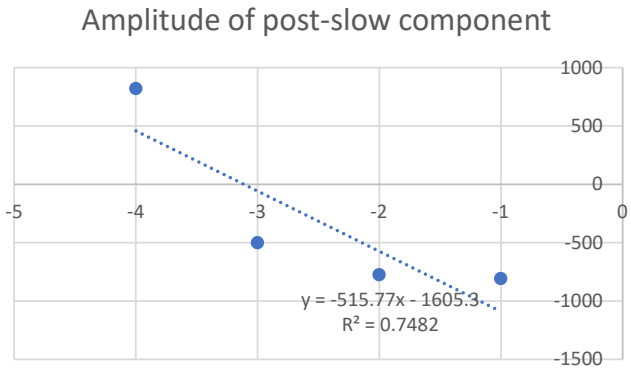

Patient 15 SZ4

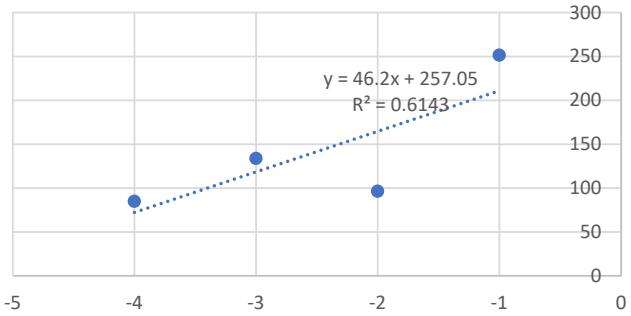

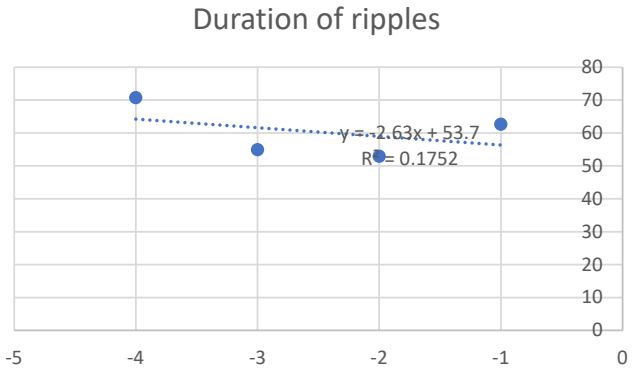

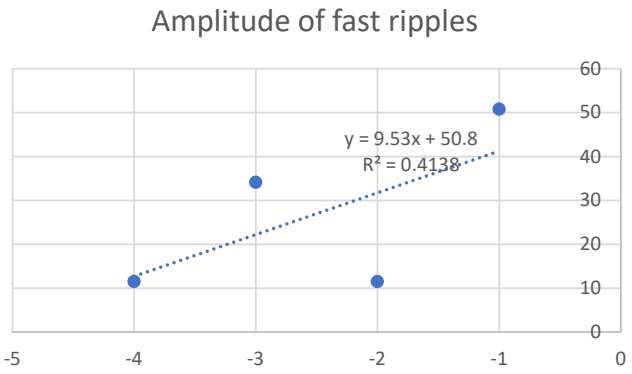

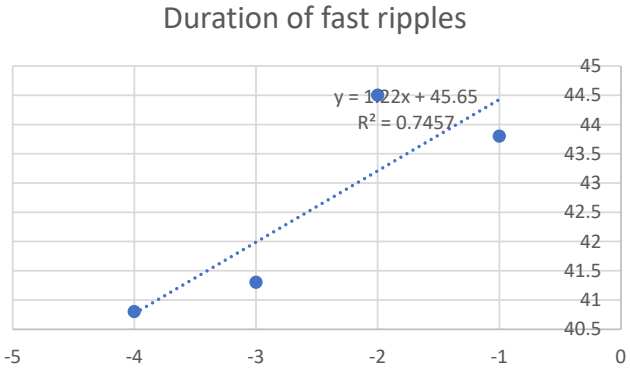

Patient 15 SZ5

Time interval

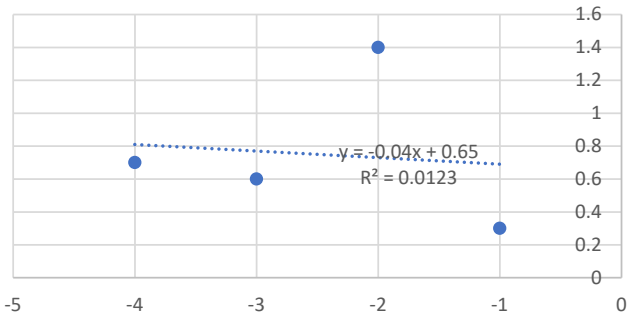

Amplitude of sharp wave

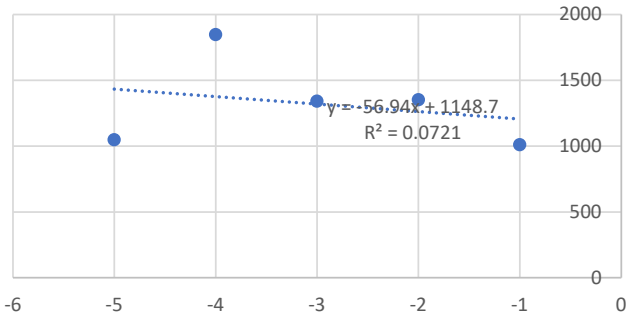

Duration of sharp wave

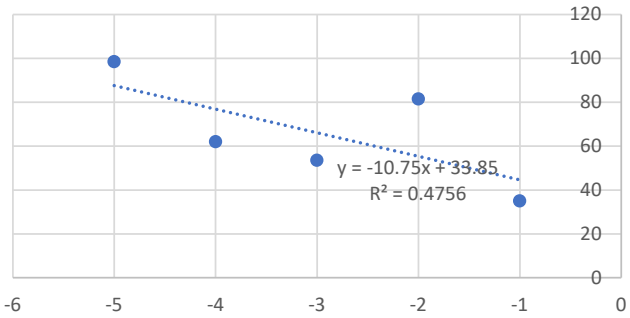

Amplitude of slow proper

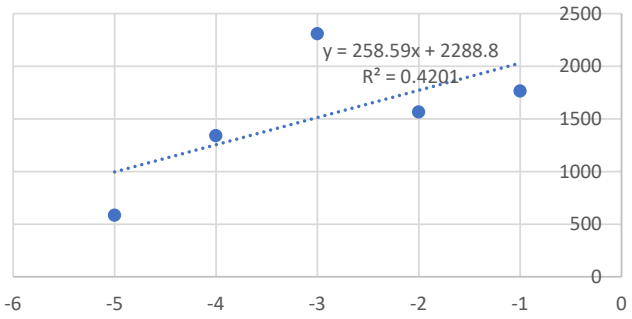

Duration of slow proper

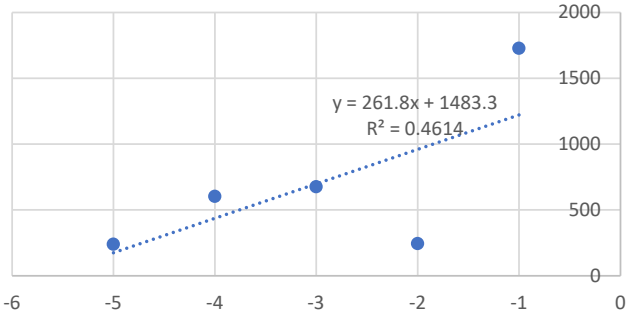

Amplitude of post-slow component

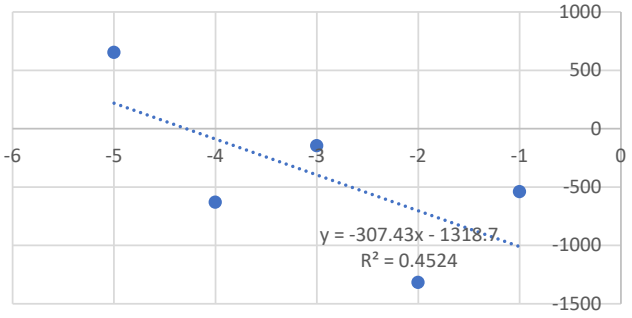

Patient 15 SZ5

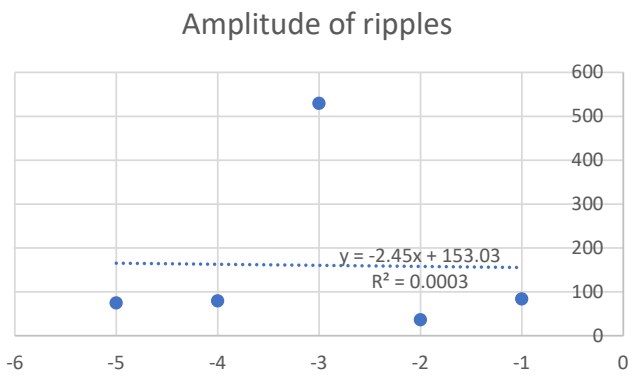

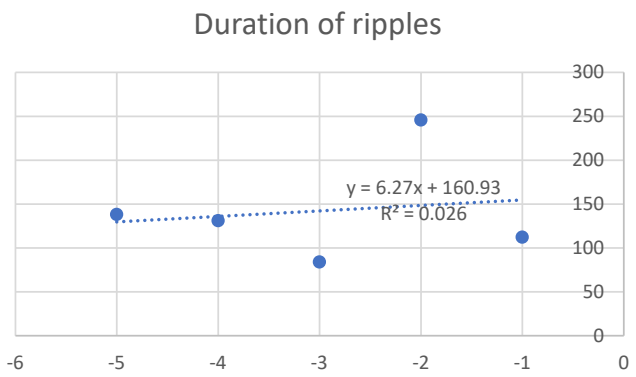

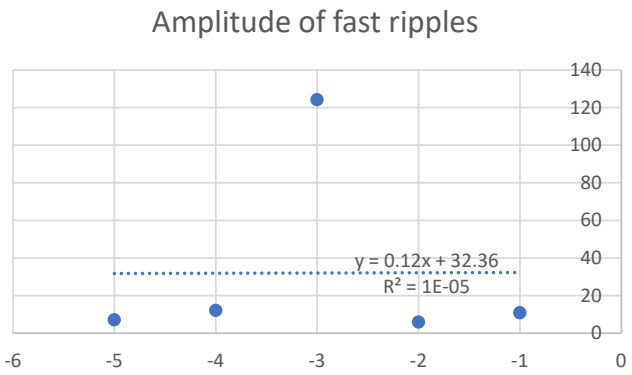

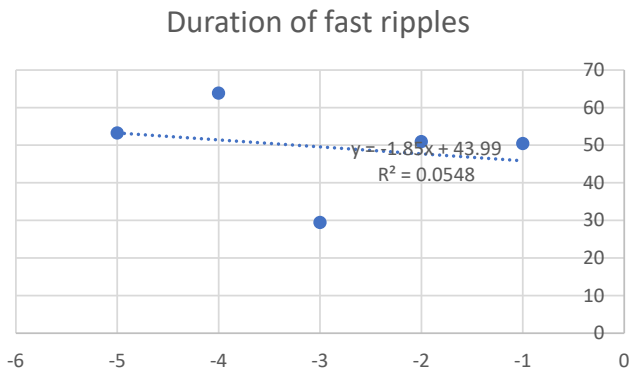

Patient 16 SZ1

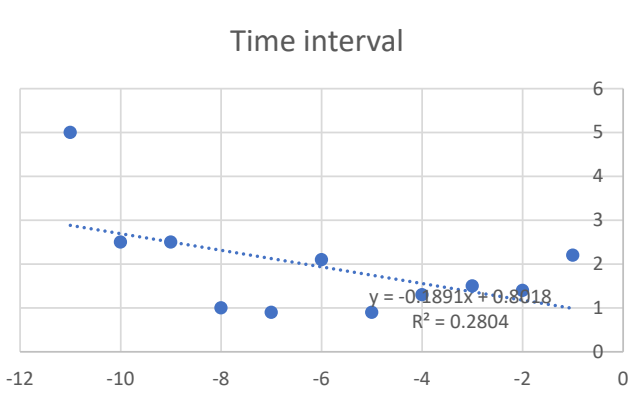

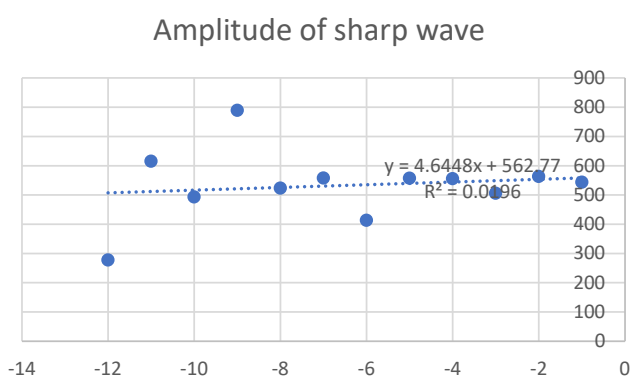

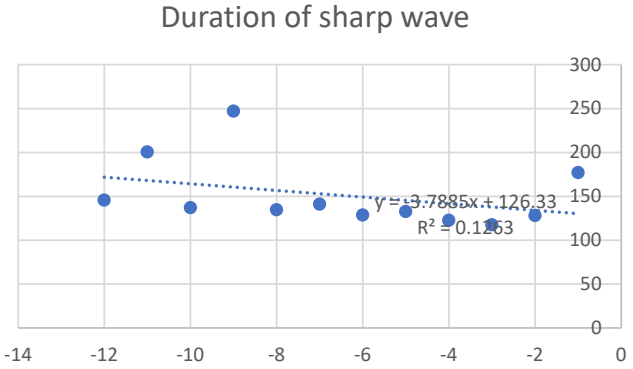

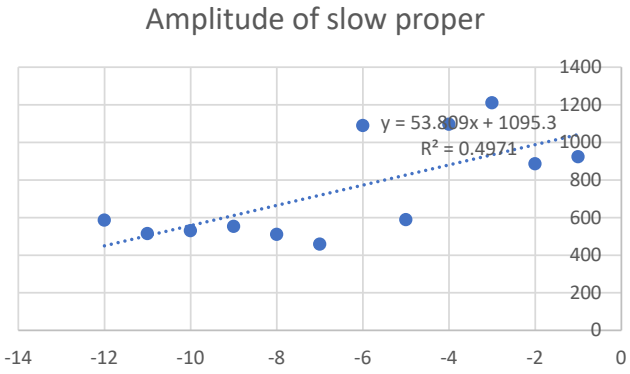

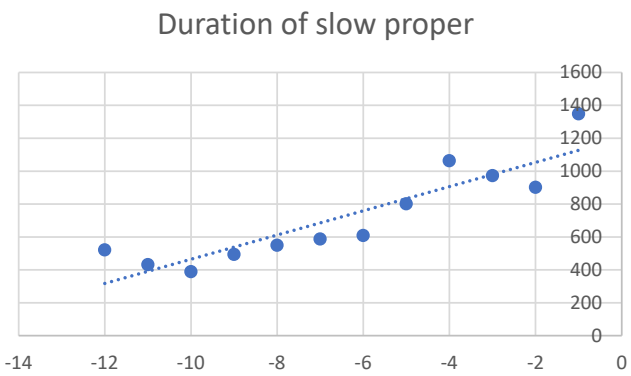

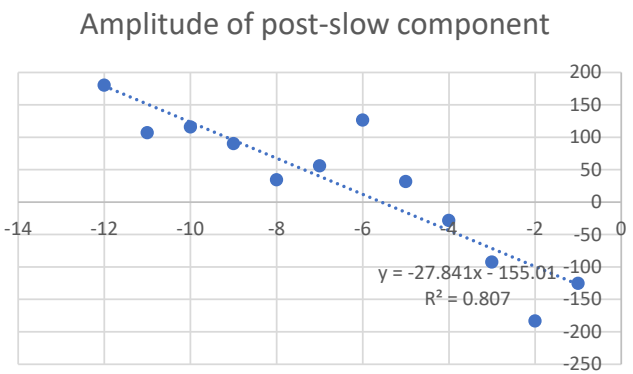

Patient 16 SZ1

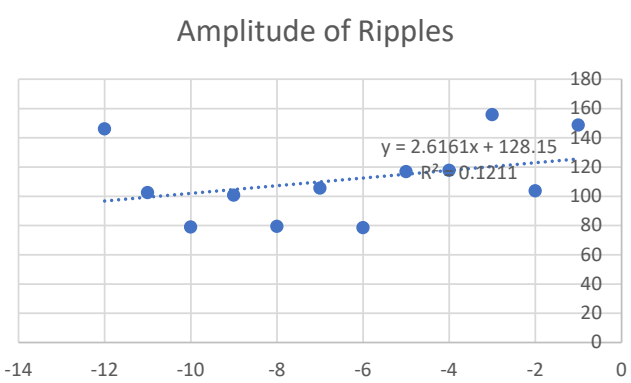

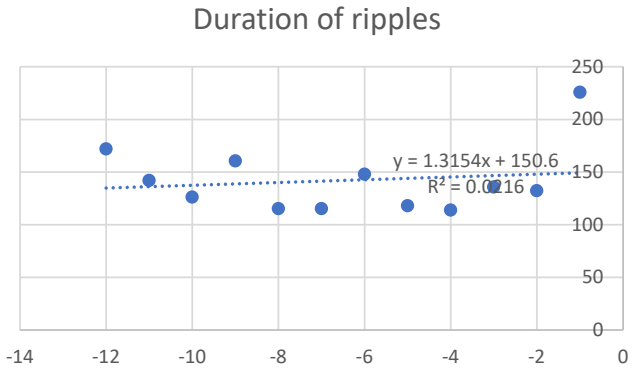

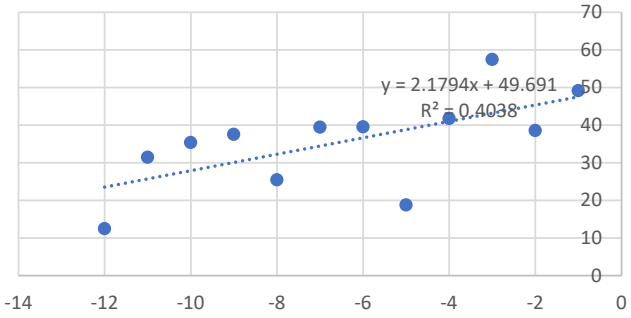

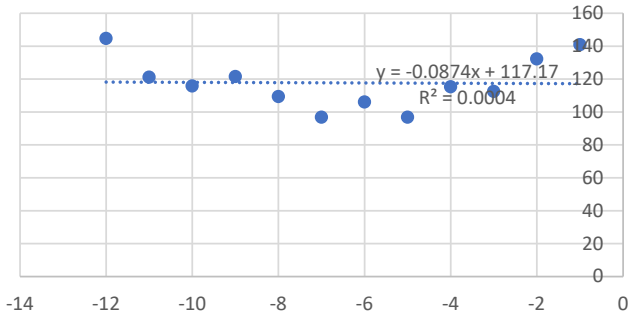

Patient 16 SZ2

Time interval

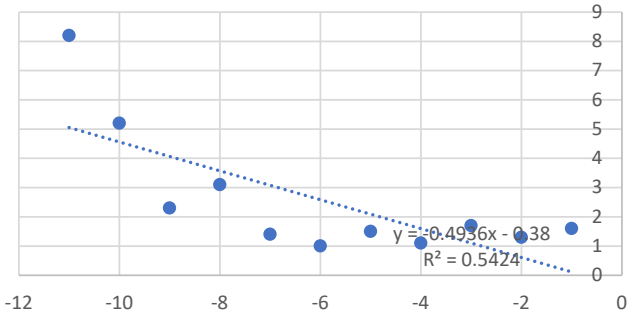

Amplitude of sharp wave

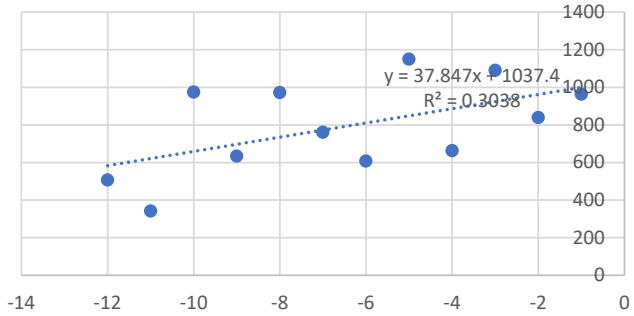

Duration of sharp wave

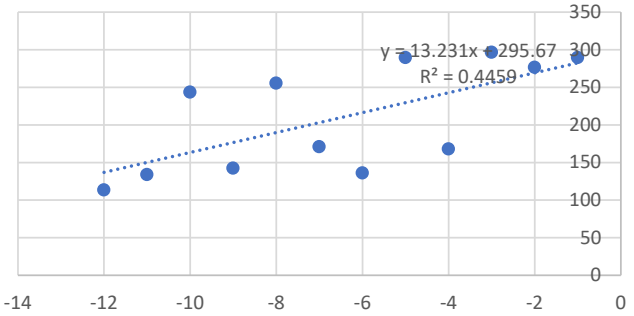

Amplitude of slow proper

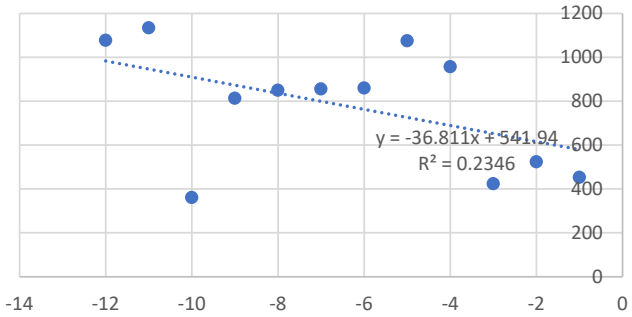

Duration of slow proper

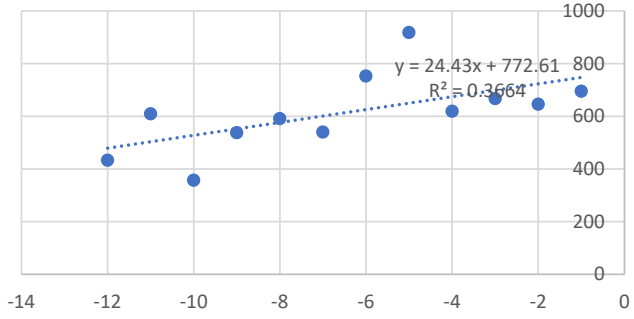

Amplitude of post-slow component

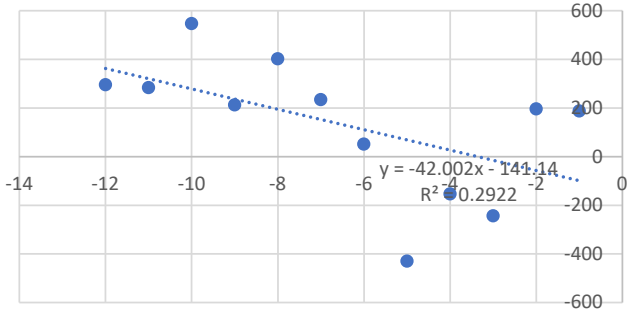

Patient 16 SZ2

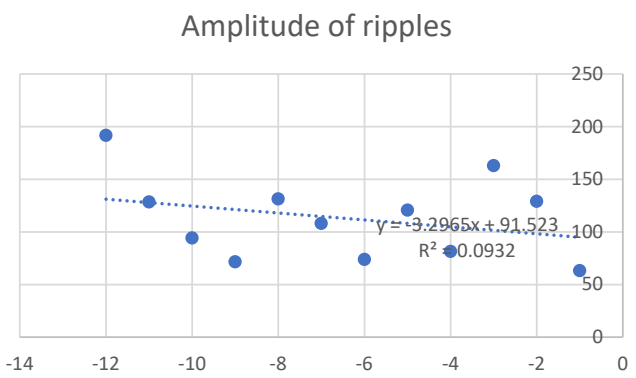

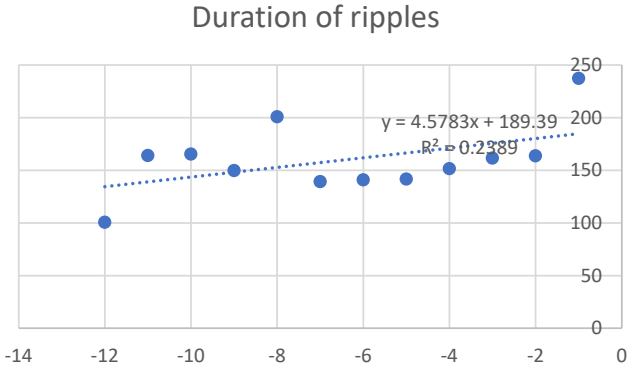

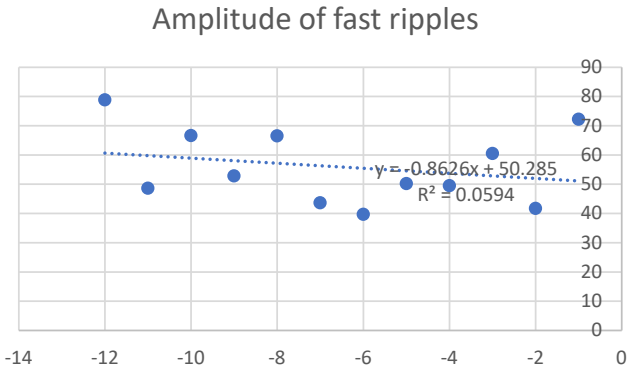

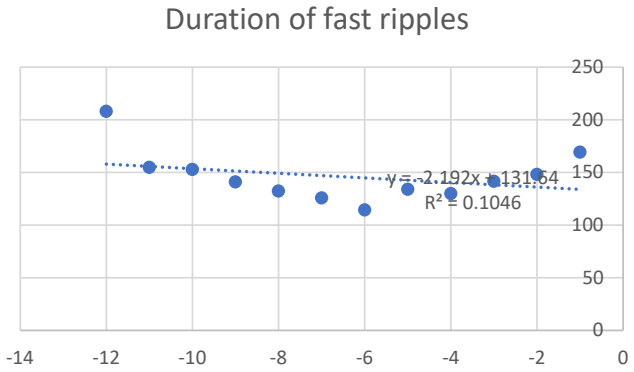

Patient 16 SZ3

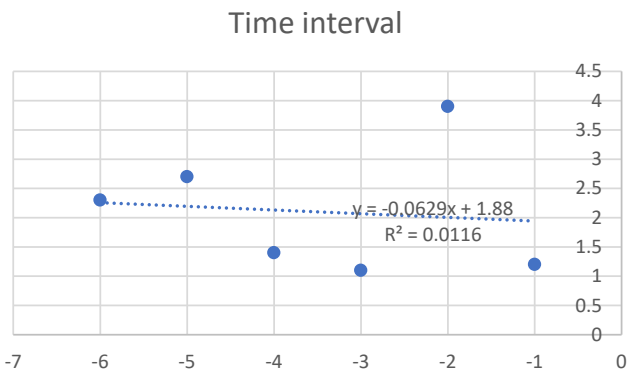

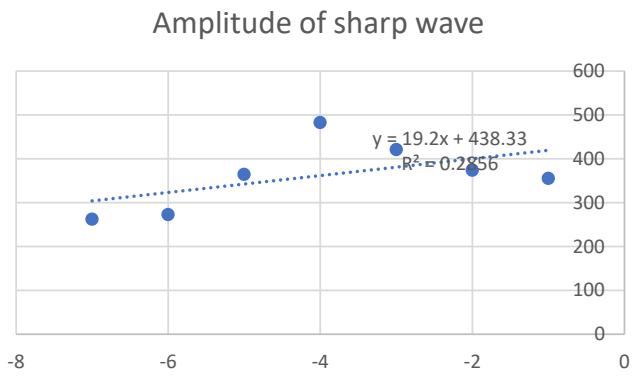

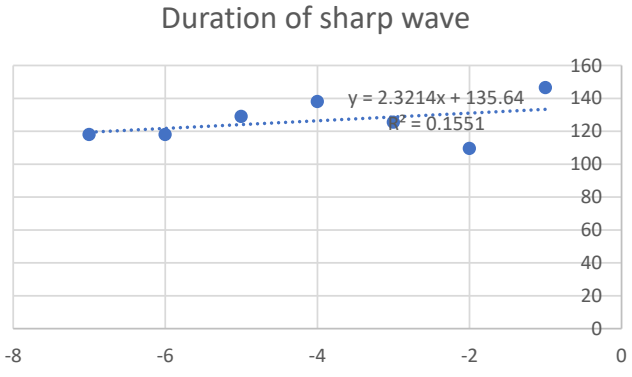

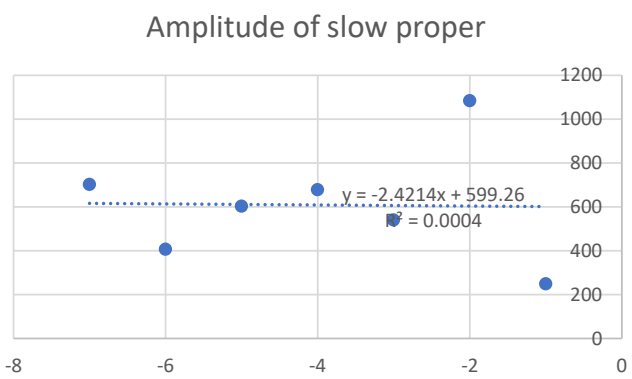

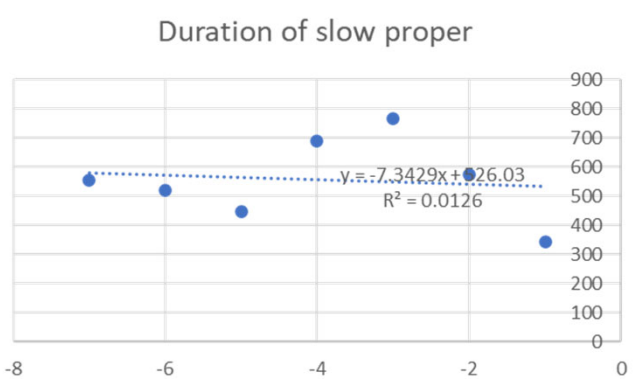

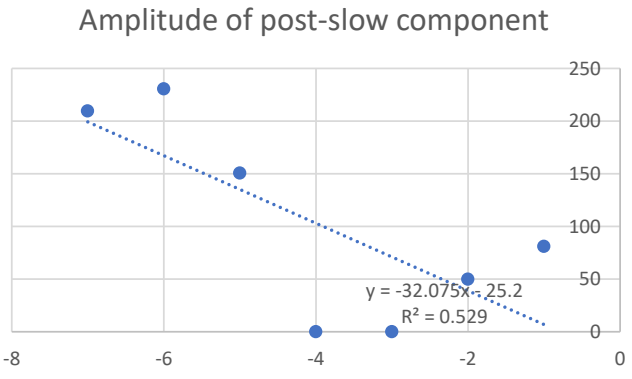

Patient 16 SZ3

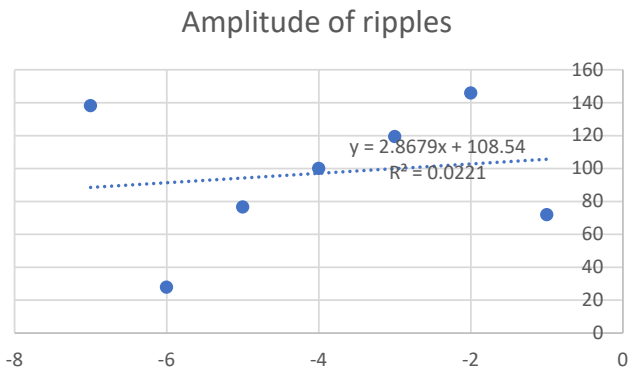

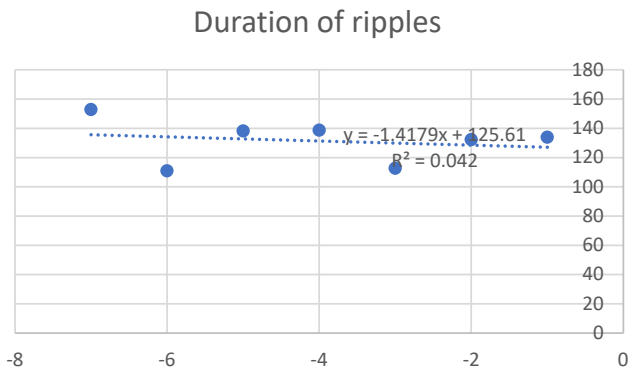

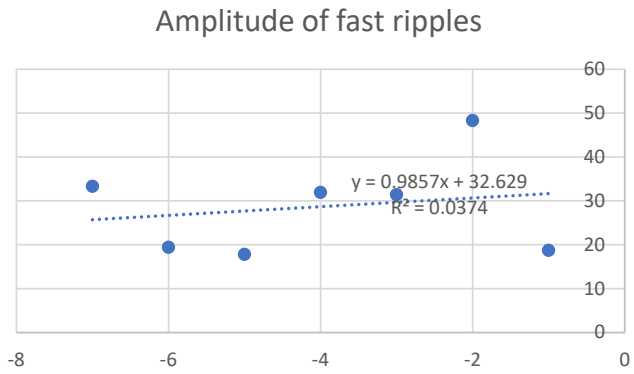

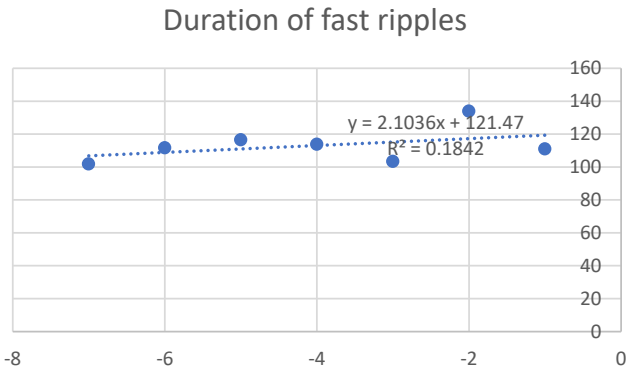

Patient 16 SZ4

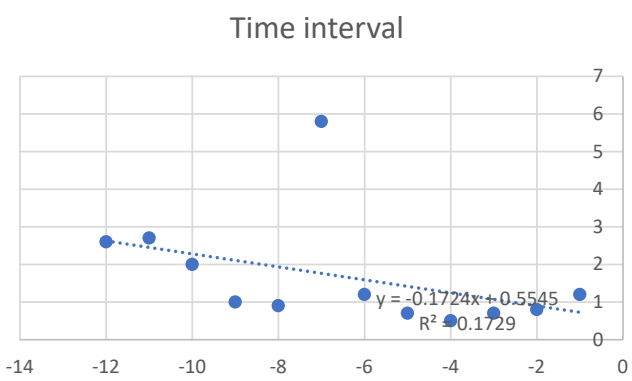

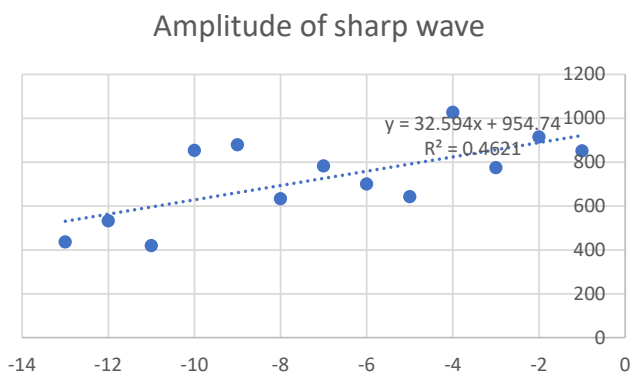

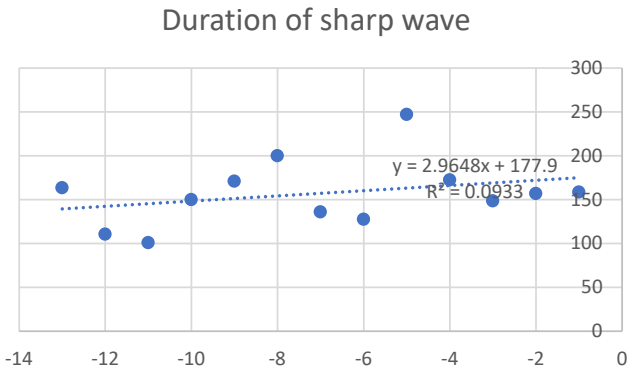

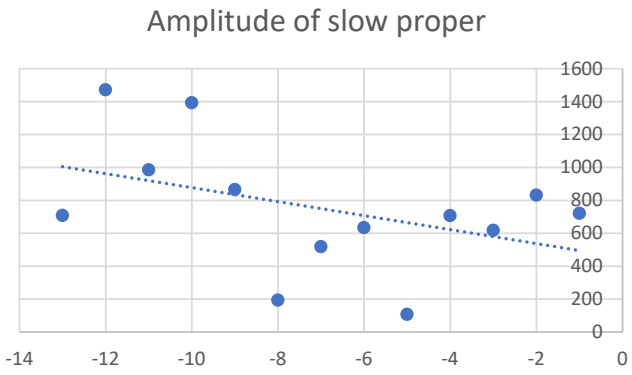

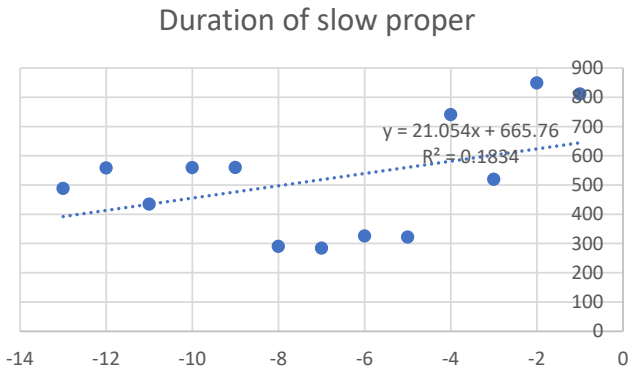

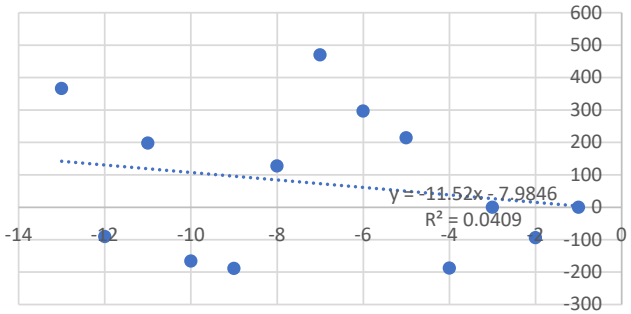

Patient 16 SZ4

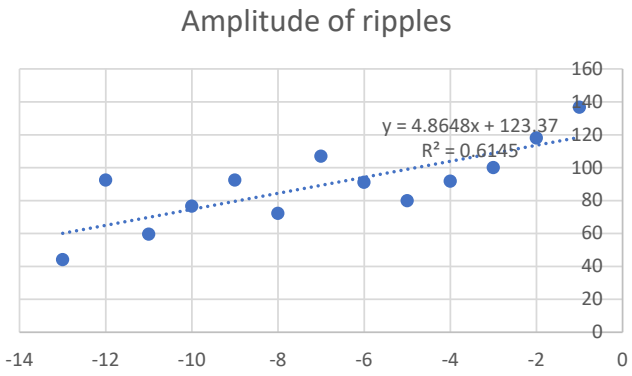

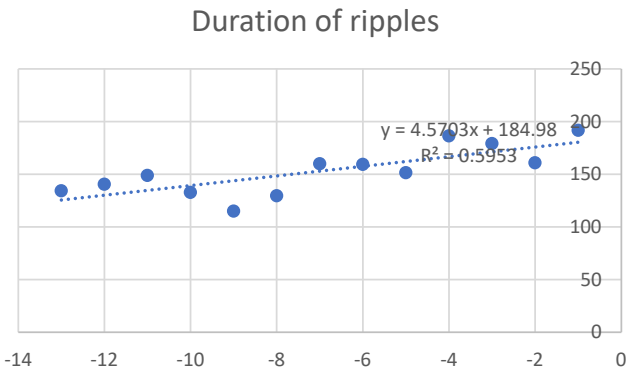

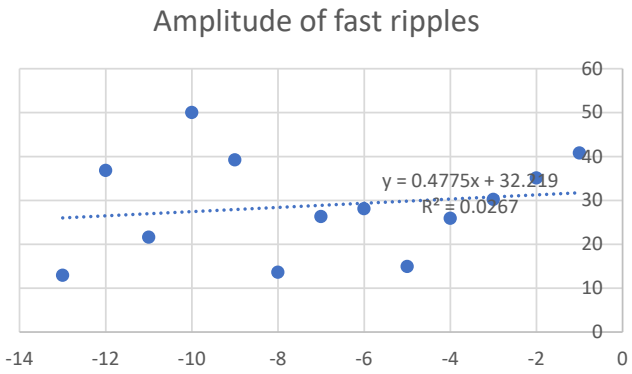

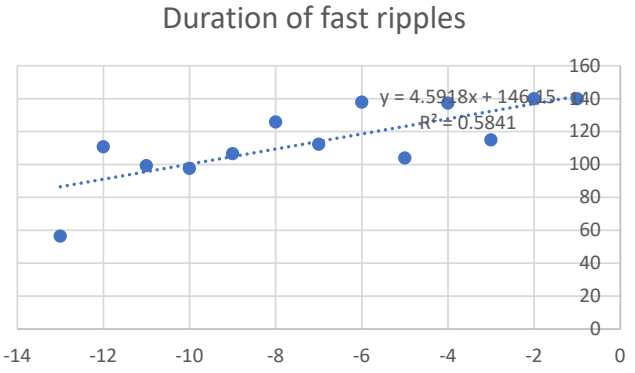

Patient 16 SZ5

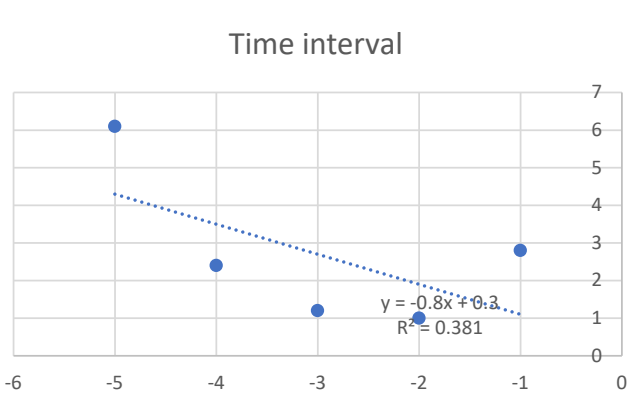

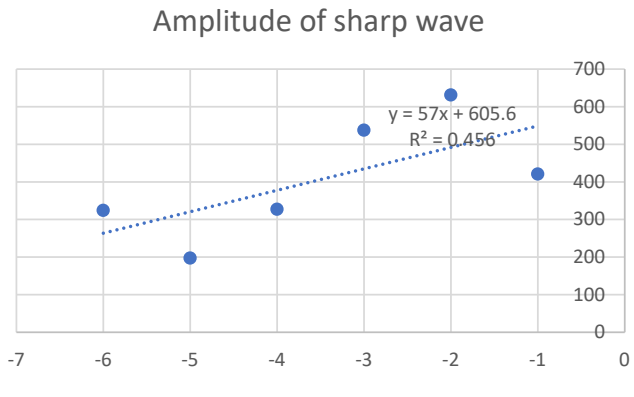

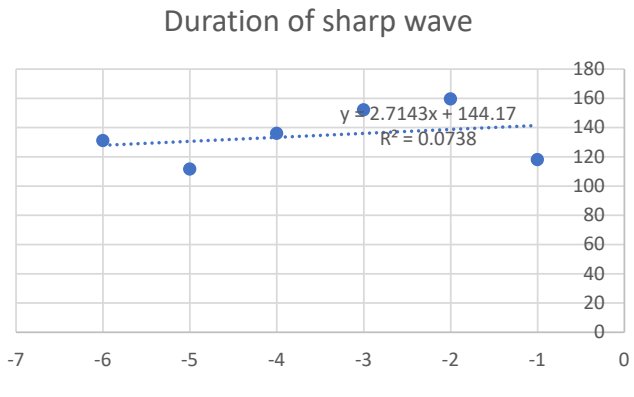

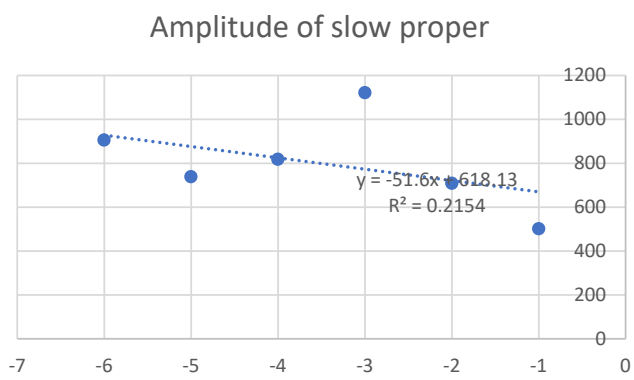

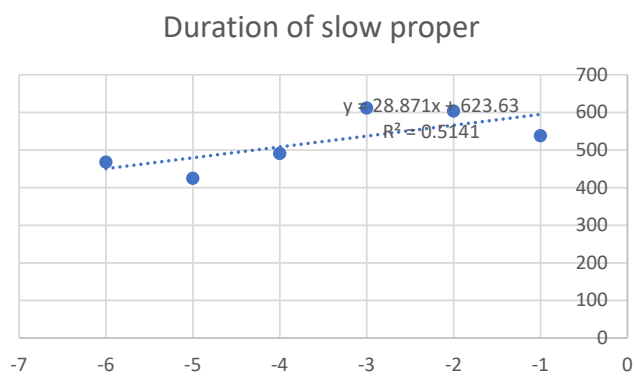

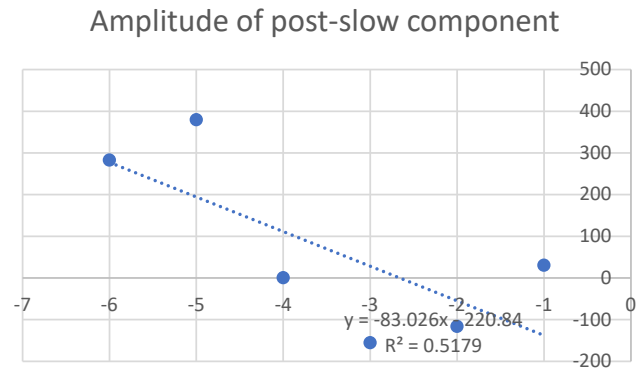

Patient 16 SZ5

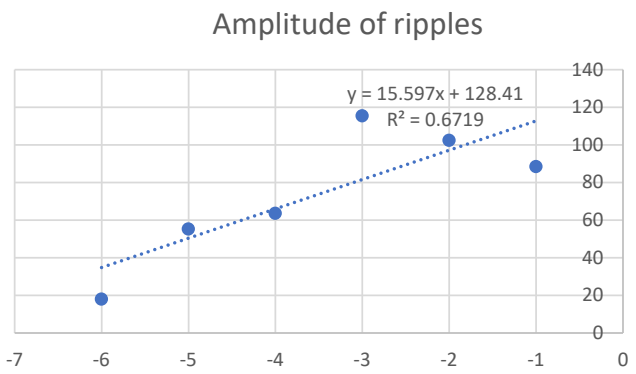

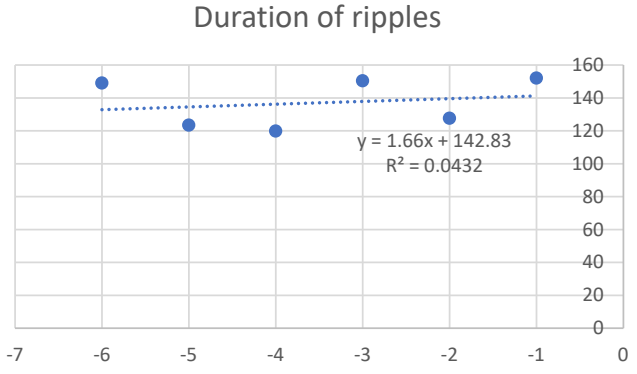

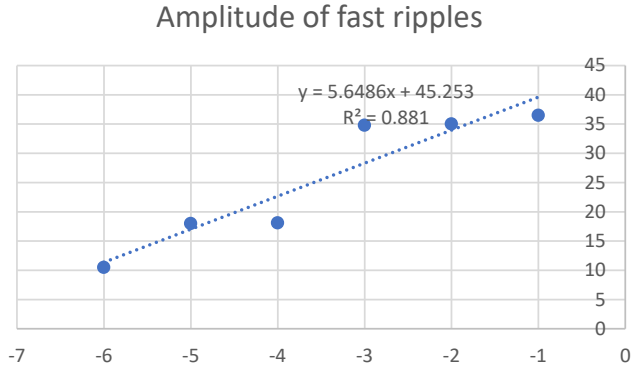

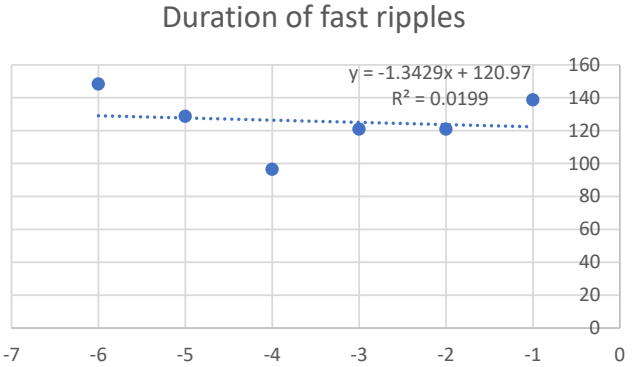

Supplement: Supplementary file 1 [file Data_Sheet_1.PDF]
